# STEM undergraduates’ perspectives of instructor and university responses to the COVID-19 pandemic in Spring 2020

**DOI:** 10.1371/journal.pone.0256213

**Published:** 2021-08-27

**Authors:** Sherry Pagoto, Kathrine A. Lewis, Laurie Groshon, Lindsay Palmer, Molly E. Waring, Deja Workman, Nina De Luna, Nathanial P. Brown

**Affiliations:** 1 Department of Allied Health Sciences, University of Connecticut, Storrs, Connecticut, United States of America; 2 Department of Psychology, The Pennsylvania State University, University Park, Pennsylvania, United States of America; 3 Department of Women’s, Gender, and Sexuality Studies, The Pennsylvania State University, University Park, Pennsylvania, United States of America; 4 Department of Mathematics, The Pennsylvania State University, University Park, Pennsylvania, United States of America; Fondazione Ugo Bordoni, ITALY

## Abstract

**Objectives:**

We examined undergraduate STEM students’ experiences during Spring 2020 when universities switched to remote instruction due to the COVID-19 pandemic. Specifically, we sought to understand actions by universities and instructors that students found effective or ineffective, as well as instructor behaviors that conveyed a sense of caring or not caring about their students’ success.

**Methods:**

In July 2020 we conducted 16 focus groups with STEM undergraduate students enrolled in US colleges and universities (N = 59). Focus groups were stratified by gender, race/ethnicity, and socioeconomic status. Content analyses were performed using a data-driven inductive approach.

**Results:**

Participants (N = 59; 51% female) were racially/ethnically diverse (76% race/ethnicity other than non-Hispanic white) and from 32 colleges and universities. The most common effective instructor strategies mentioned included hybrid instruction (35%) and use of multiple tools for learning and student engagement (27%). The most common ineffective strategies mentioned were increasing the course workload or difficulty level (18%) and use of pre-recorded lectures (15%). The most common behaviors cited as making students feel the instructor cared about their success were exhibiting leniency and/or flexibility regarding course policies or assessments (29%) and being responsive and accessible to students (25%). The most common behaviors cited as conveying the instructors did not care included poor communication skills (28%) and increasing the difficulty of the course (15%). University actions students found helpful included flexible policies (41%) and moving key services online (e.g., tutoring, counseling; 24%). Students felt universities should have created policies for faculty and departments to increase consistency (26%) and ensured communication strategies were honest, prompt, and transparent (23%).

**Conclusions:**

To be prepared for future emergencies, universities should devise evidence-based policies for remote operations and all instructors should be trained in best practices for remote instruction. Research is needed to identify and ameliorate negative impacts of the pandemic on STEM education.

## Introduction

On March 11, 2020, when the World Health Organization declared COVID19 a pandemic [1], colleges and universities around the US swiftly made plans to close their campuses, send students home, and move to emergency remote instruction in a 1–2 week period. This period was rife with confusion and anxiety as many students had difficulty securing housing [2] and others were forced into living situations that were not conducive to remote instruction [3]. Some universities were criticized for how they handled the abrupt move [4]. Once courses went online, the degree of disruption intensified, with some courses more affected than others. Courses in the STEM fields (science, technology, engineering, and math) are inherently difficult to move online with no preparation or instructor training given the frequent use of laboratory experiences, group projects, and the common use of “chalk talks,” all of which present unique challenges and require the use of specialized technologies to conduct remotely. We know little about STEM undergraduate students’ perceptions of how their universities and instructors handled remote instruction under these emergency circumstances. Such insights can inform institutions’ strategies for insuring effective teaching and learning since it is likely that the COVID-19 pandemic will continue to impact higher education well into the 2021–2022 academic year.

As universities shutdown instructors had little time to pivot to remote instruction and their success largely hinged upon their previous knowledge and abilities, availability of training at their institutions, and the time available to be trained. Although much research establishing best practices for remote instruction exists, it has not been well disseminated outside the fields of educational technology and instructional design [5]. A recent survey study of undergraduate students revealed a great deal of variability in course modalities used by instructors in Spring 2020, such that 65% of students surveyed reported recorded lectures, 60% reported live lectures, 55% reported pre-recorded video, and 25% reported breakout groups during a live class [6]. One study found that course workloads also changed during the shutdown, with about one-quarter of students reported that their instructors decreased the workload while one-third reported that their instructors increased the workload [7]. Much variability was also reported in terms of grading in Spring 2020, with 60% of students reporting that they were given a choice between grade and pass/fail, 34% reporting no pass/fail option, and 6% reporting mandatory pass/fail [6]. The same study found that the proportion of students rating their course as somewhat or very satisfying from pre-shutdown to post-shutdown dropped from 87% to 59%; and only 17% said they were satisfied with how much they were learning after the shutdown. The pandemic’s disruption to higher education in the spring appears to have been substantial: 13% of students will delay graduation as a result and 40% lost a job, internship, or job offer [8]. These numbers are sure to rise as the pandemic continues through 2020 and 2021.

The pandemic will affect at least 2 cohorts of undergraduate students in the US. Research on STEM students’ perceptions of the abrupt shift to remote instruction is now needed given the unique challenges associated with conducting STEM courses online and the potential impact on academic performance and retention, as STEM courses are already notorious for “weeding out” students [9]. Research is also needed on STEM students’ perspectives on how their instructors handled the move to remote instruction not only in terms of the tools and technologies that were used, but also in how much instructors conveyed their investment in student success under circumstances that were stressful both for students and instructors.

The variability in course modality, technologies used, instructor preparedness, quality of instruction and grading in Spring 2020 point to a role for universities in developing policies that create a more cohesive approach to remote instruction and for those policies to be guided by the large body of research on online instruction. Given the unprecedented nature of the pandemic, it is unlikely that universities had policies for closing campus or how to implement campus-wide remote instruction and they had little time to create and enforce new policies. Institutional policies are now needed to guide a more seamless transition, but this needs to be data-driven and informed by students. Students’ perspectives of universities responses, including what students felt their university did that was effective and ineffective may be useful to inform policies and the development of university playbooks on how emergency responses can be more evidence-based in the future. Such knowledge could be leveraged in the context of this pandemic, future pandemics, and other emergency situations that force school closures (e.g., wild fires, snowstorms) [10].

To address these needs, in virtual focus groups, we queried a diverse sample of STEM undergraduate students about their perspectives of how their universities and instructors handled campus closures and the move to remote instruction in Spring 2020. Specifically, we asked about strategies, tools, and technologies that universities and instructors used that were effective and ineffective. Then, we asked about instructor behavior that made them feel the instructor cared or did not care about their success. Given the exploratory nature of this work examining an unprecedented event in modern history, we put forth no specific hypotheses.

## Methods

Focus group methodology was selected because very little prior knowledge was available on this topic. In July 2020 we recruited undergraduate students attending US colleges and universities to participate in a study about their experiences in Spring 2020 related to the abrupt transition to remote instruction. Participants completed an online survey [11] that included questions about their demographic characteristics and participated in focus groups conducted via video conferencing software.

### Recruitment

We recruited students through faculty in the Math Alliance, a national organization of faculty in mathematics, by asking them to forward a recruitment email to their students and other faculty teaching courses in the calculus sequence. Recruitment ads were also posted on Reddit in subreddits that targeted student and/or STEM interests and through course listservs. Researchers targeted students within the calculus sequence as these courses are required of most STEM majors. Interested individuals completed a brief online survey to assess eligibility. Eligible students were aged 18 years or older, enrolled full-time in a college or University in the United States in spring 2020, STEM majors, and met criteria for inclusion in one of 16 demographic-stratified focus groups. STEM majors included students who had a declared major within the natural sciences (e.g., biology, physics), engineering, mathematics, and technology but excluded students in the social sciences (e.g., economics, psychology). To create a diverse sample, eligible students were required to fit into one of 16 strata defined by gender, race/ethnicity, and SES (see Table 1). Students whose responses to questions about gender, race/ethnicity, or SES did not place them in one of these strata were excluded from participation. Participants reported their gender as woman, man, or with an ‘other’ write-in option; students who did not identify as women or men were excluded. Participants reported whether they identified their ethnicity as Hispanic/Latinx, and reported how they describe their race(s). Based on responses, we categorized participants as non-Hispanic Caucasian, Hispanic/Latinx (any race[s]), non-Hispanic Black, non-Hispanic Asian, or other race/ethnicity or multiracial; participants identifying as another race/ethnicity or non-Hispanic multiracial were excluded from participation. For socioeconomic status, we categorized students as low SES if they: 1) were eligible for work study or Pell grants, 2) reported that it was hard for their family to pay for basics (e.g., rent, heat, food), and/or 3) reported that parental education was less than or equal to a high-school diploma or GED and a household income of less than $75,000. Students were categorized as higher SES students as students whose parental education was a Bachelor’s degree or higher, who were not eligible for work study, were not eligible for a Pell Grant, and reported that it was not hard at all or somewhat hard to pay for basics. Students whose responses to questions about financial aid, parental education, household income, and financial strain did not place them in either of the above groups were excluded from the study.

**Table 1:** Characteristics of undergraduate students majoring in STEM fields at US colleges/universities who participated in focus groups in summer 2020, n (%).

Age (years)	
18 years	15 (26)
19 years	25 (42)
20 years	11 (19)
21 years	4 (7)
22 years	2 (3)
36 years	1 (2)
Gender	
Female	30 (51)
Male	29 (49)
Race/ethnicity	
Non-Hispanic white	14 (24)
Non-Hispanic Middle Eastern	2 (3)
Non-Hispanic Black	14 (24)
Hispanic (any race)	12 (20)
Non-Hispanic South East/East Asian	6 (10)
Non-Hispanic South Asian	11 (19)
Socioeconomic status (SES)*	
Low SES	28 (47)
Higher SES	31 (53)
Highest parental education	
High school/GED	8 (14)
Trade/tech/some college/Associate’s degree	5 (8)
Bachelors or higher	46 (78)
Hard for family to pay for basics	
Not at all hard	40 (68)
Somewhat hard	19 (32)
Very hard	--
Eligibility for federal aid programs	
Work study	14 (24)
Pell grant	16 (27)
Other need-based loans or grants (e.g., Stafford Loans)	10 (17)
Class rank in college in Spring 2020	
1^st^ year/freshman	40 (68)
2^nd^ year/sophomore	14 (24)
3^rd^ year/junior	5 (8)
4^th^ year/senior	--
University/college characteristics	
Public	54 (91)
Private	5 (9)
Minority serving	25 (42)

Of the 416 respondents to the initial eligibility screener, we emailed the 154 who were eligible and recruited participants into gender-SES-race/ethnicity focus groups with a cap of 6 students per focus group. A total of 59 students from 34 institutions participated in one of 16 focus groups ranging in size from 2–6. Of the 34 institutions represented in the sample, 88% were public, 12% were private, and 38% were minority serving. Institutions had a mean of 30.14% of students on Pell grants (sd = 13.10). Participants received a $50 gift card for participating. The Penn State University (00014866) and University of Connecticut (L20-0060) Institutional Review Boards approved this study. Participant characteristics are shown in Table 1.

### Focus groups

Focus groups were stratified by gender (male, female), socioeconomic status (SES; low, moderate/high), and race/ethnicity (non-Hispanic Caucasian, Hispanic/Latinx, non-Hispanic Black, non-Hispanic Asian) so that participants did not experience discomfort expressing the challenges they experienced during emergency remote instruction due to being in a group with participants of differing social class, gender, or race. We conducted 16 focus groups, one group with each gender x SES x race/ethnicity group (e.g., non-Hispanic Black women of moderate/high SES). Thematic saturation was achieved with 16 focus groups. Focus groups lasted 60 minutes and were conducted by an investigator (NB, LP, KL, ND, or DW) who was paired with a note taker. To keep the focus groups as uniform as possible, the facilitator followed a script that was produced by the investigator team which reflected multiple disciplines and career stages, including 3 professors (mathematics, clinical psychology, epidemiology), 2 graduate students (gender studies, social psychology), and 2 undergraduate STEM majors. Through discussion, the team developed a list of questions about the strategies instructors used and how instructors behaved towards students during the move to remote instruction as well as what universities did and how they could have done better. The discussion drew upon each team member’s experiences and observations during this unprecedented historical event and the goal was to produce responses that could inform tangible policy changes. No research was available at the time to inform the focus group script S1 File. The final focus group script posed the following questions:

1. What are some examples of strategies, tools, or technologies that your instructors used that you found to be very effective (in that they made it easier to learn) during remote instruction?
2. What are some examples of strategies, tools, or technologies that your instructors used that you found to be ineffective (in that they did not help you learn) during remote instruction?
3. What are some things your instructors did during remote instruction that made you feel like they cared about their students?
4. What are some things your instructors did during remote instruction that made you feel like they did not care about their students?
5. What are some things that your university did to help students be successful during remote instruction?
6. What do you wish your university did to better help students be successful during remote instruction?

During the focus group, each participant was given a turn to respond to each question but they were not specifically asked to react to each other’s responses. When a participant’s response was cryptic, the facilitator probed for clarification. Participants were given the option of providing no response if they could not think of an answer to the question (e.g., if they found no professor strategies helpful they could say “none”).

### Analysis

Transcription software was used to produce transcripts that were then reviewed and edited for accuracy by research assistants S2 File. We summarized participant characteristics using descriptive statistics. We conducted a conventional content analysis using a data-driven inductive “framework” approach to coding the content into major and minor themes for each of the six focus group questions [12]. As a first step, a pair of investigators, including one who was present during the focus groups and one who was not, read through the transcripts (*familiarization*) to identify emerging themes (*identifying a thematic framework*). They then developed a codebook that was then applied to all of the data (*indexing*). Once each coder finished independent coding they met in pairs to resolve discrepancies as described elsewhere. [13] A third investigator was brought in for unresolved discrepancies. This process resulted in a total of 46 themes across 6 questions. Interrater agreement ranged from 81% to 100% and Cohen’s kappa statistics ranged from 0.202 to 1.0, with only 2 of the 46 having a kappa of < .5. This exceeds the recommended standard of 80% agreement on 95% of codes [14].

Data management and descriptive analyses of the survey data were conducted using SAS 9.4 (SAS Institute, Inc, Cary, NC) while interrater reliability statistics were calculated with SPSS 26 (IBM, Armonk, NY).

## Results

Focus group participants (N = 59) had a median age of 19 years old, 51% were women, 47% were low SES, and the sample was racially/ethnically diverse (Table 1). In Spring 2020, participants were enrolled in 29 US colleges/universities, including private colleges/universities (n = 4, 14% of schools), public universities (n = 23, 79%), and community colleges (n = 2, 7%). When surveyed in July 2020, students were living in 20 US states/territories including Puerto Rico, with two students living outside the US. The distributions of responses to each question by the 16 race/ethnicity, gender, and SES subgroups are depicted in Table 2. The representation of the 16 subgroups for each theme of each question are depicted in the S1 Table.

**Table 2:** Characteristics of focus groups and distribution of responses by subgroup.

	N	# of institutions	Effective instructor strategies	Ineffective instructor strategies	Instructor caring behavior	Instructor uncaring behavior	What universities did well	What universities could have done better
			Total responses	Total responses	Total responses	Total responses	Total responses	Total responses
**Women**								
Black low SES	3	3	7 (7.5%)	4 (5.9%)	6 (6.9%)	4 (5.5%)	8 (6.9%)	6 (8.7%)
Black high SES	4	3	5 (5.4%)	4 (5.9%)	5 (5.7%)	5 (6.9%)	9 (7.8%)	5 (7.2%)
White low SES	5	5	8 (8.6%)	5 (7.4%)	7 (8.0%)	6 (8.3%)	8 (6.9%)	6 (8.7%)
White high SES	4	4	6 (6.5%)	6 (8.8%)	4 (4.6%)	4 (5.5%)	7 (6.0%)	5 (7.2%)
Hispanic low SES	4	4	6 (6.5%)	4 (5.9%)	4 (4.6%)	5 (6.9%)	7 (6.0%)	6 (8.7%)
Hispanic high SES	2	1	3 (3.2%)	3 (4.4%)	4 (4.6%)	3 (4.1%)	6 (5.2%)	2 (2.9%)
Asian low SES	5	5	7 (7.5%)	3 (4.4%)	8 (9.2%)	7 (9.7%)	8 (6.9%)	5 (7.2%)
Asian high SES	3	3	6 (6.5%)	3 (4.4%)	4 (4.6%)	3 (4.1%)	8 (6.9%)	4 (5.8%)
**Men**								
Black low SES	2	2	4 (4.3%)	3 (4.4%	3 (3.4%)	3 (4.1%)	8 (6.9%)	3 (4.3%)
Black high SES	5	5	8 (8.6%)	7 (10.3%)	9 (10.3%)	6 (8.3%)	9 (7.8%)	5 (7.2%)
White low SES	3	2	2 (2.1%)	4 (5.9%)	6 (6.9%)	2 (2.8%)	4 (3.4%)	2 (2.9%)
White high SES	4	2	7 (7.5%)	5 (7.4%)	9 (10.3%)	4 (5.5%)	8 (6.9%)	4 (5.8%)
Hispanic low SES	2	2	1 (1.1%)	3 (4.4%)	2 (2.3%)	3 (4.2%)	2 (1.7%)	2 (2.9%)
Hispanic high SES	4	3	4 (4.3%)	4 (5.9%)	4 (4.6%)	4 (5.5%)	7 (6.0%)	5 (7.2%)
Asian low SES	4	4	9 (9.7%)	4 (5.9%)	6 (6.9%)	6 (8.3%)	8 (6.9%)	4 (5.8%)
Asian high SES	5	5	10 (10.8%)	6 (8.8%)	6 (6.9%)	7 (9.7%)	9 (7.8%)	5 (7.2%)
**Total**	59	53	93	68	87	72	116	69

### Effective instructor strategies, tools, and technologies

The 59 participants provided 93 responses to the question: “What are some examples of strategies, tools, or technologies that your instructors used that you found to be very effective (in that they made it easier to learn) during remote instruction?” Two major themes and three minor themes emerged in responses (Table 3). The most common (35%; n = 33) response referred to hybrid instruction, meaning the instructor’s use of both synchronous and asynchronous modalities. For instance, many participants preferred live remote lectures that were also recorded and posted, allowing students who experienced disruptions during the live portion to watch later at their convenience. The next most common theme (27%; n = 25) referred to instructors’ use of multiple tools to reach and engage students, such as discussion boards, study groups (e.g., in breakout rooms), or supplementary materials like making lecture notes or slides available to students. Specific technologies mentioned include communication platforms (e.g., Piazza, Discord, Nectir, Gauchospace, Slack, GroupMe, Blackboard Collaborate Ultra) and streaming/video conferencing platforms (e.g., Zoom, Boing, Microsoft Teams, Twitch). Minor themes included instructor’s communication strategy (15%; n = 14) such as quick and clear email responses to student inquiries; recorded lectures (12%; n = 11); leniency (5%; n = 5); and live lectures at the regularly scheduled class times (3%; n = 3). Finally, two responses (2%) indicated there was nothing the student found particularly effective.

**Table 3:** Effective instructor strategies, tools, and technologies (N = 59 participants; N = 93 responses).

Theme	Responses N (%)	Illustrative examples
**Hybrid Instruction:** combination of asynchronous and synchronous instruction	33 (35%)	“Yeah, the two class styles I liked was some would do live lectures and then posted afterwards so you’d have the opportunity, or you’d have the option of viewing it live or later, and others would post the recorded lecture before class. And then they would open up class for like an office hours session where you can just talk to them live and get questions answered. I think both of those were effective.”
**Multiple Resources:** use of supplementary materials, discussion boards, study groups, break-out rooms, or review sessions in addition to lectures.	25 (27%)	“PROBLEM SOLVING TOGETHER IN BREAKOUT ROOMS ARE THE ABSOLUTE BEST.” [Emphasis in original]
		“They opened discussion spaces via Nectir or Gauchospace.”
**Communication:** timely, clear communication from instructors	14 (15%)	“They responded to emails a lot quicker than they did during the year.”
		“Our instructor used to mail us regularly with what to do and how much we needed to do and complete by the week.”
**Pre-recorded lectures:** preferred pre-recoded lectures over other instructional modalities	11 (12%)	“The one thing I did find that was a positive change was…pre recorded lectures.”
		“Instructors often pre-recorded lectures which allowed us to watch them at our convenience.”
**Leniency:** more lenient with grading, assignment due dates, or course content	5 (5%)	“Some instructors sort of were more lenient. Gave more extra credit, stuff like that.”
		“You can do open notes on your test, which was, I mean, yeah, that was definitely helpful.”
**Keeping the same schedule:** preferred courses that kept the same schedule	3 (3%)	“I liked when they posted their class at the same time as it normally met, because it helped me keep on a schedule.”
**Nothing:** did not mention anything that they found effective	2 (2%)	“During remote instruction, some things my instructors have been doing haven’t been too special.”

### Ineffective instructor strategies, tools, and technologies

The 59 participants made a total of 68 responses to the question: What are some examples of strategies, tools, or technologies that your instructors used that you found to be ineffective (in that they did not help you learn) during remote instruction? About 1/5 of students (n = 14; 21%) had no examples of ineffective strategies, tools, and technologies to share. Otherwise, 5 major themes and 1 minor theme emerged (Table 4). The most common theme (n = 12; 18%) encompassed any strategy that instructors used that served to increase the difficulty level or workload of the course relative to how the course was conducted pre-shutdown. Such measures included increasing the weighting of proctored exams to reduce the impact of cheating on final grades, adding new tasks such as participation on discussion boards, or changing exam and assignment formats in ways that would avoid cheating but also increase the difficulty level for students (e.g., open-ended responses instead of multiple choice or replacing exams with time intensive projects). The next most common theme (n = 10; 15%) was instructors using pre-recorded lectures. Responses cited that pre-recorded lectures that were used in place of live lectures made it harder to feel motivated to attend and harder to learn from given the lack of opportunity to ask questions in the moment. An equally prevalent theme (n = 10; 15%) was instructors’ lack of a communication strategy, tool, or technology, with examples including no formal ways to interface with the instructor or poor email responses. Another major theme (n = 9; 13%) was the use of any technologies that had frequent technical difficulties, were inefficient, required bandwidth or computer storage that not all students had, or that required more training than was provided. Examples included test taking platforms in which students experienced technical difficulties that interfered with test performance and/or the time available to take the test, dissemination of large files that students didn’t have the ability to download, use of breakout rooms with little guidance on how students should utilize the time, and the use of online tests that didn’t allow students to skip difficult questions and return to them later. The next theme (n = 8; 12%) was related to instructors’ approaches to lecturing, including the use of long-form recorded or live remote lectures, posting outdated lecture recordings, and use of poor quality recordings. One minor theme was instructors requiring attendance at live lectures (n = 4; 6%). One response was coded as “other” because it did not fit into any of these categories.

**Table 4:** Ineffective instructor strategies, tools, and technologies (N = 59; N = 68 responses).

Theme	N (%)	Illustrative examples
**Increasing Difficulty or Workload**	12 (18%)	“He changed the weighting of the final from 40% to 50%. . .because he wanted to ensure that people were being honest because there was a proctored final.” “Excessive assignments such as discussion boards for each daily meeting of our class.”
Instructor assigning additional material, assignments, or stricter grading to adjust to online format and/or to prevent cheating.		
		“He used free response on exams (something he previously did not do) in order to combat cheating. He did not alter the time allotted to take the test.”
**Pre-recorded lectures**	10 (15%)	“I was more likely to attend a live session than I was to just go and look at already pre-recorded material.”
Use of lectures recorded in advance and posted for students to view.		“There wasn’t anything that was live it was just pre-recorded.”
**Inadequate communication strategies** Instructor not responsive to email or infrequent interactions with students	10 (15%)	“I found the most ineffective form of teaching was when they would just basically just give us slides, and just say good luck, you’re on your own.”
		“When you send an email they respond like really vague so that that wasn’t great.”
**Technology Failures** Technologies that frequently failed, were inefficient, or required bandwidth/storage that not all students had access to	9 (13%)	“My calc instructor decided to randomly switch the format for our final to this website…that kicked people out or made you resubmit your answers.”
		“There was no backtracking [in online tests], so if you didn’t know the answer, you just have to guess. You couldn’t just skip it and come back to it later.”
**Long-form or outdated lecture formats** Instructor use of lengthy lectures, outdated recordings, and/or poor quality video or audio.	8 (12%)	“So because lectures were online they weren’t restricted to 50 minutes…so they would just make the lectures an hour and a half to fit all.”
		“He was just posting notes and then reciting the notes in an mp3 recording.”
		“I had one instructor that was using [video] lectures from 2014. So they were outdated. Like if, this lecture video said chapter 13, it was like chapter 25 in my current book.”
**Required attendance at live lectures** Instructors making attendance at live lectures part of the course grade.	4 (6%)	“This was a demotivator because when it is when you are attending a lecture in person it’s much different from attending it via Zoom. So requiring Zoom attendance just I don’t know why but it just doesn’t work.”
		“I think a lot of students had trouble attending [live lectures]…since you’re home…you can’t block out the same amount of time.”
**Other** Did not fit other categories	1 (%)	“Um I personally hated group projects.“
**Nothing** Had no examples of ineffective strategies, tools, or techniques	14 (21%)	“Yeah, nothing comes to mind at the moment.”

### Instructor caring behavior

The 59 participants made a total of 87 responses to the question: “What are some things your instructors did during remote instruction that made you feel like they cared about their students?” Two major themes and 4 minor themes emerged (Table 5). The most common response, accounting for 29% (n = 25) of responses related to instructor leniency and/or flexibility with respect to course policies or assessments. Examples included use of pass/fail or other flexible grading policies, allowing homework to be turned in past the deadline, and allotting more time for assignments. The next most common theme (25%; n = 22) was instructor responsiveness and accessibility to students. Examples included prompt replies to student emails, flexible office hours, and frequent engagement on discussion boards. About 13% (n = 11) of responses related to instructors bonding with the class, such as through words of encouragement or just spending time acknowledging the pandemic and asking how students were coping. Similar to this theme which refers to instructors’ interactions with the entire class, another 13% (n = 11) of responses related to the instructor offering one-on-one opportunities to check in with students and provide emotional support. The next theme, comprising 9% (n = 8) of responses related to any sign the instructor put in effort to ensure the class was a success, including learning and using new technologies, expressing enthusiasm, or generally seeming to put in significant effort to maintain high-quality instruction. About 7% (n = 6) of responses related to instructors seeking student feedback on how the course was going. The remaining 5% (n = 4) of responses suggested the student could not think of any examples of caring instructor behaviors.

**Table 5:** Actions that made students feel their instructor cares (N = 59; N = 87 responses).

Theme	N (%)	Illustrative examples
**Leniency:** relaxing policies (e.g., grading) or deadlines, and accommodating unforeseen challenges like technology issues.	25 (29%)	“Reduced the number of tests.”
		“Showed extra leniency for technological issues or late assignments.”
**Responsive and available:** instructors being available (e.g., discussion forums, extra office hours) and responsive to email and other inquiries	22 (25%)	“They had more office hours, and they just responded faster to emails.”
		“Some instructors were active all the day on the forum.”
**Bonding:** attempts to bond through casual chatting about pandemic news, offering encouragement or conveying empathy for challenges faced by students	11 (13%)	“We’d just talk about how stuff is going and if everyone’s staying healthy and stuff.”
		“They routinely offer encouraging words to us.”
**Individual support:** openness to or encouragement of one-on-one discussions of experiences or challenges	11 (13%)	“Offering one on one help both academically and emotionally.”
		“She used to like, personally ask everyone, "how are you doing?"
**Put in effort:** made ample efforts to insure good instruction was happening, such as learning technology or providing additional study materials, and/or maintain a positive environment	8 (9%)	“Instructors that tried new technologies, even though they did not know how it worked, instructors who the made the effort to, you know, go the extra mile.”
		“I had the one very enthusiastic instructor.”
**Sought student feedback:** instructors asked class was going, how to improve, and valued their input	6 (7%)	“A lot of my classes actually didn’t proctor exams or anything like that, especially after students talked about privacy concerns.”
		“He was trying to adjust to how we would take quizzes in the class and he sort of like tried a couple different formats and he was like, oh, do you like this? If it doesn’t work like you can do corrections on it. We can try a different format.”
**Nothing:** did not answer or gave no examples	4 (5%)	“Um, not much really.”

### Instructor uncaring behavior

The 59 participants made a total of 72 responses to the question: What are some things your instructors did during remote instruction that made you feel like they did not care about their students? Over one-quarter of responses (25%; n = 18) had no examples of uncaring behavior by instructors to share. Otherwise, two major themes and three minor themes emerged (Table 6). The most common response (28%; n = 20) referred to poor communication, including unanswered emails, lack of empathy conveyed in communications, and scolding the class for underperformance. The next most common response (15%; n = 11) referred to instructors increasing the difficulty of the course, including making exams harder to offset the assumed impact of cheating, assigning more work, or grading harder. Minor themes included instructors being unprepared or disorganized (13%; n = 9), including expending minimal effort into the online format, posting assignments at the last minute, and frequently changing requirements, rules, and standards. Another minor theme (11%; n = 8) was inflexibility which included the instructor making no accommodations for students including international students, students with unreliable technology/internet access, students in different time zones, or students with special educational needs. The final minor theme was insufficient instruction or guidance (8%, n = 6). Examples included instructors posting assignments and lectures and leaving students to work on their own with no guidance, minimal or no opportunities to engage with the instructor, and minimal instruction provided for assignments.

**Table 6:** Actions that made students feel their instructor doesn’t care (N = 59 participants, N = 72 responses).

Theme	N (%)	Illustrative examples
**Poor Communication:** unresponsive to emails, communication lacked empathy or was discouraging, or did not answer students questions adequately.	20 (28%)	“We all emailed him, no response, and then we had to email the TA. And the TA also was like, he went MIA on he’s not responding to my emails.”
		“He is so pessimistic about the future, I mean, whether it’s true or not, it just didn’t really need to be said.”
**Increased difficulty:** Exams, assignments, course activities and/or grading became more difficult, stringent, or more laborious.	11 (15%)	“Just blatantly making [an exam] harder because he doesn’t want students to cheat.”
		“Lengthening lectures. That hurt. let’s see, also just kind of like raising the standards.”
**Unprepared:** instructors were disorganized, changed rules or standards, put little effort into moving course online, or posted last minute announcements	9 (13%)	“My Calculus class didn’t let us know what the format of final would be until the last minute, which lead to the spread of misinformation.”
		“Not updating students about the course syllabus changes in a timely manner.”
		“The ones that didn’t put as much effort just like seemed like they didn’t really care about it as much.”

**Inflexible:** did not accommodate special circumstances or needs, including time zones, or generally was inflexible with rules	8 (11%)	“I had a…major timezone difference. And so…refusing to post lectures online and requiring live attendance…caused problems for me and I just felt like they weren’t going out of their way.”
		“He would take off 10 points per 30 seconds late.”
**Insufficient instruction or guidance:** not providing clear instructions, holding consistent class meetings, or leaving students unsure about requirements and standards	6 (8%)	“When you’re not doing the lab [in person] and you’re just kind of like getting a dataset and a couple videos, you have no idea what’s actually going on.”
		“But if [you] had to actually ask about everything and you know, everything was unclear. So, to me, they’ll like Oh, they don’t really care.”
**Nothing**: did not answer or mention a concrete example	18 (25%)	“I did not have an experience like this. All of my instructors were great.”

### What universities did well

The 59 participants made a total of 116 responses to the question: What are some things that your university did to help students be successful during remote instruction? Two major themes and 6 minor themes emerged (Table 7). The most common response (41%; n = 48) was classified as administrative flexibility, examples of which included university-wide pass/fail policies, waiving limits on counseling sessions, and extending administrative deadlines. The next most common response (24%; n = 28) was provision of remote services such as tutoring, counseling, and advising. One minor theme was agile response (9%; n = 10) which referred to how quickly and smoothly university services changed to meet student needs. Other minor themes included seeking student input during the process via town halls, surveys, and direct email solicitations (6%; n = 7); provision of technology such as wifi hotspots or laptops (4%; n = 5); effective communication strategies (4%; n = 5); and financial assistance in the form of fee waivers and refunds (3%; n = 4). A small percentage of responses (8%; n = 9) suggested the student could not think of any examples of what the university did well.

**Table 7:** University actions which helped students be successful in Spring 2020 (N = 59 participants, N = 116 responses).

Theme	N (%)	Illustrative examples*
**Flexibility:** students felt administrative flexibility such as pass/fail options for final grades, waiving limits on counseling sessions, or extending administrative deadlines was helpful	48 (41%)	“We went the pass/fail option was the big thing because that that is a lifesaver and a grade saver.”
		“If you went to counseling on campus I think there was a limit to how many you could go to, but they lifted the limit.”
**Remote services**: students felt remote services such as advising, tutoring or mental health counseling were helpful	28 (24%)	“Tutoring it was also being done through zoom.”
		“They also did provide remote counseling services which was really helpful.”
**Agile response:** students felt their university was prepared and quickly took steps to minimize disruptions	10 (9%)	“I would say they were very quick in moving everything online.”
		“They kept the library open if you didn’t have internet in your place.”
**Sought student feedback**: students appreciated university’s efforts to get their input through townhalls, surveys, or direct email solicitation	7 (6%)	“My university held townhalls on COVID.”
		“They were very attentive to the students voices.”
**Technology**: students felt assistance with technology issues such as internet access, laptops, webcams or online resources were helpful	5 (4%)	“They made sure that every student has some form of internet access.”
		“They were providing loans, laptops to borrow, to the students.”
**Good communication**: students felt university’s efforts to keep them informed were helpful	5 (4%)	“They explained their plan as it was going along so it kind of gave some closure as the decisions that they were making and what steps they were gonna take for next semester.”
**Financial assistance**: students felt fee waivers, refunds and other financial support was helpful	4 (3%)	“They also had some type of like funding, where if students had financial need they could fill out a form and get up to like $1,000.”
		“We also had our Distance Education fees for the summer waived.”
**Nothing:** students did not mention any helpful actions	9 (8%)	“I think the university did a bad job.”

### What universities could have done better

The 59 participants made a total of 69 responses to the question: What do you wish your university did to better help students be successful during remote instruction? Three major themes and six minor themes emerged (Table 8). The most common response (26%; n = 18) was that students wanted the university to create policies for faculty and departments to increase the consistency in how courses were carried out in terms of grading and teaching modalities. The second most common response (23%; n = 16) was a university communication strategy that was honest, prompt, clear, and transparent. The next most common response (10%; n = 7) was to improve the responsivity and support provided by university offices and services (e.g., financial aid, counseling, tutoring). Minor themes included engaging student input to crisis response (6%; n = 4), provision of technology (4%; n = 3), provide specific accommodations for international students (4%; n = 3), refund fees for services not rendered (3%; n = 2), provide opportunities for students to interact with one another (3%; n = 2), and provide more effective policies to prevent cheating (3%; n = 2). The remaining responses (17%; n = 12) suggested the student could not think of examples of things the university could have done better.

**Table 8:** Things students wish their university had done better (N = 59 participants, N = 69 responses).

Theme	N (%)	Illustrative examples
**Policies for Faculty and Departments:** student felt faculty and departments should receive clear and unified policies, not just guidelines or suggestions, to ensure uniformity and fairness.	18 (26)	“I would have preferred if their guidelines were actual policies, as many instructors chose to ignore them.”
		“Put down harsher restrictions on instructors to make sure they do not harm students.”
**Communication**: student wanted university communication to be more honest, humble, clear, consistent, transparent, or less abrupt	16 (23)	“I just felt like the way that they were reaching out wasn’t very effective…it just seemed very superficial.”
		“Communicate that they don’t know what they’re doing.”
**Nothing**: student was generally satisfied with their university’s actions	12 (17)	“Yeah, nothing nothing really comes to mind. I think they did the best they could and I wouldn’t have any suggestions.”
**Offices and Resources:** student felt university offices could have been more responsive and support for resources increased	7 (10)	“I think the central offices of the universities could have been more organized like financial aid…they were really unavailable to everyone and took like months to reply.”
		“I know one girl mentioned her school provided counseling resources and I wish my school did that.” “When it comes to like tutoring, like it was kind of hard for me to like find people that will actually help me.”
**Student Input:** student felt university should collect and incorporate student input into their actions and policies	4 (6)	“In general, just taking in student input and gauging how students feel about this, or this decision or that decision uh would have helped out a lot.”
		“I wish my university let us give feedback on the instructors and their teaching methods so that changes to cater to each student could be made through the semester.”
**Technology:** student felt technology for remote instruction or training for faculty in said technology could have been better	3 (4)	“I wish they had better software systems in place to help with remote instruction because the ones they used weren’t effective.”
		“They should teach some of the instructors to be more tech savvy.”
**International Students:** student felt issues impacting international students could have been handled better	3 (4)	“I think with a university such uh with a huge number of international students should have been more considerate about the international student.”
		“I guess as an international student there was a lot of confusion about visa. So I wish [my university] was more transparent with what they were doing regarding it.”
**Fees:** student felt fees should be reduced or eliminated to reflect services provided	2 (3)	“I wish my university refunded us for on-campus services that we couldn’t use such as our gym and health center fees.”
**Student Interactions:** student felt opportunities to engage with each other were lacking	2 (3)	“I’m not sure what this would have looked like, but I feel like it would have been nice to have another more ways to connect with other students in the course since we didn’t have that like face to face.”
**Cheating:** student felt policies to prevent cheating could have been improved	2 (3)	“I wish my university had a lot stricter guidelines to reduce cheating during tests…I feel like a lot of my like anxiety…was literally just from worrying about the curve being destroyed by cheaters.”

## Discussion

The results of the present study revealed that generally students expressed that hybrid instruction and the use of multiple complementary resources and technologies were preferred, while reliance on pre-recorded lectures, poorly functioning technology, and insufficient opportunities to communicate with the instructor interfered with their ability to learn course material. The preference for live over pre-recorded lectures is consistent with a recent study that showed that 80% of undergraduate students said video chat and live streaming have made remote instruction better and 72% said the ability to connect with instructor and other students over live video was important [15]. Interestingly, some students valued recorded lectures due to the flexibility they provide, and this may be particularly important for them to juggle school, work and home life. However, when recordings were in lieu of live class time, lengthy, low quality, or clearly recycled from a previous semester, they were often found to be insufficient. Even though the convenience of recorded lectures was mentioned, many students voiced that it was harder to get motivated and pay attention when classes were reduced to viewing recordings. More work is needed to examine the impact of live versus recorded lectures during the pandemic on attendance/views and student engagement as well as academic performance and intentions to stay in a STEM major [16, 17].

Results also revealed university actions that students perceived as beneficial including lenient grading policies (e.g., pass/fail), a quick and smooth response to the emergency, and remotely offering campus services. Many students reported that they experienced too much variability in policies and practices between faculty and departments which for them signaled a need for the university to step in and create campus-wide policies. They felt this would not only help them stay focused and organized but also prevent particularly egregious instructor practices such as instructors being largely inaccessible to students, conducting the entire course by simply posting lecture notes, or increasing the difficulty level and/or workload of courses. Research on student performance across multiple courses under conditions of varying modalities across courses may be needed. Another area for improvement was related to the response of university services (e.g., financial aid) during campus closures which was often cited as slow and inefficient. Research suggests that for remote instruction to be effective it must be accompanied by an educational ecosystem to support the remote learner [5]. Unfortunately, the abrupt move of campus employees to work-from-home appeared to at least temporarily affect their ability to meet students’ needs during the pandemic. Campus employees likely weren’t used to working from home but many were also likely to be juggling children who were schooling from home while attempting to do their jobs [18]. Provision of safe and affordable childcare options for campus employees have been called for in general [19] and the pandemic has revealed this need further. Universities may also need to develop protocols for an online ecosystem that provides students efficient access to services and resources when the campus is closed in an emergency. Further, some services that went online, such as telehealth student counseling, should be continued post-pandemic to increase reach (e.g., to commuter and nontraditional students) and reduce disruptions in care (e.g., winter break).

A cross-cutting theme, emerging in responses to every question we asked about both instructors and universities, was communication. Students desired communication from both instructors and universities that was responsive, transparent, timely, and empathetic. Consistent with prior research [20], when these qualities were conveyed by instructors and/or universities, students felt more aware of what was expected of them and more valued and respected. In Fall 2020, some universities were called out for harsh messaging to students that shames and blames them for COVID19 spread on campuses [21]. Our findings suggest such an approach to communication is not likely to be well-received by students. Interestingly, a survey of undergraduate students in Fall 2020 found that 37% of students said their opinion of their university declined during the semester [15], suggesting that some students have not been satisfied with how their universities have handled the pandemic.

Research has demonstrated that student-instructor and student-student interactions can promote student achievement [22], perceived learning, and student satisfaction during remote instruction [23]. Our findings on communication suggest that instructors need to create more opportunities to engage with students during remote instruction and to frequently evaluate whether their engagement plan meets students’ needs. Effective engagement strategies were mentioned by students, many of which are supported by prior research [24], included the use of formal communication platforms, quick email response times, remote office hours, offers to meet one-on-one, and live lectures that allow for students to ask questions and hear other students ask and get answers to their questions. When live lectures are not offered, the onus is on instructors to provide alternative ways to engage with students while being mindful that voluntary forms such as virtual office hours may not be sufficient to accommodate large class sizes and may be underutilized by students who are bashful, have erratic schedules, or do not wish their home environment to be seen on video.

Some students mentioned appreciating the use of novel communication platforms (e.g., Piazza) that allowed them to ask questions any time (without having to send emails), post questions anonymously, get incentivized for answering each other’s questions, organize conversations into searchable threads, and access everything via a mobile app. The reliance on email to communicate with students is proving to be insufficient as evidenced by a study that found that students who were provided novel tools to communicate with instructors and other students this fall rated their motivation and engagement with learning outside of class significantly higher [15]. The importance of student engagement is underscored by emerging research that shows undergraduate students continue to feel inadequately engaged by their instructors. A recent survey of 3,412 undergraduate students in the middle of the Fall 2020 semester found that only 40% agreed that their remote instruction experience is engaging during class time and only 32% agreed they are being adequately engaged outside class time [15]. That same study showed that 85% of students felt that instructors should foster a sense of community among the students in online courses. This signals the need for better implementation and dissemination of best practices for engaging students during online instruction [25]. Research is needed on how novel technology-based communication platforms influence both instructor-student engagement and student-student engagement and ultimately, student motivation and academic performance. Effective remote communication strategies should also be continued post-pandemic to provide more flexibility in options for students.

Two themes emerged across questions that we suspect are related. Students emphasized the need for leniency in grading, assignments, and expectations, while also expressing concern regarding increased difficulty level and/or workload of courses. Although the stress and disruption related to the pandemic likely contributed to students’ pleas for leniency, instructors should consider that pleas for leniency may also be a result of increases in difficulty level and course workload that may be occurring intentionally or unintentionally. Consistent with our findings, a survey of 148 undergraduates found nearly one-third reported one or more of their instructors had increased the workload in Spring 2020 [26]. Some practices may have created more work for students than instructors realize. For example, students reported that to avoid online cheating, some instructors replaced exams with class projects that ended up requiring more time to accomplish than the time they would have spent studying for an exam. They also reported that instructors increased the difficulty level of courses as a way to offset the impact of online cheating, by replacing multiple choice exams with free response without allocating sufficient time to complete the exam or by simply increasing the stringency of the grading curve. In addition to increased difficulty, students are reporting numerous other challenges associated with anti-cheating software, including having to read and follow elaborate instructions on how to take the test, anxiety and discomfort associated with being watched through your computer during an exam, and being accused of cheating when internet connection issues disrupt the exam [27]. Another example students gave regarding increased workload was instructors posting lecture videos that exceeded the usual lecture time. This may have occurred because nothing stops an instructor from running over time when recording a video as opposed to in-person lectures which have a hard stop time. Even when video lectures matched the length of in-person lectures, students said these took longer to digest because watching videos was more cognitively taxing compared to live lectures where interaction occurs. Class interaction breaks up the monotony of a lecture and also allows students to ask the instructor to clarify concepts and answer questions in the moment and hear answers to other student’s questions, all of which can facilitate learning [28, 29]. Some students said they needed to take frequent breaks when watching lecture videos, rewind and re-listen to parts they didn’t absorb, and work much harder to stay focused, all of which made the time spent on the lecture go beyond the time they would spend in an in-person lecture. Interestingly, their experience was that the extra time they spent on lectures did not result in better learning compared to in-person classes, but instead, worse learning. Breaking up video lectures into short units, recording live lectures so that class interaction is captured in the video, and allowing students to interact while they are watching may be ways to enhance the experience of watching class by video. Another way students said workload was negatively impacted was when instructors posted their lecture videos at the end of the week or at erratic times rather than at class time, which made it difficult for students to manage their time. Interestingly, the problems students cited were often things that could be remedied by changing practices and/or leveraging available technologies. Inadequate instructor practices may be driving the common sentiment that remote learning is generally worse than in-person. Indeed, a recent survey of undergraduate students found that 68% said remote instruction is less effective than in-person instruction [15]. This sentiment runs counter to research on remote instruction outside of the context of emergencies which shows positive outcomes on student performance [30–33] and no differences in student satisfaction relative to in person instruction [34]; however, as discussed elsewhere, it is doubtful that best practices for remote instruction were being implemented widely in Spring and Fall 2020 [5].

The present study has some limitations. Though participants were diverse in terms of gender, SES, and race/ethnicity, our sample size was too small to compare results by those demographic factors and did not represent the entire range of gender and racial/ethnic diversity in the US. Further, students were asked about their experiences in Spring 2020 which may not generalize to Fall 2020 when instructors had more time to prepare. This work was an initial step conducted to inform survey questions for a larger survey study that assessed students’ experiences in Fall 2020 and disproportionate impacts of the pandemic on STEM education by race/ethnicity, gender, and SES.

Our findings revealed that many students felt universities and instructors lacked a cohesive strategy for emergency remote instruction. To be sure, few anticipated the circumstances of 2020 and students, instructors, and administrators were all under enormous stress. However, a robust literature on online instruction exists [35, 36] and evidence points to the increasing possibility that pandemics and other natural disasters are likely to occur in the future [37, 38]. Done well, remote instruction can actually increase STEM participation and diversity, which signals the urgent need for broad adoption of best practices [39]. Going forward, universities must require that all instructors are proficient in remote instruction. This entails provision of training in remote instruction that is consistent with best practices and the adoption of policies that incentivize faculty to gain proficiency in these skills (e.g., embed in promotion and tenure criteria, teaching awards). This would not only prepare faculty for emergency situations like the pandemic, but even more importantly, this would position universities to develop remote learning programs that are designed to increase diversity in STEM. Remote learning programs have been developed precisely for this purpose at some land grant universities, for the purpose of bringing the STEM curriculum to diverse students rather than the usual approach which involves attempting to recruit diverse students to the often rurally-located land grant universities [39]. Studies have shown this model to be successful in increasing racial/ethnic diversity [39] and gender diversity [40]. Related, remote student recruitment strategies (e.g., virtual tours) used during the pandemic to showcase the university’s offerings to prospective students and their families should continue post-pandemic to attract students who may not be able to afford to travel for campus tours. The pandemic, by hastening universities’ and instructors’ capacities to deliver remote education and services, provides a unique opportunity for universities to build upon, allowing them to reimagine their approaches to increasing the diversity of their student body.

Universities should also develop protocols for campus closure and remote instruction that incorporate 1) data on what worked well and what did not in Spring and Fall 2020, 2) the vast body of research on best practices in remote instruction [36], and 3) iterative input from their student bodies being sure to include diverse voices. University-level policies should address course modalities, grading policies, student engagement strategies, and faculty training requirements. For example, universities should consider prohibiting course modalities that are proving to be unacceptable and/or ineffective, mandating instructor training in remote instruction tools and effective student engagement strategies, and examining the relationship between course modalities and student course evaluations to identify modalities that aren’t working well or are being executed poorly.

Similarly, instructors should produce remote instruction protocols for their courses that are informed by best practices identified in the remote instruction literature. Effective remote instruction requires a design process that takes into account myriad factors including instructor-student ratio, modality, synchrony, pedagogical style (e.g., exploratory, collaborative) among others [5]. To identify training gaps on the part of instructors, research is needed to examine how instructors approached remote instruction in Spring and Fall 2020 and to what extent it reflected best practices. Finally, effective strategies should be shared in venues that reach the academic community given the lack of implementation and dissemination of evidence-based practices for remote instruction thus far. A great example of such a venue is the Facebook group Pandemic Pedagogy which currently has 32.7K members and emerged shortly after the pandemic commenced as a forum for faculty to share their experiences with remote instruction.

A plan is now needed to address the potential negative consequences to STEM education caused by the pandemic. Universities urgently need to devise strategies to 1) identify and assist students who have exhibited declines in academic performance, 2) follow-up with students who have switched out of a STEM major since Spring 2020, and 3) re-engage students who have unenrolled temporarily or permanently as a result of the pandemic [41]. A generation of college students is at risk for long-term impacts of the pandemic on their educational and economic potential. Given the disproportionate impact of COVID19 on the very racial/ethnic groups that are underrepresented in the STEM fields [42–44], the pandemic may cause another gaping leak in the STEM pipeline. Finally, the pandemic has presented STEM education with an enormous opportunity to innovate by leveraging the new skillset instructors and university services have developed in the past year. Every crisis brings opportunities for growth. We now must accelerate the implementation and dissemination of best practices for online STEM education with the goal of increasing diversity in STEM while also identifying and ameliorating the negative impacts of the pandemic on STEM undergraduate students.

## Supporting information

S1 Table Representation of subgroups of STEM undergraduate students within each theme for each item. (DOCX)

S1 File Focus group script. (DOCX)

S2 File Focus group transcripts. (DOCX)
