# Supplementary material for: STEM undergraduates’ perspectives of instructor and university responses to the COVID-19 pandemic in Spring 2020
Source: PLoS One. 2021 Aug 27;16(8):e0256213. doi: 10.1371/journal.pone.0256213 (PMC8396789; doi:10.1371/journal.pone.0256213)
Supplement: S1 Table — (DOCX) [file pone.0256213.s001.docx]

S1 Table: Representation of subgroups of STEM undergraduate students within each theme for each item

|  | **High SES Asian Women** | **High SES Asian Men** | **High SES Black Women** | **High SES Black Men** | **High SES White Women** | **High SES White Men** | **High SES Hispanic Women** | **High SES Hispanic Men** | **Low SES Asian Women** | **Low SES Asian Men** | **Low SES Black Women** | **Low SES Black Men** | **Low SES White Women** | **Low SES White Men** | **Low SES Hispanic Women** | **Low SES Hispanic Men** |
| --- | --- | --- | --- | --- | --- | --- | --- | --- | --- | --- | --- | --- | --- | --- | --- | --- |
| **Effective Instructor Strategies** | | | | | | | | | | | | | | | | |
| Hybrid instruction |  |  |  |  |  |  |  |  |  |  |  |  |  |  |  |  |
| Multiple resources |  |  |  |  |  |  |  |  |  |  |  |  |  |  |  |  |
| Communication |  |  |  |  |  |  |  |  |  |  |  |  |  |  |  |  |
| Pre-recorded lectures |  |  |  |  |  |  |  |  |  |  |  |  |  |  |  |  |
| Leniency |  |  |  |  |  |  |  |  |  |  |  |  |  |  |  |  |
| Keeping the same schedule |  |  |  |  |  |  |  |  |  |  |  |  |  |  |  |  |
| Nothing |  |  |  |  |  |  |  |  |  |  |  |  |  |  |  |  |
| **Ineffective Instructor Strategies** | | | | | | | | | | | | | | | | |
| Increasing difficulty or workload |  |  |  |  |  |  |  |  |  |  |  |  |  |  |  |  |
| Pre-recorded lectures |  |  |  |  |  |  |  |  |  |  |  |  |  |  |  |  |
| Inadequate communication strategies |  |  |  |  |  |  |  |  |  |  |  |  |  |  |  |  |
| Technology failures |  |  |  |  |  |  |  |  |  |  |  |  |  |  |  |  |
| Long form or outdated lecture formats |  |  |  |  |  |  |  |  |  |  |  |  |  |  |  |  |
| Required attendance at live lectures |  |  |  |  |  |  |  |  |  |  |  |  |  |  |  |  |
| Other |  |  |  |  |  |  |  |  |  |  |  |  |  |  |  |  |
| Nothing |  |  |  |  |  |  |  |  |  |  |  |  |  |  |  |  |
| **Instructor Caring Behavior** | | | | | | | | | | | | | | | | |
| Leniency |  |  |  |  |  |  |  |  |  |  |  |  |  |  |  |  |
| Responsive and available |  |  |  |  |  |  |  |  |  |  |  |  |  |  |  |  |
| Bonding |  |  |  |  |  |  |  |  |  |  |  |  |  |  |  |  |
| Individual support |  |  |  |  |  |  |  |  |  |  |  |  |  |  |  |  |
| Put in effort |  |  |  |  |  |  |  |  |  |  |  |  |  |  |  |  |
| Sought student feedback |  |  |  |  |  |  |  |  |  |  |  |  |  |  |  |  |
| Nothing |  |  |  |  |  |  |  |  |  |  |  |  |  |  |  |  |
| **Instructor Uncaring Behavior** | | | | | | | | | | | | | | | | |
| Poor communication |  |  |  |  |  |  |  |  |  |  |  |  |  |  |  |  |
| Increased difficulty |  |  |  |  |  |  |  |  |  |  |  |  |  |  |  |  |
| Unprepared |  |  |  |  |  |  |  |  |  |  |  |  |  |  |  |  |
| Inflexible |  |  |  |  |  |  |  |  |  |  |  |  |  |  |  |  |
| Insufficient instruction or guidance |  |  |  |  |  |  |  |  |  |  |  |  |  |  |  |  |
| Nothing |  |  |  |  |  |  |  |  |  |  |  |  |  |  |  |  |
| **What Universities Did Well** | | | | | | | | | | | | | | | | |
| Flexibility |  |  |  |  |  |  |  |  |  |  |  |  |  |  |  |  |
| Remote services |  |  |  |  |  |  |  |  |  |  |  |  |  |  |  |  |
| Agile response |  |  |  |  |  |  |  |  |  |  |  |  |  |  |  |  |
| Sought student feedback |  |  |  |  |  |  |  |  |  |  |  |  |  |  |  |  |
| Technology |  |  |  |  |  |  |  |  |  |  |  |  |  |  |  |  |
| Good communication |  |  |  |  |  |  |  |  |  |  |  |  |  |  |  |  |
| Financial assistance |  |  |  |  |  |  |  |  |  |  |  |  |  |  |  |  |
| Nothing |  |  |  |  |  |  |  |  |  |  |  |  |  |  |  |  |
| **What Universities Could Have Done Better** | | | | | | | | | | | | | | | | |
| Policies for faculty/departments |  |  |  |  |  |  |  |  |  |  |  |  |  |  |  |  |
| Communication |  |  |  |  |  |  |  |  |  |  |  |  |  |  |  |  |
| Nothing |  |  |  |  |  |  |  |  |  |  |  |  |  |  |  |  |
| Offices and resources |  |  |  |  |  |  |  |  |  |  |  |  |  |  |  |  |
| Student input |  |  |  |  |  |  |  |  |  |  |  |  |  |  |  |  |
| Technology |  |  |  |  |  |  |  |  |  |  |  |  |  |  |  |  |
| International students |  |  |  |  |  |  |  |  |  |  |  |  |  |  |  |  |
| Fees |  |  |  |  |  |  |  |  |  |  |  |  |  |  |  |  |
| Student interactions |  |  |  |  |  |  |  |  |  |  |  |  |  |  |  |  |
| Cheating |  |  |  |  |  |  |  |  |  |  |  |  |  |  |  |  |
