# Supplementary material for: STEM undergraduates’ perspectives of instructor and university responses to the COVID-19 pandemic in Spring 2020
Source: PLoS One. 2021 Aug 27;16(8):e0256213. doi: 10.1371/journal.pone.0256213 (PMC8396789; doi:10.1371/journal.pone.0256213)
Supplement: S1 File — (DOCX) [file pone.0256213.s002.docx]

S1 File. Focus group script

Welcome

Welcome to the focus group! I am ______ a research coordinator from Penn State University.

Rules Discussion

We will be asking a number of open-ended questions. There are no wrong answers but people may have differing points of view. Please feel free to share your point of view even if it differs from what others have said. Keep in mind that we're just as interested in negative comments as positive comments. We want everyone to feel comfortable participating and encourage everyone to talk. Because we have to keep this to an hour I may need to cut you off, so I will apologize in advance. We want to get the full breadth of experiences and opinions but some of you may have very similar experiences and opinions. If your answer is the same as another person you may simply say “My answer is the same as Sam’s.”

It is difficult for us to take notes on everything that is said in the group. So, we will also be recording the session so that we may transcribe it later. We will not associate your name with anything you say in the focus group.

You may refuse to answer any question or withdraw from the group at any time.

We understand how important it is that this information is kept private and confidential. We will ask participants to respect each other’s confidentiality and not repeat things said within the group to anyone outside of the group.

Are there any questions before we get started? Please say your name at the beginning of each of your responses so we know who is speaking. Let’s get started!

Overview

The COVID-19 pandemic has presented big challenges for undergraduate students. As you know, universities across the nation have closed, moving all instruction online with very little time for students or faculty to prepare. Many students have encountered challenges related to the closing of campuses, relocation, and the movement to online instruction. In this study we want to learn how the closing of campus and the pandemic in general has affected you; and we want your opinions about the good and bad on how things went as well as what your professors and universities did that was helpful and unhelpful, and what you wish they would have done.

**Let’s start by discussing where you went when your campus shut down. I’d like to hear from each of you.**

Where did you move to? And who else lives there?

Tell me about your space in that home. What space do you have to do your school work there? Comment on whether the space is private or shared and if shared, who you share it with.

What challenges have you encountered attending classes or doing your school work in this space?

Thinking about the people who also live in that home, what if anything did they do to help you to attend classes and do your school work?

What if anything did they do that made it harder for you to attend classes and do your school work?

Overall, what challenges did you encounter in terms of your ability to attend your classes and do homework at home (or in the place you were relocated to)?

What was helpful to you in terms of your ability to attend classes and do homework at home (or in the place you were relocated to)?

Tell me about any technology-related issues you encountered. For example, access to computers, wifi, knowledge of online platforms etc.

If you live with siblings, to what extent were there differences in how much parents supported each of you in your ability to attend classes and complete school work? For example, were some kids in the house given more time, space, and consideration to do school by parents than others?

**Now let’s talk about what other responsibilities you had after your campus closed and how these differed from responsibilities you had before campus closed.**

Describe your household responsibilities such as chores during this time and how they were alike or different from your household responsibilities before school closed. (Do not include child care, I will ask about that shortly.)

Describe your work responsibilities (meaning employment) during this time and how they were alike or different from your work responsibilities before school closed.

Describe your childcare responsibilities during this time and how they were alike or different from your childcare responsibilities before school closed.

Describe any other caregiving responsibilities you had during this time such as caring for elderly or ill family members, and how they were alike or different from you caregiving responsibilities before school closed.

Did you have any other responsibilities during this time at home that we didn’t discuss? What were they?

If you live with siblings, to what extent were there differences in how much your parents expected each of you to take on household, work, childcare, and/or family caregiving responsibilities?

**Now let’s talk about how your academics were impacted by the shutdown. I will ask you how the shutdown has impacted a variety of things. If you don’t think it impacted something I ask you about just say “no impact.”**

How has the shutdown impacted your class attendance?

How has the shutdown impacted your academic performance (grades)?

How has the shutdown impacted your ability to understand the subject matter of your courses? Which subjects were impacted?

How has the shutdown impacted your career goals?

How has the shutdown impacted the quality of the education you are receiving?

How has the shutdown impacted the availability of other learning opportunities such as internships?

How has the shutdown impacted your confidence in your ability to successfully complete your bachelor’s degree?

How did the campus shut down impact your employment and/or finances?

**Now let’s talk about what your professors did and did not do during the move to online learning.**

How did your professors conduct their online courses?

What are some examples of strategies, tools, or technologies that your professors used that you found to be very effective (in that they made it easier to learn) during online learning?

What are some examples of strategies, tools, or technologies that your professors used that you found to be ineffective (in that they did not help you learn) during online learning?

What are some things your professors did during online learning that made you feel like they cared about you or other students in the class?

What are some things your professors did during online learning that made you feel like they did not care about you or other students in the class?

How did grading work in your classes? For example, some courses turned into pass/fail and sometimes students were given a choice between a grade and pass/fail.

**Now let’s talk about how your university responded to the move to online learning.**

What are some things that your university did to help students be successful during online learning? Mention any resources they provided or actions they took to help students be successful or feel supported.

What do you wish your university did to better help students be successful during online learning?

In general, are there any ways that your ability to be successful in school was affected by the pandemic that we have not discussed?

How do you feel about going back to campus in the fall?

What concerns do you have about going back to campus in the fall?

What concerns do you have about staying home and learning online in the fall?

**That is the end of my questions. Thank you for participating in this focus group.**
