# Supplementary material for: STEM undergraduates’ perspectives of instructor and university responses to the COVID-19 pandemic in Spring 2020
Source: PLoS One. 2021 Aug 27;16(8):e0256213. doi: 10.1371/journal.pone.0256213 (PMC8396789; doi:10.1371/journal.pone.0256213)
Supplement: S2 File — (DOCX) [file pone.0256213.s003.docx]

**S2 File:** Focus Group Transcripts

**Low- SES Black Women Transcript**

Moderator 1:30

Hi everyone, it is three o'clock but we are waiting on a couple people so I'm gonna wait a minute or two just to make sure they're not on their way or anything like that but we'll get started in a second. Okay, um we're just gonna go ahead and Get Started. So welcome to the focus group. My name is Moderator. I'm a research coordinator from Redacted University. Um Moderator is going to be our note taker today. Moderator, do you want to say hi.

Hi, everyone. Thanks for coming. excited to get started.

Umm we'll be asking a number of open ended questions. There are no wrong answers, but people may have differing points of view, please feel free to share your point of view, even if it differs from what others have said. Keep in mind that we're just as interested in negative comments as positive comments. We want everyone to feel comfortable participating and encourage everyone to talk. Because we have to keep this one hour I might need to cut you off. So I will apologize in advance for that. We want to get the full breadth of experiences and opinions. But some of you may have very similar experiences and opinions. If your answer is the same as the person's you can just say, my answer is the same as theirs. It's difficult for us to take notes and everything that said in the group, so we're going to be recording the session today. So that we can transcribe it later. But your name will never be associated with anything you say in the focus group. You may refuse to answer any question or withdraw from the group at any time. We understand how important it is that this information is kept private and confidential. So we'll ask that participants respect each other's confidentiality and not repeat things said in the group to anyone else. So first, let's go around and everyone say your name. What year of college you just finished and one word to describe what life has been like during the pandemic.

Umm 60, we can start with you.

60 5:37

Hi, um, my name is 60. You want my last name too?

Moderator 5:41

Uh, no, that's okay.

60 5:44

Uh, I'm currently a student at the Redacted College of Redacted. And what's the last thing you said? I'm Sorry.

Moderator 5:51

Um, one word to describe what life during the pandemic has been like for you.

60 5:56

uhh roller coaster

Moderator 5:59

okay. And 59.

59 6:03

Hi, I'm 59, I go to the University of Redacted. Um and I think one word that would describe my life during the pandemic would be an adjustment, so adjustment. Uh just because I think everyone is like learning how to live in this new normal. So, yeah.

Moderator 6:20

Okay, so the covid 19 pandemic has presented big challenges for undergraduate students. As you know, universities across the nation are closing and moving all instruction online. With very little time for students or faculty to prepare. Many students have encountered challenges related to the closing of campuses, relocation and the movements to online instruction. In this study, we want to learn how the closing of campus and the pandemic in general has affected you and we want your opinions about the good and bad on how things went as well as what your professors and universities did that was helpful and unhelpful and what you wish they would have done. So let's start by discussing where you went when campus shut down, tell me you about your space in the home. You move to what space do you have to do your schoolwork there and comment on whether the space was private or shared?

59 7:11

Which one of us should start?

Moderator 7:13

Uh um, whoever wants to start?

Oh 60, you can go

60 7:22

okay. Um for me, I commute to school so I it's not like I had any dorm to move out of. But there there are dorms in my college if you so choose to. Um, So I just have to stay home for this pandemic. Uh, I usually do my umm I would usually do my courses online either on the desktop in the living room of my home or if um my sister is has her laptop available. I'll use hers and I'll stay in my room um. Well, did I answer all those questions or did I miss one

Moderator 7:59

Oh, you did um. So, do you share your room with anyone? Or is it just your space?

60 8:04

Oh yeah, I share I share a room with my sister.

Moderator 8:07

Okay, sorry if you said that. Okay. And 59, what about you? What was your space like?

59 8:14

Um, so after it closed in March, we're all like expecting to go back. Um, but that obviously didn't happen. We had to move out super quickly. Um I had like, I took 30 minutes to move out of my dorm and we weren't allowed to, like move out, move out up at the same time as any of our other roommates. I think coming back home wasn't that big of adjustment for me just because that's actually what I'm used to is studying at home. Um The space I have um is obviously shared with my family, but I do have spaces to go where it's private, and I'm able to focus and study on my own. um so yeah, did that answer all the questions?

Moderator 8:54

Yeah, um so are those private spaces in your home. What exactly are those or are they somewhere else.

59 9:01

Yeah, usually I study in like my bed like even at school I studied in my bed. I didn't really go to the library. umm But we also just have like office space. Like, I could study there as well. Um so that wasn't like, it didn't affect my performance at school.

Moderator 9:18

Okay. What challenges did you both encounter attending classes or doing schoolwork in the space you're in?

60 9:27

Um for me (Echos for me). Oh, I can hear myself saying, um in your room sorry. Um, for me personally. uhh It was really different for me because I usually do my work at school. That's how I get my grades out. You know, I get all my work out. All any type of work that I have to do happens on campus. So when I go home, you know, I see myself as home being a place of relaxation, sleep, recharging yourself. So I once I started college, I did not do any form of work in my home anymore, and I just get them done in school so I may be at school for longer but once I'm home that's like when I clock out so having umm having to do the complete opposite and then change my way of learning completely disrupted me because I had nothing but my bed to sit on. I mean, I could sit on the chair but then I have to use my lap with a laptop and everything. Taking notes is a bit uncomfortable. The physical the physicality aside, my mental state is in a different place because I'm in a place of comfort where I get distracted by many things. You know, it's okay to be distracted at home but not when you're taking a test online. So my focus and concentration were like shot.

Moderator 10:47

Okay, okay. Uh That makes sense. 59, what kind of challenges did you encounter in your space?

59 10:56

Um I think for me, I usually work like in my dorm, like I said, so um it wasn't that big of adjustment with um like, my internet is honestly horrible. And even I actually moved to Redacted, like a year ago. So like the area I live in is more rural. So the internet was a huge, huge, huge problem for me, because in high school, I would just go to the library but because of the pandemic the library was closed. Um so ive been like having trouble when it was on live classes, which my calc class was live. Um That made it extremely difficult because if I watched the recordings, I can't ask questions. And if I don't attend the live, which I often wasn't able to Um that Yeah, I just disrupted the way that I learned, which is very interactive, because calc is hard. Uh So I think along those lines were it was also a problem with me, because I couldn't attend like from if they only have it from two to three And that's when my internet is low if there's nothing I can do about it, and asking math problems through, like email is extremely difficult. Also interrupted my uh learning, trying to think of what else? Also, I think, I don't know if this has to do just the move, but like my school, I think they had trouble transitioning to online classes just because they just weren't expecting it. I think up until like, the very last moment, they're like, okay, we're going to come back so it's fine. We're going to come back so it's fine. And then we didn't come back. And when we didn't come back, then they decided, Okay, now we have to change and they actually made two different curriculums, one for people who are taking the class as a grade and the other ones who are taking it pass fail because my school made it optional. The pass fail option. It was optional. Yeah, there was just the changes. I those were very hard to adjust to the changes to the curriculum, and then my Wi Fi was also a problem.

Moderator 12:57

Okay. Okay. And when you were saying that you felt like your school had a hard time switching to uh online Were there any other cases that made you that may make them more aware to you that they were having difficulty switching?

59 13:13

This is like very specific to the math department. Like I think the Math department had a hard time switching uh my bio department did it like seamlessly and she was so generous and like she asked us like she asked us How can I best like help you with this and she made sure to adjust as we were moving. Same thing with my English class, and my genetics classic, they all just switch I think, but the math department because I guess like most of the time, like we are always writing on like pencil paper type thing. So the switch is probably harder for them. But yeah, I Another thing I think, okay, if you're asking for like specific problems that they have, one would be how, because we have grad students as professors, right like they're Not, Yeah, they're not like, I don't think they're not like professors or grad students. So I guess they're like they're not like ta is either it's somewhere in between, I don't know how to explain it. But usually the grad student would come into the class, and he'll just start writing on the chalkboard. And then that's how we learn. Like, he'll just start writing problems. And then like teach us as he's going um with the transition, it actually became more structured. So um he had like, certain points he had to get to. But they also added a ton of new features. So we had to take participatory quizzes for those live classes, which I was unable to attend. Sometimes um they added oral exams rather than written exams. Um They changed, like I said, the pass fail, and then the regular classes methods, so it was much harder. You have to if you're taking the classes a grade, you have to do all of the work for pass fail, and then additional oral exams on top of that, which I think for people who like to do calculus, like it being able to write it down and Then say it is obviously so much easier. And we were asked questions that we would never have to do if it was a written exam. Um So this just made it harder

Moderator 15:09

Okay (at same time as 59 finishing up) Okay, so, um, that actually started getting into a question that I'm going to ask later, I didn't realize how much it was on your university side. So I'll come back to that. And I would love to hear more about those issues you were having there once I get around to that question. Okay.

59 15:26

Sounds good.

Moderator 15:27

All right. 60, did you want to add something?

60 15:31

Uh, yeah, I when she started talking. I started like, realizing that there was a lot of stuff I didn't mention on my school side. It really did depend on the department, um, if like, who had trouble. Also, it depended on uh the professors themselves, like if they were tech savvy or whatnot, because we would have some people who were just like, Okay, this is an easy transition, like, especially if they use a lot of technology beforehand. So the transit, transition wasn't that bad. You know, like, my calculus Professor she already taught with her umm, with the projection on the screen. And um with her...whats the thing...the her laptop. So anything she wrote down on her laptop, it was on the screen. So all she had to do was share her screen and then teach that way. You know what I mean? She wasn't really, you know, with the chalkboard method anymore. As for the math department as a whole, I think they flipped honest to God. They had a whole meltdown. You know what I mean? Because they, I'm trying my best not to exaggerate here, you know, because we have four um exams. Well, the professors are required to do at least three exams for the semester, and I had only taken one before everything uh had shut down. And for our final if you take a your math classes at a certain time, you're taking the department final, the department final is always 40% of your overall grade, that's starting off. When this thing happened, you know, we're trying to figure out how we're going to go about things. And depending on what class you were in, you could have your final be 65 to 77% of your overall grade. So for calculus students, it was 65. For what I'm hearing precalc students, it was 75. And for college algebra, it was 77%. Frankly, I'm not sure why they did this. But um, the final ended up being online, literally 13 questions meaning, each question will be 5% of your overall like towards going towards your overall grade. You know, it added a lot of stress to these students um for and for other classes. It wouldn't be that hard because uh classes that are lecture based like anything in the English department, you're just talking most of the time and you could take notes on your own accord. Um only other department that I think would have a *echo and unclear* is like classes like when I said bio and chemistry because those are lab based, you know, so we ended up doing dry labs, what they called it, um, you know, they will talk about a few things. Or we would do like an online assessment of some sort to show that we're still actively learning about something, but obviously isn't the same. So when it came to the more STEM side of things you're a bit more hands on, which is the problem with the transition like that, while As for other classes, like economics classes that I was taking, all you have to do is speak frankly, so it really wasn't um, depending on the subjects, really determined, how well the transition was for my school.

Moderator 18:34

Okay, um, thank you guys for sharing all of that. So, I will probably once again, come back around and touch on that later on. But thank you for sharing all that information. That's great. So now let's talk about what other responsibilities you had after campus closed and how those differed from responsibilities you had before beforehand. So can you describe your caregiving responsiblities like child care, elder care taking care of ill loved ones, and how are they alike or different from before the shutdown?

59 19:07

Um I i'll go first for this one just because I think mine is pretty quick, I don't really have um or I've never had those responsibilities as well. I don't really have any children in my family, my parents are able to like, go to their work, and we don't really take care of them as well. Um So in terms of that, that never like changed for me, I actually got a job after school closed because I had more time and I was able to, um and I could since a lot of jobs were moved online, I actually started teaching English to students. So like I was able to get the job because I had more time

Moderator 19:42

Okay.

60 19:44

Um for me, a few things changed but nothing crazy drastic, um, because I'm in school more often I have to be more responsible with my own diet. You know, I can't come home at like 11 o'clock and have it still haven't eaten dinner. So I'm I've already started to buy my groceries by myself. Um When pandemic happened, my dad is an essential worker. And my mom she is a stay at home mom, but she has pre existing conditions and they're both above the age of 60. At this point, well, my mom is 60. So I guess they're both in the 60 range. So um she hasn't stepped outside since March literally has not stepped outside the building since much but she would do most of the grocery shopping. So me and my sister we take turns doing grocery shopping and my sister does more of the cooking now. And we both share responsibility of cleaning everything I mean, we were already cleaning before but seeing has anything that comes into the house has to be disinfected if it hasn't been already wrapped in plastic. Obviously, if it's wrapped in plastic, it's clean, we just get rid of the plastic. Um, the problem is sometimes the time that I have to go grocery shopping during school or what has to be very quick, because me and my sister both had classes. It's not like she was done with school at the time. So if my sister had a class but there's No more groceries, I had to go out and then rush to make sure that I'm home in time before I start my class. So we have to work with each other's schedules. There was one time that I was a bit late for calculus. Um Because I had, I was waiting for the bus that doesn't come that often because at least in the city right now, um up until this uh coming August, buses are free. So that's my form of transportation because I don't have to go pay more money, because money is also an issue due to the economic crisis that will hit eventually which technically it's hitting now. So my form of getting resources have changed, but they've also been expanded. So I have to be, I had to be wary of time management. It it wasn't too hard to get used to. I think living in the city you tend to adapt really quickly and what not. So it wasn't too much of a problem, but it was more of a hassle because I am taking um hardcore classes. You know what I mean? So Just as a double that on top of making sure I come home in time to actually do my work was something that I had to take into consideration that I wasn't expecting to.

Moderator 22:10

Okay. And so you mentioned that you go grocery shopping for your family, um are your parents still able to take care of themselves and help take care of the house? Or is it just you and your sister handling that?

60 22:23

Um my parents, can take care of themselves. Uh For majority of the week, my father's at work, so my mom would prepare his meals and he would take it to work. So when he comes home, he comes home fairly late. So he's already been fed and everything. My mom she take care of herself, but sometimes um the responsibility for cooking is sort of shared with between the three of us, me, my sister, my mother. So it really depends on what we're making, you know, what, what people like what people don't like, it's not like strict like my mom can't um do this unless it has to do with her health involved. But that's been happening before the pandemic. So in terms of um uh meals and whatnot, it really hasn't changed all that much.

Moderator 23:04

Okay. And cleaning, you said you and your sister mostly handled the cleaning. Um Do your parents help with that?

60 23:13

Um to be honest no, uh my parents are are immigrants. So they have sometimes a hard time grasping what tends to be clean and what what? Well, not what tends to be clean, but what needs to be cleaned because it's sort of like if you can see it is a really dirty type thing. Uh I just tell them if they touch something that I haven't cleaned it to wash their hands, they don't like having to frequently wash their hands sort of annoying, which frankly, is annoying for everybody because then your hands turn a different color at some point, you know, I mean, from all the all the washing. It wasn't that hard to get used to because I've just been a clean freak. uh Not a clean freak, but um I tend to know what needs to be cleaned. But I never had to do that before because there was nothing to risk. You know. I mean, you do Your general amount. But since seeing that we don't know that much about this virus no one taking any, well I shouldn't say no one (laughs) but we shouldn't be taking any chances. You know? So I would clean the doorknobs when if someone left the house if you lock the door. Um basically what the uh health professionals have been telling you to be cleaning anything that you you touch often.

Moderator 24:24

Mmhmmm, Okay, okay. Uh so this next question is going to pretty much touch on some of the things you said before once again, sorry about that. um you don't have to repeat yourself if you don't have anything new to add. But if you didn't mention anything, now's the time. Describe your other household responsibilities such as chores, cooking, caring for pets during this time, and how are they alike or different from before school closed?

59 24:54

well for our house like I wasn't home before um, so it pretty much just went right back to where was when I was in high school my parents are also immigrants. So that like all falls on us, but I was also just used to doing those things already. umm I feel like yeah, my life in general just went back to from when I was before college because I was I'm a rising second year. um So it hasn't been that much of adjustment. I've never considered myself independent, even in college. So like, this is just normal for me.

Moderator 25:27

Okay. And can you tell me more about um, you said, Your parents are immigrants, so all that type of stuff like falls on to you, can you tell me more about what you mean by that.

59 25:38

Well just that like usually um in immigrant families, especially like black immigrant families, all of those chores are the child's responsibility. Uh My parents do do the cooking. Um but we do all of the cleaning and like the work around the house and we usually help them like my mom needs help with cooking and stuff like that always goes with us um but she does the cooking because She thinks she does traditional food better than we do. So uh (laughs) yeah, but um, yeah, I think um, I'm pretty sure it's a common experience, I find that most of my friends who also are uh first generation Americans, they're used to doing most of the household chores and taking on things like that, like, with the groceries every Saturday, I just like take whatever's on the list, and I just go get groceries real quick, and then come back. So those are just chores that I'm used to having.

Moderator 26:26

Okay. Oh 60, was there anything else?

60 26:30

Yeah, sorry. Um, for me, the only difference that I had to uh probably do was take people's temperatures in the beginning. Um that was something I had decided to do because um at the time the only symptoms we had was like for possibly contracting COVID was like a high fever. Uh Now we know loss of smell and taste, you know? So sometimes a degree of temperature body change, you won't feel yourself, but we can feel with a ther.. thermometer Excuse me. My sister has one. So it was mostly checking my her and my mother's um uh temperature because my sister went outside way more often than I did because she had a lighter schedule. But my mom, you know, she has pre-existing conditioning and whatnot. So uh she, what do you call it? Um, we took a temperature first too, but in the beginning because she had still had been going outside before this, but she's not hasn't left the house and so there's no need to anymore. And at least in Redacted, we're down the curve. So we're still cautious but not as so hyperactive. Like we were in the beginning. Um Yeah, and so I agree with 59, you know, immigrant children, they sort of you you teach them once and at that point, sort of do everything for them unless they decide to do it. themselves, but I'll be going groceries for myself and my mom. My my List will like triple because my mom says go get the whole store and they come back, you know, and I mean, Um yeah, but it's pretty much the same as what 59 said.

Moderator 28:00

Okay. And to what extent where you're expected to take on more household work, childcare or family caregiving responsibilities than other people in your home, for example, was more expected out of some people than the rest of the people in the household. Once again, if you've already touched on this, you don't have to repeat yourself if you don't want to.

59 28:23

Right. Um, so yeah, um, my child care or like family care, that hasn't changed, actually, um, my mom works in the hospital. So she's been going to work and my dad. He's a government employee, and he's been able to stay home and he actually is retiring soon, in like, a couple months, um so he's been saying home. So I think those responsibilities actually probably been shared out more than usual than us if I was in high school and both my parents were working. Um Yeah.

60 28:56

Uh for me, other than you know what I've already said, it looks like the cleaning and the extra grocery shopping, there really hasn't been that much difference. Um My mom, she's she stayed at home my dad, he's an essential worker, but there uh for him, it's actually a bit better because there's less people with. He's a valet, so there's less people with cars coming in. Because there's a lower density of people, there's less of a chance for him to even be exposed. He's still being careful, but uh things haven't really changed too much in that aspect.

Moderator 29:29

Okay. So now let's talk about how your academics were impacted by the shutdown. I'm going to ask how the shutdown has impacted a variety of things. But if you don't think it impacted you, you can just say, no impact. In what ways if any has to shut down impacted your academic performance, aka grades and your understanding of the subject matter if your courses? (Pause) 60 do you wanna (someone starts talking at same time) Go ahead, sorry.

59 29:59

I think it would, oh oh overall because it wasn't expected it would have a negative impact. I think if they had more time to prepare, um it could have been fine. um Like going into fall 2020 my school has gone kind of a hybrid situation of where the larger classes are online and the smaller ones you can meet in person. Um But even some of my smaller classes have said that they won't be meeting in person at all. Um but because that they are aware of the change, they'll have more time to have materials um and more stuff prepared for us so I think now it'll be better than last time so it definitely had a negative effects. Um um I think I mostly learned from like face to face. Anyway, like I pay a lot attention more coming home. I think. Um I think college comes with it's own distractions but so does home life as well. I'm obviously with my family all the time. Um We can go different places and college I was really only stuck on campus. I didn't have a car I couldn't really go anywhere. Um but there are obviously things to do on on a college campus so that part was distracting. Uh I think with everything, there was an adjustment and I was still adjusting to college. So moving online was just another adjustment. Um so that's, that's what happened for me.

Moderator 31:11

okay. And so to um to make sure I heard everything, you saw a decrease in both grades and understanding of subject matter?

59 31:20

Oh, so, um, I think, huh how do I put this? Well for grades, I thi, I,...I'm sorry, I'm trying to think of a way to put this because our grades change like they got like for the class I told you about like bio English and then the genetics class I was taking um those got easier because they were making it easier on us saying that like, okay a lot of this is going online. So this test is now open book, um things like that So that made it easier. calc um it did not get easier. It got har harder, because the way that they changed it, they added more tests, they added more homework. So it was doing twice the amount of work that I would have done if I was at school. Um which was absolutely ridiculous. I hated that class. I talked to the coordinator so many times. And he kept saying like, this is what other schools do. So this is what we're going to do. So things like that, that definitely had a negative experience. Um And that's part of the reason why I decided to take that class as pass fail, I opted out of taking the grade for that class. So for math, there was definitely a decrease. Um But overall, I think the rest of them showed like the same, the same, um like what I would have gotten if I was at school.

Moderator 32:31

Okay, thank you for clarifying that. 60, did you want to touch on that?

60 32:37

Um for me, my grades definitely were affected, especially compared to the fall semester of um 2019. Uh I managed to bring my GPA up uh dramatically uh last semester and then I did also have the option of credit no credit, it's technically pass fail, but with credit, no credit if you pass you get the credit. If you don't pass um you don't get the credit,but your GPA won't be affected. Um, I ended up having to do credit no credit for all of my courses because they were just so low that it would drop my GPA um uh immensely. I ended up unfortunately, um not passing calculus, which was no fault of my own, in my opinion, because of the way they had done that final, which again was 65% of our overall grade. It wasn't your typical math test. You know what I mean? They tried very hard to make so that the students would not cheat. So they did very word filled problems of like definition type things um for calculus, you know, like theorems and whatnot like I understand you know, cheating is obviously a possibility because you can just take any regular app and then scan the thing and then you're good at the same time it to I feel like you're gonna try that hard to make sure we don't cheat there's also a chance you're making it that hard for us to you know, fail. So I already had I was in the A-B range before this, and um remember, I had no assessment afterwards. So it was all up to me to see how it was going. And I was doing pretty well up until that point. So, you know, with that final it was all or nothing. Uh for my other classes, bio did become easier, because for some reason I was un-, able, excuse me, I don't speak english well (laughs). I was able to better understand uh um the things in bio after the fact because it wasn't so hands on that you couldn't explain in much detail, certain topics without being um physically there. So it got easier than the rest of my courses. I took micro and macro economics as well. Uh micro economics class was already online, so really nothing changed. The only problem is that my school we had a lot of breaks in between March and April. The week that we came um the week that we went Inside our homes, we had a recalibration period is what they called it. You know what I mean? Just to have the staff and everyone prepare as some way to do courses online and then we had class. Then we had another break because we needed students who weren't able to have access to the internet, get laptops and everything. So we had another break for a week. Then we had a three day spring break (laughs).So it was like class, no Class, Class, no class. So now I'm just like,what are we doing? Um, you know, having such abrupt things happen. I know we can adapt in Redacted, but gesh so in the first few months, the first few months, it was very hard to have a strong foundation. Once we did that, you know, were um some classes the professors they just weren't there anymore for my microeconomics class he saw no reason to be teaching when he could just find the video on YouTube and then that would be that so it was all up to us. If we asked a question we get it. It was I was very detached because like 59, I prefer to be inside a classroom. I can see what you're saying, you know, because I'm not seeing anyone's faces here. I'm all I'm seeing is a screen. Um So it was a much different learning experience. My Grades did drop. Um, did I answer all the questions? I'm sorry?

Moderator 36:25

No, I think you did. I think you did.

60 36:28

I feel like I was trying to not rant, because the changes were a lot.

Moderator 36:35

That's totally fine. We're here to collect the full breadth of information. So you're doing great. Um in what ways, if any, has a shutdown impacted your long term career goals.

59 36:53

Um I I'm planning on going into epidemiology. So this uh probably boosted my prospects I think, um not to say that I'm glad or anything like that. It's just something that happened. So um I think actually, before going into this year, I was pre med and I just because I wanted to do research, um but I didn't really want to be a doctor. I just wanted to do research. So I'm actually going I learned more about the fields because of COVID-19. So I'm actually going into epidemiology now to study public health. I don't know what if I'm doing data science, public policy or focusing on the biology side, but I know that's what I want to do. So it didn't negatively impact my future career.

Moderator 37:41

Okay and 60?

60 37:44

I wouldn't go as far to say that it impacted my future career because like you know, we have a lot to live for but um in terms of completing my degree, it did delay me because of that one class that I did not pas that was the only class stopping me from applying to the engineering school that I wanted to apply to within my my my school, um Computer Science because it's a um I believe my campus is the only one that has an actual engineering school within our campus in the state. Uh so I need to fulfill certain requirements before applying. And I had to be at least taking calculus two because there's three calculus courses um for my college, I had to be taking calculus two and taking a second science course by the time I applied, which would have been in the fall semester, but because of this mishap, I have to wait until the spring of next year in order to apply meaning that I have to, first, then figure out what classes I'm taking in the spring because I'm done with my gen- general requirements. And then computer science has 50 courses alone as the major so I will its delaying my graduation essentially. Which financial aid wise isn't a problem as of right now, but because of all the changes that have been happening its not necessarily the pandemic but the response to that pend, to the pand,English, the response to the pandemic um weren't the best choices because it was so abrupt. So now I have to uh recalibrate my own ideas and whatnot. And I'm planning to add another major, economics because I see how I like it so much. So I'm basically double majoring. So I'll be in school for a while, hopefully, before I had my first kid, but you know (laughs)

Moderator 39:37

okay, okay. Um, I'm actually going to touch on, I guess the impact of the pandemic on the time it took to complete your bachelor so we'll actually end up circling back around to that one too. Uh but for now, in what ways if any has the shutdow impacted the availability of other learning opportunities such as internships?

59 40:08

Well, because of the shutdown, like, um it definitely stopped because we have internship fairs at my school. And that's usually where we see everything. And I wanted to work in the epidemiology department at my school, um which is two hours away. So I'm now unable to do that. I couldn't do that this summer either. um So that's disappointing. um I think definitely opportunities decreased after that, um, Even I was I was going to shadow at a hospital um and it was in the pediatrics department, and obviously, that is a high risk area. So I wasn't able to do that either. Um so it definitely decreased opportunity as a whole.

Moderator 40:44

Okay. And, 60, what about you?

60 40:47

Yeah, I did apply to quite a few opportunities over this for over the summer. Uh I usually do this um probably a month into the spring semester because you really have to with internships, especially technical internships you have to start early in order to get like a good spot. And a lot of them were either canceled completely due to risk a risk of a pandemic, some didn't want to say for whatever reason, and the rest I got rejected to, which wasn't that many. So it really decreased my options. I saw a lot of students scrambling, you know, posting their resume on LinkedIn, which I really don't see often, um, because, you know, your profile itself is linked on LinkedIn. Excuse me, your profile itself is sort of your resume, but they actually posted their actual resume on LinkedIn for everybody to see, just to look for an opportunity because three um, college is different from public school. We have technically three months of vacation for three months of doing completely nothing. At least, Um academic like officially academic wise is um not an option for some people. So uh also, other programs that I do in the summer are canceled because they were always going outside to do so everything completely like shut down, because like that's something that no one was preparing for.

Moderator 42:11

And similarly, did you guys notice any new learning opportunities or career development opportunities emerging during the shutdown? (Pause) And if no, that's, that's, that's the simple answer too.

59 42:29

um, well, for me, I started taking like these Coursera classes. So like that was helpful. Some of them are on COVID-19 and contact tracing, um which was further into my education because then I'd be able to be a contact tracer and that would be a really good opportunity. But I'm just now starting out because I'm, I just finished my first year. So I don't really have a lot of experience under my belt to be able to do those opportunities. But because of those field I want to go in. Those are the kind of things that opened up for me since COVID is kind of like kind of in the ballpark of what I wanted to deal with in the future.

60 43:06

Uh I actually also started those Coursera courses because they're free for a limited time. I think in a few days, it won't be free anymore. Um, because I haven't um gone into the business major yet. I'm still looking into it. But I have been taking a few entrepreneurship type courses on Coursera. So it is allowing me to go deeper into stuff that I'm interested in. I did also uh get opportunities to do stuff like contact tracing, I got a few emails on that, but I just forward them to people who might have been interested in or they just needed um uh ways to make more money because they also are looking for things either they lost their job, or um they also got rejected from internships its just that it wasn't in my particular field, nor did I care for it. So I did get those opportunities. I just did not, you know, choose them.

Moderator 44:00

Okay, that makes sense, in what ways if any has to shutdown impacted your confidence and in your ability to successfully complete your bachelor's degree at all or in a timely fashion? 60, I know you were talking about this earlier, Is there anything you wanted to add to this one?

60 44:17

Um, to be honest, I think its helped me, it's, you know, throwing something at me that I wasn't expecting, because I sort of had this plan to finish in 2022. And then be on my way to whatever career choice I wanted, but seeing as it just, it just sort of gave me a wake up call saying anything can happen. So I'm now i'm telling myself I want to graduate in 2023 not that far off. And, you know, uh seeing how my classes would go, it is delaying me because I had this not happened I would have still probably be, been able to graduate in 2022. But Um seeing as how anything can happen that has helped me be more prepared and more thorough. In any type of planning that I'm doing academically or not, but in this case, yes academically.

Moderator 45:07

Okay. And 59, how has your confidence in your ability to successfully finish your bachelor's degree in a timely fashion been affected?

59 45:18

Uh we were still able to get the classes that we needed uh and it's just like the regular like scheduling thing that messes everyone up. Um I don't think it like overall changed because in my school, we actually don't declare a major until the end of your second year. Um So I don't think it affected me that much actually had a lot more time to figure out like what I was going to do and what major I was going to pick and plan out all my classes, but I don't think it affected me negatively.

Moderator 45:49

Okay, in what ways if any, did the campus shutdown impact your finances?

59 45:57

Well, my school had promised to adjust Tuition and um housing after we went home in March since we there was like two or three months that we um were paying for and we didn't get. I have not seen that money other people have, I guess it just like depends on how it works. I don't know. I'm gonna call them soon so... Um I think financially it hit everyone. Colleges are a business like everything else, and they still have um the procedure to make sure that they're like their tuition prices haven't changed. Um They're not lowering it because most of its online Um their- I think the reason why my school introduced the hybrid system is so they can still charge us for being there. Um While also like having online classes so we don't get sick. Um So things like that, that's definitely given me a financial hit. And I actually, if it was truly a hybrid system, I would choose to stay home but I'm an RA this semester. Um I signed up to do that last year before I knew this was gonna happen (laughs). But if I decide to stay home, then I have to give up my RA position, which I don't think is fair. Um so things like that. And I actually, like I got the RA position because of the fin- the financial reasons, like primarily. Um so, yeah.

Moderator 47:15

Okay, thank you for sharing that.

60 47:18

Um, for me, it's been sort of flip floppy, to be honest with you, um, they just recently raised tuition at least here I don't know about other states, but here, about about 1000. I do receive financial aid from, you know, federally and the the Pell Grant and through the state, so I'm good for now, but that's only for a limited time. And that the amount that's been that will be able to be given in future semesters. Means due to all the economic things going on uh Redacted did give an emergency grant recently to people who do receive financial aid and I think who fit into other requirements as well. I did receive that, um from my spending, I actually saved the money because I wasn't going outside buying outside food because it was outside all the time. Uh I didn't spend that much money on laundry because I'm not wearing outside clothes that often either but this is only a temporary thing, I am getting older and I'm buying more things that I need. So this was more of a temporary thing happening. Um, later on, I will have to seeing as you know, prices are most likely gonna go up, I am going to be spending more money on things including groceries and everything. uh And with my financial aid, it definitely there's a chance that it will be affected. There was also there was a scare not that long ago that people were not getting their tap, which is Redacted state aid, right? They might have just disappeared, literally like it wasn't there anymore, and then I would have to owe money. It did go back. But, um, things are are sort of wonky and whatnot. I think it's 2020 curse just saying, but (laughs) it really is unstable. So it's really hard to determine where things are gonna go, here.

Moderator 49:13

Okay. Um so now let's talk about what professors did and did not do during the move to online learning, what are some examples of strategies, tools or technologies that Professor used tha- that helped you learn, and what were some of those same tools that did not help you learn during online learning.

59 49:38

Um so, I'll like to use the example of my biology versus my math class. So for biology, they introduce more um um open discussion forums. So before we usually have like talks in class, and then we have that lab period to discuss things that we didn't understand before. So the teacher this year, which is completely new, she opened like Piazzas, so or something- I think it's called that um and she had it so students can ask questions to other students. And then students can ask questions to her and then um if someone else knew they can answer it. So it was very, like open. They had open discussion. Um, She also provided like the notes online and it wasn't live. So she allowed us to see it at any time. And because it was recorded, and we had that question forum, it was, I think it was very much easier. For my math class, things I did wrong was obviously keep the live um the live lectures, which I think a lot of students had trouble attending sometimes just because since you're home, you may be doing like your schedule is obviously much different from then, if you were at school, like you can't block out the same amount of time. So I think they could have been a lot better with that. Um Other things that they added um they change the schedule as a whole to reflect the fact that we were going online. Um so and the fact that we were in a pandemic, I feel like that It just needs to be addressed. For one like my math class, at no point, did they ever, at no point were they were like, okay, we understand the stress that you're under, and we understand that you're adjusting um so this is what we're going to do. No, they said, Oh, because of this, we have to add more, which didn't make any sense to me, because we were all trying to figure out how to fix the situation and obviously, we're more settled now. But when it happened, it was so new and raw, and they did nothing to help us like our physical or mental health at all. Um so my biology class on the other hand, she took students suggestions very seriously. And she talked about it with us. If she made a decision that she thought um um wasn't in our best interest or wanted, she wanted feedback on she made sure to explain it and explain her side explain why she can't change certain things and why she kept certain things the same. So communication was huge. And I think that the calc class that I was in, didn't do that at all.

Moderator 51:57

Okay. Thanks for sharing that. Um 60?

60 52:03

Oh I'm sorry, I'm sorry. Um, aside from the pro-, the departments as a whole, it really depended on how the the professor, how prepared the professor was in general that determined how things were gonna be later on. Like, for uh an example, with my calculus professor, she was just a prepared person in general. So she was able to transition very quickly. Um, she was always, always on point to begin with, aside from, you know, the rules that he had to abide by because she is under the math department. You know, she had a, she asked the students what times are available for office hours if you have questions. Um She always told us our email, er her email, you know, she was always communicating with us. Um and we, we had different uh professors have different options of where they wanted to do their courses. So we we use Blackboard and In Redacted, I don't know about other states, I think other states do use it. So we had this thing called Blackboard Collaborate Ultra, where you can still talk to students um, and broadcast wha-whatever you're teaching. I did this for two classes. My other classes, we already use a separate platform. So that was a struggle, because depending on what your professor decided, it wasn't in one same place because I use Tophat for my macroeconomics, and then Blackboard for my two classes and then for lab, I use zoom, it was like all over the place. Um And the times, the times of the classes didn't change, so I'm glad that that stayed the same. But um be--, uh because there was no unified way of teachers going about things-- It was stressful on top of that. Um but what I did like for my bio professor, he already had discussion boards ready, like that's what he did beforehand. So he was prepared for this because all he had to do was put the lecture online and then figure it out what to do with the labs, we were still able to communicate with each other whenever we did, he's very responsive to emails he will respond to you in like 15 minutes. And he has a student body of like 400 people so I'm very impressed by that. Um And he also had the live lectures able to be downloaded and looked at at a later time. My um Calculus Professor only did this sometime so uh we were able to--- no I'm sorry, we were only able to download a few of them but both classes for Blackboard Collaborate were recorded so when I was late to one of my classes I was able to go back and see what I missed. Um, that really wasn't a problem for me in terms of catching up but still online is online so least I get it I get if I don't I don't I there's not much further I can do other than talking to actual people to further understand the content, but the changing of of sources and and and abrupt um Excuse me, oh, wow, I really lost my train of thought there, the changing of platforms back and forth depending on what class it was really was frustrating because I had to um move from one place to another and then like, change my thought process depending on the course.

Moderator 55:18

Mm hmm.Okay.So what are some things that professors did that made you feel like they cared about their students? And opposite of that? What are some things professors? Did that made you feel like they did not care about their students?

59 55:35

So yeah, I kind of touched on this before, but just the open communication and transparency um of why they were picking certain procedures, why they chose this type of curriculum. Um I think just explaining that to students um really put a lot of us at ease, with biology, and English, um and then giving us like it for English because she knew that people had internet issues or they have like other things to do at home. Since either have added responsibilities at home. Um so she gave us sometimes extensions, like more extensions than she would have um if we were a day or two late with discussion forums that we didn't answer. Um, she understood. Um my math class, on the other hand, whenever I asked him a question, he seemed like he was deflecting it. Um And he he truly just couldn't explain why he chose to add certain um measures and the only thing he said was because other other schools did it. Um So it seemed like he um couldn't explain it himself. So he lacked transparency and communication.

Moderator 56:38

Okay, and because he was not communicating with you, it seemed like he didn't care.

59 56:42

Exactly. It it just felt like um he said he was doing it for himself or he said something about the integrity of the class but couldn't explain like how it threatens the integrity of the class um to make sure that certain measures were um uh suitable for all students. He just didn't take that into account at all. So, yeah,

Moderator 57:03

okay, okay.

60 57:06

Um, for me it depended on the course surprisingly, even for my online classes, my micro economics, my professor was still very um open to curving for us because he did understand that there were special times and microeconomics isn't particularly hard. So for the fact that you're going to curve on a class that wasn't so difficult, I really did appreciate that. And he was very responsive to any of the emails that anybody had. He would actually email all of us at once if one student had a particular question just to make sure that we were all on the same page. Um for- I think for my macro economics course there was the least amount of communication because um like I said, Professor, his lectures he would just send us a video and call it a day. Um but for my and for my biology course, um, the, the threshold as to what grade you need in order to pass the class did change before you needed, I think a 70 something I think that's like a low b a b minus or something like that. But all you needed to do to pass the course was get a 67. So I did appreciate that for my bio professor, I think the weirdest mix of um consideration for the students was calculus, because my professor, she understood that some people still work, you know, some people, um they don't have good connection on their computers. She understood that at the same time, she sort of just said, do what you have to do. So I do realize that she was aware it's just that, you know, she can only do so much. But there was also uh with the studying that we had to do like unless you had um um a a legitimate reason as to why you couldn't be doing certain classes. She either said, maybe you should just drop the course or, oh, you're at home now you have all the time in the world. You should be studying more this and that and I'm just like, well it don't work like that too much, but that's her mentality because for her, she can cook while teaching and she actually was cooking while teaching. So it's like if I can cook while teaching and still yell at my kid, you can definitely study more. You know what I mean? Like, it just you can't compare your life to everyone else's it just doesn't work that way.

Moderator 59:21

Okay, so by projecting that expectation on me, you felt like she didn't care. Got you.

60 59:26

In in some in some aspect. Yes.

Moderator 59:29

Okay. Okay. So now let's talk about what your university responded to the how your university responded to the move to online learning. What are some things that your university did to help students be successful during online learning, for example, pass fail options, town halls, remote counseling, etc.

59 59:51

Um so my school passed to an optional pass fail option. Um I actually don't think I'd prefer that I think they should have made it um required. Just because um I was pre med at the time and I know this is a problem with a lot of people who are applying to graduate schools, medical schools and other professional schools is that if you have the option to take it as a as a letter grade, um they want to see that letter grade. Um so it didn't help that we are making this change and that even if I wanted to take it pass fail, I was encouraged to take the letter grade even though I didn't think I was like completely ready. Um so for that part, my school did cha- change. They tried to adjust but I didn't think that what they chose was the best option for everybody.

Moderator 1:00:37

Okay, and 60,

60 1:00:41

Um, my school is under Redacted. So I go to Redacted and under, you know, the Redacted University of Redacted, so the Redacted school as a whole the univers-, I feel like my school they did a lot. They offered a lots of help. They made sure that every student has some form of internet access um available so either gave a a laptop, a tablet something in order to get our work done. Um We have a counseling we have a psychological center and a Counseling Center um in on our campus so they were providing mental health services um to anybody who was stressed out. They even um had a not a forum um sort of like a group, uh talk about support the Asian community because they were getting a lot of backlash from the Coronavirus starting in China, which we know is not based on race, it doesn't discriminate. Um Mental Health Service wise, financial wise, we were all offered a lot of um support. For the pass fail, we were offered credit, no credit, it was an option. But they did um recommend that we speak to an advisor and we speak to our financial aid advisers before making the decision just to make sure that we're making the right decision. I doubt anybody with an A wants to just get credit, you know, and I mean, you just keep the A, but in terms of any programs that you're applying to, um, within the school any programs that you were applying to what any requirements that need a specific grade for the semester only unless we see things changing later on, they will be bypassed. So let's say for my um for applying for computer science, I get a D but I needed a C in I don't know, Bio right, if I add, if I qualify for credit, I can still get into these programs for this semester because of its um they considered it a a great academic disturbance. So, you know, those rules don't apply this time. Um, so I think they in terms of the accommodations they made as a whole, not, you know, with the little classes, but as a whole they did a really good job. As far as if people took that advice, I'm not sure I can't speak on that.

Moderator 1:02:56

Right, right. Okay. We are out of time for today. So we're gonna have to email you the last two questions, but they shouldn't take you long. Just remember to respond to that email with the answer to your question and with the name that you're using here today. So thank you all for coming today and I am ending the group now.

59 1:03:21

All right, thank you. Bye

Moderator 1:03:23

Bye

Emailed Questions

1. What do you wish your university did to better help students be successful during online learning?

2. In general, are there any ways that your ability to be successful in school was affected by the pandemic that we have not discussed?

**Survey ID 59**

**1.** I wish my University would be more transparent when it comes to financial issues. They've also hinted at next Fall being a "short semester" so I wish they would just make a final decision and have everything online rather than half online/half in person.

**2.**

- Internet issues
- Distractions of being at home, wanting to hang out with my family, being back in my hometown.
- Lack of resources from certain classes (Calc) and the increase of work to makeup for time lost.

**Survey ID 60**

1. I do wish that we had the option to come together as students to speak with the people making decisions for us. Communication is key but it has to go both ways. Not just official talking to students.

2. No, anything that affected me was mentioned in the discussion already.

**High SES Black Women**

Moderator 10:51

Those who just came in. Hi, welcome. We're just waiting on one more person so I'm going to give them a couple more minutes and if they don't show up by them we'll just get started. But either way, we'll be started soon.

Okay. Um, let's just go ahead and jump into it. Welcome to the focus group. I'm Moderator. I'm a research coordinator from [Redacted], and this is our Note-taker. She's going to be our note taker today, Note-taker, do you want to say hi?

Hi everyone. Thanks for coming.

So we're going to be asking a number of open ended questions. There aren't any wrong answers but people might have differing points of view. Feel free to share whatever your point of view is, even if it differs from what others have said. Keep in mind that we're just as interested in negative comments as well as positive comments. We just want everyone to feel comfortable participating and encourage everyone to talk. Because we have to keep this to an hour, I might have to cut you off so I apologize for that in advance. We want to get the full breadth of experiences and opinions but some of you might have very similar experiences and answers and if that's the case, you can just say, my answer is the same as so and so's. It's difficult to take notes on everything that happens in the group, so we'll be recording this session so that we can transcribe it later. But we won't associate your name with anything that you say today. You have the right to refuse to answer any question or withdraw from the group at any time. We understand how important it is that this information is kept private and confidential so we'll ask that participants respect each other's confidentiality and not repeat things said within the group to anyone outside of the group. Are there any questions before we get started?

57 13:20

No.

Moderator 13:25

All right, so with that being said, why don't we go ahead and just go around the room. If you could please say your name um and what year of college you just finished, and one word to describe what life has been like during the pandemic.

57 13:47

Hi everyone, my name's 57. Uh just finished my freshman year of college, and life since the pandemic has been.

Moderator 14:06

Oh 57 I think we lost your audio.

57 14:10

Oh, can you hear me?

Moderator 14:11

Yeah. Uh what was the one word that you said?

57 14:13

I said busy.

Moderator 14:14

Okay, busy.

40 14:21

Hi, um my name is 40. I just finished my sophomore year of college, and one word to describe all of this has been stressful.

Moderator 14:33

And would you rather me call you 40?

40 14:35

You can call me 40. That's just my nickname.

Moderator 14:38

Okay.

33 14:40

Hi, um my name is 33 and I go to University of Redacted, about be a senior and one word to describe all that's going on for me, I would say like evolution, because I think I've grown a lot through like these four months or so. Yeah.

Moderator 15:03

And can you say your name for me one more time, please?

33 15:04

Yes, uh 33.

Moderator 15:05

33.

33 15:07

Yes.

Moderator 15:10

Okay.Thank you.

62 15:12

Hi my name is.

Moderator 15:18

Hello? I think we might have also lost your audio. Can't quite hear you.

62 15:52

I'm gonna log back in on a different computer and see if that helps.

Moderator 15:56

Okay, we can hear a little bit better now too, but whatever you want to do.

62 16:00

Ok um so my name is 62. I just finished my first year of college and one word to describe this would be interesting.

Moderator 16:13

Okay, interesting. Nice. Okay, so the COVID-19 pandemic has presented big challenges for undergraduate students. As you know, universities across the nation have closed, moving all instruction online with very little time for students or faculty to prepare. Many students have encountered challenges related to the closing of campuses, relocation, and the movement to online instruction. In this study we want to learn how the closing of campus and the pandemic in general has affected you. We want your opinions about the good and the bad on how things went as well as what your professors or universities did that was helpful and unhelpful, and what you wish they would have done. Let's start by discussing where you went when your campus shut down. I would like to hear from all of you. So, could you tell me about your space in the home you moved to? What space do you have to do your schoolwork there?Comment on whether the space is private or shared. And [redacted], if you want to start us off.

57 17:10

Oh okay yeah. Um I went back home um to a a two story house. Um my study um area was pretty much my room. It's private because I have a door but my parents are very loud and sometimes they like to walk into my room, and see what I'm doing and interrupting class. Um so that's kind of what I went back to. Yeah. I think the answers the question.

Moderator 17:39

Yeah. Okay.

33 17:43

Um so for me, I had to go back to my house and I live with seven other people: my parents, grandma, uncle, I have a twin sister, a younger brother and an older brother. So we all were in the house. I have a room but I share it with my sister and I don't have a desk in my room. So um, during that time we had to set up like a little space in the basement for us, for my sister and I, and it was a little tiny desk. It was kind of hard for me to focus at times, but I think things are now like a little bit better. I actually got like a bigger desk, another monitor so things are better now than it was before.

Moderator 18:24

Okay, and so are you the only one who uses that desk or do some of your the other people in your house use it too?

33 18:29

So um, I'm in the basement and um I have my own desk and then my sister's kind of like right near me. She has her own desk as well so.

Moderator 18:39

Okay, so you shared the basement, but not the desk?

33 18:41

Yeah, correct.

Moderator 18:42

All right, and 40. What was your space for school like after the pandemic?

40 18:49

Um pretty similar to the other people that spoke. I have my own room, but like I live in a house that with on average five to seven people, so it got pretty loud, um and I don't have a desk in my room, either. But we do have a desk in the living room but then working in the living room, it's a lot louder and there's a lot going on. Even in my room I can hear everybody so it's just a lot.

Moderator 19:13

Okay, and you did most of your work in the living room desk where other people were?

40 19:18

No, I actually did most of my work in my room, but when I took exams and stuff I'll go to the desk.

Moderator 19:23

Okay, and your room is just yours?

40 19:26

Yes.

Moderator 19:27

All right. Okay, and 62, what was your workspace like?

62 19:33

Um, so I came back to my bedroom. Uh I, it's just me in my room. But um two rooms on either side of me, my mom works in one room and my dad works in the other. So I can hear like their meetings throughout the day so it's really loud. Um I don't have a desk so I do all of my homework on my bed, and um my brother is in the basement and sometimes my parents take meetings in the basement so there isn't really room for me to do anything other than sitting in my bed.

Moderator 20:09

Okay. Um so next up, what challenges did you encounter attending classes or doing your schoolwork in this space? And you might have already touched on it, but feel free to elaborate now. And 57 we can start with you again.

57 20:23

Um the studying in my bedroom. It's it's really like a simple thing but I don't have a desk either, like everyone else mentioned, and studying in your bed is just not a good idea I don't think, at least for me personally, because you really want to take naps all the time. Um and the other thing that I guess was harder for me adjusting to school this way was um one of my professors in particular, they just decided to make class harder.

Moderator 20:58

Okay.

57 20:58

I guess because he thought people could cheat. So, our tests just got way harder and that was a difficult adjustment for me.

Moderator 21:09

Okay. And um I'll ask about professors and their strategies and technologies and things like that later as well so I'll give you more space to elaborate on that later on. Um but for now, are those all the challenges you saw with working in the physical space you were in?

57 21:27

Yes.

Moderator 21:28

Okay. And um 33.

33 21:34

Yeah so um, with me, because I live with so many people I have to tell them okay I'm taking an exam at this time. Please don't bother me everyone. And then, you know, there's noise during the exam so I have to text them be like can y'all just please keep it down or I like I'm really busy. I'm trying to study. So, sometimes I had to like, they all go to bed around like 9pm or so so I would kind of wait after then to study, so I won't have any disruptions.

Moderator 22:05

Okay.

33 22:06

So just issues related to the people in the house.

Moderator 22:10

Mhm. And were they usually respective of your boundaries like when you told them I'm gonna have class did they actually acknowledge that or did they just continue on?

33 22:17

Yeah they they acknowledged it I would say. Um I mean, but they if they needed something they would still call me or whatever so.

Moderator 22:25

Okay.

33 22:25

Yeah.

Moderator 22:28

Um 40, what kind of challenges did you experience in your space?

40 22:33

Uh my experience is pretty similar to 33. Uh when I, one of the first exams that I tried to take in my room, like everybody was downstairs laughing whatever, even though I had told them I had an exam so I had to actually get up and go ask them to be quiet. And they were respectful and generally were respectful, but I still had to deal with like a lot of noise. Like when I'm just studying it's fine. I'm not bothered by it, but during exams that's when I had to like, ask them to not do that, and they listened for the most part.

Moderator 23:05

Okay, okay. So once again sounds like the people in the house are the challenges.

40 23:11

Yeah.

Moderator 23:13

And 62, what about you?

62 23:16

Um, so for exams, I had to like I would tell my family like I have an exam at this time for this amount of time. Um if my parents had meetings during those times there was really nothing I could do about it. Like I would just have to hear their conversation. Um, exams in and of themself depending on the subject were a little bit harder, because I'm more of a visual learner. So like, I like to write things down. Like I'll I'm the type of person that'll highlight certain things or underline certain things to make sure to draw attention to it and I didn't get a chance to do that with online exams. So that was a little bit more difficult. Um and studying. I sometimes I get distracted kind of easily to like hearing other noise and stuff, I'll just be like I can't work right now and then just stop entirely and that would throw off my study schedule, or like, I would be in a time where I really need to study and everyone else in my house was like, oh, like we finished everything we need to do so there'd be a lot of noise, and like I asked, uh oh like can you turn it down or something like that and sometimes it would, happen sometimes it wouldn't. So like it would, it just all depended on the day and on what we were doing.

Moderator 24:32

Okay, I'm getting like a reoccurring uh theme of it being the people in the house that are the challenges. Okay. Um so speaking of people, what are things that you or other people in your house did that made it easier for you to attend classes and do your schoolwork?

57 24:52

Can you repeat the first part of the question?

Moderator 24:54

Yeah. What are the things that you or other people in the house did that made it easier for you to attend classes or do your schoolwork?

57 25:05

Um, I don't necessarily think that um my family members in my house made it any easier. I think it made it harder.

Moderator 25:13

Okay.

57 25:14

From disruptions from them. I mean, some of them weren't intentional and, but some sometimes like I would put on my door, "taking a test" and yet someone would still come in my room.

Moderator 25:27

Okay. So trespassing those boundaries again. Um 33, what are some things that people in your house did that made it easier for you?

33 25:37

Yeah, so I think my dad was like kind of the most understanding, and he um got me a desk, like I mentioned before. It was a smaller desk, but towards the end of the semester he got me like a much larger desk, which is nice but um yeah desks. And then he cleared out the space for me to put the desk in and so that made it helpful.

Moderator 26:04

Okay. 40.

40 26:06

Um, yeah so my biggest issue was just the noise but other than that, everybody was pretty respectful. Like if my door was closed, they didn't come in, didn't even really knock until like I came out of the room so I guess that's, like, it was helpful.

Moderator 26:19

Okay and 62.

62 26:25

Um I would kind of agree with 40 at the beginning. Um, I well I'm taking summer classes now so they're a little bit better in the summer, but during the year I'd have to lock my door during exams cuz like family members will kind of walk in, so like they were, they've gotten better, but it started off as them being more like less helpful than they are now. Like now um my I'll tell my parents when I have things due and they're like, oh okay like make sure you remember this, or like they'll give me little reminders. Um so, I'd say I'd say it's gotten better over time.

Moderator 27:06

Okay. Um, so now let's talk about what other responsibilities you had after your campus closed and how those differ from responsibilities you had before campus closed. Describe your caregiving responsibilities including childcare, elder care, or caring for ill loved ones. How were they alike or different from your responsibilities before they shut down?

57 27:33

Um they were I didn't really have to care for anyone per se. Um most of since I I got home from school um my grandmother was staying with my mom with my parents um until about last week actually. So, I would help with her but she's in no way like fully incompetent where she can't do anything by herself. Um, and I didn't have to do anything like that at school but I mean I would obviously help like do like cook stuff for her so she just doesn't have to do as much work.

Moderator 28:08

Okay. And no like sibling care or anything like that?

57 28:12

No, no, sibling care.

Moderator 28:14

Okay. All right.

33 28:17

Hey, sorry about.

Moderator 28:19

No, it's fine.

33 28:20

So for me I I mean I didn't have to do anything like extreme. Um so I have a younger brother, he's 12, and he also had to do like online stuff, but I never had to like tell him okay, sit down and do that. My parents kind of did all the duties like that. But I have a grandmother who's like in her 70s. I had to tell her to like she has to take medicine at a certain time, so I have to like remind her, or check if she took it. And I also have my uncle who is diabetic. So, um he also has some issues related to that so I had to kind of check both of them at certain times but it wasn't like extreme craziness stuff like that.

Moderator 29:04

Okay, okay.

40 29:07

Um for me, during the spring semester there wasn't really that much I had to do. I do have a younger brother. He's eight, um but everybody was home at that time so it wasn't really something I had to worry about, but then I'm also taking summer classes right now so then my with everything sort of opening back up, or some things opening back up, um my dad has gone back to work and I have to make sure I take care of my brother, help him with his work a little bit and like, just, it's just me and him, most of the time during the day. So that's something that I don't really deal with when I'm at school. But it's also something that I deal with when I'm at home in the summer anyways so it's not like a COVID thing. I would have to do this anyways.

Moderator 29:48

Okay.

62 29:53

Um, I haven't really other than like basic chores that I didn't have to do in a dorm, I haven't really had to do much. Um, uh my I have a brother. He was taking summer classes and I'm really good at calculus, so like I tutored him in calculus for that. But other than that, I haven't really done much that I...do.

Moderator 30:21

Um so next, describe your other household responsibilities such as chores, cooking, caring for pets um during this time, and how they were alike or different from from before school closed.

57 30:35

Um, my responsibilities just pretty much consisted of like you know, washing the dishes, vacuuming, um doing laundry sometimes, taking out the trash. It's more just like household duties that I was doing in high school. Um they're definitely more than I had to do in college because I'm washing the dishes for more than just myself. I'm doing it for like the whole house after we eat dinner. Um but I still did this cleaning stuff in high school, it just the only difference is it's on a larger scale because, I mean, it's more people in the house now.

Moderator 31:14

And so, actually I'll ask you that about I'll ask about that next question um.

33 31:24

Okay so, um, with me um before coming back home I lived in an apartment so I had to like buy groceries for myself and all that. And coming home, I would say the duties are like way less cuz there's so many people in my house that we kind of just divide everything up. But I still like, buy groceries I don't know maybe like once a month for the house like if they need something I go out and get it and then um mopping the kitchen and all that stuff I still do.

Moderator 31:56

Okay. Um, 40 what about you?

40 32:01

Um, so I was living in a dorm for sophomore year so my, I had a meal plan so I didn't really have to worry about food or anything like that. But when I got back home, we have food and everything. I cook sometimes but I would do that anyways. Uh I take care of my own clothes and I wash my brother's clothes, stuff like that. But I feel like the responsibilities weren't super extensive, just like normal stuff you do around the house.

Moderator 32:29

Mhm okay and 62.

62 32:34

Um I agree. I don't think things have really changed much since um what I did in high school. Like I also had a meal plan so I didn't cook and things. Before like I would get snacks for the room occasionally. But now, like, there's food in the house for the whole family and so just cleaning up after everybody, mopping the floor, sweeping, things like that.

Moderator 32:57

Okay. Um so to what extent were you expected to take on more household work, childcare, or family caregiving responsibilities than other people in your home? For examples, did your parents expect more out of a um one sibling or did your roommates not contribute equally? Anything like that?

57 33:19

I didn't have any siblings living with me. My um my older brother he he doesn't he doesn't live with us. He actually lives in a different state. So, there was no no person I could really compare to him and sometimes I felt like I was doing more like cleaning than other people were but that's also what they make me do so.

Moderator 33:45

Okay. Why did they make you do that?

57 33:49

Um, it's always been a responsibility for me to um like clean. I mean yeah my dad will go clean, um especially sometimes when I'm like really busy or if I. We act-pretty much if I'm like really busy he'll like clean the kitchen after dinner but it's just one of my responsibilities to make sure I'm doing my part for the house, cuz like that's how my family sees like everyone should put in their part whatever that is. You know, if it's if it's cleaning or if it's money. Obviously I can't put in money cuz I'm not working like the type of job that my parents are but um just giving my way showing that I appreciate what they're doing.

Moderator 34:26

Okay, so your parents do the money thing but you do the house care things.

57 34:32

Yeah.

Moderator 34:33

Okay. Uh 33.

33 34:36

Yeah, so for me I think my parents took it easy on my sister and I, because we're in school so like they didn't really expect us to do like a lot of chores. Um just cuz they didn't want to add, I guess too much stress on us cuz like we mentioned like how we were like really stressed out being in this house so they understood that, and um so yeah it was less chores than normal.

Moderator 35:00

Okay. Okay. Um, and I wanted to ask, were you the only one checking in on your grandmother and your uncle?

33 35:08

No, it was the whole family basically.

Moderator 35:10

Okay. All right. And 40, what about you?

40 35:16

Um, like I mentioned before, like a lot of times uh taking care of my brother does fall on me if my dad's working or something like that. Um also I was never really asked to do like a lot of the cleaning stuff like dishes and everything, but if I don't do them they're just not going to get done, so I just did that.

Moderator 35:33

Okay. Okay. And 62.

62 35:41

Um honestly I think I did more in the house but my brother does like mows the lawn and things like that. Like he works more outside and I'll do inside the house. Um, but I would honestly say it's kind of easier now, because before I left for college it was just me in the house cleaning up after uh myself and my parents. But I know he's here to kind of divide the work. I'm cleaning less than I would have before. But um my parents also take it easy on me like um I do stuff in the kitchen, like my dad does the bathroom, so my brother works outside so it's all kind of like evenly split.

Moderator 36:26

Okay. Okay. Um so now let's talk about how your academics were impacted by the shutdown. I'll ask about the how the shutdown has impacted a variety of things and if you don't think it applies to you or there was no impact, you can just say no impact. In what ways, if any, has the shutdown impacted your academic performance, grades, and your understanding of the subject matter of your courses?

57 36:53

I think that overall my grades well not overall but yeah, my grades pretty much stayed the same. I don't really think in actually if anything I think that I started studying more for some for one class in particular, just because that class got substantially harder, but it didn't really affect me. Overall like looking back I think my teachers I think did a good job, relatively speaking at keeping class the same way.

Moderator 37:23

Okay.

33 37:26

Yeah and for me, um, our school my school um they implemented this thing called like pass or fail so you can get a P, which means you'll, you'll pass if you have a D or higher, and it won't affect your GPA. So I thought that was like a blessing because I had a stats class, I was borderline um, I had like a C or something so at the end I I took a pass for that one. I believe if I if COVID didn't happen and all this didn't stuff didn't happen I don't think I would have passed that stat class. Just cuz, like it was so hard so I just took the P. And as for the other classes I took um some of them I kind of like didn't really care about anymore, cuz it was I felt like it was pointless. I didn't really try. I didn't really study and I didn't really watch any of the lectures, so um I kind of didn't try as hard.

Moderator 38:25

Okay, so an improvement to the grades, but a disservice to understanding the subject matter.

33 38:31

Yes.

Moderator 38:32

Okay. And 40 what was your experience?

40 38:38

Me right yeah.

Moderator 38:39

Mhm.

40 38:40

Oh okay. Um, so my school was similar. Instead of pass or fail we had credit no credit, so that till you opt into that the pass fail, we um the teacher would have to assign that to a class. But I ended up taking three classes credit no credit and because the whole system is very stressful for me. I find it hard to learn without being able to like get the help that I'd normally get for certain classes, and one of my classes also a stats class um in that class I was doing pretty well in before all of this happened, but then the teacher was so concerned with people possibly cheating, that once we went online he made everything way harder to the point that I wasn't sure if I was going to pass or fail. So that's why I took it um credit no credit and it worked out but so so my grades overall were not like affected but I don't think I learned, as well as I wanted to this semester.

Moderator 39:36

Okay, that makes sense. Um 62, how was your academic performance and understanding affected?

62 39:43

Um, so I, there were only two classes that I had that could have gone either way. The other ones once we got off campus, I kind of ignored them cuz I knew I was going to do well in them regardless. Um so one class, I ended up getting an A in that class and doing better than I expected and the other one my grade dropped from a B to a C, because, um, like everybody's been saying, teachers were afraid of people cheating. So they would ask questions so the way that they did it is, um they would ask questions on the exams from like the pictures in the notes, things that they didn't go over in class and they're like, well, it was on the slides that we gave you so technically, it was fair game. So a lot of people started not doing well after that. And uh we started emailing them saying like, um, we get that you think that people are cheating. Like we get that it may be an issue, but is there like a compromise we could do cuz I was talking to some of my friends, a lot of people who were getting A's on exams when we were on campus were now getting F's on exams.

Moderator 41:00

Okay.

62 41:01

And so, uh I didn't really like the way that was handled cuz they kind of just told us this it is what it is. Like this is what's happening. So I don't think that was handled very well and that in turn negatively impacted a lot of people but other, I think, other than that specific department department everything else went pretty smoothly with the transition.

Moderator 41:26

Okay. So how did you feel like that affected your understanding?

62 41:33

Um, I didn't really get all of the material. Like I got a C in the class I chose not to do pass fail, but it's one major class so I decided to retake it.

Moderator 41:49

Okay.

62 41:50

And I'm retaking it now, and I'm going through and a lot of information like if this was how it would have been done in the spring, it would have made a lot more sense, because now they're saying like, oh, well, um, we're not going to ask you this question because we know it's difficult. So, I have a better understanding now because they realized that in the spring, they were asking for a lot and they aren't doing that anymore, but in the spring, they kind of just ignored the fact that they knew it was hard, and did it anyway cuz I think it was because a lot of people had made that class pass fail. So they didn't think that it would have uh impacted the people who didn't, but for everyone I know that didn't choose pass fail, their grade dropped a letter grade.

Moderator 42:38

Okay.

57 42:41

I definitely have to agree with that last part. What she just said.

Moderator 42:49

Okay. Um in what ways, if any, has the shutdown impacted your long term career goals?

57 42:59

Um like, I want to be a um orthopedic surgeon, and I'm on like the pre med track, obviously, and I was had study abroad program signed up for the summer. I was gonna um actually studying in redacted, working at a hospital and studying medical um Spanish. And I think like overall probably on the bigger spectrum, it hasn't necessarily affected me um from this spring semester but in the back of my head I'm always like concerned that I didn't get to do any um study abroad programs or research or anything to help build my resume for applying to med school. Um and also, I just found out I have to take organic chemistry online in the fall. And I know that's a very heavy class um for the MCAT and it's just a hard class in general, so I'm a little concerned about that and how that is going to hurt or potentially help I guess um my future career goals.

Moderator 44:09

Okay. So as of right now they're the same but you're kind of weary about this upcoming class.

57 44:15

Yes.

Moderator 44:16

All right.

33 44:19

Um, so for me, um, I was to be in Redacted for an internship. I'm a computer science major so I was going to be in Redacted for an internship um with like an investment bank. And um luckily they made it a remote internship. So, um, but they cut it down. It was going to be 10 weeks so they cut it down to six. Um I'm in the middle of it now. Um I have a few more weeks, but I think honestly like other than like not being in Redacted I feel like the experience is still the same, cuz with them I have to do zoom calls as well too but I honestly um I'm okay with like the situation how it's turned out um just thinking positively and. So I guess it hasn't really affected me that much other than like the position or the place I would have been.

Moderator 45:13

Okay. And I'll ask about internships and things like that later too. Uh sorry 40. Go ahead.

40 45:20

Okay, so we're not supposed to talk about internships or what was the question again? Sorry.

Moderator 45:25

Uh the uh question was, in what ways, if any, has the shutdown impacted your long term career goals? So like, is what you want to do with your life different now than it was before the shutdown?

40 45:39

Um I would say no. What I want to do is not different, but similar to what someone said before me, I am an engineering major so I was counting on having like an internship or something just so I could show that I have skills and I've developed skills, and I'm worried about like when I graduate, all I won't have had an internship this summer so that might hold me back a little bit, but otherwise like my long term career goals haven't really changed because of this, but I'm just worried about like seeming qualified after I graduate.

Moderator 46:14

Okay, okay.

62 46:17

Um I would agree with that, the whole seeming qualified part and um not really being able to go to programs. I um well for me personally, I am a biology major biology pre med major so I'm on the pre med track, and I don't know what type of doctor I want to be and I was hoping to figure that out this summer. So I don't have that opportunity so it kind of puts me behind while sophomore year a lot of people know that what they want to do and pick research based on what they want to do. I have to figure out what I want to do and then now take first year, do first year programs my sophomore year, to try and catch up to where I could have been from the summer. And I also wouldn't say that my career goals have changed entirely, but I know a lot of people say like, if you become a doctor slash when you become a doctor like a lot of your time has been gets put towards your craft. And I, for me personally, I don't want to be one thing. So like all the other ideas I have like I have an interest in film. I have an interest in like books, things like that. Figuring out a medium for like do I need to take classes for certain things like figuring all of that out like what I want to do has been interesting. I wouldn't necessarily say that I'm going that I would choose to change my career, but I have been thinking about just steps to take towards other interests.

Moderator 47:55

All right. Okay. Um in what ways, if any, has the shutdown impacted the available the availability of other learning opportunities such as internships? And if you already touched on this, you can um well, it's okay if you already touched on this I should say.

57 48:16

I kind of touched on this because my summer plans were the study abroad trip and obviously that was one of the first things canceled. Um I'm not just concerned on how this was canceled but I'm concerned on how future internships will be applying for um the spring of next year um to help build my resume for med school could possibly be canceled because at the rate that we're going like, I wouldn't be shocked. And I'm just really concerned how that's going to affect me when I'm applying to med school.

Moderator 48:51

Okay.

33 48:55

Um yeah, I already touched on it but I guess the big thing um like how they cut it down to six weeks instead of 10. So it just feels like a lot like the weeks are going by so fast. I'm on my fourth so um time is going by really fast. I wish it was a bit longer, so.

Moderator 49:13

Okay.

40 49:15

And then for me, I was originally supposed to go on a sustainability and engineering intern um not internship study abroad program to Redacted. I applied last semester but I found out pretty early on that was cancelled and I'm a little disappointed because I feel like I just missed out on learning about all the technology the green technology they use there. And then I applied to another internship in Redacted in February, and I got that, and then it was cancelled in March, because everything shut down. And then I applied for another internship in May, um near my area and then that was also cancelled because of the virus. So I just feel like I missed out on a lot of good experiences that could have, I guess I mean, because I know it that when I like graduate I'm going to be an engineer and everything but I missed out on experiences that could have shaped exactly what industry I want to go into. So now I'm just more conflicted than ever.

Moderator 50:12

Okay. And 62.

62 50:16

Um I applied to five programs, all of which were canceled. So that kind of was really disappointing. A lot of those I was really excited for uh different types of research, trying to figure out what I want to do. And I also uh kind of 40 touched on this, she mentioned how she's not sure how things are going to be next summer. I was uh intending to study abroad within the next summer or two, and now I don't really know where that's going to go. So, um, it's been um I'm just going to say I'm confused because I don't want to stay in a program, and not want to do it in the future. Like I want to figure out pretty quickly what I want to do, and I'm not getting the opportunity to do that with all that's going on.

Moderator 51:15

Okay. Um so did the shutdown create any new learning opportunities, or did any new development opportunities emerge during the shutdown?

57 51:30

Um, I think the one thing that emerged during the shutdown I started working a lot. Um I was already a math tutor at school, um but since the shutdown happened, we had to start working way more hours like sometimes over like 15 hours a week just tutoring students. And I realized that I I actually kind of liked that. I liked the helping people learn and um teach people math, um and also my other opportunity that I wasn't planning on doing cuz I wasn't gonna be here this summer but I started working at the tennis club. Um I'm I'm a college tennis player as well and I realized that I like teaching from that front as well so. Different experiences that I didn't expect to have.

Moderator 52:20

Okay.

33 52:23

Um I I none of them come to mind right now so I'm gonna yeah.

Moderator 52:28

All right, that's fine.

40 52:30

Um for me, I like the fact that a lot more summer classes are available now than I would have ever been able to take over the summer. Like classes that they never had online are now online so I've been able to take two classes that I was going to take over the next couple of years and now my schedule is a lot lighter and I have more room for another minor, so that's exciting. And then also I'm doing like a mentor program, and it's a a remote thing but it's been really exciting to do that so.

Moderator 52:59

Okay.

62 53:02

Um I would agree with 40. Um I have been taking a semester of classes this summer. I've opened, like I don't have to take more than 12 to 14 hours for the rest of college, so I thought that was a really good opportunity to come from this, because I I have asthma so I don't really go outside, just for fear of risk. So, I stayed busy taking classes and that's really the only opportunity I have been able to have.

Moderator 53:37

Okay. Um so in what way, if any, has the shutdown impacted your confidence in your ability to successfully complete your bachelor's degree at all or in a timely fashion?

57 53:51

Um I think, if anything, um this solidified the fact in me that I can actually graduate early if I want to. Um I came into college with um a lot of credits from AP classes, and then I took summer classes too because I was like why not, I'm here. And it just really accelerated my time track like everyone else said. So it's not four years for me, maybe it's like three and a half.

Moderator 54:19

Okay.

33 54:23

Um I think the shuts, I mean the shutdown. I I'm sorry, what was the question again? You said, how did it affect my performance in school, or?

Moderator 54:34

Um it was, in what was has the shutdown impacted your confidence in your ability to successfully complete your bachelor's degree at all or in a timely fashion?

33 54:43

Okay. So um yeah, I have one more year so I and next semester all of my classes are online so I think I'm gonna I'm on track to finish. Um so it hasn't really affected it in a negative way so.

40 54:59

Uh for me, I touched on this before, but I now that I took these classes I have more room to do a couple more minors which I wanted to do, and I don't have to worry about taking an extra semester, which is what I was worried about cuz I didn't want to have to pay for that. And now I can kind of do that and I can still graduate on time so it's exciting.

Moderator 55:19

Okay.

62 55:22

Um, I am I is inoperable. Sorry, I'm trying to think of the right way to say this. I could graduate early if I choose. Um so I guess that's a good thing to come out of this. The only thing I'm really worried about is taking the MCAT and how online classes will affect that because I also have to take organic online. I know somebody mentioned that earlier. Um, and the way things are going spring classes may also be online. So let's orgo one and orgo two is like a big portion of the MCAT, and in the spring I would also be taking genetics. Those are just like really big classes that I'm not sure how I'll do in them, considering it's not face to face, but um I don't have any problems with graduating on time, I don't think.

Moderator 56:18

Okay. In what ways, if any, did the campus shut down impact your finances?

57 56:29

Um, I don't think it's necessarily affected me. If anything, actually I'm working more hours now. So my personal finances have um grown but I know that for my parents, and obviously since they're supporting me um their finances have definitely shifted a little bit, um because my mom gets less hours and it's just my dad works for a job that you're traveling all the time and like, you're selling products to other companies. So, definitely cut back. Um so that's a little bit concerning on how like they're definitely not as financially stable as they were prior to COVID-19.

Moderator 57:19

Okay. And 33.

33 57:22

Yeah, so, um, I guess kind of affected mine but mainly my parents. So I lived in a my sister and I, we lived in an apartment throughout the whole school year and when they shut down our school like there was no point of us being at the apartment so we left. And we wanted to get out of our lease, but they said no. And so well we still left so it's like we're still trying to like not cuz there's around 3000 for each of us that we have to pay. So we're trying to like get out of that because like well we weren't living there and there was no point of us living there. And I know lot of people um are going through that like trying to get forgiveness for their rent. So, um yeah we're gonna see what's gonna happen with that.

Moderator 58:13

Okay.

40 58:16

Yeah, for me, like uh others who said before, overall my finances have been okay been okay, but for my family I know it's been a little harder, not being able to work as much. Um but one benefit for me was that I I since I was living in a dorm, we did get refunds for the months that we weren't living there. So I've been able to use that money for my summer classes and I'm going to use the rest of it cuz I signed a lease also for next year. We signed at our school we sign them very early. So I'm already in a lease for next year, but I can use some of the money that I got back to just apply it there so it's like a little bit less stress about how I'm going to pay for that. So yeah.

Moderator 58:56

Okay. Okay.

62 58:59

Um I, so leaving um a lot of the like cuz as a freshman I didn't have a car on campus so like Ubering places, um to put storage away, buying a storage unit, like all of that put a little bit of a strain on my personal um finances. But when I got home, I talked to my parents. Um they were able to like help me out. They gave money back to me for everything that I um spent. I also got a refund check which I've put towards my summer classes as well. Um, I haven't really discussed with my parents if they have gone through any financial strain. I haven't seen any signs of it. But um we were fortunate enough not to have been affected very much by that.

Moderator 59:58

Okay. Sorry if you guys just heard my dog also. I'll try and keep her quiet. Um, so now let's talk about what your professors did and did not do during the move to online learning. What are some examples of strategies, tools, or technologies that your professors used that you found to be effective, and or very ineffective during online learning?

57 1:00:23

So, the majority of my professors used zoom, which I'm pretty sure everyone's familiar with. We're on zoom right now. Um and I'm not the biggest fan of like the interface. But one of my professors he actually used this thing called Boing. I don't know if anyone knows what that is, but it it it's similar to zoom and I think it works a lot better um just because you can do more things like the search bar, not the search bar, like there's a chat bar all on the side where everyone can see what's going on. I just feel like I've had a lot of less technical difficulties with Boing than I did with zoom. Um another thing my professors did were virtual office hours. Um some people did it through Skype and some people did it through, like a zoom, or a Boing, but I think those were the main things that my professors did.

Moderator 1:01:16

Okay. Oh 33 go ahead.

33 1:01:18

Oh yeah. Um, yeah so my professors they also did like zoom office hours, um which I attended some of them. They were pretty cool. And then, uh like the TAs for my computer science class they had some. You have to sign up beforehand and then you get to speak them for like 15 minutes. I do wish like time, the time that given was a little bit longer cuz in person it would be probably 30 minutes but they cut it down. Um so yeah, zoom office hours.

Moderator 1:01:49

And 40.

40 1:01:50

Yeah, mine was pretty similar. Most classes offered zoom office hours. I didn't really use them, but some teachers um some professors also were okay if you emailed them different questions you had so I emailed several peo- professors several times and they were very like good about responding quickly and keeping that line of communication open, which I liked.

Moderator 1:02:14

Okay.

62 1:02:16

Uh my professors they all um they were they responded to emails a lot quicker than they did during the year. Uh they would tell us the times that they were going to be looking at their email to make sure like uh they would get to us, effectively, and a lot of my teachers chose to do their own review sessions on top of the review sessions from the resource centers. So like if it was a class where they were making their own exams that was extremely helpful. Rather than getting just like collective information, I got specific information catered to what they were looking for.

Moderator 1:02:53

Okay. And what are some things that professors did during online learning that made you feel like they cared or did not care about their students?

57 1:03:01

Um they cared by like having more office hours, being more respondant to emails, but the non care wise was just when my professor blatantly made tests extremely harder. Like I had never made lower than an 84 on a test. I had an A in the first bio and A in this bio. There's no thinking that I would not get an A in the class and then I get a 66 on the first test that's online. Just blatantly making it harder um because he doesn't want students to cheat, but it was pulling students with actually learning the information, and just trying to figure out how to get that grade that they wanted. And that was like the non care thing that I hope from the results that happened with that class, he realizes and reevaluates his methods.

33 1:03:56

Um, so for me, my, my stat professor he was actually like really accommodating to things and he made like a grading system like two different ones. So, um, like two different weightings for exams. So if you did bad on one weighting then you'll get the other one which is better. So, um to try to really help people, make sure they pass. And then for my other uh teacher, he made like our exams, you can take them any time of the day, just when you start, you can't like pause it so yeah like he didn't say you can take it only at this time so we could take it throughout the whole day which is really nice too.

40 1:04:37

For me, um, overall, I felt like this for my classes they really listened to what the students had to say about like different issues. So a lot of my classes actually didn't proctor exams or anything like that, especially after students talked about privacy concerns, and my math class was similar to 33's. They weighted, they had two different grading scales and if whichever one made your grade higher that's what they used when they calculated your final grade. For my stats class though, I was kind of irritated because the other class and it's the same class but two different professors, they had 12 hours to take an exam and it was a take home exam, and we had a 40 minute proctored exam so of course our average was way worse and our overall average for all the exams was like a 50%, but the teacher just didn't care, and it was just annoying.

62 1:05:32

Um, so I had so one um thing that my teacher did is my chemistry teacher would go through and we would be given a grade and she would go through and hand grade everything to make sure that um, cuz like you know some people type something in wrong. Like it'll be off by point something, uh just calculation errors. She would go through and give us a range, and then change all of our grades if they fit the range so you would do either, most likely you would do better than uh the grade you were originally given. And my calculus teacher he, um, made the I don't really know how to explain it. He had, he did all these percentages and basically was like, "you need this percent of the points left to get this grade" and then he would give us take home quizzes. We got a take home exam. The final he gave us an hour longer than we were originally supposed to get. And uh I would agree with 40 for how things were bad. Um just certain teachers saw that people were doing a lot worse and kind of just didn't care. Um, I had mentioned earlier, just the tests average had gone down a lot and the uh department just didn't really do anything about it. They didn't listen to people either when they expressed their um concerns and that was disheartening for me.

Moderator 1:07:08

Okay. Um, now let's talk about how your university responded to the move to online learning. What are some things that your university did, for example, pass fail options, town halls, or remote counseling to help students be successful during online learning?

57 1:07:26

I think overall my university did a pretty good job. Um everything was very sudden because first we were told okay we're online till further notice and I was still there for tennis. The next week they said, "you're online for the rest of the semester". I was still there for tennis and then finally they canceled sports. Um but that had to do with like the whole body of NCAA and stuff like that itself. Um they also did offer like you could go virtual. Um we had like health um counselors and like for um counseling and stuff like that you could still do those. You could schedule appointments and do that. They did a lot of town hall meetings, um discussing upcoming plans for the fall. And that wasn't just something that was like thrown on us. They, we got to vote on how we wanted some of our classes, if we wanted them asynchronous or synchronous or online or in class. Um they also did, what did they do um. They kept on offering all of our resource centers for all the math classes, um physics, organic chemistry, general chemistry. None of the resources that we had in school um were not offered over the course of being online. They still offered them. They even did like zoom parties and stuff like that, just to try and keep the morale up between students.

33 1:08:55

Um so with my school, um a lot of people didn't really like the response to everything. So back when everywhere was shutting down in March. Um so Redacted is like aligned with all the other Redacted schools like Redacted, um some other schools. They'll those schools shut down but ours didn't, and people believe they didn't shut down because it was um midterm season and they didn't want, you know. They wanted everyone to take their, their exams in person. So um there's a lot of pushback on that. Um but um now we just get a lot of emails from them. A lot of vague emails, and um they have town halls, too. So, but yeah, it's just, I don't really like their response to things.

40 1:09:49

That's funny. That's pretty similar for my school too. We get a lot of a lot of emails, but they don't really say much about anything, which I kind of understand because these are uncertain times. So, I mean, it's difficult to figure out, but I know a lot of people at my school also were just irritated by the fact that they didn't really know what was going to happen, what was going on. We didn't have like a clear response from our school. But one thing they did do that I talked about earlier is they gave us the option to take classes credit no credit and then also, they changed the date, like they made the date later that you could requested um credit no credit for your class just so you had more time to decide like if you thought you would pass the class and if that was a good option for you. So I did appreciate that. And then my specific department also just sent out emails and things to ask us about what was working and what didn't work for us in regards to different classes, so that they can change things around for next semester. So, I mean, even though this semester wasn't the greatest, I do like that they like cared enough to want to change it for next semester.

62 1:10:56

Um, we got the pass or fail option as well. Um, I personally didn't get a chance to use it because um after our pre med office went online well all of our like offices and things like that went online, but I specifically contacted the pre med office and they advised us not to choose pass fail just because, um, they didn't know how medical schools were going to react. They were like cuz some medical schools don't accept pass fail. So they were like honestly just because we don't know what's going to happen we don't know how people are going to respond. They advised that we didn't do it. Which I mean now considering um we'll be online in the fall and possibly the spring as well, it'll be a little bit harder for med schools not to accept pass fail, but that was just um something that happened. I didn't like how they handled us having to leave campus um because uh I go to Redacted University in Redacted, and they-- when Redacted shut down is when we were supposed to shut down. And Redacted shut down on a Friday, but they waited for a Monday evening to tell us that we were shutting down. And so I had to scramble, find a flight that wouldn't conflict with classes, then completely move out of my dorm which I wasn't ready for, and then they just a lot of they gave us a lot of information. They were like okay, this needs to happen, this needs to happen, this needs to happen, but then like deadlines and things were later. Like they decided after they told us that we need to move that they were going to give us some days off where people could move. They told us that like two days later. And I was like, well, my flight's already booked now and I've already missed half my classes for today trying to pack up my room. Like this I could have used this information yesterday. And they they've done better for the fall. Um we also got an email asking if we want remote uh remote classes, um in person, all of that stuff. And I like that. Like I chose to get a mixture of both. And I'm just, I'm not sure how that'll play out so that's my only concern because like there are one or two orgo classes that are in person, and then the rest of them are online. So it's like I don't know if the people who have class in person will do better than the people online just because they have that in person experience. I'm just not sure what the difference will be like.

Moderator 1:13:34

Okay. Um thank you all for participating today. We are out of time, unfortunately. We did miss a couple questions so we'll send them in our follow up email and we'll ask you to send written responses to them, along with the names that you're using here today. Um, but yeah, we'll go ahead and wrap up with that. Thank you all for participating. Have a great rest of your day.

Emailed responses High SES Black Women

1. What do you wish your university did to better help students be successful during online learning?

2. In general, are there any ways that your ability to be successful in school was affected by the pandemic that we have not discussed?

Survey ID 33

1. I wished that my school provided a clear structure for their plan when it came to how the professors where to conduct classes. Each professor did their own thing at their own time and I definitely got overwhelmed because of that.

2. I guess one thing that was not really touched up on was the mental impact. When the quarantine started in March, I had very little motivation to be productive because since I was in the house the entire day and did the same routine, I got caught up in not caring about anything anymore. I grew out of that as the months went along though

Survey ID 40

1. Although my school extended the credit/no credit request deadline, I wish that they would have moved it to after finals to decrease some of the stress on students. I know several seniors who wanted to utilize the option, but felt like they were gambling with their graduation date (if they did end up receiving no credit). Additionally, I know one girl mentioned her school provided counseling resources and I wish my school did that. Even during the regular school year, without a pandemic involved, it's extremely difficult to get counseling help. This is partly due to the large student population though.

2. I mentioned feeling like I had a decrease in learning quality this spring semester because of the pandemic. I'm worried that all the material that I didn't properly learn this semester will reflect in my future classes because my classes build on each other. I'm going to somehow have to find time to properly relearn the new topics I learned to make sure my foundation is strong enough for the rest of my undergraduate classes.

Survey ID 57

1. I think that it should be necessary for all professors to record their lecture because there are more likely now to be circumstances where students can miss class

2. Overall, I don't think there are any other factors that have inhibited my overall ability to be successful in school

Survey ID 62

1. I think that for the most part my university did well to make sure that people were successful. I only really had issues with one specific department, and I just wish they would have been more understanding about the drop in test averages and how that was mostly due to moving off campus. I also wish they would have told the students that they were going to make the test questions that much harder and allowed us to adjust studying methods accordingly.

2. I'm not sure if we explicitly discussed this, but there is a different headspace being at home versus being on campus which made it a lot harder for me personally to focus in class. I just feel a lot less productive at home and think that being either in a dorm or in the library are ideal environments for studying/doing homework rather than being in my bedroom at home.

**Low SES Black Men**

Moderator 6:23

Hi. Okay, so I think we might just be looking at a turnout smaller than what we expected. So if that's the case, then why don't we just go ahead and get started. Welcome to the focus group My name is Moderator I'm a research coordinator from Redacted, Moderator's gonna be our note taker today. We're going to be asking a number of open ended questions there are no wrong answers, people well. Well, you're not gonna have different points of view but feel free to share your points of view. Keep in mind that we're just as interested in positive comments as we are negative comments, we're not really worried about time today, so feel free to just speak, whatever you need to speak so we can get the full breadth of experiences and opinions. Yes, it's difficult for us to take notes on everything that said in the group. So we'll also be recording the session so that we can transcribe it later, but we won't associate your name with anything that you say here today. You have the right to refuse to answer any question or withdraw from the group at any time. Everything you say here is confidential and private. Are there any questions before we get started? Okay. So, why don't you start by just telling us the way your college just finished in one word to describe what life has been like for you during the pandemic.

56 7:47

Oh, I live in REDACTED, REDACTED State so I go to community college, I'm studying civil engineering, and I'm transferring to university this fall. Since the pandemic classes are all been online so it's been kind of nice and kind of hard at the same time because, you know, online is like. You can do pretty much at your own pace and you can do it anytime but the downside, was that I had a lot of tough classes, and it was really hard got like, you know, understanding them when they're online.

Moderator 8:34

Okay, so the covid 19 pandemic has presented big challenges for undergraduate students, as you know universities across the nation have closed caption online available time for center faculty to prepare. Many students have encountered challenges related to the closing of campuses relocation and the movement online instruction. So in the center you want to learn how the closing campus and the pandemic in general has affected you. We'll start by discussing where you went when your campus shut down. Tell me. Yeah. So can you tell me about the space in your home that you moved to what kind of space, did you have to do schoolwork, was it private or shared, so on and so forth.

56 9:14

Oh, so I live with my two brothers so each one of us have his own room so it wasn't really hard for me to find the space to study. But, you know, I had to maybe get a better desk and a better chair because I had to. So, pretty much, living at home, you know, because even the library's all been closed, you can really go nowhere but you know just study at home.

Moderator 9:44

Okay.

56 9:44

Did I answer the question?

Moderator 9:46

Yes you did. So what kind of challenges did you encounter attending class in your room?

56 9:55

Let's say, like, a lot of times maybe I had to email the instructor, you know because things maybe weren't clear or, you know, maybe we had to use like a software and something's, you know, didn't work. So, you know, I had to contact the supervisor, the professor more than often, you know, to kind of like, see how can I resolve all these issues. You know, also it is really harder to study for, especially like math classes because I'm studying civil engineering, you know, math can be, you know, especially upper classes. It's kind of really hard, you know, to study for them and to practice when you're doing all the things at home.

Moderator 10:49

Okay, and what challenges did you have by working in your room that you wouldn't have had if campus was still open?

56 10:57

I'll say maybe the internet. Sometimes it will, you know, go down whatever. Even my laptop I had to change it because you know there, my old laptop, it wasn't really that strong to kind of like hold everything that I'm putting on it and you know just kind of like do everything that I wanted to do so. I had to buy a new laptop, you know, in order to do all this stuff for school.

Moderator 11:29

Okay. And what are some things that you or other people in the House did that made it easier for you to attend classes and do your schoolwork?

56 11:42

Pretty much all of us in here are students, so there wasn't really anything that we did new but we kind of like maybe, Just like, you know, like I said earlier, like bought some stuff you know and like furniture stuff in, you know, we also, just became more understanding of each other's like times, you know, maybe the other person is on a meeting or just having a class, you know, so you got to maybe be a little quieter, or something like that.

Moderator 12:15

Okay, so let's talk about the other responsibilities she had after campus closed and how this differs from responsibilities you had before campus closed. Describe your caregiving responsibilities, including child care elder care or taking care of ill loved ones. And how are they alike, or different from your responsibilities before the shut down?

56 12:39

But maybe was perfect for this survey but pretty much I have nobody that I had to take care of. So, in terms of responsibility. I'll say that, you know, pretty much, I really didn't have, like, my job, you know, I had too much to talk to my supervisor about my hours and, you know, you know, because a lot of classes, kind of like switch at times you know just everything got mixed up together so I go back then. I can maybe work a little later, you know, and stuff like that but in terms of child care stuff like that. You know I don't have any.

Moderator 13:25

Okay. Um, what kind of household responsibilities, did you have like chores or cooking or taking care of pets and how are they alike or different from before school closed?

56 13:39

I'll say more because now I'm, you know, staying at home or so you know everybody's home. So we got to cook and eat and clean, you know cuz you just saying how more so obviously you're gonna have to do more stuff. In terms of like household stuff, you know, I still have to pay my rent, you know, so I have to, you know, pay all the bills. So it wasn't super hard but it was kind of hard because my job like they kind of like gave us less hours, you know when COVID started so you know my hours were reduced and, you know, I had to figure out how to how I'm gonna pay for it.

Moderator 14:28

Okay, so you said you're paying rent. Were you paying rent before and you're still paying rent or just, What's going on there?

56 14:38

Yes, I was still paying rent but, like, in terms of my job, my hours were reduced. So, you know, like my money was less so. is less now so you know I had to figure it out some ways to you know make more money so I can pay the rent.

Moderator 14:56

Okay, I'll come back to financial things later. So, okay, I'll ask about that, again, in a sec. To what extent were expected to take on more household work child care or family caregiving responsibilities and other people in your home? So like in comparison to your brother or anyone else who you live with; Do you feel like the work was divided up evenly?

56 15:20

Yeah, for the most part. I'll say one of my brothers work more than us you know so much he wasn't at home so the time so maybe me and my other brother had to do most of like the chores and stuff. So, yeah, for the most part it was like evenly but you know a couple of things.

Moderator 15:45

Is it just you and your brothers in the household?

56 15:49

Yes

Moderator 15:51

Okay, so let's talk about how your academics were impacted by the shutdown. I'm going to ask about a variety of things but if it wasn't something that affected you you can just say no impact. In what ways if any has to shut down impacted your academic performance grades and your understanding of the subject matter of your courses.

56 16:14

Understanding the you know the subject, you know, I feel like with online, you know for some courses. You know, I'm not really. I don't really like understand it as much as, you know, your standard when I'm in class. You know less effective when it comes to understanding the subjects. Yeah.

Moderator 16:39

Okay. And what about your grades were they better or worse than they would have been without the shut down?

56 16:46

I'll say the same you know cuz I cannot figure some way to do this stuff without actually understanding it. So grades were an actual big problem for me was, you know, I'm really not understanding this subject, as I should be, you know.

Moderator 17:06

Okay, okay. So in what ways if any has a shutdown impacted your long term career goals?

56 17:16

I'll say. So, this fall. We were supposed to have a career fair and, you know they moved it to virtual instead of in person, so I'll say that that kind of affected me because you know I was preparing to you know go in person, you know and maybe get a internship, you know, and which will help me in my future career.

Moderator 17:46

Okay, and is the goal that you have now different from the one you have before the shutdown or have you always wanted to be that?

56 17:57

I mean it's always been like that, it wasn't different.

Moderator 18:01

Okay. In what ways if any has shut down impacted the availability of other learning opportunities such as internships?

56 18:14

No impact as of right now.

Moderator 18:16

Okay, did new learning and or career development opportunities emerge during the shutdown?

56 18:24

Like new learning opportunities, you said?

Moderator 18:28

Yeah, any like learning and professional development opportunities.

56 18:38

No, no, no, no comment honestly.

Moderator 18:45

In what ways if any has the shutdown impacted your confidence in your ability to successfully complete your bachelor's degree at all or in a timely fashion.

56 18:55

I will say definitely impacted my, you know, confidence that I'm going to finish on time, you know, because now you know, everything is pretty much unknown. So, always like thinking, you know, will I be able to take classes online, you know, when will this class be available, you know next semester, you know, because what if all classes change online and you know the professor that teaches you a specific class. Now decided, you know he or she don't want to do it because you know, maybe they want to go in person, you know, that kind of thing. So, definitely.

Moderator 19:45

Okay, so now in what ways if any did the campus shutdown impact your finances?

56 19:52

Oh, like I said earlier, mostly. I mean, it wasn't the campus itself but it was more like my job, you know, campus, but it's kind of related because some of my classes changed their times, you know, and maybe I used to work at that time. Now I had to go back to my job and tell them, you know, I need to kind of change my stuff, mostly for upping money, you know, I can.

Moderator 20:25

Okay, now let's talk about where your professors did and did not do during the move to online learning. What are some examples of strategies or tools or technologies that your professors used that you found to be effective. And similarly, what are some things they use that you found to be effective.

56 20:46

One of the things that couple professors actually did which I found very effective is to kind of like pre record all the lectures instead of, you know, have a live zoom meeting, you know, because sometimes you know, during that time I had to do something, go to work or just do any thing so it was very convenient to have all these videos, you know, whenever I needed them in terms of inneffective. Maybe I had couple professors who weren't really communicated as they should have been, you know, like you had to reach out all the time and, you know, ask about things that wasn't clear, maybe, you know.

Moderator 21:38

Okay. And what are some things professors did during online learning that make you feel like they cared or did not care about their students.

56 21:49

I'll say definitely the clarity of the course content, you know that you know the the more clear the professor was the more effective or, the more they cared. They seem to be, you know, if that makes sense. You know, if, if they made everything clear, you know, to me, though, you know, it's like, oh, they, you know, they should. But if he had to actually ask about everything and you know, everything was unclear. So, to me, they'll like Oh, they don't really care. So, I hope that makes sense.

Moderator 22:27

Yeah, I think, I think that makes sense. So let's talk about how your University responding to the move to online learning, what are some things that your university did to help students be successful during online learning. So for example, that might be pass/fail options, town halls, remote counseling, anything like that?

56 22:45

I don't really have a ....

Moderator 22:52

Um What do you wish your university did to better help students be successful during online learning.

56 23:03

Like I said earlier, like one of the things that I wish all classes did, was to pre-record lectures, you know, instead of, you know, having this zoom meeting like a live meetings. You know, like with everything changes. It can be hard first to another thing. Maybe I'll say help more with technology resources, you know, kind of like, if some students have laptops, you know, that kind of thing.

Moderator 23:37

Okay, so providing technology. Okay. And in general, are there any ways that your ability to be successful in school was affected by the pandemic that we've not yet discussed.

56 23:53

Oh not really.

Moderator 23:55

Okay. Um, in that case, that is all the questions. Thank you for participating in this.

Participant 64 – LS Black men email response

First, I’d like you to tell me about the space in the home you moved to. What space did have to do your school work? Was this space private or shared? If shared, who you shared it with.

**When I moved home, my parents gave me my sister’s room and it became my study room and my own room. It was for the most part private, I spent most of my time doing work in my room.**

What challenges did you encounter attending classes or doing your schoolwork in this space? Could you tell me about why this was challenging for you?

**It was more difficult paying attention certainly. Since you could easily turn off your camera, occasionally it was easier to fall asleep in class, which I already did when I was at school. Additionally since most classes backed off of work and tests, it was easier to get by doing assignments without truly knowing the material.**

What are things that you, or other people in your house, did that made it easier for you to attend classes and do your school work?

**Certainly, allowing me to be in my room most of the day without any distractions.**

If you live with siblings, to what extent were there differences in how much parents supported each of you in your ability to attend classes and complete schoolwork? For example, were some kids in the house given more time, space, and consideration to do school by parents than others? Could you tell me more about why you think it was alike or different?

**I lived with my sister and my brother. My brother has already graduated, so he didn’t have online classes. My sister however did. My parents didn’t really provide any support, I attended a boarding school for 4 years before college, and therefore my parents were confident in my ability to maintain my studies. However, they routinely had to remind my sister to do work and maintain her studies/focus.**

**In our next set of questions, let’s talk about what other responsibilities you had *after* your campus closed and how these differed from responsibilities you had *before* campus closed.**

Let’s start with care-taking responsibilities. Could you please describe your care-taking responsibilities? This can include childcare, eldercare, or caring for ill loved ones. Please describe how your care-taking responsibilities after the shut-down were alike or different from your responsibilities before the shutdown.

**I didn’t have any care-taking responsibilities before or after quarantine.**

Now let’s talk about house-hold responsibilities and how they changed before and after the shut-down. Please describe your other household responsibilities such as chores, cooking, caring for pets during this time and how they were alike or different from before school closed.

**Before quarantine, I was at college, and therefore I didn’t really have house-hold responsibilities. After quarantine, occasionally I’ll have to buy groceries and throw out the trash, but my parents didn’t really impose any work on me other than laundry, which I tend to do a lot because I run a lot.**

After the shut-down, to what extent were you expected to take on more household work, childcare, and/or family caregiving responsibilities than other people in your home? For example, parents may expect more of one sibling, or partners and/or roommates might not contribute equally.

**My parents expected more from my brother because he was older and also didn’t have to attend classes. My sister was also probably expected more, just because our family is traditionally Ghanaian, and they believe that women should learn to do household work.**

**Thank you all for sharing. Now let’s talk about how your academics were impacted by the shutdown. I will ask you how the shutdown has impacted a variety of things. If you don’t think it impacted something I ask you about just say “no impact.”**

In what ways if any has the shutdown impacted your academic performance (like your grades) and your understanding of the subject matter of your courses?

**My grades boosted when I converted to online (4.0) because teachers were more accommodating due to coronavirus in addition to the capacity to remove one class as P/F. However, I came out at the end of the semester realizing that I had not internalized the material.**

In what ways, if any, has the shutdown impacted your career goals? (follow-up: could you tell me a little bit more about why they have changed?)

**I don’t think that the shutdown has necessarily impacted my career goals, but because most summer programs have moved remote, it allowed me to do more programs in my field and have more free time to do math/ read math books. As a result, I became more interested in math.**

In what ways if any has the shutdown impacted the availability of other learning opportunities such as internships?

**The shutdown has actually widened the availability of learning opportunities because many of them are moving toward a remote format which removes requirement to travel and ability to do multiple at once. Many also pushed their deadline closer to the end of summer.**

Did new learning and/or career development opportunities emerge during the shutdown and if so, what were they?

**Many tutoring opportunities arose which is good because I’m also interested in education.**

In what ways if any has the shutdown impacted your confidence in your ability to successfully complete your bachelor’s degree at all or in a timely fashion?

**It has made me more confident because when I’m online and at home, I don’t have to attend extracurriculars/ travel between classes. As a result, with this extra time I’m able to take a larger course load.**

In what ways if any did the campus shut down impact your finances?

**Since as a Redacted college is fully funded, I actually made much more money because of quarantine. I don’t end up paying for my education, but since the semester ended prematurely and converted online, the school refunded some of our meal points and tuition, as a result, I made a net profit.**

**In our next set of questions, let’s talk about what your professors did and did not do during the move to online learning.**

What are some examples of strategies, tools, or technologies that your professors used that you found to be very effective (in that they made it easier to learn) during online learning?

**PROBLEM SOLVING TOGETHER IN BREAKOUT ROOMS ARE THE ABSOLUTE BEST. Professors often pre-recorded lectures which allowed us to watch them at our convenience.**

What are some examples of strategies, tools, or technologies that your professors used that you found to be ineffective (in that they did not help you learn) during online learning?

**I don’t think that my professors did anything that I considered ineffective.**

What are some things your professors did during online learning that made you feel like they cared about their students?

**My professors were all very accommodating and understanding about the situation. I believe that they were as frustrated as we were and as a result we bonded over the situation. I routinely zoom with my class and my professors over the summer. They also routinely offer encouraging words to us.**

What are some things your professors did during online learning that made you feel like they didn’t care about their students?

**Nothing really.**

**In our final set of questions, we would like to know more about your university. Let’s talk about how your university responded to the move to online learning.**

What are some things that your university did (e.g., pass/fail options, townhalls, remote counseling) to help students be successful during online learning? Mention any resources the university provided or actions they took to help students be successful or feel supported.

**Our school offered pass/fail options for all classes which largely reduced the stress for all students.**

What do you wish your university did to better help students be successful during online learning?

**I believe that my university handled things relatively well.**

In general, are there any ways that your ability to be successful in school was affected by the pandemic that we have not discussed?

**Not really, I believe that the pandemic will make more successful in the future for school just because I believe I can focus more and have less non-school work to do.**

**That is the end of my questions. Thank you for participating in this focus group.**

**High SES Black Men**

Moderator 7:04

Alright, looks like everyone is here so I'm gonna go ahead and get started. Um welcome to the focus group. My name's Moderator. I'm a research coordinator from Redacted. Um and I'm going to be your coordinator tod- uh coordinator today. Note-taker is going to be our note taker. So Note-taker, if you want to say hi.

Hey everyone, thanks for coming.

So today we're going to be asking a number of open ended questions. There are no wrong answers. So feel free to say whatever your point of view is. Keep in mind that we're just as interested in negative comments as positive comments. So feel free to say either or. We want everyone to feel comfortable participating and encourage everyone to talk. Because we have to keep this to an hour I might have to cut you off so I apologize for that in advance. We want to get the full breadth of experiences and opinions but some of you may have very similar experiences and opinions. If your answer is the same as someone as someone else's you can just say my answer is the same as theirs. It is difficult for us to take notes on everything that is said in the group. So, we're also going to be recording this session so that we can transcribe it later, but we won't associate your name with anything you say in the focus group. You have the right to refuse to answer any question or withdraw from the group at any time. We understand that this information is very private and confidential so we'll ask that we respect everyone's confidentiality and not repeat things said within the group to anyone outside of the group. Are there any questions, thus far? Okay. So why don't we just get started. Why don't we all go around and say your name, along with what year of college you just finished, and one word to describe what life has been like during the pandemic.

Um sorry, 44, do you want to start?

44 8:55

Sure. And it's 44.

Moderator 8:58

44. Okay.

44 9:00

So yeah, my name's 44. Uh I go to REDACTED University and life has been busy.

Moderator 9:10

23, what about you?.

23 9:12

Hi, my name is 23 and I go to REDACTED, and I would describe it as unconventional.

Moderator 9:21

Okay. M, do you want to take a go?

35 9:28

Hi I'm 35. I go to REDACTED University. Life has been complex.

Moderator 9:38

Okay. Um I don't want to butcher any more names (laughs) to someone who hasn't gone, want to go and tell me how to pronounce your name? Um 42?

42 10:03

Hi. Um actually, my name is 42.

Moderator 10:05

Oh okay.

42 10:07

...use the computer, changed the name, long story, okay.

Moderator 10:09

Okay.

42 10:09

But um, uh you know, life has been uh different, but you know, just been adapting change, you know, it's been it's just been hard to get used to, but, you know, as time goes on, you get used to the circumstances, at at play. So it's been it's been okay I guess for the most part.

Moderator 10:28

Okay and um 18.

18 10:34

Uh, hello. I go to REDACTED College. Um, so life has been, has been kind of difficult. Like staying at home for so long, kind of going crazy. Yeah.

Moderator 10:49

Did I say your name right?

18 10:50

Yes, you did.

Moderator 10:51

Okay. Cool. All right, so the COVID-19 pandemic has presented big challenges for undergraduate students. As you know universities across the nation have closed moving all institutions online with very little time for students or faculty to prepare. Many students have encountered challenges related to the closing of campuses, relocation and the movement to online instruction. In this study, we want to learn how the closing of campus and the pandemic in general has affected you. And we want your opinions about the good and bad on how things went as well as what your professors and universities did that was helpful and unhelpful, and what you wish they would have done. So I'll start by discussing where you went when campus shut down. Tell me about the space in your home you moved to. What space did you have to do your schoolwork there?Comment on whether the space was private or shared, and if it was shared, who did you share it with? And we can go in the same order so 44 if you want to start.

44 11:52

Okay, uh so once you know school closed down I went back home. Um I do most of my work in my room. It's fairly private, but uh we have really thin walls so I can kind of hear everything going on in the house. But yeah. Was that all the questions?

Moderator 12:08

Yeah so was your room private, like just your room?

44 12:11

Yeah.

Moderator 12:12

Okay, and 23.

23 12:18

So after we went back well after everything closed down in our college I went back home. And I first I was in my dining room but then my mom, she's a physician and she had to do her patients, well she had to see patients through zoom in their dining room so then I moved to my bedroom. And it was private, both were private.

Moderator 12:40

Okay.

35 12:43

So for me. Yeah, it's been private, I have my own room. I do work from my room predominantly. Um I live with my mom um my mom up here, and she takes the dining room I take my bedroom. We don't really get in each other's way like that. I can hear sometimes when she's on a meeting, but otherwise it's been alright.

Moderator 13:06

Okay. And is your bedroom like private or do you share it.

35 13:14

Yeah it's private.

Moderator 13:16

Ok and 18.

18 13:20

Uh so I, I it didn't really affect me cuz I commute to school so I just kind of just stayed at home.

Moderator 13:32

Oh so you were already where you were.

18 13:34

Yeah, it didn't really affect me that much.

Moderator 13:36

Okay, so where do you usually do work?

18 13:39

Usually, I have um a space, a computer space I just usually do work in.

Moderator 13:47

And is that just your space or do other people use it too?

18 13:50

No just my space.

Moderator 13:51

Okay, cool. All right. What challenges did you all encounter attending classes or doing school work in this space? 44 if you could? Yeah.

44 14:07

Uh let's see, I found it kind of hard to wake up on time, and like listen to lectures. It's my bed is like five feet away from me. So, um that was a difficulty, as well as like I have younger siblings who are like, what like nine and seven. So they're kind of loud and they're always running around the house because they have nothing better to do so. It can be a bit distracting at times. But yeah.

Moderator 14:34

Okay.

23 14:40

For me, waking up was also a struggle at first. And I also think that um, time management was harder in my room with rather than this, rather than school there's so many places you could go to study, do work, just being like, stuck in a room doing your work is just kind of difficult, compared to how I usually do my work in school.

Moderator 15:08

Okay.

35 15:08

Uh I find that it was quite bittersweet, um in a sense that I felt more unmotivated. Uh at times you know I would slack a lot, uh put off like work I usually do like on the spot. But at the same time I found I was performing better because when I did decide to work, I kinda was more productive. And you know, I wasn't motivated to do more work like outside of school like in classes where I struggled with the professor, I found myself um I had more time to go on YouTube and Khan Academy and teach myself certain things I was struggling with during the semester because I didn't have certain distractions like um my organizations, you know, like on campus organizations all that stuff, and all the classes were synchronous now so I could work whenever I wanted to. Uh I guess I perform better in that regard.

Moderator 16:15

Okay uh 42.

42 16:20

Yeah. Sorry is there an echo? Can you hear me?

Moderator 16:25

I can hear you.

42 16:26

Okay. Um, so the main challenges I face was mostly that some everybody else were coming home, I had to deal with like my little siblings, you know, and like really young like I'm talking like, six, eight and I have a little sister who's like um two. So I was they always came in my room whenever I had a lecture or when I was busy with homework and they would often ask me for help and it's hard to say no. So I took I would have to go and help them. Um also, distractions, there were more distractions here at my house. Um because you know I got I got all these things to do and I got a I got a game right next to me. My bed is like right behind me. It's hard to it's really it was very hard to focus and do work, you know. But yeah that was it.

Moderator 17:08

Okay, we're actually going to touch on some of the parts about siblings or whatnot later on. So hold that thought and we're gonna get into that later. Um 18.

18 17:20

Yeah, so most of my um classes were pre recorded. Um they had pre-recorded lectures so, like waking up wasn't really, I just woke up when I woke up whenever I wanted. And I just listen to them. Yeah. And also like everyone else, I have little siblings, so that kind of made it a little harder. So you just like making noise. It's just harder to, like, to then just pay attention to lectures. Yeah.

Moderator 17:47

Okay, so I'm kind of getting that it was hard to remain motivated when there are so many things of comfort surrounding everyone. What are uh things that you or other people in the house did that made it easier for you to attend classes or do your schoolwork?

44 18:20

Can you repeat the question?

Moderator 18:21

Yeah. What are things that you or other people in the house did that made it easier for you to attend classes or do your schoolwork?

44 18:30

Okay um one thing that I did that made it easier for me to attend classes was like, rather than like like waking up and then like studying all day or just working all day, I would like spend, I would like to start a timer. And then I would pause that timer every time I like did something else. So I would like tell myself I was gonna dedicate like three solid hours to my work. And then every time I like goofed off or went on Instagram or something like that I would pause the timer. That way I can make sure I was actually spending that three hours, and that kind of kept me accountable. Uh because rather than just, you know, wasting my entire day spending, like doing something else I was actually making sure that I was doing my work. I guess something that my family did was, they tried their best to keep the noise down, which, which definitely helped a little bit so when I was like, attending like lectures online or something or like maybe like a, like a group call for like help or something, they were kind of quiet during that so that was definitely helpful.

Moderator 19:26

Okay, okay.

23 19:31

For me, I took all pretty much all 8 and 9am's so really early classes, and my parents would wake up like seven, eight to do their work or go to their work, and usually hearing them waking waking up and like hearing them eating food, cooking food, getting ready for work that would motivate me more. Because in college um it's really on, I'm accountable for my own stuff but it was just, it was a little bit motivating to sort of hear other people being productive so I guess that kind of boosted me, pushed me to do the same, to be honest.

35 20:10

Uh for me, I guess, like I mentioned before motivation was the big issue for me so I found myself having to force myself to do work. I wasn't as productive as I was on campus. Campus I could probably work, maybe one or two hours um without too many interruptions. Home I found myself, you know, I didn't want to do that, you know. No matter what I did, I couldn't work the same way I did at campus, because an absence of environment like the library. So what I did was I would do what I could within a certain timeframe, you know. I didn't say I have to do this with 30 minutes. If I did 10 minutes and I got bored I'll go do something else like maybe answer and come back to it. Uh if I did the next round 30 minutes, and then you know match that and then 30 minutes of Instagram or whatever. Um and that's how I did it. I would do create the same amount of time I spent studying, I would then do uh playing or doing something I enjoy, and that's how I got through it.

Moderator 21:20

Okay so maintaining that work life balance. Good.

35 21:23

Right.

Moderator 21:25

All right.

18 21:31

Okay, so I set um aside Tuesdays and Fridays just to listen to lectures. And then I had a live class that we had to attend that was on Wednesday. So that was the only time, and it was early in the morning, it was like eight o'clock. So that was the only time that like I was kind of motivated to get up and actually listen to the to it because, yeah, cuz all the other days, I was like, I had nothing to do. So I'll just watch lectures and then do something else. So that was really, really it.

Moderator 22:05

Okay. Time management skills. Nice. Um so. If you.

42 22:18

Are you doing?

Moderator 22:19

I'm sorry

42 22:19

I wanted to, can I say can I uh answer your question?

Moderator 22:24

Yeah.

42 22:25

Yeah, yeah. So, um for me, um I came to realization that I couldn't um learn or do lectures at specified times like back in college, you know like, I would have to. I'd have a lot of time to just go to lecture, whenever the class lecture scheduled for, but at home you know my my schedule varies from time to time, so it was best that I studied when I was when I had time, when I could find free space, free space in my schedule, because it varies depending on what's happening around me. So, yeah, so I I utilize other resources like YouTube, Khan Academy, recorded pre recorded lectures, instead to study or to learn when I had the time. So that was something I had to realize. Cuz before I used to wake up I don't know really early like seven, eight to to do work but then I realized that I I couldn't deal with the stress anymore. It was, it was making me do less work. That it was so that was a bit that was really helpful.

Moderator 23:23

Okay, so going out of your way to find those external sources.

42 23:27

Yeah.

Moderator 23:28

Okay. All right. So if you live with siblings to what extent were there differences in how much parents supported each of you and your ability to attend class and complete schoolwork? So for example, did you notice differences between the amount of support that you and your siblings received?

44 23:49

Yeah, so my siblings are younger, so like third grade and like maybe like, okay, so they obviously got like a lot more support because they won't do their work otherwise, and my parents usually don't really get on me about my schoolwork. So that was that didn't really change but that's just kind of how it was. That's I guess a noticeable difference.

Moderator 24:11

Okay, and what kind of support do they get specifically like example wise, that you weren't getting from your parents?

44 24:18

Uh just like reminders that they needed to get their work done, and if they like were struggling with it my parents would have sat them down, but I can't really expect my like my kids, or my parents to like sit me down and try and teach me calc like that's just not gonna work.

Moderator 24:32

Fair.

23 24:36

So for me I have one younger sibling in middle school and one older older brother, in also in college, and, um, for me and my older brother we didn't really get much feedback. We were, I mean they expected us to do our work and I think we did it. We did we always do that, it's been that way for like for all of college, and with my younger sibling, it was more of a they well they would help them set it up. Set up the zoom meetings, set up their work, email their teachers. It was more, for us we were more dependent on ourselves and we were more accountable. So, and they needed more help too which they got. It's more of a back and forth thing, to be honest, with them so that's how it varied between my siblings.

Moderator 25:28

Sorry, I missed a word that you said. You said it's more of a what thing?

23 25:31

It's more back and forth with my younger sibling but with with me it's, it's just get it done, the work done.

Moderator 25:41

Okay, and would you attribute that like entirely to age?

23 25:44

Yeah.

Moderator 25:46

Okay.

23 25:46

Because we're more responsible for our work.

Moderator 25:49

Okay.

23 25:50

Actually, yeah.

Moderator 25:52

Understood.

35 25:52

Uh so for me, all my siblings are older. So they've done the whole college thing and moved out so it's me by myself here at home. Uh so I don't think the question really relates to me as much as everyone else.

Moderator 26:14

All right.

18 26:19

Okay, so I have two um younger siblings in elementary school, and I hav e a older sister in high school. So, for them they woke up earlier and then they had zoom meetings. I think they had the zoom meetings every other day. So like my dad will wake them up and then they'll have zoom meetings. And my older sister she, yeah she did her stuff by herself too. Nobody was really on us. We just had to just get it done. Okay.

42 26:57

So, wait can you repeat the question one more time?

Moderator 27:00

Sure. If you live with siblings to what extent were their differences in how much parents supported each of you and your ability to attend classes and complete schoolwork?

42 27:12

Um well obviously my little siblings are like way younger than me so like they like they obviously need more support, because for me, they just, you know, said just do your work and you'll be fine. Like just focus and try best I'll try and keep the kids occupied so you can focus. So they have tried to help me, so I could do my work whenever you know, whenever they had time and I had time. Um obviously my little siblings got more support than I did because they obviously needed more. They they um they they lack the ability to learn by themselves. Um like as you get older you can learn by yourself easier, but for them they couldn't do that. So they all they needed help more often I did so, yeah.

Moderator 27:49

Okay. So now let's talk about what uh other responsibilities you had after campus closed and how these differ from responsibilities you had before campus closed. Describe your caregiving responsibilities including, including child care, elder care, or caring for ill loved ones and how they were alike or different from your responsibilities before the shutdown.

44 28:25

Um I don't, I I can't say that my responsibilities really changed that much.

Moderator 28:31

Okay.

44 28:32

Um I guess I was a bit more responsible for my siblings, because you know, we were in the same house, but for the most part, everyone kind of takes care of themselves.

23 28:44

For me I didn't really have any older or younger family members that I really had to, like, more responsibilities with at home. I guess it was more just household chores. And I mean I guess you at college you kind of do that kind of stuff with laundry and cleaning your room but it was more stuff but it didn't really change that much. It didn't affect my schoolwork at all I would say.

Moderator 29:08

Okay. And obviously, um I'll ask about chores later and household responsibilities too. 35.

35 29:17

Um for me. I don't really have a drastic change. Um it was the same as on campus. Like the previous speaker said, same situation.

Moderator 29:36

18.

18 29:39

So my parents still had to go to work but I just had to just watch my little siblings, so it was really, it didn't really affect anything that much.

Moderator 29:48

How often um were you watching your little siblings?

18 29:52

Before the shutdown?

Moderator 29:54

Before and after.

18 29:56

Um I mean I had some earlier classes and then I'll come home so it was like, five I would say like five hours a day and now.

Moderator 30:08

Okay, sorry go ahead.

18 30:09

And now it's just like, all day I'm just with them.

Moderator 30:13

Okay.

18 30:14

Yeah.

Moderator 30:14

So for five hours to all day. Okay. And 42 what about you?

42 30:21

Sorry my sibling's crying. Uh anyway, uh me, I had uh to, I had more responsibility definitely. Like, um you know in college you the only thing you really have to worry about is your, is your dorm and uh taking care of it but you know when I came back home I obviously had more responsibilities and chores, stuff like that. But I also had to like help my siblings occasionally with their homework uh cuz, you know, sometimes my uh parents had to step out to you know cuz in they're in the medical field sometimes they step out and go do other things. I had to be responsible for my little siblings, help them with their schoolwork. And that'll take a lot because I'm surprised at how much work they get but they get a lot of work. So I had to help them out. I'd sit them down um to teach them the best way I know how and uh yeah but besides that I mean, it was, it was a little bit more work but it was it was okay. It wasn't too much, uh too much extra work you know.

Moderator 31:08

Okay. Um, describe your other household responsibilities such as chores cooking or caring for pets during this time, and how are they alike, or different from before school closed.

44 31:23

Yeah, I was kind of responsible for most of my meals, which is kind of like a good thing because I kind of like to cook. But at the same time, I was also more responsible for like cleaning around the house so like, in terms of vacuuming and stuff that kind of has always been my chore whenever I'm home. So, when I came back home with the shutdown that was once again my chore. Um but yeah, and then I have a cat but I feel like everyone kind of takes care of her so it's not really a, an issue.

Moderator 31:52

Okay, and uh remind me do you have siblings?

44 31:55

I do. Yeah.

Moderator 31:56

So do you have to cook for them too or just yourself?

44 32:00

Really just myself. Like they everyone kind of figures out what they're gonna eat by themselves so.

Moderator 32:06

Okay.

23 32:11

I don't think it changed that much for me as well. I mean, my parents mostly cook the food and I would just like heat it up, day to day. It's, I mean, I did cook, sometimes for myself simple stuff, but like soup or like mac n cheese but I did that in college too. I really don't think it changed there, took out too much of my time cooking.

Moderator 32:38

Okay. 35.

35 32:43

Uh so, for my mom and I we usually like split split up a lot. Um I'll usually do most of it, but given the circumstances we we did kind of split it. Um so when she cooked, I would like do the dishes, and um we'd have certain days when we'd clean up the house you know split it up. I'd vacuum and she did this and that. So we split up the household chores. And, yeah, that's how we went about doing it.

Moderator 33:16

Okay.

18 33:19

Most of the chores um in house came on to me, but like cooking wise, we didn't really cook. We just like we just ordered more food, surprisingly now, and then we then just a whole lot take out

Moderator 33:35

Okay and 42.

42 33:40

Um I definitely had like since I came back, you know, I had to like, you know, clean more, cook more for my little siblings. Um in general, like sometimes for the whole household so that's occa- that's occasional. That's my parents just trying to teach me how to cook and but like also time I would just uh cook food for most of things, you know lunch sometimes dinner um for myself and I had to clean the living room, maybe like once or twice a week. Um, other than that, yeah not much difference so just added chores. Um but you know it's fine.

Moderator 34:19

Now a lot of you might have touched on this so don't feel like you have to go on a long answer again, if you can just quickly restate what you said if you mentioned already. But, to what extent were you expected to take on more household work or childcare, or family caregiving responsibilities than other people in your home? For example, parents may expect more of one sibling, and or partners and or roommates might not contribute equally.

44 34:45

I was kind of expected to take on the responsibilities that I had before I went to college. So like, just generally like the same kind of like cleaning around the house and contributing. Once I came back it was just like, that's kind of expected of me once again.

Moderator 34:59

Okay and is that because um you're the oldest?

44 35:04

Yeah, that and just the fact that like, there's kind of like this like if you're going to be in the house you kind of have to contribute sort of thing.

Moderator 35:11

Okay.

44 35:11

Yeah.

23 35:15

For me, it also didn't change drastically. Um, me and my brother are both in college and my younger sister's in middle school so I mean we did more than her but it was, I think it was pretty equal. We all did did generally the same work where we split it up, amongst ourselves evenly. I think.

Moderator 35:38

Okay.

35 35:40

Um for me, I think I did less household chores than I usually would have under normal circumstances. Had I been back home, I found myself you know my mom and I split up like a lot more. Uh she would do like a lot more of the stuff she would never do like when she was going to work. And let's say during the summer when I'm back home I would find myself cooking, and then doing like a lot more of the cleaning uh while she's at work. So then subtle change.

Moderator 36:14

Okay.

18 36:14

Yeah it didn't really affect me because I was already home, most of the time so it maybe increased like marginally but didn't I don't really see an increase in any like things or chores or stuff like that.

Moderator 36:34

Mhm.

42 36:34

Um definitely an increase. Um I'm at home now so, I have uh some responsibilities that I have to do when I'm at home. Um so like because if I don't do it, no one no one else is going to do it so it has to be done. It's just that kind of thing where you have to do it because no one else is going to do it. Um yeah so that's it.

Moderator 36:52

Okay. Okay. So now let's talk about how your academics were impacted by the shutdown. I will ask how the shutdown has impacted a variety of things, but if you don't think it's impacted something I asked about just say, no impact. In what ways if any has the shutdown impacted your academic performance, grades, and your understanding of the subject matter of your courses?

44 37:17

I would say that my grades weren't impacted so much but my understanding definitely was. Um I felt like uh in a lot of my classes like when I took them in person, in person lectures and had like recitations and things where you could like ask questions, I really understood the content a lot better. But when it came to online classes, it was just like, understand enough so that I can do the questions on the exam, hopefully, and then that's about it.

23 37:44

For me, I think I understanding the concepts and topics didn't really change much as I transitioned to online classes but my grades did improve significantly from the first semester.

35 38:02

I think for me, most of my grades stayed the same and then for classes where I was struggling, I had more time, and they improved significantly. Um though I wouldn't say my understanding of the the classes themselves improved. It was just more when I learned I learned to you know just so I could get the stuff right not to actually learn the content.Yeah.

Moderator 38:34

Okay. 18.

18 38:41

Um, I wouldn't say it really had an impact on my grades, it was just like some of the hands on classes like labs, like doing labs online, it's, it's not doable. I don't, I don't know how that was a good idea but like hands on classes, those had an impact. I hadn't had an effect on my grades. And, like, for example, um, what else calculus, though for that I could just, I just Khan Academy'd, a lot of the topics so it didn't really affect anything. But it was mostly the lab classes that impacted my grades.

Moderator 39:15

And it impacted lab classes negatively right?

18 39:19

Yeah negatively yeah.

Moderator 39:20

Okay.

18 39:21

Cuz watching videos is different from doing the the experiment hands on. Yeah.

Moderator 39:27

Ok. Yeah. Uh 42, what about you?

42 39:34

Um this was kind of weird but like the classes themselves didn't they became more difficult in a sense that you know you had less you had more distractions, uh it was hard to focus um more responsibilities. But what really made it more difficult was the fact that, um because you're at home some classes or essentially um became uh uh people just using Chegg and cheating, so the the standard was raised higher it was not hard, easy to cheat. So that that reflected on the curve so like, uh what originally would have been to get an A or it would have been like you only needed like an 80 right but then because of all the the inflation of grades, it was like, oh, you need a 90 to get an A. So my grades did improve but that was only because I was able to use this pass or fail thing at my school at Rutgers. But if it wasn't for that my grades would definitely be a lot worse than they were um last semester. So yeah, definitely not definitely not a positive thing in in my opinion.

Moderator 40:29

Okay. Okay. Um, in what ways if any has to shut down impacted your career goals.

44 40:41

Um, I wouldn't say it really changed my career goals at all but I would say that I definitely was able to spend a bit more time like planning. That was one of the things that I thought that I I dont't know like I just had a lot more time on my hands. So I kind of like started like planning what I wanted to like where I wanted to apply for like further education and stuff like that, and looking at like requirements and and just, just that sort of thing just like learning a bit more about like the application process.

Moderator 41:08

Okay.

23 41:11

Um, for me, and people with this same career path as me I think it affected them negatively because, like well I'm pre health. So, anyone similar with me, they know like they have to do lots of volunteering, they have to do lots of stuff outside of class, research, all that stuff. And that those opportunities sort of dwindled down because of us being at home and everything like that. So, I think it was, there are less opportunities available for them and that that will be harder for them in the future.

Moderator 41:46

Okay. And we'll come back to once again we'll come back to that topic later about learning opportunities as impacted by COVID-19.

35 41:57

For me, I think um I know for a lot of people in my major they had a lot of internships that were canceled because of the COVID thing. For me I wasn't planning on intern- interning over the summer but I was intending to get like a summer job, and I couldn't do that. But on the other side, a lot of employers are hosting career fairs. And I went to a lot of those virtual career fairs. So, it was good in the sense that you know I got like a lot of beneficial information um that I don't think I would have had, or I would have to have had traveled, like somewhere to get. Uh yeah.

Moderator 42:43

Okay.

18 42:47

Yeah so I'm on so that most of the clinical um volunteering were all canceled so that's like the only because I was planning on doing a couple but they were all canceled. And they all moved to they all moved virtually so those those are not also not the same. So, yeah, that was it.

Moderator 43:09

Okay. 42.

42 43:12

For me career goals weren't really that affected because, I mean, it's I mean maybe it will change if this situation, you know, goes on for longer but for now, there wasn't really a change in what I want to do once I graduate or what um I'm doing right now because I'm right now I'm just aside from school, I was doing research as well. That didn't change. We were able to transition to an online format. It was harder but you know I'm still doing it so it wasn't, there wasn't really that much of change, otherwise.

Moderator 43:42

Okay. Um, so in what ways if any has the shutdown impacted the availability availability of other learning opportunities such as internships? And don't feel pressured to add to this if you've already talked about it but if you haven't talked about it yet and you have something to add, feel free.

44 44:02

Yeah, so I had an internship a research internship lined up, and then it kind of got moved online and it's kind of difficult to do online research so like the whole formatting and like structure of the whole program kind of got shifted. So I guess in a way that change I didn't really get the research experience that I was looking for. Um and then I, you said the question was about like learning opportunities?

Moderator 44:25

Yes.

44 44:26

So one other thing that I found that really helped me like last semester was supplemental instruction. So that was just kind of like a thing that our school did. It was kind of like, I guess you could consider it tutoring. It was just like extra help, if you needed it. So I found that was also really helpful but because of how everything kind of got shut down that got moved to an online format and that was just not as effective or helpful as the in person one so that kind of that fell off a little bit.So, yeah.

Moderator 44:55

Mhm. Ok.

23 44:55

For me I wasn't personally doing any research or internships, but I know there's lots of people in my position that were my age that were. And I know that you can't really do biological or chemical research at home. Um so, that certainly affected, a few of my friends who did have that lined up.

Moderator 45:24

Okay.

35 45:27

Yeah. Um I also didn't have any internships or anything of that sort lined up, but I do know people that were affected, had theirs canceled as I previously mentioned.

Moderator 45:43

Ok. 18.

18 45:45

Yeah I really have nothing to add besides everything being moved online and closed.

Moderator 45:51

Ok and 42.

42 45:53

Yeah, I didn't really apply for any internships. Um just as I said before I did, I did research under research. I was doing research before, luckily it got it transferred over and yeah so not much changed my previous answer. So, just now that I'm doing um research and that's it, but it wasn't really affected, so.

Moderator 46:13

Okay. Did new learning and or career development opportunities emerge during the shutdown and if so what were they?

44 46:20

Um no, not really.

Moderator 46:32

Okay.

23 46:38

For me no as well.

Moderator 46:39

Okay.

35 46:41

Sorry, may you repeat the question for me?

Moderator 46:43

Sure. Um, did new learning or career development opportunities emerge during the shutdown and if so, what were they?

35 46:51

Oh okay. So yeah, um I found a lot of people, like a lot of employers. I was in career fairs and informational sessions for people in my major so I attended a lot of those, and you know, I'd get like my one professor would like email them to me. Or like um the actual college would like email them, email us like a lot of like seminars that were happening online, and I attended quite a few of those and I found they were quite beneficial in that they gave me a lot of information related to my major that I didn't know, and I feel will be quite good for when I start applying for internships this coming semester.

Moderator 47:38

Okay.

18 47:41

No, I I have nothing. No, nothing like that.

Moderator 47:44

That's fine. Uh, 42.

42 47:47

Uh same here. Nothing, nothing happened.

Moderator 47:51

Ok. In what ways, if any has the shut down impacted your confidence in your ability to successfully complete your bachelor's degree at all or in a timely fashion?

44 48:06

Uh it it hasn't.

Moderator 48:07

Okay.

23 48:09

I also don't think it has. I'm still on the same track, and I think it'll be fine.

35 48:16

Um, I think last semester was actually quite hard for me, it was very difficult. So, I think I gained confidence, after, after the shutdown, because I started doing better in like classes I was really struggling and I thought you know, I'm not gonna, I'm have to withdrawal from like one or two of them. But then after shutdown I had more time. Uh and also the the syllabus changed like the way we had to do it. Like I found uh there was like a restructuring, in terms of like the marks and stuff and you know we got like discussion boards and more homeworks, more labs. So that really helped to boost my grade in some of the subjects.

Moderator 49:05

Okay.

18 49:07

Um it hasn't really affected it. I'm still on track to graduate within four years.

Moderator 49:13

All right. 42.

42 49:16

Um for me I'm just I mean, I'm I'm on track, but like everyone else but it's just made it more difficult to do well, I would say. Um you know, as I said before, because the standards I mean I wanna assume it's going same, in the in the next semester in the fall semester. But, um the standards to do well have shot up drastically. Most of my classes just because if you know a lot people in the class, you can always work together on a test or homework or whatever, but if you don't you're basically on your own. It's like it's like not accomplished kind of shocked cuz like I feel like I have to like do 10 times more work or work to or like be 10 times better if I want to do, uh as well as I was doing before. So uh that's basically it.

Moderator 49:57

Okay. Understandable. In what ways if any did the campus shutdown impact your finances?

44 50:08

It didn't.

Moderator 50:10

Okay.

23 50:11

I don't think that it had an effect on my finances, as well as well.

Moderator 50:16

Okay.

35 50:16

It didn't have a ma-. Sorry, uh it didn't have a major impact on my finances. Um just you know I think I would have had more if I had done the summer job, but nothing major.

18 50:34

Yeah, like everyone else. It had no impact on me too.

Moderator 50:37

Okay.

42 50:39

I think it actually improved my finances, in a way. Uh in other ways, it didn't, but like, in terms of, like, tuition and stuff, my school gave back uh money for the fees um because we went halway we left halfway through the semester so they gave us some money back so that helped a little bit. Um and the following semester I don't have to pay for room and board. That's a plus. Um they gonna also give us more um give, reduce the campus fees and stuff like that so we we'll have to we'll pay less to go to go to school and but one way it did impact negative is like I can't get a job now. I can't get job that I want to get. Um you know that's what but I think, like, in all I did save money in this. That's one plus I saved money so.

Moderator 51:24

Okay. So now let's talk about what your professors did and did not do during the move to online learning. What are some examples of strategies, tools, or technologies that your professors used that you found to be very effective during online learning? In other words, what do they do that made it easier to learn?

44 51:42

Um I had professors who moved like office hours online and then would have like host like like multiple times during the week where you could just like ask them questions. So I guess they kind of like opened up their office hours a bit more because they knew that we couldn't really just come to them whenever we had questions or ask them after like lecture or something so they were able to make up with that. And I think this specific professor used uh Microsoft Teams, I think. Um, but yeah. I think that's about it.

23 52:21

I don't think there was much use- much used by professors. I think some professors, well, one of my professors offered a bunch of office hours. He really was hoping to help everyone. I also think some professors sort of were more lenient. Gave more extra credit, stuff like that, rather than do in person stuff but each professor was different.

Moderator 52:47

Okay.

35 52:49

Um, what I found for like majority of my classes uh lessons were synchronous. So, you would have like a lot of pre recorded lectures that you could watch. For me it was good cuz in a lot of the classes I had like one class in particular where I didn't really understand the professor's teaching, and um it was like a Cisco network, network networking class and I stopped, you know, watching a lot of the lecture videos. I had more time to actually do the work. Um and watch other people's videos and do the work by myself, then independently. So I feel for stuff like that, it really helped. Um another instance is the professors were more lenient. Like I mentioned before they changed the syllabus a little bit like the assignments introduced some, and the changes, switch the grading around a bit um to make it easier for students affected by the shutdown.

18 53:58

Uh there was an increase in office hours and tutoring. It was way more tutoring than there was before the shutdown. And the the professor were actually pushing us to actually go to tutoring.

Moderator 54:14

Alright.

42 54:17

I found that the best type of class was the ones that were they pre recorded lectures and you're able to watch it um at your own convenience, and you're able to like rewind and watch it over and over, watch the parts you didn't understand and you're given more time to understand the concepts. Um as well as having like uh a designated time for, you know, office hours so we could go and ask your teacher or a prof- I mean your professor, um you know, questions, they didn't understand from the lesson. That was that was pretty useful. Um that was by far the most effective form of classes, um in my opinion.

Moderator 54:51

Mhm okay. And now um kind of like the opposite of that question. What are some things your professors did during online learning that uh that was ineffective, or that did not help you learn?

44 55:06

Yeah, the one thing that comes to mind is like like lengthening lectures. So because like lectures were online they weren't kind of restricted to that 50 minute, or I guess hour or whatever it is um limit so they would just make the lectures an hour and a half to fit all. And that was not help because I definitely did not have the like mental capacity to sit there and watch an hour and a half of like, calc- calculus. It just wasn't it wasn't effective. Um yeah. Ooh one more thing. I think they definitely graded a lot harsher. So I noticed with my French professor, she just like the stakes were like raised, and she just expected us to have like perfect grammar, perfect everything. So like, even if like you used all sorts of like grammar checkers or whatnot, she would still dock you points, because it wasn't like perfect and I noticed that like that definitely was not how it used to be. So I think the standard was definitely raised.

Moderator 56:05

Okay.

23 56:08

For my, I had two lab classes and for both of them it was, it was more of uh them recording that experiments what they would do. Then we would like write the lab report as normal. There's not much else they could do. I feel like with labs, it's really limited with online. So, I didn't really like learn the concepts of inside the labs and how to do the experiments, but it's not really, there's not much we could do with labs, in my opinion.

Moderator 56:40

Mhm.

35 56:42

Uh could I get the question one more time, please?

Moderator 56:44

Sure. What are some examples of strategies, tools, or technologies that your professors used that you found to be ineffective, or that it did not help you learn during online learning?

35 56:54

Okay. Um so, I think I agree with 44's point where he said um I-- the lectures were really long. I mean, they did split up uh maybe like on one day I get like five 30 minute videos or something like that but it was still a lot to take in. Um and I found I had to like split it maybe like a one day's worth of lectures over like three days, or two days depending on how much content and how interested I was in the content. So there was that, and um I think that's about the only issue I had with the learning online, the length of the lectures.

Moderator 57:46

18, what about you?

18 57:48

Um so the only thing was, like doing tests. The, the professor's made it um, what was it? They gave us like little time and they increased the amount of questions that we had. And there was no backtracking, so it made it like if you didn't know the answer, you just have to guess. And, yeah, I was. that's.

Moderator 58:14

Mhm. Okay. 42.

42 58:21

I found the most ineffective form of teaching was when they would just basically just give us slides, and just say good luck, you're on your own. Have fun. I was like, you're not going to help us at all or not? So like basically they'll just give us um a bunch of slides for to look at, and then they just give us a test and the homework, but then there'll be no interaction with us and the teacher, like she would not, they would not have, there were not in, there were not like office hours where we could ask questions. It was just, you were just on your own basically. That was that was that was horrible. Um also like uh tests where like 18 said like, where you couldn't backtrack um cuz one of the best ways to do a test is that, you know, you go to the questions you can do quickly and then go to the ones that are harder, they need more time to solve right but in this case we couldn't do that so you had to like, if you'd didnt have enough time you'd have to guess. Just move on to the next question. That was, yeah that was by far the worst.

Moderator 59:10

Yeah so when teachers didn't teach it was hard to learn. What are some things your professors did during online learning that made you feel like they cared about their students?

44 59:29

I think they like increased office hours, and just generally like they kind of made it a little bit more clear like hey if you're ever like struggling with anything and you need like extra accommodations don't, feel free to reach out. So that was pretty helpful.

Moderator 59:45

Uh huh.

23 59:48

I think um I also agree with the office hours, and my TAs also had more office hours, which was really nice because they're also students and they were going through what we were going through as well. With everything and I mean, some teachers were helpful in that they were a little bit lenient and understanding. Maybe some were just a little bit lazy, but I think they sort of understand what we're doing in the transition, it just wasn't it wasn't natural for us so they helped us through that, to the best of their ability.

Moderator 1:00:22

Okay.

35 1:00:22

In general, uh a lot of my professors, gave us like uh more time to do work than they would usually have given us. So maybe I found I would get like one assignment every two weeks for some of the class, or um I just have more time and there were more accomodations. So if you need an extension they were pretty lenient on that regard. And then there was one professor I had that particularly stood out where uh she would go past the academic aspect and be like hey if you guys actually need to talk or anything. I know some people went through like you know, it was quite traumatic going back home because like their situation back home is not so good so if they ever needed to talk she she gave them you know that option if they you know, and she wouldn't share with anyone else, the school, anything. So if you just wanted to talk, you could come to her.

18 1:01:21

Yeah like 35 said most, most of my teachers were very accommodating. So you could you could hand in work like if you asked for an extension, like a week, essentially they're going to give it to you. So that was really. And also, most of TAs had had a lot more um office hours and you could email them at any time and they will answer back.

42 1:01:46

Um may-- can you repeat the question one more time? I just want to make sure I got I got it right.

Moderator 1:01:51

Sure, what are some things that professors did during online learning that made you feel like they cared about their students?

42 1:01:58

Oh, um like when they gave they reduced the number of tests because in my, in my um school they they gave me like three midterms and a final. When there was a day um, two or three midterms and final and what they did was they reduced it to like most times like one midterm and then the final, which could be bad or good but they they did try to make the tests, they gave us like the, uh the whole day to do the test. Like I mean we would have like a certain window. Like we only have two hours to do a test but we could do it at any point in the day. That was nice um you know, cuz you know my my schedule was pretty busy. I have a lot of siblings so I, I could pick time and I knew that I could just focus on my test so that was one good thing that I liked.

Moderator 1:02:38

Okay and what are some things your professors did during online learning that made you feel like they did not care about their students?

44 1:02:51

Lengthening lectures. That hurt. Um let's see, also just kind of like raising the standards and like, just, yeah. Um let's see. I also felt like online labs were just kind of brutal and, like, they didn't really do enough to help explain and like, help us understand what was actually going on because that was like kind of the big issue with online labs. Like when you do the lab you know what's actually happening but when you're not doing the lab and you're just kind of like getting a dataset and a couple videos, you have no idea what's actually going on. So I felt like they could have been a bit better about that. Um, yeah, that's really about it.

Moderator 1:03:35

Okay.

23 1:03:37

For me, I had one professor that we usually had two lectures per two in class lectures per week and he would like wait til, It was Tuesday/Thursday and he would wait til Friday, and he'd post both of them and it was an hour and a half long class it would be three hours of lectures. So that, like clumping everything together-- together didn't really help us giving us lots of lectures just to go over real quick. I didn't like that.

Moderator 1:04:03

Okay.

35 1:04:04

So I had one professor where um they didn't-- when you email them sometimes they would not respond in a timely manner and I found myself having to email the TA. Um like, especially for like the final, everyone was in a panic over the final cuz we were given about a week to do it. Um it was like, more like a final project, like a coding thing, and um he was supposed to release it on Tuesday, and we all emailed him, no response, and then we had to email the TA. And the TA also was like, he went MIA on he's not responding to my emails and uh and we ended up having the final project released on the Thursday. And that was my biggest problem with the, with the classes ok.

Moderator 1:05:04

Okay. 18.

18 1:05:05

Yeah so I had some of my professors not um answering back. That was really, that was really it.

Moderator 1:05:11

Okay.

18 1:05:12

At least we, most of us we um sent mass emails and they just never responded.

Moderator 1:05:17

Mhm.

18 1:05:17

I think some of them even blocked us. Yeah.

Moderator 1:05:24

42.

42 1:05:25

When teachers were totally unflexible to the rules. Cuz um what happened was that one of my friends was doing a test and he had to --to, basically was it-- it was a calc test so he had to like take pictures of each because it was like he had to write down the answer, do the math, you know take pictures of it and upload it, right. So, and they also had a time limit for the test. Um so what happened was that he had an internet connection um unstable in the connection and, and it didn't upload in time, so he quickly he emailed it to the professor, but the professor still gave him a lot of trouble for it, like he was trying to-- he was trying to explain to him that that his internet cut out, so he couldn't um upload it on time but he sent it to email at like two minutes after the deadline. So I don't -- but it still gave him a lot of trouble for it and eventually he eased up a little bit, but the, but the fact that he gave him so much trouble for that what happened even though it was out of his control was showed that he just didn't care. Yeah.

Moderator 1:06:22

Right. Okay, um, it is past five o'clock so I do have to let you guys go, but thank you for participating in the focus group today. You're going to receive a follow up email with the rest of the questions and we ask that you respond to those along with the name that you used here today. And yeah, that's all I have for you guys today. Thank you for coming.

**High SES Black Men Email Responses**

1. What are some things that your university did (e.g., pass/fail options, townhalls, remote counseling) to help students be successful during online learning? Mention any resources the university provided or actions they took to help students be successful or feel supported.

2. What do you wish your university did to better help students be successful during online learning?

3. In general, are there any ways that your ability to be successful in school was affected by the pandemic that we have not discussed?

**Survey ID: 44**

1. Our school allowed students to opt in for a pass fail option to help students feel supported and our wellness center/gym sent out home workouts and activities to keep people active

2. I wish our university set limits for how much work professors could assign, especially considering a lot of students also had to work at the same time and support their families.

3. I can’t think of any!

**Survey ID: 18**

1. My university introduced a credit no credit system for any classes taken during the Spring 2020 semester. The Counseling and wellness center had weekly workshops as well as one on one counseling.

2. I wish teachers had not increased the workload just because everything was online.

3. It was harder to be more productive. Going to classes felt more like an option than something I actually had to do.

**Survey ID: 35**

1. They introduced an optional pass/fail system for students and allowed students to selectively withdraw from their classes if they were struggling.

2. I think the university did a very good job, and I think there is nothing major to improve on.

3. I think people's mental health was greatly impacted by the pandemic and I know a lot of people were impacted so much so that it ended up being detrimental to their school work.

**Survey ID: 23**

1. My university as a whole did offer the pass/fail option. My university also promoted and encouraged us to have more meetings with advisors and professors. I ended up meeting with a science advisor to discuss classes and a research advisor to discuss potential research opportunities in the near future.

2. I wish my university let us give feedback on the professors and their teaching methods so that changes to cater to each student could be made through the semester. After the semester ended we were able to give feedback, but I think It would have been more helpful if this was available throughout the semester

3. I do not think my ability was impeded by the pandemic, but it certainly was a bit of a stumbling block. We had to adjust drastically to the changes that were put in place. I think ability to adapt is crucial in order to be successful, so in the end, I think it will help us in the long-run.

**Survey ID: 42**

1. They did have a pass or fail system in place so I GPA's wouldn't be affected and they extended it into the summer. They also did provide remote counseling services which was really helpful.

2. I wish they had better software systems in place to help with online learning because the ones they used weren't effective.

3. No, you have covered all the points.

Moderator 17:41

Alright, let's go ahead and get started. That was the longest three minutes of my life. Um, so welcome to today's focus group. My name is Redacted, I'm a research coordinator from Redacted. Redacted is our note taker today if you could just wave to everyone Redacted, so they know who you are. Thank you so much for helping out today. So today we're going to be asking a number of open ended questions. There's no right or wrong answers but people may have differing points of view. So feel free to share your point of view, even if it differs from someone else. Keep in mind that we're interested in, negative comments, just as much as positive comments. So we want everyone to feel comfortable participating today. So, because, but because we only have an hour, we may have to cut you off so I apologize in advance. I'll raise my hand like this so that way you know to finish your thoughts so we can move on to another person. We want to get the full breadth of experiences and opinions from everyone today but some of you may have very similar experiences and opinions as someone else. If your answer is the same as someone else today. You may simply say, my answer is the same as Sam's. It is difficult for us to take notes on everything that's going to be said today. So we're going to be recording everything for transcription purposes, but we're not going to associate your name with anything you say. In addition, you may refuse to answer or withdraw from the group at any time. We understand that it's important to keep information private and confidential, and we're going to ask that everyone respect each other's confidentiality, by not repeating things said within the group today. Um, Redacted if any if there's any more late arrivals, you can go ahead and let them in. Um, because I'm gonna go ahead and change my screen view. Alright, are there any questions before we get started? All right, let's go ahead and get started then. So first we're going to start by going around and each person saying your name and what year of college you just finished and one word to describe what your life has been like during the pandemic. So let's go ahead and start with 2. That's what your the Zoom says.

2 19:55

Hey

Moderator 19:56

Hi

2 19:57

Hi

Moderator 19:57

So, what's your name, year in college, and what year you just finished,and one word to describe what it's like during the pandemic for you.

2 20:05

Hey, alright so my name is 2, um I'm a returning student um I just finished my sophomore year at um REDACTED which is uh a part of the REDACTED system. Um I'm studying public health. Um and one word would be, I'm sorry, uh and one word would probably be disruptive.

Moderator 20:28

All right, thank you for sharing, Um 12. So, could you say your name, year in college you just finished and one word to describe what it's like for you during the pandemic.

12 20:37

Yes, I'm 12 I just finished my freshman year and I'm going into sophomore year, I'm studying physics, and one word I would use to describe it would just be hectic, I guess.

Moderator 20:51

All right, and 11.

11 20:52

Uh, my name is 11, I just finished my freshman year at the University of REDACTED and one word I would use to describe would be just like anxious overall.

Moderator 21:06

All right, and 4.

4 21:08

Yeah. Hi, um I'm 4, I'm an electronics engineering student I just finished up my third year. Um I guess one word to describe everything since all this COVID stuff is has been stressful.

Moderator 21:22

Alright everyone, thank you for sharing and um partaking in that little icebreaker. So let's go ahead and move to the focus group discussion. So the COVID-19 pandemic has presented big challenges for undergraduate students across the nation. As you know, many universities across the nation have closed moving all instruction online with very little time for students or faculty to prepare. Many students have encountered challenges related to the closing of campuses, relocation, and the movement online instruction. So in this study, we're trying to learn more about how the closing of campus and pandemic has affected students in general. And so today we want your opinions about the good and the bad on how things went well, or didn't go well, and what your professors and universities did that was helpful and unhelpful and what you wish they would have done. So we're going to start today by discussing where you went when your campus shut down. And I'd like to hear from each of you. So we can start with 2 again is, I'd like you to tell me about the space in the home that you move to so what space, did you have to do your schoolwork and was this space private or shared and if you shared this space, who did you share it with?

2 22:31

Okay, so I actually I moved back to my childhood home, um which was a little disruptive and it's, you know, uh so I you know I have my, my own bedroom, but you know it is, it is my childhood bedroom. Um and, you know I didn't have to share it. Um, and, you know, I did, I moved into a house at least you know so that I there was enough space so wasn't that you know, that wasn't one any of the issues of having to share space.

Moderator 23:04

Okay. And 12. So could you tell me more about the space you moved to and what it was that you, the place that you had to do your schoolwork and whether it was private or shared.

12 23:14

Um I just went back home. Um I live with my family, most of the year so I just went back during spring break and then they canceled everything after that. Um while I just studied mostly in my bedroom, it was kind of distracting because my dad was working from home and he's just very loud with his work so it was, it was just a very strange environment but it was kind of alright, since I just was closed off in one room by myself.

Moderator 23:40

Okay, and 11. What about you?

11 23:44

I have the same answer. I went back home. Both my parents are working from home, I ended up uh having a desk to myself but sort of the same situation as everyone else.

Moderator 23:55

So it was a private space like a closed off room by yourself?

11 24:01

Yeah, it was. There was definitely interruptions and you know parents popping in and sound overlapping so it was not ideal or as ideal as you know a library space or anything like that, but there was you know a door in between us and everything.

Moderator 24:15

Okay, thank you. And 4. Right.

4 24:19

They're both they both work. Um for me, mine is also pretty similar. Um I was on spring break and then they randomly during while I was still still at spring break, they're like, hey, everything's online, you know you can't come to campus for a bit. Um and so I was at my childhood home, I have which is like you know I have my room and everything. Um the thing is I have three younger siblings, which can, which made everything a lot more disruptive my parents were working remote too. So it was a lot of just hectic stuff going on. In addition, since they cut it off during halfway during the semester, I didn't have everything that I needed to do my schoolwork, so I had to like go back and grab everything and then come back um once they let us but that was like pretty well into my education. So, it was, it wasn't ideal.

Moderator 25:05

Okay, all right, but when you say it was the same as everyone else, what you mean is kind of like a closed off space, even if there were some interruptions?

4 25:12

Yes.

Moderator 25:13

Okay. So, everyone has kind of touched on this a little bit but we want to know what challenges you encountered attending class or doing your schoolwork in your space, so we can start with 2 again this time.

2 25:28

Um, I would say, staying motivated was like very difficult for me. Um, I, you know, kind of, because my schedule was so like disruptive it was hard to manage my time. Um the days kind of started running into each other. Um and it was, you know, it was hard not being like in a classroom able to, you know, speak to other people, you know, having to do everything like over group chat. Um yeah, those those are the probably the biggest.

Moderator 26:04

And so, particularly in this space, um, what was could you tell me a little more about, you know, maybe in this space what was challenging for you?

2 26:15

What do you, I'm sorry, can you clarify?

Moderator 26:17

So, um I think before you had mentioned that you know there were noise. Um so a little bit more about kind of like this new space, um

2 26:26

Oh the space itself?

Moderator 26:28

Yeah

2 26:29

Um I think just kind of, um it got a little monotonous just being in the same room, all of the time, you know like not being stimulated, um having to set a timer to get up to make sure I stretched, you know at least every two hours. Um you know, those those were probably the biggest.

Moderator 26:52

Okay, thank you so much. And 12. What about you?

12 26:56

Um, for me, I would say the biggest problem I had was time zones because the school I go to is a couple states away from where I live um when I'm not at school so it was a big problem with, like, FaceTime meetings, there was a lot of confusion with a bunch of students and the fact that they wouldn't let us go back to campus to get our materials. So it was just like a lot of online work that wasn't really working out for a lot of people for like time management and stuff but for the most part I found it was not too confusing doing it like at home instead um just because my parents were really respectful and not coming in, when they knew I had like a meeting or something but I would say the whole timezone thing was a big problem for me personally.

Moderator 27:44

All right, and 11.

11 27:46

Um, so the space itself. I think my biggest issue was the fact that there weren't other people around. I really feed off of other people, even if it's in a quiet environment I just like being around other people and I like having the option to go get a coffee and just take a break and stuff like that. Another issue with my space itself was my organization got dramatically worse. I had a backpack but I wasn't really using it and I just had a lot of printer paper everywhere because I was using it to scan for homework in different classes so my organization just got really bad and I just had piles of paper from various classes on my desk kind of all the time. So.

Moderator 28:31

okay, and 4.

4 28:33

Yeah. So for me, my space itself was like fine. Um I think I usually when I'm in class like physically in lecture um I don't have my computer out because I find it hard to focus. So whenever you're like doing all your classwork on a computer that really kind of messed with my uh learning because I'd always get distracted by my computer. Um on top of that my little siblings, um I haven't like since I do, I work during winter break and summer break um with internships, I haven't been home for a while so my siblings are obviously really excited when I came back so they would constantly kind of like come up to my door and like you know want my attention want to like spend time with me and stuff, which was like obviously I want to give it to them but it's really stressful when you're doing schoolwork um on top of working. Um and I think my major's a very hands on um major so we have like these little development board kits that we have to utilize in our labs for schoolwork and I didn't have access to those when our professors expected for us to have those, so not having access to my school materials really threw off my learning this semester. Um but those are like my biggest challenges.

Moderator 29:38

All right, thank you everyone. So what are some things that you or other people in your house did, that made it easier for you to attend classes or do your schoolwork. Um, we can start with 2 again.

2 29:50

Um, I think I would make out a schedule of times when I couldn't be interrupted. And you know I needed noise like don't run the vacuum, don't do the blender. Um and you know I respected, my, you know, my mom would do the same and we each respected each other's space and time. Um, and you know, I think if I needed a break and I needed to go into a different room, you know, being able to swap with her like, I would do the office, she would work in a different room um just to to uh just so it didn't get so repetitive.

Moderator 30:28

Okay, and 12.

12 30:31

Um I would have to agree a little bit with 2 on that one like my family did this thing because my sisters were also doing school from home, where we like printed off our schedules for that week um and taped it on our doors so people would know not to go in, during like specific times. Um just so there wouldn't be as much like interruptions as there would have been otherwise. Um I feel like that really helped in the long run, just so we wouldn't get distracted when we were trying to do work and stuff so I think just doing that really helped me.

Moderator 31:03

Okay. All right. Thank you, and 11.

11 31:06

Um, my mom switched rooms with me so I took the office and she moved into the family room just because I needed a desk space whereas she kind of just needed a laptop and a table. That was one thing. Another thing, they just really tried to make me happy. Whether it was you know asking me what I wanted to eat and making sure I had things in the house that made me happy. They also allowed me to redo my childhood bedroom, which kept me busy when I wasn't doing schoolwork, but it also provided me with like a newer space that really felt my own so they just really tried to do their best to make sure I was happy and still working and everything.

Moderator 31:45

Alright, thank you so much and 4.

4 31:48

Yeah, so for me it was um I would, I technically would be busy from about 8am to 8pm every day during the semester, because I guess expectations kind of shifted when everything went remote. Um so I wouldn't really have a lot of chances to like eat during the day so my mom would essentially bring meals to my doors so that you know I wouldn't have to disrupt what I was doing, while um, you know, but I still needed to eat. Um on top of that my parents didn't really expect me to do a lot of, you know, chores around the house any housework. Um there wasn't an expectation of me, just because of time. Um and on top of that if I was ever feeling like overwhelmed or stressful both my parents, we'd like sit down and they've helped me calm down, stuff like that and be less anxious. Um those were really helpful.

Moderator 32:32

All right. Thank you all. So if you lived with siblings, we want to know to what extent there were differences in how much your parents supported each of you and your ability to attend classes and complete schoolwork. So, for example, were some kids in the house given more time, space, or consideration to do schoolwork by parents and others. 2 did you mention you had siblings?

2 32:53

Um I'm actually an only child. So, yeah, so it doesn't apply to me.

Moderator 32:58

And 12, I think you might have mentioned you had siblings.

12 33:01

Yes, I have another sibling, who is in college right now and then one who is in high school. So, for like the two of us that were in college, we constantly had our parents like reminding us getting on our case about getting stuff done and turned in on time, but more for like my younger sibling, um they kind of just let her do whatever she wanted to do so that was kind of helpful for like the Wi Fi situation where we would get everything done before, like 2pm, on a day and then she would like, do it after us. So everything worked out kind of like that but yeah I would say definitely for the um those of us actually going to college, it was a lot more intense for them like their attention for that so that was kind of my situation.

Moderator 33:48

All right, thank you 12. And 11.

11 33:50

I'm an only child.

Moderator 33:52

Okay. and 4

4 33:54

Yeah so I have um my four siblings, but one of them's already graduated so it's kind of irrelevant. Uh so my three younger siblings, I have one in high school, two in elementary school. Um when it came to me my parents don't like get involved with my schoolwork at all. I can handle it all myself. I don't really have them be involved with that at all. But both my parents were working full time. My, my, um, my sister in high school, essentially, she kind of was expected to sort of, I guess, arrange her own schedule, like she was supposed to know when her lectures were at with her class, because when her class was meeting on zoom she was supposed to keep up with that, keep up with her AP exams and stuff like that. Um just because my parents didn't have the capacity to essentially work full time and run three different like schooling systems for the kids because with my younger two siblings, um their teachers essentially gave work packets and gave all this work and was like, oh you know for this library class you have to read them this book, there's a lot of, I guess, step by step intense schooling for them that my parents couldn't keep up with because they were working. Um and so I think like to an extent like my younger siblings, with the ones in elementary school, definitely kind of were expected to know what they're supposed to do, um and kind of facilitate their own education. It didn't work out super well for any of my younger siblings just because of how hectic everything was. My younger sister slept through a bunch of her zoom meetings and missed one of her AP exams, um because you know at that age it's a little bit difficult, uh because the structure of being in person helps a lot. And my younger two, the ones that elementary school really were just like, you know, they would pretend to do the work, and then not actually do it but it was hard for my parents to keep track of that just because you know how hectic everything was. Um if that answers the question.

Moderator 35:37

It does. Uh thank you all. Um so in our next set of questions, we're going to talk about other responsibilities you had after your campus closed and how these differed from responsibilities you had before your campus closed. So let's start with caretaking responsibilities. So could you please describe any caretaking responsibilities that you had. So this can include child care, elder care, or caring for ill loved ones. So we want to know how your caretaking responsibilities after the shutdown were alike or different from your responsibilities before the shut, shut down. Um if this didn't affect you, you can just say, this wasn't, this isn't relevant. So 2.

2 36:14

Um this uh. I'm trying to think of. Maybe because I was spending more time with my mom, I didn't, you know because I was, we were like living together 24 seven. Um I didn't have, you know, I didn't have to schedule time to do things with her and like pay attention to her. Um so that made it a you know that made it a little easier. Um and I don't you know have any other uh caretaking responsibilities.

Moderator 36:45

Okay, and 12.

12 36:48

Um this wasn't really relevant to my situation at the time.

Moderator 36:52

Okay, and 11.

11 36:54

I would say just being more aware of how other people are feeling because living together 24 seven but other than that this is not relevant.

Moderator 37:03

All right, and 4.

4 37:04

I guess before, obviously COVID hit I didn't have to deal with like my siblings, for the most part. I'd call them every weekend or so but since I wasn't there physically, I wouldn't have to worry about it. Um whenever I came here I was kind of expected on the weekends to, you know, spend time with my siblings because during the week I wasn't able to or like you know at night if my parents had to go out, um you know for work or anything I was expected to take care of them kind of develop devote more time to, you know, making sure my siblings are doing okay compared to beforehand. Um but besides that, nothing else.

Moderator 37:40

Alright so now let's talk about your household responsibilities and how they changed before and after the shutdown. So please describe your other household responsibilities such as chores, cooking, caring for pets during this time and how they were alike or different from before your school closed. So 2.

2 37:57

Um, so usually there's a, like a cleaning person who comes in, but because of the virus, um I took over most of it just because like we didn't want, it wasn't safe to um have an extra person in the house. Um so just like normal, you know, vacuuming, doing the bathrooms um and the kitchen, dusting. Um we com, there's a compost um center by our house, and they closed down for the virus. So, you know, having, so we did our own composting. Um you know, doing the doing starting to do most of the cooking because we couldn't go out to restaurants. That's basically it.

Moderator 38:40

Yeah and was that a lot different than kind of whatever your situation was before

2 38:45

Yes, um I have a newfound respect for people who do this all the time. It is a tremendous amount of work and it feels like the second you end, you have to start over again, like it's just it's just never ending.

Moderator 38:58

Mhm. And 12, what about you?

12 39:03

Um, so I didn't really have to deal with it too much when I was at school but as soon as I came home I was in charge of like taking care of our dogs that we have, um because my parents really couldn't take them out for walks or like, make sure they were doing all right when they were like, on meetings and stuff so whenever I would just have a break from school I would take care of them or I'd be in charge of like driving my sister to and from work because my parents couldn't do it. And I could just like schedule around that so that's kind of where I was at. Um in addition to like cleaning more than I probably would have prior to this just for, I don't know, something to do.

Moderator 39:41

And 11.

11 39:44

I would say I didn't really pick up any more or less responsibilities as far as all those things go. But I definitely was doing more cleaning and cooking and baking and things like that just because I was really bored and I am a person that likes to be kept busy so I did those things more for fun as opposed to a responsibility.

Moderator 40:06

Okay, and 4.

4 40:08

Uh yeah so I guess as far before uh COVID hit I was like help, I was kind of in charge of my, like you know, not my dorm but like I had a bunch of friends we were living together who were younger than me so I kind of like you know did chores and I had this certain amount of responsibility at that house. Um and so you know I was cooking, cleaning, whatnot I do that consistently. And then when I came back home when I was still adjusting to classes initially for the first like month and a half I wasn't, you know my definitely my responsibilities lessened because I was struggling to maintain everything and my parents kind of helped out with that. Um but then I think Ramadan happened during um quarantine. And like I'm Muslim so, I think, it wasn't until and so once I was getting more accustomed to actually doing lectures and stuff like that um I would actually cook while I was listening to lectures, um you know, help my mom cook and everything just to make sure that you know we got done because there is a lot of cooking involved. So like after that I guess once I got more adjusted to everything my responsibilities did increase.

Moderator 41:10

Alright, thank you all for sharing. So this next question is something that you guys have already kind of gotten at a little bit, but we'd like to know to what extent you were expected to take on household work, child care, or other caregiving responsibilities in your home. So for example, parents might expect more of one sibling um and there might not be the same sort of contribution throughout the people in your household. Um 2 we can start with you again.

2 41:38

Um, you know, I don't know. I don't know if it was expected just because it was like it was so sudden. Um there's just two of us in the household. And, uh you know, my mom had to adjust to uh working from home as well so you know I wasn't asked to do it, but I could see how much she was struggling. Um you know, I just kind of took it on myself.

Moderator 42:03

Okay, and 12.

12 42:05

Um so personally I don't think anything was like, super expected of me but considering both of my siblings had like, seemingly heavier workloads going on with their school um both of them going towards like graduating this year. Um I kind of just decided that I would step up and do all of the chores that we were asked to do collectively. So I think I kind of just put that onto myself but at no point was I ever really like encouraged to like do things like that they wanted me to do they were just kind of like hey if you're not busy go ahead but I kind of just decided I was going to do it myself.

Moderator 42:44

Okay, and 11.

11 42:46

I wasn't outwardly expected to do much of anything different, but there were definitely undertones when it came to like waking up in the morning and being a person and an active member of you know society and my household. So I feel like there were subtle expectations, just to avoid like disappointment almost if that makes sense.

Moderator 43:12

Alright. Um and 4.

4 43:15

Yeah. So besides like you know being I was definitely outright expected to help out with my siblings wherever possible, um just because again it was too much for my parents to handle on by themselves, but like you know the cook cooking um and stuff like that, like smaller household chores I wasn't outright expected to. Um whenever I did do it I did it kind of out of obligation because I felt bad or I wanted to or I felt like I wasn't contributing enough. Um but my only outright expectation was like childcare.

Moderator 43:43

Okay. And so, 2 and 12 so you said that you kind of just did this volition, but on your own volition without a lot of expectation. Could you just give me a little infor- more information about why you decided to just take on this extra work.

2 44:02

Uh um so, I I think I did it because otherwise it just wouldn't have gotten done. Um you know, my, my mom was just, I think it was easier for me to switch to online just because you know I'm I'm better with technology. My mom was like crying every day, having to um having to work from home. Um and I think if the, if I didn't do it, it just wouldn't have gotten done.

Moderator 44:33

Okay, and 12.

12 44:35

It was kind of the same situation with me like, whereas I was able to adjust to working online fairly easily, like both of my parents like had a rough transition and I could tell they still wanted to help out as much as they had done previously, but I could just kind of tell like if someone else who was available to step up to do it like didn't do it then sometimes things just wouldn't happen that needed to be done so I was just like, might as well be a nice daughter and try to just step up and do some things around the house, just to be nice I guess I don't know.

Moderator 45:13

Alright, and 4just a follow up question for you is that you said you might you, you might have felt a little bad. Could you tell me a little bit more about why you thought you might have felt that way? Oh, 4 are you still there?

4 45:39

Yes. Sorry, could you repeat the question?

Moderator 45:41

Yeah. So you said that for some of the other tasks you kind of took them on because you felt you might have felt bad. Could you just tell me a little bit more about that?

4 45:50

Yeah cause um I'm very like a per I'm a person who likes to help. I get a lot of like personal worth from helping others. Um just kind of who I am and so obviously like my parents are struggling and like everyone was struggling const- pretty in my household, like consistently in my household, and I was like hey you know I'm not expected to do this, but like, you know, I like what makes me so special to where like I'm not expected to help out you know if everyone else is doing their share I should be able to do my share as well. I don't know I come from a collectivist culture so there is kind of that expectation of, you know, everyone does stuff for the good of the group. So that might play into it. Um I just kind of wanted to help out, you know, wherever I could.

Moderator 46:32

Alright. So thank you all for sharing. Um, so we're gonna move on to our next question which is going to be about your academics and how they were impacted by the shutdown. So I'm going to ask you about how the imp- how the shutdown impacted a variety of things. If you don't think it impacted something, you can just say no impact. So to begin, um and we can start with 4 this time, are in what ways if any has the shutdown impacted your academic performance, like your grades and your understanding of the subject matter in your courses?

4 47:05

Of course um it impacted them pretty badly. Um I picked an awful semester to max out my hours. I took like 20 hours total, you know at my university and a community college so I was like oh I'm just going to grind through everything. Um obviously a terrible idea. Um and so my major um I'm an electronics engineering major. It's a very hands on um major each every single class that I have has a four hour lab associated with it. So on top of lectures I also had to attend these virtual labs. Um and so with these labs we have like these electronic kits that we're supposed to utilize. We usually worked in groups. My lab partner moved out of the state um after COVID hit and so for one of my classes we had like this little development board, and we had a final project where we had to use that to simulate like do an elevator simulation in person using LEDs. It was a very hands on project. And so the way that my lab partner and I split the project was that I had the dev kit and he had the lab kit so that you know when we came in person we'd each kind of have um something to bring together. Um and so my professor for some of my, some of my professors weren't very understanding. Um so my professor for this class was like, you need to have this project done. It has, I don't care you know whatever goes on. If you don't have this project done, you're going to fail. There's no way to do it for uh remote. Um if you have to mail stuff go ahead and do it. And so, um, and this was like very early on into um what was going on because, so like all like the post offices were um kind of closed or, you know, on limited uh functionality. So I drove out of the state to drop off my dev kit to my lab partner and we and it was, it was an awful experience. Um you know just the fact that my professor wasn't understanding and I didn't want to fail this class. Um so from that standpoint that was frustrating because you know I didn't want to retake this class. Um and then on top of that since my major is so hands on and I'm not getting that hands on experience because we don't have a lot of the lab equipment, you know at home like oscilloscopes and stuff like that um for my, you know, I have a class where it's just focused solely on um circuits and stuff like that and you need all of these heavy machinery to actually do them. I had to watch videos of people doing them which isn't the same one to one experience as doing it yourself. Um and I think next year I actually have to do my senior uh design which is building an electronic device from the ground up, and I needed that experience this past semester to actually be able to do that. So um while I like I ended up I was on track to get like you know almost all A's this semester. Um I ended up like you know getting still getting good grades you know I didn't do as badly as some of my friends had done, but it was still frustrating that like you know if this hadn't happened um I would have gotten you know all the experience that I would have gotten that hands on experience. I would have had like, you know, actually gained the information I needed to succeed in my next courses, and my GPA would have been, you know, my GPA would have risen, but you know it is what it is. Um it just definitely was a huge um I guess crippling impact on my education for me.

Moderator 50:17

Thank you so much. And 11, so in what ways did the shutdown impact your academic performance like your grades and your understanding of the subject matter?

11 50:26

Um my understanding was definitely was way harder to grasp concepts for me. I'm a person that I really have to try to learn things they don't just come naturally. Um, as far as grades go, my grades on tests definitely dropped but the thing was that everybody's grades on tests dropped. So our curve reflected that, so like my D on my calc test was a B plus. Um so, things like that. My GPA was fairly good and it probably would have been similar at school. But the curves were more dramatic because I think everybody was really struggling. Um so that, that was my and then I also had a lab that definitely was a struggle. For my chem lab, they would give us data and the data they gave us wouldn't make sense. My TAs didn't understand it. So we would just make up numbers because things didn't make sense. So my understanding of some concepts were definitely uh shifted negatively.

Moderator 51:31

Alright, and 12, what about you?

12 51:33

My experience is very similar to 11's just because, like the professors knew that like everyone was struggling at home so the curves were a lot better. But I will say like the situation with labs was very difficult for me, um because they weren't really working too well, they tried to do like online labs and those didn't really have the correct numbers they wanted. And then on top of that, I had research still going on that we couldn't complete because we weren't on campus. So it was a lot of like FaceTimes with the professor who is in charge of it and then we just had a lot of miscommunications in there, which ended up just confusing me with everything going on. And so I would say my understanding dropped but my grades were pretty much where they were originally going to be just because professors were anticipating that.

Moderator 52:26

Okay, and 2.

2 52:29

Um so I had probably the worst semester of my academic career. Um a little background, I have a huge gap um in my education because I actually dropped out of college when I was 20. Um I'm 36 now and I decided to go back to um get my degree. So, I don't remember chemistry. I don't remember physics. I don't remember like calculus so having to go back and not having that um you know I don't I don't remember stuff from 16 years ago. Um, so when I was on campus I was making sure to take advantage of the tutoring, to you know make sure uh all my professors knew me, you know making um using their office hours, and once we switched online I you know I didn't have access to any of that and it was much harder to um have them help fill in some of those things that I couldn't remember, um, and, you know, it is it's a community college so I think in the back of my head, I was just like, it doesn't matter what my grades are because you know like when I transfer it's not gonna you know it's not gonna matter. It's you know the school's, very affordable. Um and I, you know, the, my professors were very understanding, and I think like, if you just showed up if you if you had a pulse they were just passing everyone, but I felt like I really needed to know that information because it it will be something that I need in future classes. So I just decided, you know, so I am going to have to retake a lot of the class, a few of the classes from the spring semester now in the fall.

Moderator 54:09

Great, thank you all for sharing. So we're going to start with, 2 again. Sorry switching up the order, again. Um, so, in what ways if any has the shutdown impacted your career goals?

2 54:24

Um, you know, it definitely. You know, so I I have been working for you know for the past 16 years so having to, you know, what I thought I don't really know what what I want to do in life. Um so, you know, for the fall, I've changed my, you know I changed my major. I don't, I I think it's hard to see what the world's gonna look like in five years. Um and I, I've I'm so I don't really have a focus to begin with. Um so it's just added another like layer of complexity in terms of like, what, what do I want to do with my life.

Moderator 55:04

Alright, and 12. How has the shut down if in any way impacted your career goals?

12 55:10

Um so personally, I had an internship I was supposed to do this summer that unfortunately had to get cancelled because they couldn't have anyone come in person. So I would say that most drastically is what shifted my career goals and then I had to get like a part time job that ended up getting shut down too so I'd say in the long run like it just kind of just messed with the plan I had going for the long run and now I don't have like as much experience in the field as I would have liked to have but, you know, we'll, we'll get there eventually. We'll we'll figure it out but um yeah I would say having my internship shut down was probably the most drastic thing for that.

Moderator 55:53

Alright, and 11.

11 55:55

My career goals weren't um drastically impacted. But I do have to say that a positive thing it has emphasized my want to travel. I've always loved to travel and so I think I might try to incorporate that more into my career so.

Moderator 56:12

Alright thank you and 4.

4 56:13

Uh for me, my career goals were kind of not shifted but like I kind of started to think about other options. Um I've been interning with the same company since I started college. And so, um um I I'm still interning with them this summer but the internship got cut drastically. It went remote and they actually shifted my project away from something related to my major, um and they current company currently has a hiring freeze that they don't know when it's gonna end. Um so I'm trying to forecast and see. Um talk to like people at work and see if I can come back next summer and do an internship, and then after that, maybe see if I can go full time but I've started to realize if this doesn't get um the hiring freeze doesn't lift, or if I don't get that next internship. Um and if you know we do go into a recession where you know people aren't hiring my major, I've started kind of opening up you know, do I do grad school and if so like kind of preparing myself to do that even though I don't really have a desire to, but it just kind of seems like if I'm not going to be able to get a job when I graduate I might as well do something with my life. Um so I wouldn't have considered that before this all hit. Um so, it's shifted in that sense.

Moderator 57:27

Alright. And you've already. A few of you have mentioned this briefly in some of your other responses, but in what way if any has the shutdown impacted the availability of other learning opportunities such as internships? So 2.

2 57:42

Um, you know, I I wasn't exploring that at this point, um just because I don't really know know what I want to do. Um, you know it. I am slightly worried I'm much better in person, you know, so if going forward if if you know if there, if we are switching to, you know, a work from home model. I am worried about how I translate, you know, over zoom or over email you know like I'm, I'm much better face to face. Uh so I am trying to you know maybe work on those skills.

Moderator 58:22

And 12.

12 58:24

Um so I would have to agree with what um 4 had just said previously on the last question where, like, my field is very experience driven so if internships aren't going to be able to happen I have just been looking into maybe having to do grad school in the future just so I have something more on my resume. Um just because learning experiences aren't really happening that people would be looking for when hiring. Then I have to try to find that somewhere else, which is kind of difficult right now, with everything being online. So I'm just kind of hoping everything goes back to normal eventually, and things would work out.

Moderator 59:03

And 11.

11 59:04

Because I just finished my freshman year I'm in, I'm looking to major in ChemE but I'm not decided yet just because that's how my school works. Um so this upcoming year and looking into the future I'm hoping that internship opportunities happen and research opportunities happen but because I was only in Gen Ed's I don't really have professors in my department that I know and I'm worried about uh not being able to find a research program or an internship that I like. So I'm definitely more nervous about that, especially because I've never had an internship or done any sort of research or had lab experience, I don't, I don't really know where to start with all of that now.

Moderator 59:49

Alright, and 4. Oh, how could this affect the availability of internships?

4 59:56

Yes so this uh as I mentioned before, like I've been, this would this current summer is my third internship so I am grateful enough to have a plethora of experience however my past two were in like project and program management which isn't what I want to do and so this was my first going to be like my hardcore technical internship. Um and so my major is very hardware focused and obviously when things go remote you can't really do a hardware project. So I got put onto a software development project which has nothing to do with my major, so I'm not getting any good while I still am learning and I'm getting experience it's not related to what I want to do in the future. Um and so when I talk to my friends in my major, it seems to be like, you know, a pretty consistent thing where my friends who also want hardware based internships either got them cancelled or they went to like more software based internships, so I think for my field, the learning opportunities for hardware related experiences is drastically decreased as a result of this, but the amount of software based um experiences and ones that can be done remote obviously have increased a lot. Um and so, again, from what I'm forecasting from talking to people at work and stuff like that is that the plan is to have internships and keep it open, it's just the full time hiring that's been impacted, but I don't have to worry about that until the end of next year so I'm kind of just putting it off and just thinking about grad school as a backup. So.

Moderator 1:01:20

So, if if the answer is to this is just no impact you can just say no impact but did any new learning or career development opportunities emerge during the shutdown and if so what were they? So 2.

2 1:01:33

Uh no impact.

Moderator 1:01:35

12.

12 1:01:36

I would say no impact as well.

Moderator 1:01:38

11.

11 1:01:39

I did have one class, it might be miniscule. I took a project based, just like generic uh engineering intro class, and they had to clearly adapt it. It was based off of 3d printing, I don't have a 3d printer. So they ended up just having each student design their project and then they put together a website, so I can technically say that I'm like featured on a college website. I have a project that I designed that's featured so that's something that I could put on a resume that I wouldn't have had but you know it's not super drastic I guess.

Moderator 1:02:19

Okay. And 4.

4 1:02:22

Yeah, so like nothing super drastic. Um three courses that were usually always in person in my um major were made online so I'm catching up on schoolwork while I'm working so I'm taking three classes now. Um and then from a work standpoint, I'm like, you know, doing a software development so I have my project with my internship um and based off that um trying to see maybe if I can leverage that into an internship for next summer. Um and I've just kind of started doing like remote work, um kind of like freelance, you know, closed captioning in my free time as well, but nothing super huge.

Moderator 1:02:58

Okay. So, everyone so, um, in what ways has the shutdown impacted your confidence in your ability to successfully complete your degree at all or in a timely fashion. So, 2.

2 1:03:13

Um, you know, it's definitely because I'm I am going to have to retake some of the classes, it's going to extend it. Um I don't have a you know a normal um you know academic career to begin with, so I'm not, you know, I think it's less of a of an issue with my situation, um but it, it's, you know, I would like to to get this over with. And it you know it is extending it.

Moderator 1:03:42

Alright and 12.

12 1:03:45

Um, personally, I would say, I feel a lot less confident now with pursuing my degree because it is very lab driven and you need like immediate feedback from professors and stuff. I'm not getting that so much now that we're online. Um overall I think I could still figure it out and get my degree within the time I wanted to it's just I don't know if I feel like as great about it as I did previously before this had all happened.

Moderator 1:04:14

Ok and 11.

11 1:04:16

My confidence hasn't really shifted. Um I do have to say that if it continues into say my junior year of college, I might have to make some drastic changes just because out of state tuition's expensive to just be online and stuff like that. But if we are just online this fall and potentially some of my things in the spring, I don't think I will have shifted that much.

Moderator 1:04:40

Okay. And 4.

4 1:04:42

Yeah so similar to 12, my confidence has decreased quite a bit, especially with this more lab based um more of these lab based classes being moved online. Um but, um like I'm not super worried about finishing my degree on time because having more online classes, um lets me take some of the less important classes online and kind of get them over with. Um so I'm actually graduating a semester earlier than I expected since I'm taking these classes this summer. Um but in the fall, my university is actually opening up um and sort of the expectation from what I've heard from people in my department saying is that you're expected to be in person, and take these in person labs, so all my lectures are online I have to go in for, like all day Fridays and just do labs in person. Um so on the bright side I'm still getting that lab experience, but you know as someone who's immunocompromised I'm obviously kind of worried. Um and I'm just going to try and wait things out and see.

Moderator 1:05:42

Thank you everyone. So, in what ways did the campus shutdown impact your finances? So 2.

2 1:05:50

Um, you know, it didn't really, um, I I would say, not that much of an impact.

Moderator 1:05:57

Okay, and 12.

12 1:05:59

Um I would also say it wasn't a huge impact because I was already home for spring break, uh so I didn't have to worry about transportation too much, and they refunded us for like meal plans and housing and stuff that we weren't using so I would say, in a way, it almost helped me, because I got some money back versus it just making a bad situation.

Moderator 1:06:20

And 11.

11 1:06:21

Uh no real impact.

Moderator 1:06:23

And 4.

4 1:06:25

It was a pretty big impact for me. I pay for my own college. I live off campus so I still had to pay for a lease in a house that I wasn't living in. Um I had to take out more financial aid because I had to buy more lab parts because this university wasn't providing them. My internship when it got moved remote, I was, um they used to give us like a housing stipend, that I had planned some finances around and they revoked that. They cut my internship by a month so I was making like a third like two thirds of what I was expecting to make this summer. So I had to take out a significantly more amount of loans than I was expecting to.

Moderator 1:07:01

Alright thank you everyone. So in our next set of questions we're going to talk about what your professors did and did not do during the move to online learning. So what were some examples of strategies, tools, or technologies that your professors used that you found to be really effective in that they made things easier to learn during this online learning process? 2.

2 1:07:21

Um, so they would they would find modules from like that better schools were were putting up, and um I don't know if they had a licensing agreement or we were just using them like on the, on the sly. Uh, so you know I I thought that was inventive than instead of like you know reinventing the wheel. You know they were able to just, uh you know, take take these online modules that schools that had much more resources were able to develop.

Moderator 1:07:57

All right, and 12.

12 1:08:00

Personally I would say my specific school didn't do a great job in finding new tools to better our education like that. Um but the one thing I did find that was a positive change was I really like how most of my professors did pre recorded lectures. So whenever I was studying if I needed to be reminded of a concept I could just go back and rewatch that one lecture rather than having to try to look it all up on my own.

Moderator 1:08:24

Alright and 11.

11 1:08:25

Um my favorite thing that one of my professors did was my calc professor, uh and they worked with another professor in the--- that was also teaching the same class. They had on our Canvas page which is what we use for grades and stuff, they had set up a calendar that had office hours on it, and all you had to do is click the name, and it showed what time they were at. And it would just immediately put you on that zoom office hour and that was really beneficial. I also kind of enjoyed, this one's a little weirder, they had a map, and you could pin your location on the map and it just kind of showed where other students were and that was just kind of an interactive sort of fun positive thing. That was neat to see that I had, you know, there was another student from Pittsburgh and me having class in Minneapolis, it was interesting to see that there were, you know, more students in that class around me.

Moderator 1:09:16

Ok and 4.

4 1:09:18

Um I definitely loved uh when professors would not require cameras to be on during lecture. Um I also appreciated like you know when you're it's not just the cameras but like you know you don't have to attend lecture and they had them recorded, to where you can decide to join live if you'd like to but it's also available afterwards if you so prefer, just to kind of help with that flexibility. Um, let's see I also had a professor who essentially would do pre recorded lectures, um so you can watch this in your own time and you study those whenever, but he would be online. He would be on the zoom call anytime during lecture. You could hop in and do office hours that way, that was really helpful. Um also open note exams and non cumulative finals were extremely helpful especially given like there's a definitely a different dynamic to um online learning, at least in my case, and I felt like I benefited much more from open note exams than the ones where I'd have to like scramble to memorize things um for these you know online exams and stuff like that. Cuz it's like also I think the mindset is that you know people are going to cheat anyways if online stuff happens, might as well have people honestly get the answers and learn while they do it. Um if that makes sense.

Moderator 1:10:31

That does make sense. Thank you for sharing that. So on the flip side, what were some examples, or strategies of tools or technologies that your professors used to that you found to be really ineffective, such that they didn't really help you learn. So 2.

2 1:10:49

Um, you know, uh we had very, we had few pre recorded um stuff, a lot of it was live, and uh we were told we weren't allowed to record it ourselves even for like our own use. Um so I felt that was, you know, a a little difficult, because if you, if you had something conflicting, if the phone rang, um, you know you you were had to be glued to that zoom.

Moderator 1:11:20

And 12.

12 1:11:22

Personally my lab professors tried to use modules from like other schools they found online to try to do a replacement for our labs. For the most part, they were alright but they really didn't convey what they were trying to teach us at the time, and they had problems like glitching so people would have to redo labs frequently. So I just personally found that to be not very successful. And they're trying to fix things.

Moderator 1:11:50

And 11.

11 1:11:51

Our discussion rooms for like my calculus and my physics classes we did not talk when we were in breakout rooms, and that just was dramatically different than how it was on campus. Um, it was better in my physics class because we did have to have our microphones on and cameras on and that helped me honestly in those classes and the discussions. Um another thing that I didn't like, my calc professor decided to randomly switch the format of testing for our final, and they switched it to this website that we did our homework on that we have always done our homework on, but we switched it for a test, and there were lots of issues. The website kicked people out, made you resubmit your answers, you had different numbers, it took dramatically longer so there was definitely some issues with just how professors went about it.

Moderator 1:12:43

And 4.

4 1:12:44

Um I personally hated group projects. I had a lot of group projects this semester, weirdly, where it was like you had to like meet up outside of class and do PowerPoint presentations and collaborate, you know, and I think collaboration on PowerPoints especially is super difficult remotely. Not everyone's available, not everyone like you know is able to do that. Um and so it essentially turned into, you know, we're a group of six. Two of us are actually doing the work. And when it comes to present it's clunky you know you can't really like properly present it, um even when you do like audio um to where like you enter your own audio into each slide and you just play the slides, it was just really difficult and frustrating. Um and obviously I had professors who still expected us to do group projects with our hands on labs, which was ridiculous and it was a whole dumpster fire. Um so I think a lot of the collaboration, you know group work and like, you know, interaction between students needs to be revamped um from what my school had done.

Moderator 1:13:45

Okay. And so what were some things that your professors did during online learning that made you feel like they cared about their students, or that they didn't care about their students? So Nina I'm combining that question. 2.

2 1:14:03

Um, they would always begin the lectures by asking um how everyone's doing, um, and that they worked stuff-- uh I had a couple of professors that extended their office hours to um subjects that non academic subjects just it's like you needed someone to talk to, um or you needed to, you know, help processing something uh I you know, I thought that was very sweet of them to at least acknowledge that, you know, some people out there needed someone to talk to.

Moderator 1:14:37

Um I did want to say right now that it is two o'clock, but we started a little late. Um if you all can stay a few more minutes we just have a few more questions and I just wanted to finish up this question if that's okay. But I understand if you need to leave. Um. So 12 if you are able to stay, so what were some things that your professors, did that made you feel like they cared about you, or that they didn't care about you?

12 1:15:01

Um I'll say some of my professors during like their office hours they would immediately start just by asking you questions to kind of just make sure you were doing all right um and that you didn't need just help on like schoolwork, but that you were actually like okay in the environment you were in. And then some of my professors I kind of felt like they didn't care that much because they would never answer emails or get on their own office hours they just kind of would say things to make it sound like they actually were there to help you and then just not actually help you when it came time for that.

Moderator 1:15:33

And 11.

11 1:15:35

Um honestly, I felt like my TAs is cared more. I had a lot of understanding from them and they were really reaching out um and things like that. As far as professors go, some were better than others and it depended on the day. A negative thing I definitely had some professors after tests, say like, we expect more of you. We expected these things to be higher and stuff like that and it wasn't as understanding. But overall, I'd say most of my professors and TAs were very understanding and positive, and I do have to get going. So, thank you.

Moderator 1:16:08

Alright 11. I'll email you like three more questions if that's okay.

11 1:16:12

Yeah perfect.

Mod 1:16:13

Feel free to leave.

11 1:16:14

Okay.

Moderator 1:16:16

And 4

4 1:16:18

Uh things that I really liked that my professors did was obviously open note exams. I felt like when I'd had like open note exams I was more focused on learning you know what the material was and where to find it rather than trying to memorize it and it felt like it helped a lot more of my understanding of the material. Um I had like you know whenever I would like miss a homework or, you know, deadlines were obviously really frustrating to deal with because you know everything's really confusing. I had professors who would like you know let me submit things late without penalty um and not have an issue, you know. Um things that I hated. I just had really big problems with this one professor who just hated you know making any sort of accommodations for these online classes. The same one who expected me to drive the same one who made me drive out of state just to drop off a lab kit. Um he also like when we would do our exams he would have us he would record us like he would have us have two webcams set up, like we had to make two zoom accounts, one to show our screen and one to show that like our bodies while we were taking the exam, kind of like where our hands were at. And so he had it to where he would um he was like oh in the last five minutes of the exam you have to submit it. So if the class ends at 8:30 you have to have it submitted by 8:25 right but he wouldn't give us any sort of timing and no call outs or anything. Uh and he would take off 10 points per 30 seconds late. The thing was, and we don't have we can't look at we're not allowed to have like you know screens or anything besides like the one that we have where the exam is up. So, obviously people submitted it late. People got like you know 40 points off on an exam, because the timing stuff was weird and he had like really high expectations, and so you know that was awful. Um but besides that nothing else really screams out to me.

Moderator 1:18:02

Alright. Thank you, everyone, for sharing today. Um, I have three more questions but I'm going to go ahead and email you because we are right at an hour from the time that we started. Um, if you can just answer those whenever you're ready and then once you're done with those, and the survey, we can go ahead and email you your gift cards, but thank you all for coming today.

Low SES White Women Email Responses

1. What are some things that your university did (e.g., pass/fail options, townhalls, remote counseling) to help students be successful during online learning? Mention any resources the university provided or actions they took to help students be successful or feel supported.

   2. What do you wish your university did to better help students be successful during online learning?

   3. In general, are there any ways that your ability to be successful in school was affected by the pandemic that we have not discussed?

Survey ID 12

1. My university offered pass/fail for non-major related classes, so unfortunately I was not able to utilize this because all my classes were required for my major. There was really not anything else that they tried to do to aid us in our at-home education.

2. I wish that they had sent out information more frequently as we rarely heard from administration regarding the switch to online classes or plans for a return to campus.

3. No, I feel as if we have adequately discussed most of the obstacles that came with the switch to online during the pandemic.

Survey ID 4

1. My university provided a Pass/Fail option for all courses and in addition allowed us to Q-drop any course without it counting towards our limit of 4. This made courses a lot less stressful, as if we did not do as well as we would have hoped we could either drop the class or take a chance and see if we can pass it without impacting our GPA. Their decision to let us choose between options was helpful, as if we were doing well in a course, we could have our grade format stay the same and still increase our GPA if we wanted. We also had our Distance Education fees for the summer waived, these fees are about $500 per credit hour, and as a result, I was able to take a few distance education courses, that I otherwise couldn't have afforded. Other than that, we had online advising available, which was useful for planning out my degree plan when not on campus.

2. I wish my university refunded us for on-campus services that we couldn't use such as our gym and health center fees. I also wish more Asynchronous classes were available, so that those of us who might have to work in order to navigate the financial impact of COVID could keep up with our schoolwork much more easily.

3. I personally think that the social aspect of university learning is very important, and extremely negatively impacted by the virus. My friends in my major and I routinely had study sessions during the week to test out the concepts we learned in class and catch each other up on subjects that we struggled with. We tried doing it remotely, but it mostly got confusing and we all decided it would be better if we worked individually instead.

Survey ID 11

1) the option to switch to pass/fail later in the semester was helpful. It was also nice to be able to drop classes if needed. My university kept in contact which was nice, even though they were fairly vague.

2) I wish they were more straight forward and realistic in regards to the fall semester (not leading students on about the possibility for in person instruction for the fall).

3) the hardest thing for me was the lack of social interaction with people. I am very extroverted and it was really hard for me to be online mentally. I was okay academically, but it is a balance and my mental health suffered as a result of COVID and the lack of social interaction.

Survey ID 2

**Question 2:** For the spring semester, I wish that assignments didn't have such hard deadlines. There were some weeks where I had more time to dedicate to school, and it would have been nice to get a head start on my work had it been available to me. Other weeks though, I found it hard to manage my schoolwork and the unforeseen obstacles that the virus would present in my life. I have trouble with time management (I'll be the first one to admit that), but it was unreasonable to expect anyone to be able to predict their schedule this past spring.

**Question 3:** I am switching majors for the fall semester, so had the virus not been an issue, I would have gone to the new department already to introduce myself and try to make new connections. Theoretically, I could email people in the department, but I'm not a strong writer, and I do much better in face-to-face situations. I also feel that the virus affected my ability to succeed in school because I now have to consider my health when making decisions on how I will plan out the rest of my schooling. Because I am 36, I don't have the liberty of risking getting the virus and hoping that it won't have a long term effect on my health. If we don't have a readily available vaccine or therapeutic, and I'm no longer able to take virtual classes towards my degree, I would be extremely hesitant to continue my education. It's taken me over a decade to go back to school, and I sacrificed the lifestyle that I had because I knew how important a degree would be, but I can't risk my health to return to in-person classes.

SURVEY ID 1

Overview

The COVID-19 pandemic has presented big challenges for undergraduate students. As you know, universities across the nation have closed, moving all instruction online with very little time for students or faculty to prepare. Many students have encountered challenges related to the closing of campuses, relocation, and the movement to online instruction. In this study we want to learn how the closing of campus and the pandemic in general has affected you. We want your opinions about the good and bad, on how things went as well or didn’t, what your professors and universities did that was helpful and unhelpful, and what you wish they would have done.

**Let’s start by discussing where you went when your campus shut down. I’d like to hear from each of you.**

First, I’d like you to tell me about the space in the home you moved to. What space did have to do your school work? Was this space private or shared? If shared, who you shared it with.

I moved out of my dorm room in early March and moved in with my boyfriend’s family in REDACTED. My family moved to REDACTED the summer before. My boyfriend’s family has a large camper that we used as a makeshift library, so it was considered private. I shared the space with my boyfriend who was also taking classes at the time.

What challenges did you encounter attending classes or doing your schoolwork in this space? Could you tell me about why this was challenging for you?

We had a difficult time with WiFi connection due to spottiness in the area and bad weather (wind, especially). Occasionally we would have to move inside due to this disruption. Similarly, extremely hot weather pushed us inside because the AC could only do so much. It was difficult to focus on schoolwork when we needed to relocate throughout the day.

What are things that you, or other people in your house, did that made it easier for you to attend classes and do your school work?

My boyfriend’s parents allowing us to use the camper was a huge help and gave us a quiet, private space to attend lecture and take exams. For the most part, we were left alone throughout the day so we weren’t disturbed.

If you live with siblings, to what extent were there differences in how much parents supported each of you in your ability to attend classes and complete schoolwork? For example, were some kids in the house given more time, space, and consideration to do school by parents than others? Could you tell me more about why you think it was alike or different?

I was not around my siblings, but my boyfriend has a younger brother that finished his junior year in quarantine. He preferred to work in his room, so no one had priority to study space. My boyfriend’s parents didn’t have any control over our responsibilities (didn’t remind about deadlines or asked about assignment completion, etc) and instead managed the brother and his work.

**In our next set of questions, let’s talk about what other responsibilities you had *after* your campus closed and how these differed from responsibilities you had *before* campus closed.**

Let’s start with care-taking responsibilities. Could you please describe your care-taking responsibilities? This can include childcare, eldercare, or caring for ill loved ones. Please describe how your care-taking responsibilities after the shut-down were alike or different from your responsibilities before the shutdown.

I did not have any care-taking responsibilities before or after shutdown.

Now let’s talk about house-hold responsibilities and how they changed before and after the shut-down. Please describe your other household responsibilities such as chores, cooking, caring for pets during this time and how they were alike or different from before school closed.

Since I moved from my dorm, my house-hold responsibilities increased. I helped his family with chores (dishwashing, maintaining the pool, errands), occasionally helped with cooking, and mostly cared for his two dogs. I did not have these responsibilities pre-shutdown.

After the shut-down, to what extent were you expected to take on more household work, childcare, and/or family caregiving responsibilities than other people in your home? For example, parents may expect more of one sibling, or partners and/or roommates might not contribute equally.

Since I was a guest I was not expected to do household work. I did my best to be a respectful guest in their home by doing little tasks and made an effort to make shutdown easier on everyone.

**Thank you all for sharing. Now let’s talk about how your academics were impacted by the shutdown. I will ask you how the shutdown has impacted a variety of things. If you don’t think it impacted something I ask you about just say “no impact.”**

In what ways if any has the shutdown impacted your academic performance (like your grades) and your understanding of the subject matter of your courses?

It had a largely negative impact on my understanding of material, but had no impact on my grade performance. Courses were more lenient with work and I was able to get by with what I could do.

In what ways, if any, has the shutdown impacted your career goals? (follow-up: could you tell me a little bit more about why they have changed?)

I had already been considering a major change from biology to communications, and shutdown gave me more confidence in completing that change. It is something I am more interested in and would grant me more flexibility if the pandemic continues for more than a year.

In what ways if any has the shutdown impacted the availability of other learning opportunities such as internships?

No impact, I wasn’t actively looking pre-lockdown.

Did new learning and/or career development opportunities emerge during the shutdown and if so, what were they?

I discovered free certification courses related to my field of study that I am currently taking.

In what ways if any has the shutdown impacted your confidence in your ability to successfully complete your bachelor’s degree at all or in a timely fashion?

I have lost confidence that I will complete it as strongly as I would have without shutdown. I feel that I have lost networking opportunities with classmates and professors, job experience on-campus, and other organizational involvement with volunteering that would have benefitted my resume.

In what ways if any did the campus shut down impact your finances?

I was removed from my housing contract at the end of my freshman year and the beginning of my sophomore year. I will be saving money on housing my living at home (I flew home the beginning of June).

**In our next set of questions, let’s talk about what your professors did and did not do during the move to online learning.**

What are some examples of strategies, tools, or technologies that your professors used that you found to be very effective (in that they made it easier to learn) during online learning?

They opened discussion spaces via Nectir or Gauchospace that students and professors could use. Also, having lectures recorded made it easier to study material.

What are some examples of strategies, tools, or technologies that your professors used that you found to be ineffective (in that they did not help you learn) during online learning?

My CHEM professor implemented free response on exams (something he previously did not do) in order to combat cheating. He did not alter the time allotted to take the test. I feel he made the tests way more difficult than in-person and grades suffered.

What are some things your professors did during online learning that made you feel like they cared about their students?

All professors emailed updates and told students to let them know if they had anything going on. They were honest if they were struggling as well. Assignments were extremely flexible. They were accommodating for protests as well.

What are some things your professors did during online learning that made you feel like they didn’t care about their students?

I did not have an experience like this. All of my professors were great.

**In our final set of questions, we would like to know more about your university. Let’s talk about how your university responded to the move to online learning.**

What are some things that your university did (e.g., pass/fail options, townhalls, remote counseling) to help students be successful during online learning? Mention any resources the university provided or actions they took to help students be successful or feel supported.

My university held townhalls on COVID and COVID responses regularly. They shifted academic counseling online for students. They made changes to the online course system to accommodate lecture uploads, etc.

What do you wish your university did to better help students be successful during online learning?

My university made pass/fail optional for departments. I wish that wasn’t the case because a lot of students struggled in classes that didn’t allow them to.

In general, are there any ways that your ability to be successful in school was affected by the pandemic that we have not discussed?

N/A

**That is the end of my questions. Thank you for participating in this focus group.**

High SES White Women

Moderator 0:47

Hi everyone. If you feel comfortable, you can go ahead and turn on your camera we're going to go ahead and get started. Okay. So, everyone. Welcome to the focus group. My name is Moderator. I'm a research coordinator from Redacted. Today our note taker will be Moderator, you can see her screen over there in the corner. So today we're going to be discussing a number of open ended questions. There are no wrong answers, but people may have differing points of view, so feel free to share your point of view, even if it's different from someone else's. On the other hand, some of you may have really similar points of view. If your experience is similar to someone else you can just say my answer is the same as Sam's. So keep in mind here that we're interested in negative comments, just as much as positive comments so we encourage everyone to share whatever is on their mind today, but because we have to keep this to an hour I may have to cut you off so I apologize in advance. If I need you to finish up your thoughts so we can move on to someone else I'm just going to raise my hand like this. It's difficult for us to take notes on everything that's happening today so we're going to be recording this for transcription later. Um we won't be associating your name with anything you say today in the focus group. In addition, you may refuse to answer any question or withdraw from the group at any time. In addition, we will ask participants to respect each other's confidentiality and not repeat things outside of the group today. Are there any questions before we get started? All right, so then let's just go around and say, each person say your name, what year you are in college, and one word to describe what life has been like in the pandemic. So, I see 22.

22 2:47

Um yeah hi my name is 22. Um, I forgot what else we were supposed to say just now, I'm sorry.

Moderator 2:54

Yeah, so your your your name, year in college. And,

22 2:59

Um, just starting my junior year in the fall. And I guess complicated would be the word I would say.

Moderator 3:08

Okay. And so, 14.

14 3:15

Um I am going to be a sophomore. Or I guess I technically am now. Um and the word I would use is exhausting.

Moderator 3:28

Okay. And, 29, or is it 29?

29 3:33

It's 29. Um yes, I'm 29. I'll be a junior in the fall and I think the word I would use is mixed, because there's a lot of a lot of positives and a lot of negatives.

Moderator 3:45

Okay, and 24.

24 3:55

Can you guys hear me?

Moderator 3:57

Yeah.

24 3:57

Oh okay uh I'm 24. Um I'm a sophomore and I would probably describe it as difficult.

Moderator 4:07

Okay, so thank you all for sharing. Let's go ahead and move to the focus group discussion. So the COVID-19 pandemic has presented big challenges for undergraduate students. As you all know, uh universities across the nation have closed moving all instruction online with very little time for students or faculty to prepare. Um most um most many students have encountered challenges related to the closing of campuses, relocation, and the movement to online instruction. So in this study, we want to learn about the closing of campus, and how the pandemic in general has affected you. So we want your opinions on the good and the bad, what thing things that went well or didn't go well, and what your professors would have you wish they would have done, and things that your universities you wish they would have that that you also wish they would have done. So let's start the discussion today by discussing where you went when your campus shut down. So I'd like to hear from each of you. So let's start with 22. So I'd like you to tell me about the space in the home that you moved to. So what space did you have to do your schoolwork and was this space private or shared?

22 5:16

Um so before the pandemic I was living in an off campus house um with other roommates. I have three other roommates. Um, and I'm from the area too, that I went to school at so when the school shut down I just stayed there. Um, and then my workspace was a little bit difficult for me because I have, I have just a small desk in my room and there's not that much space, and especially because teachers sometimes want to see like your hands and the paper and the desk space like while you're taking a test and have your camera on so it was kind of hard to do that sometimes. And then um we had one other shared space that I would do work at, so it was kind of trying to go back and forth between that to keep some variety in like, where I was and what I was doing.

Moderator 6:05

Okay, and 14.

14 6:09

Um, so I just went back to like my house. I moved out of the dorms. And um I pretty much do all my work in my bedroom now, which is pretty small, but uh nobody else really comes in, so. Yeah, I don't know. It's pretty much the same as what my dorm was like.

Moderator 6:35

Alright so pretty private.

14 6:39

Yeah.

Moderator 6:41

Okay and 29. What about you?

29 6:45

Yeah, so, I was actually living at home, and commuting before um the pandemic hit. So, I am living yeah at the same space, which um is nice because I have my own bedroom, and it's pretty big, so I had like a, I can have a designated work area in addition to like where I sleep and stuff so yeah.

Moderator 7:12

Okay and 14, or.

14 7:23

Oh, yeah, I already went um.

Moderator 7:26

24, sorry my screen is moving around and 24.

24 7:34

Sorry uh, so I was, um, I had to go back home, so I went back home and I have like I have my desk, um, but like I had to like tell my other family if I was going to be taking an exam on my computer or something. And so, because we don't have really good Wi Fi so we would just be like, hey, I'm going to use the computer right now so, can everyone hop off unless, so we kind of took turns like taking exams, like that because I have a sister too so she was doing her work too so that's kind of the situation we were at.

Moderator 8:07

Okay. And so what challenges did you have attending classes or doing schoolwork in this space? Start 22 again.

22 8:16

Um I think I already touched on it a little bit but um just that it was a little small for me. Um, and also not having um I guess like a designated space. It was more like I would use that space for other things too. And it was hard to, I guess, assign like work time for me or like school time because it was all like merged in one area. Um, so it was harder for me to be productive, I think. And that's, yeah, that's pretty much it.

Moderator 8:49

Okay. And I'm going to keep moving by the order of who appears on my screen so 24.

24 8:55

Um, I had I had the same troubles as 22. Um, it was kind of, it was, it was harder to stay productive, I would say, um, especially because my timing was off like now like home life, school, everything was at home. So, it was just hard to like set timing and like figure out a good schedule that worked for me because I'm a really routined person. So what I ended up started doing is actually like making a daily routine inside of my house and um that ended up working but it was just hard because I never saw professors, I had to email a lot of questions, I had technical difficulties and so uh it was definitely frustrating to figure out.

Moderator 9:38

Okay, and 14.

14 9:41

Yeah, I feel like that too where it's, it's hard to do like all your schoolwork, but then the rest of your like, activities, all in one room. And it just kind of starts to all blend together. Um, but other than that, I think it's also kind of difficult because I don't have a desk in my bedroom and I did in my dorm. So I kind of just sit on the floor all day. But um, yeah that's that's pretty much it.

Moderator 10:17

Okay, and 29, what about you?

29 10:20

Yeah, so I think the challenge for me was like, get like using my designated areas to study because like I had like tables and I had a desk, but I would just mainly end up doing my work and watching my lectures in my bed, which is not like good for me like posture wise at all.

Moderator 10:40

Okay, so what are some things that you or other people in your house did that made it easier for you to attend classes and do your schoolwork? So 22.

22 10:52

I mean, first we would all we have a group chat with my roommates, and we would just let each other know if there was a test that we like didn't want people to be too loud or anything like that, just be respectful of um what other people were doing. Um, that's pretty much it. We kind of did our own thing so we each just did our work at home and didn't really help each other out that much, I don't think, besides letting each other know.

Moderator 11:24

Okay, and 24.

24 11:27

Uh, my family was really supportive about it. Uh like so I would be in my room watching my lectures, and I was like really scared to miss anything because um I'm very visual person. I like to go to ask questions so especially like I did calculus online and that was by far really challenging for me. Um, and so my family was really supportive. They were actually like, hey, if you like, need anything text one of us and we'll bring it to you. Um, and everyone was like super quiet. We all like shut our doors. Um and so we just didn't and we didn't like barge in on people like we knew like we would be on like a scheduled call and stuff so they were always really supportive.

Moderator 12:10

Okay, and 14.

14 12:13

Um I just told my parents like hey these are the days I have my classes and what times the lectures were going to be and so then they wouldn't like come in and disturb me. And that was pretty much it.

Moderator 12:29

Okay, and 29.

29 12:32

Yeah, so I told my family when I had my um tests, and then they would be made sure that they're being super quiet, and then leave me like the kitchen so I had like the kitchen table to spread out all my different notes, and things for the test and be like really close to the printer so I could print my test when it got uploaded. So, yeah.

Moderator 12:56

Okay. And so I know that this isn't relevant for some of you so if it's not just say, this doesn't apply to me. But if you lived with siblings, to what extent were there differences in how much your parents supported each of you in your ability to attend classes and complete schoolwork? So, 22.

22 13:15

Yeah it doesn't apply to me.

Moderator 13:17

Okay, 24.

24 13:19

Uh so my sister was finishing up her senior year in high school. So I think that they weren't as like, you have to get it done just because I don't think that they were taking grades anymore if I recall. So my parents were just kind of like eh like you don't really have to do it, you should. Um, whereas like they knew my schedule and if I didn't attend class they'd be like, what were you doing, why'd you sleep in or something like that. So, um, I think it was just because it was her senior year and she was missing out on all those like fun events that you get for senior year. Um so that was kind of like our difference if that's what you were asking.

Moderator 13:58

Yeah.

24 13:59

Okay.

Moderator 14:00

And so, 14.

14 14:03

Yeah, also doesn't apply to me.

Moderator 14:06

Okay, 29.

29 14:08

Yeah, so I had a brother who was a sophomore in high school and I think my parents were definitely like equally supportive of us, but I think for me like they gave me like ownership of my schedule and like what work I was doing. Like they didn't really care, and didn't really, I mean they could like support me doing it but they weren't like on my case or anything because they knew like I like normally I'm responsible for my own schedule and doing my schoolwork, but I think for my brother like they're very much like you have to get up and go to class and like do you know you have class in an hour like kind of keeping him on schedule.

Moderator 14:42

So you think that, um, that there were some differences because of just personalities between you two or is was there something else? Could you just tell me a little bit more about that?

29 14:55

Yeah, I think, maybe like more personalities. I mean, or I think it actually is more like age, like, I'm in college like they're not don't have like any oversight of my schoolwork, like when I'm at when I'm normally doing college so I think they just felt like they didn't have to take any oversight when I was at home all the time, and like my brother's in high school so they still like make sure that he gets his homework done when he's like in normally goes to school so they they had to do that when he was like at home doing online school as well.

Moderator 15:28

Okay, so in our next set of questions we just want to talk about some responsibilities that you had after your campus closed and how those differed from responsibilities you had before your campus closed. So we're gonna start with caretaking responsibilities. If this is something that wasn't relevant to you, you can just say, this isn't relevant to me, but I just would like everyone to describe caretaking responsibilities that they had um before and after the shutdown and how these things were alike and different. These can be things like child care, elder care, or caring for ill loved ones. So, 22 could you tell me about your caretaking responsibilities and how they might have changed before and after.

22 16:05

Um, I mean sometimes I would like help my family out and watch my siblings and I think once the pandemic happened um that was less often because, I mean my parents were home too but also because um my family didn't want, like I wasn't around um before that much so to have me back involved um when I could be infected or they could be infected they just didn't want um us interacting as much, I guess. So, less um responsibility.

Moderator 16:43

Okay, and 24.

24 16:46

Um, so I don't, I don't have like younger siblings or anything. I have younger cousins, but in that time we actually quarantined from like all of our family, like we like completely cut off contact and we just literally stayed inside. But, like, would other responsibilities like as far as like upkeeping an apartment count or not?

Moderator 17:06

Yeah we'll get to household chores in a minute.

24 17:09

Okay. Like doesn't take care of other people.

Moderator 17:11

Yeah. So 14.

14 17:17

Yeah, um I don't think that one applies to me.

Moderator 17:21

Okay, and 29.

29 17:23

Yeah, I don't think it applies to me either.

Moderator 17:27

Okay, so now we can talk about household responsibilities and how they might have changed before and after the shutdown. So if you can just describe your household responsibilities, such as chores, cooking, or caring for pets, and how this might have changed before and after. Um, 22.

22 17:44

Um I have a dog so it was nice to be at the house more because I wouldn't have to before with my schedule I'd have to kind of plan time so I could come home and let her out and feed her and things like that. Um, but now it was more like more flexible with when I could do those things and how much time I had to be with her. Um, and then in terms of chores, I was kind of doing a lot of household chores before and after so that didn't change. Um, I probably cooked a lot more though, being home. Compared to not.

Moderator 18:19

Okay, and 24.

24 18:22

Um, so before like the whole like we had to go to, like, home and online stuff, I was living in an apartment on campus. So I always had to like deal with that. So, I mean, obviously, you do your own dishes and stuff like that. Um but like there was also like a lot of like the apartment, but there's a lot of leaks and like just the issues that I had to get fixed so like I was constantly stressing about that. So it was actually kind of nice because I was able to get out of my lease so like it kind of hit perfect timing. Um, but then when I got home it was, it also, it kind of helps because I can do laundry and dishes and chores around the house and not necessarily like keep up with the whole apartment so that was nice. Umm prior to having COVID, um, or not having it but being in it, um, my cat was at home and my sister was taking care of her and then when I came back, um I started taking care of my cat again so yeah.

Moderator 19:18

Okay, so your household responsibilities, decreased when you moved back home.

24 19:22

Yes.

Moderator 19:24

Okay. Um, 14.

14 19:28

Um, yeah, I would definitely say that I had a lot more household responsibilities in my dorm. And when I came back like you know I didn't really ever cook as much or do any of that. And um I also I take care of my cat now cuz she was at home but overall it's definitely decreased.

Moderator 19:52

Okay and 29.

29 19:54

So I'm usually pretty busy during the school year, like really, busy so I don't normally do, like any household chores, which is really nice my parents to do everything. And so, that didn't really change like once COVID hit, because I didn't really still didn't really do much, so.

Moderator 20:19

Okay, so now let's talk about to what extent were you expected or not expected to take on household work, childcare, or caregiving responsibilities in your home. And this can be um things like parents or roommates, you know whoever you live with. Um, so, 22, can we start with you? So to what extent were you expected to do things that you did in your in terms of your household chores?

22 20:45

Um, I mean I think I'm kind of it I don't think it changed that much. I was expected to do the things that I was doing before and after. Um yeah.

Moderator 20:59

Okay, and 24.

24 21:02

Uh, mine was just like my usual home chores like just dishes, uh laundry. Like everyone does their own laundry and like vacuuming once a week, stuff like that. Um so that wasn't like that's expected of me, but when COVID hit um they were like a little more lenient about it like, if, if my parents saw that I was in my room studying like all day then somebody else would pick it up, but I mean obviously if I was watching TV they'd be like, alright, come do it. Um, but they were really nice and just like lenient and they were willing to be flexible with those things

Moderator 21:38

Okay and 14.

14 21:41

Um, I don't really think my expectations changed that much. Um, yeah I would pretty much say they they stayed the same.

Moderator 21:52

So, um, what about the expectations of people around you in your household? Do you think that they had expectations of you to take on different chores in your house?

14 22:03

Um, no. I think, like, you know, before I left for college and, uh like, now that I'm back, it was pretty much the same stuff that I would be doing. And like obviously I had more and more expectations when I had roommates and like, you know, we all live together, but then in terms of like in my house, the expectations are the same.

Moderator 22:34

Okay, and 29.

29 22:38

Yeah, I don't think there's any pressure or ex increased expectations.

Moderator 22:46

Okay. And could you tell me a little bit more about that is that because the other people around you are doing those tasks?

29 22:53

Yeah so I think they had been doing them, so they just kept doing them.

Moderator 23:00

Okay. All right, so thank you all for sharing. Um so now let's talk about your academics and how they were impacted by the shutdown. So I'm going to ask you a few things and if you don't think it impacted you you can just say no impact. So in what ways if any has the shutdown impacted your academic performance, like your grades, and your understanding of the subject matter in your courses. So, 22.

22 23:26

Um I think it did for one class in particular, um, and I think that's just because of how the transition went for that class. I don't think that it was um handled very well. And, yeah, the way the way that that class did it was bad. I don't think that it's necessarily like in general I did worse. I would said, I did the same um before and after academically. But, um, because of the pandemic one class in particular was worse than it should have been I think.

Moderator 24:01

Okay and could you just say a little bit more about like what particularly was it about the pandemic so um?

22 24:08

Um just transitioning to online classes. Um the instructor wasn't really prepared for that and the way they did it um made us I don't know if we're going to go more into that later, but I can wait.

Moderator 24:21

Yeah we will. So. And 24.

24 24:26

Uh, so it actually impacted me a lot. Like the semester prior to COVID impacting us um I was like, doing really well in school. Um, I took 16 hours that semester, then this semester of COVID I took 17 and it was just really difficult like doing everything online. That's why I'm really nervous for this next semester, because, like all my classes are online, but during COVID, um, I didn't feel like the pro, I mean clearly the professors weren't um equipped to teach online, which I understood that, but like my biology professor he like he peaced out. He said, like, alright, you have a test on this day this day. Um everything you need is on canvas and I would look and I'm like what? So I would email him and he'd get back to me but it sucks because he was quitting his job that same that same semester. So he kind of was just like, I'm gonna quit early so um I actually barely got to be in that class and then calculus was the professor was really well prepared and really gave us the information we needed to succeed but Iike I just did not succeed with that online schooling. Um it was just, it was difficult because I would always go see him personally in office hours and work out problems on the whiteboard with him and like just really put in time and effort and I still tried to but uh there's just there's something different between online and in person and being able to go to those office hours and seeing, you know, a TA, your professor. So, uh I would say overall it negatively impacted my performance. My GPA went down. So, yeah.

Moderator 26:10

All right and 14, what about you?

14 26:14

I think that I got um you know pretty much similar grades that I would have if uh we hadn't had to go online, but I do feel like my understanding, in my classes wasn't as great since they had to be taught online. Um but my grades pretty much stayed the same.

Moderator 26:43

Okay. And 29. Could you tell me about how the shutdown impacted your academic performance? Your underst-

29 26:51

Yeah, I think that it actually um increased my performance in I think every class. Um just because I was able to use notes on my exams then. And so a lot, because I was in pretty a pretty like STEM heavy load and a lot of those classes like the tests, are just like memorizing your notes and that's kind of the hardest part of it, and then if you don't have a lot of time then you're kind of it's hard, a lot harder. So just like being able to reference my notes on the exams I think was a huge help. And then that ended up yeah bringing all my grades up, which was really nice.

Moderator 27:37

Okay. And so, what ways if any has the shutdown impacted your career goals? So, 22.

22 27:46

Um, before the shutdown I applied to maybe like 15 internships over the summer, um and put a lot of effort into, like, writing applications and putting my best foot forward I guess, really hoping that I was going to get something, and then most of them um didn't offer the internships anymore, so I wasn't able to do that. Um I just spent my time, um more focused on my current job before that, and then also trying to get some things started for the fall.

Moderator 28:16

Okay, and 24.

24 28:20

Um, as far as like after school ended, uh, it kind of gave me like an opportunity to actually have more time. I kind of I thought it was funny because you know everyone's always asking like I need more time I need more time and then we were. I think gifted we were gifted cursed in a way with all this time. And it was kind of like, what are you going to do with it? So like not everyone was comfortable getting out. Um, but like I started, I started a job at an orthopedic clinic which I want to go into orthopedics so uh they needed people to help out so it kind of benefited me in a way because it was like, easy to get in. So uh it helped me in that sense, but as far as like wanting to pursue my career, it hasn't changed.

Moderator 29:05

Okay, and 14.

14 29:10

Um, yeah. It it hasn't really changed anything about, you know, like long term career goals. But, um, I haven't like applied for a job or internships or anything so it didn't have a direct impact in that way.

Moderator 29:28

Okay and 29.

29 29:31

Yeah, so I had originally a in person internship for this summer and it got moved to become a virtual from home internship. And so, I don't think my career goals were really affected and I think like all the benefits I would have got in person. The company was really nice about make like finding ways to do that virtually, so.

Moderator 29:57

Okay, and so 29 got at this a little bit, um and some others have mentioned some internships, but now I want to know more about how the shutdown impacted the availability of other learning opportunities so it can be things like internships, but just any other learning opportunities. So 22.

22 30:16

Um so yeah, it went down with internships, but also, um, there was this thing I was interested in um that my current boss showed me, um and it was like a word vector analysis course, I guess, and um traditionally you'd have to pay for that and it's an in person thing, but because of COVID they were offering it online. So I've also been doing that. Um so I guess in that way I've been giving like a free um learning opportunity, because of it.

Moderator 30:45

Okay, and 24.

24 30:48

Um, so it kind of like helped me in a way like increase, like my opportunities. Um, I didn't know that it would be an opportunity. I'm kind of that person just like, oh, ask and if they say no, they say no, but I figured, you know, I wanted to go shadow in this clinic and I was like, surely they surely they need help, like, surely someone's out with COVID and they need help, so uh I ended up getting in and I actually got a job there which I didn't think would be possible but it happened so um I'm really grateful for that. So yeah.

Moderator 31:23

Okay, and 14.

14 31:27

I definitely think that it's had a negative impact on just learning opportunities in general. Uh even though I don't have like a job or an internship, also just like learning from like professionally related club meetings, or just even go into labs for your classes, it's kind of like, yeah, put a stop to that kind of thing.

Moderator 31:59

Okay, and 29.

29 32:01

Yeah, so as I mentioned before, I have my virt-, my internship got moved virtually so I don't think like I've seen a negative effect in that area, and my company has been really good about offering like virtual networking opportunities and kind of different speaker series and different like online tools you can use to like build skills. So I think I haven't experienced any negative effects.

Moderator 32:29

Okay, um it looks like we just lost 24 but we're gonna keep moving. I think she was having some technical difficulties as well in the beginning. So, um 22. So, um, you might have touched base on this a little bit before with your comment but just to get a little more in depth. So like, what were any new learning opportunities or career development opportunities that emerged during the shutdown and what were they and can you just tell a little about bit about why they emerged?

22 33:00

Um so, yeah, it was a word vector um analysis course I guess. I'm not sure who it's through. I'd have to look it up. But um I think that they were just trying to make it more available and give something um to do for people. And, yeah, they just put it on their website. I don't really know that much about it other than that, but it's been interesting and a nice experience to learn from.

Moderator 33:28

Okay, and 14 you said that it kind of negatively impacted um, but were there any things new that happened?

14 33:39

Um, I don't think so. I don't, I can't think of anything.

Moderator 33:45

And what about you 29?

29 33:48

I can't think of any either.

Moderator 33:52

Okay. All right. Um, so in what ways if any has the shutdown impacted your confidence in your ability to get or to successfully complete your bachelor's degree in a timely fashion? So 22.

22 34:10

Um the same. I so I started off as a different major and then I switched. Um so already I was kind of thinking that I'd have to add on, like a semester or something. But I've been taking classes over the summer, that are offered online now so maybe it's even helped because now I think that I have more time to be able to take those classes over the summer, um and now I'm planning on graduating like within the four years so.

Moderator 34:36

Can you tell me a little. Oh sorry.

22 34:38

No, you're good.

Moderator 34:40

A little bit more about your the time that you have this summer to take more courses. So what is giving you more time?

22 34:48

Um, well I guess I don't have to go in person to work. I do work online. Um, so that's one thing, and also the fact that I don't have like an internship to go to over the summer, that's another thing. I was kind of expecting that. Um, so just less um responsibilities or work in other ways that I can now focus and apply my time to the school.

Moderator 35:14

Okay. And, oh hi 24. You're back.

24 35:18

Sorry, it cut out.

Moderator 35:20

You're fine. Um, so we're just talking about like in what ways if any has the shutdown impacted your ability to successfully complete your bachelor's degree at all or in a timely fashion.

24 35:35

Uh, it hasn't affected me. Um, I'm still I'm still, I still did like I didn't drop any classes so I'm still like on court on track right now and I plan to take uh the classes I need. I am gonna like, I'm not gonna take as many hours next semester with going online but um I'll still be on track. So it it hasn't affected me.

Moderator 36:02

Okay, and 14.

14 36:05

Yeah I would say it hasn't affected me either. Um I didn't drop or withdraw from any classes, and I already kind of knew which ones I was going to take this year beforehand, and I'm still like taking and registered for them. So, I think it's the same.

Moderator 36:25

Okay and 29.

29 36:28

Yeah, I think it's increased like my, my confidence because both this semester before COVID I was taking 20 credits, and I had like a a TA position and I was also a student athlete. So I was balancing all those things. And then um so like right but I remember like the week last week of school, school we had like right before COVID, I was just like not getting any sleep because I was trying to do all these things at once. And so, I think, but then like once we went online and we had, I had and I didn't have practices, I just had a lot more time. I think then I was able to then bring up my grades, and, um as well well as other things that contributed to that but also just like then end the semester on a really high note, and feel more confident like moving forward.

Moderator 37:23

Okay. And so my next question is, is in what ways, if any, did the campus shutdown impact your finances? So, 22.

22 37:35

Um it didn't really affect me. I got less hours at work initially but my parents helped me out a little bit so it yeah. It didn't impact me that much.

Moderator 37:45

Okay, and 24.

24 37:46

Uh I got less hours at work too. I was working on campus, as a fitness instructor. Um so I didn't get to after the gym shut down I didn't get to do any of those uh teach any of those classes. But then I also moved off campus and so um. I was able to break my lease for the apartment, but it was still there was still money wasted there so um it like there was a lot of money that was just wasted so that was kind of disappointing. Um but as far as like other finances, it hasn't affected. Oh, we actually got we we got refunded some money for like parking passes and one other thing but I forgot what it was.

Moderator 38:28

Could you tell me uh just maybe in a few words, how negative your impact was?

24 38:36

Uh it was disappointing to lose out on money. That's a few words.

Moderator 38:45

Um, so 14. How did the campus impact shutdown impact your finances?

14 38:53

Um, it didn't really have any impact like when it first shut down um and after I moved home they they refunded money for housing, and stuff like that. But um, I got an off campus lease for the fall semester and like you can't get out of it. So um if I don't go back there in the fall, which it looks likely that I'm not, I'm still gonna have to pay it which is unfortunate but I do have scholarship money that I can use for it, so that's that's pretty much all the effects it's had on me.

Moderator 39:37

Okay, so sort of um in a similar vein as what I asked 24, so could you describe a little bit just how negative for you or how worried you are about um that contract in the fall. 14.

14 39:51

Um I would say it's just it's just frustrating pretty much. I don't know.

Moderator 40:03

Okay and Ana, what about you?

29 40:07

Yeah, so I definitely didn't suffer like a significant negative effect. Um, so yeah we got because we got like a little bit of a refund for like services that we weren't using on campus. And I think I saved a lot more money like not being able to go anywhere, and spend any money. And then I like had this, I was already TAing for online class before um everything happened so that was the same hours of work. And then I think the only thing is like I used to get free dinners and free snacks from the um our athletes village. So I think I had to spend a little more on food for dinners, but that wasn't like a big issue and since I was was at home, like my parents were just paying for that anyway so I personally didn't affect see any effects.

Moderator 41:00

All right, so in our next set of questions we're going to talk about what your professors did and did not do during the move to online learning. So what are some strategies, tools, or technologies that your professors used that you found to be very effective in that it made it easier for you to learn during during online learning? So, 22.

22 41:21

Um so I think one was creating a discussion board where all of us could ask questions or like group chats or things like that. Um if we're just relying on ourselves and the professor, it seemed to work, not as well, like if we could have other peers that you could still interact with outside of the online class that worked best for me. Um, and then also, another thing that helped was having our webcams on and like, I don't know some of the some of our teachers were a little more forceful than others about that. But I think it really helped because it created more of a classroom environment for me. Like you were held more responsible and like had to pay attention, and it was less easy to get away with like playing on your phone or getting distracted. So, those are two big things I think.

Moderator 42:12

Okay and 24.

24 42:15

Uh so, I would say, just like for specifically my calculus class, I really liked it because the professor used like some application where he could like write on some sort of pad and like it would show up on our screen, so it'd be like as if he was using a whiteboard. Um so like that really helped because I could visually see it um and he would like send videos and so, and he would also do like live streaming where we could like type questions and he would answer them. So I really liked that, um, and then the professors, all my professors ended up doing, I don't know if anyone else here did LockDown Browser. But it was like, um, it pretty much prevent- like you couldn't do you couldn't cheat or anything but it was really frustrating because there were so many technical difficulties with it. Like if I was taking a math exam and I was looking down to write on my piece of paper it would ding me and because it would think I was cheating, but I was just writing a piece of paper and my computer is at my eye level not down here. So it was really frustrating because I keep getting dinged, and the professor would be like, oh yeah like I can see you weren't cheating. Keep going on or like it wouldn't work or the test wouldn't show up so that was really frustrating. It was kind of like, at this point, like, just let everybody use notes. Yeah, that's kind of where I was at with it.

Moderator 43:36

Alright so, um, what is what is LockDown Browser? Is um and 22, you said you were familiar with that too. That was something to help prevent students from cheating?

22 43:47

Yeah I've used it before. Basically what it does is it um prevents you from leaving the browser that you're on so it like locks down your browser. Um so you can't pull up any other tabs or um look at anything you have downloaded. Like you have to stay on that screen, or else like if you leave then you leave the test too. You can't come back.

Moderator 44:09

Okay, and were there a lot of students cheating? Is that why they were using these tools?

22 44:14

Um, I think that they were more concerned about it. I think that it would be easier for students. Um so some teachers did that. I also had a math teacher that made us um there was a thing on your phone that would lock down your phone too, and it would have a timer that showed how long your phone was locked down for so you'd have to show them at the end of the test um how much time and make sure it corresponds with how much time the test was and everything like that. Um and then some teachers kind of just made their tests a lot harder and said, you won't have your webcam on or anything like that but the test was way harder than it would have been before.

Moderator 44:54

All right, thank you so much. We just want to let 14 talk about what some, what are some things professors did that she found to be really effective.

14 45:05

Um well, I think the most effective thing was just doing like having the ones online lectures, um where they would have like notes, and you know they talk about their notes and whatnot, but I think it was really interesting because, uh like, my professors were really lenient compared to some, some things that other people have said like they were never requiring like attendance or cameras, or any of that stuff. Um they were just like yeah you know it is what it is. You can do open notes on your test, which was, I mean, yeah, that was definitely helpful. But, um, oh other than that, um like group chats with other students was also useful. And um but this semester, one of my classes does have like an FAQ discussion board where you can ask for help if you like don't get a question on the homework or something so that's also helpful.

Moderator 46:22

All right, thank you. And 29.

29 46:26

Yeah, I think for me um it was really helpful when the professors would have like a asynchronous lecture, so not one at an assigned time. And then, so it was just helpful that you could take you could watch it at a time that you felt like you could focus on it first. Sometimes like you have three in a row, and then by the end like you feel like you should go to them all, but you're really not like absorbing anything. And then um one of my professors, did that and then because it wasn't like at a set class time he significantly like reduced the time of his lectures just because he was very like precise about what he was lecturing about and prepared and so that was nice because it took a lot less time, and it was more clear. And he would post also post the his notes that he was writing on the board, which was really nice because then if you're watching you could just focus and not worry about writing down nice notes for it and just have those notes and have the lecture.

Moderator 47:26

Okay. So what are some examples of things that your professors did like technologies that you found to be really ineffective. So 22.

22 47:36

I think for me the pre recorded lectures were ineffective, which I think goes against a little bit about what other people are saying. But, um, I just, I think it holds me less accountable and it's also hard because you can't directly talk to the teacher and ask a question like you normally would. Um, yeah, I think that's the biggest one that I can think of right now.

Moderator 47:57

Okay, and 24.

24 48:00

Uh so I had one professor that was using lectures from like 2014 I think it was. So they were really old um and outdated, in my opinion,. Like if, if, like this lecture video said chapter 13, it was like chapter 25 in my current book. And I kept telling the professor I'm like it doesn't line up. Like this is a really old lecture and I would watch all the lecture videos and would study really hard with it because he would tell me he was like, alright look at like the lecture. Like the tests are lecture heavy and not as much as the book. And so I followed what he said about that and I just literally there was no correlation between the lecture and the test in my opinion.

Moderator 48:42

Okay, and 14.

14 48:46

I also think that the uh prerecorded lectures are not as helpful. Um one of my classes this semester also has that, and I just, like, don't feel motivated to watch them, per se, as as much as you would like, oh, it's a it's a live lecture. And you kind of are more invested in that. But, um, yeah I think that's, that's it.

Moderator 49:16

Okay, and 29.

29 49:19

Yeah, I think some of my professors just weren't good at like using technology. I don't know if the technology is probably I think is more like they were their learning curve of using that technology. Um so like using like figuring out how to like write on their iPads, so that it would show up on the screen, and I think like that just kind of wasted time. And then, because of that, in one of my classes we got really behind, but they didn't want to like take anything out, and they're like, oh, we're just gonna do like three weeks in one week. And so that was kind of stressful, but I don't think that was the technology itself. And I think one thing is kind of like more of a lack of technology is I wish we would have had some sort of proctoring or some sort of system because not like pretty very few classes at the U of REDACTED had um proctoring. And so, as a result, like everyone was cheating on everything. And so like you'd hear about all these averages that were just like skyrocketing and it was like people were just like video chatting with their friends during test to like health and there's just like Chegg was like full of all these examples of like tests literally just posted to Chegg, and then people would copy the answers straight from Chegg. And so it was like there a whole mess of just like academic dishonesty because we weren't using any proctoring systems or anything.

Moderator 50:48

Okay. So what are some things that you felt your professors did that made you feel like they cared, or didn't care about their students? So 22.

22 50:59

Um I had some professors that would actively um check up on us or ask during lectures like how we thought about things, and I really appreciated that that they even asked for feedback. Um and some professors just didn't do that and did their own thing. Um and I think that was really the big difference between the two.

Moderator 51:20

Okay, and 24.

24 51:22

Uh I had one professor who we didn't hear from for four weeks and we didn't have anything to do for his class. And so we were like, we no one knew what to do and this was when we were back from break and then he sends one email, and that's all we heard from him again. Um so that was like that kind of shows that he didn't care. That was also the professor that was quitting. Um so I guess it kind of makes sense. And then I had some professors that also asked for feedback, which was nice.

Moderator 51:52

Okay, and 14.

14 51:57

Um, yeah I had like one professor where he um like he was trying to adjust to how we would take quizzes in the class and he sort of like tried a couple different formats and he was like, oh, do you like this? If it doesn't work like you can do corrections on it. We can try a different format. I thought that was really nice of him. Um yeah, mostly everything else is just the basic like video lecturing, so.

Moderator 52:36

Okay and 29.

29 52:42

Um, could you repeat the question? Sorry I just totally.

Moderator 52:47

Yeah. Was there anything that your professors were, what were some things that professors did during online learning that made you feel like they cared and things that they did that made you feel like they didn't care?

29 53:00

Okay, thank you. Yeah, um so I think I had a couple of professors who gave us extended deadlines for things near the start of the transition to online school. I think that was really helpful and made me definitely feel like they cared and understood that it was like a hard transition. And so that was nice, and then I had one professor who made the final project um extra credit, instead of part of your grade. And I think that also showed that they cared and weren't like so stringent on sticking to exactly like what everything had been before COVID.

Moderator 53:41

Okay, so in our next set of questions we want to know more about your university. So let's talk about how your university responded to the move to online learning. So what are some things that your university did like pass fail options, town halls, remote counseling to help students be successful during online learning? So these can be any resources that your university provided for you, or took action to help students be successful or feel supported. So 22.

22 54:13

Um the they did pass fail for us, and that was helpful, especially with that one class that I was struggling with, um, so I utilized that. But a lot of the other things I just didn't really pay attention to. I'm sure they offered a lot but I just kind of disregard a lot of the emails that my university sends me, unless they're they're actually applicable.

Moderator 54:37

And 24.

24 54:39

So they had a pass fail option available but we all came to find out that if um the class at all like applied to your major, you would have to retake it. Um so pass fail literally didn't help anybody. I didn't, I didn't meet a lot of people that did pass fail, um which kind of I felt like uh pass fail should have been an option. But they also sent out like a Canvas course to everybody and it had like a bunch of resources. Um I didn't look at it, per se, but I know that that was available.

Moderator 55:15

Okay, and 14.

14 55:20

Um, we also had pass fail, which I didn't have to use, but it was, it was nice to have it. And I do also know that they kept um tutoring services, and they moved them online. Um, they did have like a town hall event that was more specifically about like fall semester and like what they're gonna do about that. And people (laughs) didn't really react well to it because they wanted to keep freshmen classes in person and putting like the upper level classes online but they did have, like, a zoom session for that, so they tried. (laughs)

Moderator 56:10

Okay, and 29.

29 56:14

So we had a pass fail policy, and you were able to use it on major classes, which was really nice and decide after you got like your final grade back, which is also really nice. Um, and then our tutoring moved online too. So I had to tutor before, and then we just met over zoom afterwards.

Moderator 56:41

Okay, and was that a tutoring resource uh that you paid for or was that provided by the university?

29 56:47

Uh it was provided through the athletic academic center. So I wasn't paying for it but it was because I was an athlete. It wasn't because just a general student.

Moderator 56:59

Okay. And so what are some things you wish your university had done better to help students be successful during online learning? Um, 22.

22 57:10

Um, I just felt like the way that they were reaching out wasn't very effective. Um, especially because it was just, it just seemed very superficial just like a normal standard email that they would send out. It didn't seem like they were really connecting with the students as well as they could. Um so that and maybe um trying different ways to bring students together would have been more helpful.

Moderator 57:33

Okay, and 24.

24 57:36

Uh I really strongly dislike that, um, the pass fail wasn't an option for our university. Well it was an option but I mean if your course applied to your major at all, like it was required, then you'd have to retake it so no one wanted to do that. Um so I didn't really like that, especially because I don't feel we were provided with all of the materials we needed to succeed in the courses but uh so I I would have liked to have seen like a different option that way.

Moderator 58:08

Okay, and 14.

14 58:11

Um I don't, I don't really have any like complaints about that. I think they did the best with you know what they had so.

Moderator 58:24

Okay, and 29.

29 58:27

Yeah, so I'm not sure what this would have looked like, but I feel like it would have been nice to have another more ways to connect with other students in the course since we didn't have that like face to face, and like, in, like, in none of my classes, did anyone like ever turn on their video so it was kind of very isolating to just be in these classes with people in black boxes all the time. And just like, like I like a couple of friends like I had before, COVID, like classmates so like I was able to connect with them a little bit to study and stuff, but I wish it would have been like easier in some of my other classes to connect with fellow students, or at least feel connected in some way.

Moderator 59:15

Okay. So, in general, are there any ways your ability to be successful in school that was affected by the pandemic that we have discussed today. 22. You can just say no if the answer is no.

22 59:29

No.

Moderator 59:31

24.

24 59:31

Uh, I think we touched on it, unless there was anything like a social aspect if like in my opinion, like that could affect people like if you're a social person or you like to study with people. Um like I think that could have affected but other than that I think we touched on everything.

Moderator 59:47

Okay, and 14.

14 59:52

Um yeah, I don't, I don't think there's anything else.

Moderator 59:56

And 29.

29 59:58

Yeah, I agree with what 24 said about like the social aspect. And then I think like also like very particular to my situation like being like is it kind of helped being, like, for being an athlete, the COVID was actually really nice because it gave you a lot more time, instead of having like 15 hours of practices a week like we just could do like a little bit on our own, so.

Moderator 1:00:24

Alright everyone, thank you for participating. That was all of my questions today. It was nice meeting you and chatting with you. Have a great day.

Low SES White Men

Moderator 20:14

Okay. All right, cool. Right, so. So that's Moderator. I'm Moderator. I will be asking you guys today, number of open ended questions. It's important for you to understand that there are no wrong answers, but people may have different points of view, that's totally fine. Please feel free to share your point of view, even if it differs from what others have said, that's kind of what we're interested in is everyone's perspective. So along those lines. Keep in mind that we're just as interested in negative comments as we are about positive comments. We want everybody to feel comfortable participating and encourage all of all of us to share all of our thoughts, because we do have to keep this to an hour. I may need to cut you off. We have a lot of questions to go through so it's, it's just trying to keep on time, so I apologize in advance if we get to that, with a smaller focus group I don't expect that will be an issue, but just in case we do start to run over time. I just want you to know that I may have to keep things moving along. We do want to get the full breadth of experiences and opinions, but some of you may have similar experiences and opinions. And if that's the case, you can just say, my answer is the same as 48's or my answer is the same as 47's if they have captured what you would like to say on this, on any particular question. It is difficult for us to take notes I mentioned that Redacted will be here taking notes so we have a good record of everything that said but it's difficult to do that with 100% accuracy so we will also be recording the session. And that recording will be used just to transcribe it. We will not associate your name with anything you say in the focus group, and the recordings, as I say are just used to transcribe so we have a written record, and then the recordings will be deleted after. You may refuse to answer any question, you may withdraw from the group at any time. And we under, we understand that all of this information is very important and needs to be kept private and confidential so we do ask that you respect the other participants confidentiality and not repeat things that are said within the group to anyone outside of the group. So those are the ground rules. Before we get started, do you have any questions.

47 22:44

I'm Good.

Moderator 22:46

48.

48 22:47

Good. All good.

Moderator 22:48

Okay. Well then, let's get acquainted a little bit. So if you wouldn't mind, could you please say your name, and let us know what year of college you just finished. Did you just finished your first, second, third year, whatever. What year you just finished, and one word that you would use to describe life during the pandemic. 48 how about if we start with you, What year did you just finish. And if you could capture your life in one word during the pandemic, what would it be.

48 23:25

I just finished my freshman year and I would describe it as boring.

Moderator 23:30

I think that's gonna be a common answer. Yeah. Yeah. 47?

47 23:36

I just finished my sophomore year I study math and either depressing or devastating. Yeah.

Moderator 23:49

I'm afriad that's gonna be a common answer too. Yeah, because that seems to be widespread experience. Okay, well it's great to meet you guys I'm glad you guys are with us today. So, let me just give you an overview of the project that we're working on and then we'll just jump right into the questions. So the COVID-19 pandemic has presented big challenges for undergrad students, as you know universities across the nation have closed moving all instruction online with very little time for students or faculty to prepare. Many students encounter challenges related to the closing of campuses, related to relocating, movement to online instruction. In this study we want to learn how the closing of campus and the pandemic in general has affected you. And we want your opinions about how the good and bad. I'm sorry about the good and bad, and how things went, as well as what your professors and universities did that was helpful and unhelpful, and what you wish they would have done. So that's the big picture. Here's a specific question for you to get us started. Let's first discuss where you went when the campus shut down. So, did you go home, did you live in an off campus apartment and you just stay there, like when when the shut down at your campus took place, where did you go. 48 we'll start with you again.

48 25:16

So for me, they, we were home on spring break, and about like three or four days before we were supposed to go back, they sent us an email and said don't come back. So a lot of us ended up trapped at home without our stuff from the dorm. So a lot of people had to go a couple months without their stuff. But yeah, that's when they told us just a couple days before we were supposed to come back.

Moderator 25:37

Okay and so you were at home. Yeah, for the, for the remainder of the semester. Yeah. Okay. 47?

47 25:44

I moved around a lot. So for spring break, I went home. I don't live like too far from campus but I went home, and then over spring break, that's when they announced the quarantine was gonna start and not to go back and that was a problem for me because I have a pretty tough home life, so I just went back, even though my dorm was like technically closed. I just told him I wasn't able to go back, stayed there for like three weeks and then realized it was too expensive so I just went back home. And then, that didn't work out so I just had to get a summer apartment. That's why I'm here now.

Moderator 26:27

I see, I see. Well going forward so we're going to ask you some follow up questions about the space you have and so on and since you were living in multiple different places, if you could just try to answer the follow up questions about the place where you spent the majority of your time. Wherever, wherever that was. so yeah so what is one of these follow up questions, it's Could you please tell me about the space that you moved to and specifically what I mean is the space, what space, did you have wherever you were living to do your schoolwork, were you just in a bedroom and you had to work on a bed, did you have, you know, kitchen table that you could work at, a desk, like what was the space that you had to do your schoolwork in the place where you spent the majority of your time. 48.

48 27:22

Oh, my dad works at a school so I just had him bring, an elementary school, so I just had him bring me home one of them elementary school desks and I put that in my room and I just worked on that, I found that easier than working on my bed or working on my desk where I like play video games and stuff, it's easier to focus when I had like my own spot.

Moderator 27:41

And so, was that, if that was in your bedroom, was that a private space or a shared space.

48 27:48

Yeah, it's my own my own space.

Moderator 27:50

Okay, so you had a private space in your bedroom working on a small desk, wherever you could just call that your workspace. Okay, 47?

47 28:01

it was divided between my bed, and a shared desk. Me and three other people shared it, and only one of them, like my younger brother was a student also he was in high school, and both my parents used that desk also, and it was also like not in a quiet area.

Moderator 28:24

Okay, so heavily shared space. Yeah, it sounds like some distractions going on around you. All right. What challenges did you encounter attending classes or doing your schoolwork in this space.

47 28:44

I'll be honest, I didn't really attend classes. I mean, all I did the bare minimum. Yeah,

Moderator 28:57

and what did that, what, could you help me understand what that bare minimum might have looked like.

47 29:03

So, in a lot of, in a lot of my classes, they turned to pre recorded lectures. I would watch some of those. The most. The only graded assignments were like they were not attendance based so I could just do those whenever. like turning in a, an essay or turning in a math assignment. So,

Moderator 29:27

you've worked on that in your own time whenever you had the time. Yeah. Okay. Okay, 47 what challenges did you encounter, attending classes or doing schoolwork in that space.

47 29:40

Oh, that was me until I'm sorry.

Moderator 29:42

I'm sorry. I lost track of whose voice was whose. That was 47. Alright, 48 I'm sorry, could you please.

48 29:50

I never really had any troubles with that workspace. I actually really enjoyed it. Like, because in the past I've always done homework at just like my desk whatever but it was nice to have like a new desk. to have like an area that's dedicated solely to schoolwork. But um, I did share some of 47's experiences with doing the absolute bare minimum, like I do my math homework like the day before. Lots of times I just copy straight from Slader, not even do it you know, just to get the points. And then when they gave us our, our finals in math, they gave us like 48 hours to do our midterms and our finals and they left them open note online. So they didn't really encourage us to work at all, because it was open, it was open Internet, like they said that. they gave us 48 hours so it's like, none of us really even tried. like there was no reason to.

Moderator 30:47

I see, you just scrolled the internet to find the solutions tab.

48 30:52

Yeah. Yeah,

Moderator 30:53

got it, got it. Okay. Um, what are the things that you or other people in the House did that made it easier for you to attend classes, like what what do you think went well, you know what I mean. So what did you or other people in the house, do that made it easier for you to attend classes and/or do your schoolwork. 48?

48 31:20

they didn't bother me when I, when they knew that I had classes going on. They left me alone. I didn't really have those kind of distractions going on.

Moderator 31:30

So you felt like your boundaries were sort of respected, if you will,

48 31:33

yeah.

Moderator 31:35

47, what, if anything, went well.

47 31:39

I think my boundaries were like, one third respected. And sometimes I sometimes I would, I would say like I'm in class or whatever. Even if I like wasn't like if I was just watching YouTube video about math but not necessarily for school, I would just say I'm in class and then like my siblings would respect me but my parents wouldn't. So at least there was that.

Moderator 32:07

Okay. Okay. All right. Was there anything else that might have went well or that, like in retrospect, you wish you would have done more of because it seemed to be working.

47 32:20

There was always enough food. like that. That's something I noticed in the survey that that I didn't struggle with, like there not being enough food or anything.

Moderator 32:29

Right, right, right. Okay. All right, great. So let's move on then. Let's talk about what other responsibilities you had after your campus closed and how these differed from responsibilities you had before campus closed. So, many of these questions may not apply to you and if not, that's fine, we'll just just say so and we will move along. But if you could describe your caregiving responsibilities, including child care, elder care, caring for sick, sick loved ones. You think about your child or your caregiving responsibilities, how were they alike, or different from your responsibilities before the shutdown. 48.

48 33:13

I've been babysitting my little brother every single day since, since the shutdown, because my parents are still working. So, I have to stay home with him, and I had to do that all through the school year and I'm still doing it now.

Moderator 33:26

So, that sounds to me like quite a increase in your responsibility

48 33:30

Oh yes, oh yeah it went from zero to 100.

Moderator 33:35

Okay. All right, so that's significant. 47, how would you characterize your caregiving responsibilities before and after.

47 33:46

So before I was just in charge of myself, and I made sure to be like emotionally there for my friends. So like not too much of a burden, I guess, but at home, It seemed like all of my friends were freaking out so like, we spent a lot of time like I guess emotionally, keeping each other, sane, and at home I was pretty busy with like, hey, 47 you have to fold laundry, like, I can't, I'm doing homework like I don't care, fold laundry, like, whatever. Okay.

Moderator 34:24

Right, right. So, so, in terms of caregiving responsibilities, you would say there was an increase in not just not just i mean maybe primarily with your friends, and emotional support for your friends. But was there any caregiving responsibilities at home. I mean, in addition to the chores and things like that, or not so much.

47 34:51

I'd say just general household chores, and lots of fighting. But other than that, probably not.

Moderator 34:59

Okay, okay, well that that actually is the next question. So describe your other household responsibilities like chores, cooking, caring for pets. How were those alike, or different depending on, you know, before the shutdown and after so 47 you've already spoke to this a little bit you you had more. Well I should I, I should ask Would you say that your household chores were more after the shutdown or less compared to before.

47 35:32

A lot more a lot more.

Moderator 35:34

Okay, so household responsibilities definitely went up for you. Yeah. Okay, 48

48 35:41

went down for me cause when I was in college I was doing like everything, you know I'm cleaning my room, constantly, I'm doing all my laundry, I'm doing dishes all the time cause I only had like two bowls. And now here at home it's just like, my parents are great about just doing everything for me. So I definitely don't do very much at home, other than watching my brother every day.

Moderator 36:07

Okay, so the childcare thing increased from zero to 100 but other aspects of daily life, sort of went down.

48 36:16

Yes. Very much.

Moderator 36:18

Got it, got it. All right. Well, to what extent. let me see here. Sorry. To what extent, Would you say that you were expected to take on more household work, or those child care, family caregiving responsibilities, More household work than other people in your home so for example, parents sometimes expect more of one sibling or partners or roommates might not contribute equally So to what extent Would you say that you took on more household work than other people. 47?

47 37:02

I want to say, I'd say the household work was assigned pretty evenly.

Moderator 37:08

Okay, 48

48 37:11

Um, I think I'm definitely on like the lower end of contributions to household work. So, in college, I lived with my girlfriend in the dorm in college and we split that about evenly. But then when I went home. My parents just take care of just about everything in the house.

Moderator 37:29

Okay. Okay. All right, great. Thank you very much. So let's, let's move on to academics. So let's talk about how your academics were impacted by the shutdown. I'm going to ask about a bunch of things. And if you think one of the things that I mentioned didn't impact you, just feel free to say no impact and we'll, we'll move along. So, in what ways if any has the shutdown impacted your academic performance, your grades for instance, and your understanding of the subject matter of your courses. 48 What impact did it have on academic performance in the sub, your understanding of the subject matter.

48 38:13

Yeah, like I was saying earlier, in my math class, they just like gave us a final and it was open note open Internet open everything. So, there wasn't much of a reason to put effort in. And because of stuff like that. I don't even really remember what we learned, like I don't even remember what the last chapter was on in math or physics cause like, since it's all online it's really hard to like actually be engaged. So yeah, I think this shutdown very negatively affected my academic performance, my ability to retain information.

Moderator 38:49

Yeah, yeah, yeah that's what it sounds like. 47 how would you say your academic performance and understanding of subject matter was impacted.

47 39:00

So negatively. That, I think, like 50% of the way through like that's when quarantine started to like 85% of the way through this semester, I barely learned anything. And then like the last 15% was like finals and such, I like obviously had to learn there. When it asked in the survey how many classes, I take or how many I had taken. I had trouble remembering which classes I had taken, because,

Moderator 39:37

because that's where we're at. I don't even remember what I was supposed to be doing.

48 39:41

I have the same problem.

Moderator 39:43

Really.

48 39:44

Yeah.

Moderator 39:44

Really, that's interesting. So both of you even had to sort of really dig through the memory bank to remember what courses you were in.

47 39:53

I had to check my schedule.

Moderator 39:55

Not even the memory bank. You had to go to a written record, to find out.

47 40:00

I was like I know I took 18 hours I just don't know which ones.

Moderator 40:04

Okay. Wow. All right, yeah. Yeah, that's really, that sounds to me like a huge impact on understanding. In what ways, would you guys say that this this shutdown has impacted your career goals, long term career goals.

47 40:29

I had a summer internship lined up, and then that crumbled. So I had to take a, I ended up finding five weeks of employment, but now that, now that I'm on campus. And I only have a bike. I haven't been able to find a job. I mean like even a non academic job, that's hard to find. So going from a full summer of academic experience to none. That was a pretty hard hit.

Moderator 41:02

Yeah, yeah. Well, so, I mean I hear you and that's, that's definitely a big problem for you and a lot of other folks it's really been a struggle. But would you say that this impact of the shutdown rather has impacted sort of long term career goals where you see yourself in five years or in 10 years or is that just not been on your radar. Yet,

47 41:31

a mix between hasn't been on my radar and once I'm 30, it won't matter. Yeah, like I'll be fine eventually,

Moderator 41:44

you know, you sort of have faith that this will shake itself out in the end but it's just kind of crappy going through it right now.

47 41:51

Yeah, like, like once I'm a high school teacher, it won't matter what my. What I did this summer.

Moderator 41:57

I see so that was your career goal, both before and after the shutdown.

47 42:01

Yes.

Moderator 42:03

Got it, got it great, thanks. 48, how would you say the shutdown has impacted your career goals.

48 42:09

I don't, I don't feel like it's impacted me at all.

Moderator 42:12

Okay, great. Do you mind sharing what those career goals may be,

48 42:17

yeah I'm going for civil engineering. So, um, I just don't see a way that the pandemic would be able to affect me many years from now, if I was in 47's position and I had an internship lined up, and that fell through, then that would suck. But, like I'm just a freshman, so I didn't have a, I didn't have an internship or anything lined up, I was just working, and I'm working right now making more money than I ever have before. So, I don't really feel like it's going to impact me at all and it's like the Black Death back in the old days, that only lasted for like two years, when they got through it you know so it's like, we'll be fine.

Moderator 43:00

That's how you see it.

48 43:01

Yeah.

Moderator 43:02

Okay. All right, well so 47 already mentioned, one thing but let me ask the question anyway if you have nothing to add, just, we'll move along and if you have something to add we'll make note of it. In what ways has this shutdown impacted the availability of other learning opportunities such as internships, are there other other sorts of learning opportunities that you feel have been impacted by the shutdown. 48?

48 43:32

um, none that I can think of off the top of my head no.

Moderator 43:36

Hey, 47 Anything to add that you didn't already mention

47 43:48

this is kind of secondhand but my, my motivation to learn went down a lot, so like my motivation to like, seek out extra education opportunities, also was like, just gone

Moderator 44:06

took the wind out of your sails, so to speak.

47 44:09

Yeah.

48 44:10

Yeah. I did think of something actually, um, I was planning on taking chemistry over the summer at a community college. And then after the pandemic, I decided not to do that because I knew it would be online, and I didn't want to pursue an online course because I knew I wouldn't perform well.

Moderator 44:28

Right. No, that's, that's an important one, thanks I'm glad that, glad you remembered that and brought it up, because that's exactly the sort of thing that we're trying to get a record of so, Thanks.

47 44:42

Actually, 48 that that brings up something.

Moderator 44:45

Please, please, please.

47 44:46

Yes. This isn't definite but, um, I had hoped to take a differential equations course over the summer, and like at the end of the semester I was just going to shop around, I guess. And then when I did shop around, that's when I found out it was, it was gonna be like, all like anywhere I went, it was gonna be online, and it was still gonna cost the same, and it was absolutely not going to be worth it.

Moderator 45:11

Oh, right. No, no, that's

47 45:15

go from a maybe to a no

Moderator 45:18

went from a maybe to a definite no

47 45:20

Yeah.

Moderator 45:21

Gotcha, gotcha. All right. So, did any new learning and or career development opportunities emerge during the shutdown, and if so, what were they. 47, any new opportunities emerge.

47 45:42

So I took this one, I took this craft class that was one credit hour, and it actually took place during the second half of the semester so it was entirely online. The class is called design your life and career, it's, it's kind of meant as like a, the analogy that the instructor used was like this is kind of like a compass, for your life and career and like this class is like teaching how to use the compass. And a lot of it was like reflective responses like hey read this article about how a career shouldn't be all about money and then reflect on it. I thought that like every bit of content and that whole class was really relevant and helpful for me. even the aspects that didn't quite like change anything, it at least like helped me get my bearings helped me like, keep my cool, I guess. Okay. It was one of the things that went well.

Moderator 46:42

Yeah. So this was a course that you were signed up for before we knew that there was going to be a shutdown, is that correct,

47 46:51

yes. Originally it was gonna meet in person.

Moderator 46:54

Okay, okay. But if I'm hearing you correctly. Even though you were signed up for this before the shutdown took place, the, the opportunity or the sort of new experience that you had was, you felt like this course really helped you get some perspective and reflect on the, what your life would be like going forward in the shut, or after the pandemic is that is that what I heard or No.

47 47:23

Yeah, and also like a lot of the content was like time relevant, meaning like, how can you change this during quarantine or how can you reflect on this during quarantine also.

Moderator 47:38

Oh I see, so they sort of tailored some of the content, of course, to the pandemic to help you guys deal with the pandemic. Yeah, I see, I see, okay, no that makes that makes more sense to my head now so thank you for clarifying I appreciate that. 48 any new career development opportunities emerge during the shutdown.

48 48:03

Um, I actually did get a job at a manufacturing plant. Doing inventory. And I don't think I would have got that without COVID. And I view that as like a resume builder because it's my first like job outside of retail or food. so I think going forward having that on my resume will help me in my career.

Moderator 48:28

See, okay well that's really great. Congratulations on that. That's great news. Um, let's see what's my next question so in what ways if any has the shutdown impacted your confidence in your ability to successfully complete your bachelor's degree in a timely fashion, 47. What impact is has had on your confidence.

47 48:53

Deep down inside of me. I've always known that I'm going to complete my bachelor's degree. I've never failed a class, and I'm surprised that I actually got good grades this semester. That kind of helped, like, I actually got the, like my best GPA, this semester. In all of college, even though it like went to total chaos. For most of it, so that in a weird way I'm, I'm kinda like, Well at least I got through the worst part of college. So my confidence is like 5% increased. After like a 700% decrease.

Moderator 49:48

Is that good now I know I am struggling to understand so

47 49:52

net net positive a little bit

Moderator 49:54

net positive, a little bit increase in your confidence to withstand really chaotic experiences is that.

47 50:03

Yep,

Moderator 50:04

that's about right. Okay. Great, thank you. 48. What impact did the shutdown have on your confidence to successfully complete your degree.

48 50:13

I'm still very confident. I don't think it decreased my confidence at all. I think um 47's experience it's kind of the same for me it's just like going through something like this makes me realize that like, no matter how bad the classes I'll probably be able to get through it.

Moderator 50:29

Ooh. Okay, so, would you characterize that then as a small net increase or or no.

48 50:38

In, in what, in my confidence.

Moderator 50:40

Yep, Yep.

48 50:41

Um, maybe a little bit. Yeah.

Moderator 50:44

Okay. All right, great. Thank you very much. So both of you have sort of touched on this already but if you wouldn't mind just sort of repeating yourself so we make sure we get it straight. In what ways if any did the campus shut down impact your finances, and I don't just mean, like, you know, you got a better summer job. All of the finances. Were there implications for work study programs that you were part of for, you know, financial aid, any sort of impact on finances. What were they all these 48.

48 51:26

Um, University of REDACTED actually treated us really well. They gave us about $3,000 back for room and board, and they continued to pay us for our work study, like our, they took our average hours that we had been working and they just kept paying us through the end of the semester. And on top of that I started working three different jobs. So, financially, I'd say I'm in a very good spot. Despite the, despite the pandemic.

Moderator 51:57

Right, right. Okay, all right, wonderful, 47, what was the impact on your finances.

47 52:06

I had been struggling with like paying for college. The entire like throughout my first two years. And like that was like something very stressful for me. So I ended up, like when I did like have to move out of home. I had to use that small amount of savings to like sublease an apartment. And like also because of that I've been like buying food and buying, soap, and clothes and stuff that I wouldn't normally have to buy. Yeah, and like utilities and such, a lot of funds that I didn't expect to have to pay. And also, the I had two part time jobs during the semester, like tutoring and like this other side hobby, and both of those ended with the pandemic.

Moderator 53:03

Ooh. Sounds like, sounds like a lot of negative impacts to me. Oh yeah.

47 53:09

Yeah, so it's pretty big negative impact.

Moderator 53:14

Okay. Well I'm sorry to hear that. So, let's move on then to a new topic, which was your professors. So, basically, we'd like to know what your professors did well and didn't do well, roughly speaking, but let me be more specific. What are some examples, concrete examples if possible, of strategies tools technologies that your professors use that you found to be effective, and that they made learn, learning easier or very ineffective during online learning so what the strategies tools technologies. What did they do that was effective or ineffective. 47 why don't you start this time.

47 54:01

So to start, in all in all of my classes. The professors and instructors knew that -- all like, everybody was affected by the pandemic so they just did their best. And that's really all we could ask for. In my math and stats class. Originally we would meet three times a week, again in person that time was like instead they started doing better lectures pre recorded and the original extra time was just there for office hours and like a help session office hours were by arrangement. So like, Hey, can you meet right now it's like nah give me 30 minutes. Okay, cool. That's typically how it would go with my instructors in one class though our lectures turned into, like, every single lecture was turned into a PowerPoint, and often the PowerPoints were so large, we would have like a bunch of students would say hey it's too large for us to download, so he would have to like manually separate it into two and then like at the end I deleted like over a gigabyte of PowerPoints.

Moderator 55:14

So I'm afraid I lost your audio right when you said they were so, and then it went out and then you came back and said you've got a bunch of huge PowerPoints, so they were so what?

47 55:27

The PowerPoints were so large..

Moderator 55:29

So large, okay,

47 55:30

They couldn't be downloaded, like at once

Moderator 55:34

hmmm

47 55:34

And they broken into two by the professor and often that was like a day after the lecture was supposed to take place.

Moderator 55:43

Okay, okay. So that sounds very ineffective. How would you, how would you characterize you'd said that your professors, often pre-recorded lectures and then they would have office hours during regularly scheduled class, or perhaps, in addition to on-- by-- by appointment, would you characterize that as effective or ineffective?

47 56:11

I'd say effective. Um, I know that a lot of classes did that, it wasn't like original in my classes but it was definitely a good solution. And honestly, that's kind of the strategy that like Khan Academy and other newer learning resources are adopting so I think it was definitely a good move on the university's part.

Moderator 56:34

A great thank you 48, what did your professors do strategies tools technologies that was either effective or ineffective in your opinion?

48 56:45

So for my math class, I actually never figured out how to access the lectures. So, I never attended a single math lecture since the coronavirus started..

Moderator 56:57

Ok

48 56:57

In physics everything was pre- recorded, but my professor he would all -- he would do it all in one take. And he mumbles and and stutters a lot. So, um, those lectures were really hard to follow, even though they were half as long as the in person lectures he did, he did them in one take, so it was really hard to follow my writing class, we met together in a live zoom call and I think that went really well because it was mandatory. So you couldn't leave so the whole classes there and we had good discussions and it was basically the same as an in person class and philosophy. She held live lectures, and also recorded them. So if you couldn't make it. You could watch it later. And that was my favorite way of handling the whole thing being able to watch it later if you need to. But been encouraged to join it live.

Moderator 57:53

I see so so of the-- I just want to repeat what you said and make sure I got it correct of the various class structures that your instructors use the one you thought was the best was the one where they would have live lectures and students were encouraged to be there but they also recorded it just in case you couldn't...

48 58:17

Yes

Moderator 58:17

.....make it or wanted to, you know, brush up on some particular point afterwards.

48 58:21

Yep. And it was also really nice having that as review for the final like that was actually something about these uh about the coronavirus times that I did enjoy is having those lectures available to go back and check for topics you might have missed I use that in my physics class particularly, um, because it was really nice to be able to go back to like the lectures from the very beginning of the coronavirus and be able to catch up on that stuff because in traditional in person classes, it's not something you can do you have to go to the textbook.

Moderator 58:55

And you you you took advantage of that?

48 58:57

Yes

Moderator 58:57

Like you actually went back and watch the lectures again.

48 58:59

Yeah, for review.

Moderator 59:03

Could I ask Did you ---was that an option for you and if so, how much would you say you went back and watch lectures?

47 59:12

So, in my stats class. Kind of like similar to 48 story I didn't, I actually never figured out how to access my stats lectures, but I know that those were recorded because students would ask questions live and like that that would be something students could do later, but in my other classes. I don't know if this was accurate, but professors would say like we're not allowed to record students because that would like violate privacy laws, and I thought that was bizarre. Because it seems so ineffective and impractical, but also like something that university could totally change if they. I guess made it more clear,or if they thought ahead.

Moderator 59:55

So I you saying then that a lot of like...

47 59:58

Alot the other classes were not recorded,

Moderator 1:00:00

Okay, right,

47 1:00:01

there was only one

Moderator 1:00:03

And the one that was recorded you weren't able to get access to the recordings anyway.

47 1:00:08

Yes

Moderator 1:00:09

Got it, got it. Okay. Well, it sounds like you guys had quite different experiences in that regard. Um, so, next question. What are some things your professors did during online learning that made you feel like they cared, or did not care about their students are there specific examples of things they did that made you you know gave you the impression that they did or did not care about we start with you.

47 1:00:42

I had to ask, several of my professor, professors, like for deadline extensions just because like, things were kind of out of my control for a while, and school is included in that they were very understanding and they didn't. They didn't ask for a ton of details, they just said like, I trust that you're being honest. So there are deadline extensions, and generally just like they would check in with us via email and say like, hey, how's this class going. Am I doing an ALRIGHT job do you feel like this class is still happening I guess. So they kind of like, do progress check ins.

Moderator 1:01:25

Right. Okay, the fact that they were reaching out to you made it feel like they cared about your success and well being and so on.

47 1:01:35

Yeah. Yeah.

Moderator 1:01:36

Okay, wonderful. Thank you. 48, what did your professors do that made you feel like they cared or didn't care.

48 1:01:44

My stem professors kind of just ignored us.

Moderator 1:01:47

Oh no

48 1:01:47

So that made me feel like they didn't care.

Moderator 1:01:50

Yeah

48 1:01:51

But um, I mean I've always felt like that about the stem professors. But um, my math TA. She was incredibly helpful during the coronavirus thing like I would send her an email asking a question about one specific thing. And she sent me back like two pages of her own writing, explaining that one specific topic, and I thought that was really really nice and it helped me a lot. And also my philosophy professor she was so sweet, she would check in with emails all the time. She during classes, she would just let us know that she understands what what's happening, she's going through it too. And on our final she cut off the most difficult unit, because she understood that it's really hard for us to get all this reading done when we're dealing with all the online classes and stuff. So, she was very attentive and that made me feel like she cared, the stem professors just completely ignored us, and that made me feel like they didn't care.

Moderator 1:02:54

That's a powerful statement. So, if I could ask, then how many of your courses where would you regard a STEM courses and the other non STEM courses,

48 1:03:04

I had physics and maths, those are my two STEM courses and then I had writing and philosophy.

Moderator 1:03:09

Okay. And the physics and math teachers, just fell off the radar, so to speak.

48 1:03:15

Yeah, yeah, they just did the lectures and that was it.

Moderator 1:03:18

Right. Right. Okay,so we're on to the last sort of broad category. We'd like to talk now about your university, and how your university responded to the move to online learning. And here I just like to ask you to distinguish between what your professors did which we've already talked about course, and what your university did. So what are some things that your university did for example, did they give a pass fail option for grades. Did they have town halls for students to voice concerns was their remote constantly What did your universities do to help students be successful. During online learning. And if you could mention any against specific resources that the university may have provided. That would be super helpful for us so 47 what did your university, do

47 1:04:11

I think the university did a bad job. Overall, with its like with its academic coronavirus response. Um, The-- I don't think the university gave us any money back for like the classes being online and like the quality being severely affected. I don't know if they had any way of holding professors accountable for teaching classes. I know that like I lucked out with professors that cared, but I know a lot of people are like yeah I didn't hear from my professor until the final like one of my close friends said that. And like she ended up failing that class. One thing that really upset me and a lot of students is they did have a pass fail option, but you had to decide may 1. Like before, and before finals before grades were even reported at all. And then they they so generously extended it like five days. Still not into the realm where it would have been helpful. So you. The thing. Basically what they were doing is the students would have to like, I guess gamble on their grades and be like, I don't know? Am I gonna fail this? Am I gonna pass it?

Moderator 1:05:35

I see I didn't, I didn't quite understand you, but what what you're what you're saying there is that you had to take a guess as to whether you're going to get a good grade or a bad grade, before you took your finals before you knew. And you felt like you were making that guess without enough information to make a good guess.

47 1:05:55

Yes. And also like because of the general kind of disorder of the semester, barely any grades in any of my classes were reported at all. At that time, so I --- it truly was just a shot in the dark. Um, Yeah.

Moderator 1:06:16

okay. Okay, so that didn't go too well.

47 1:06:21

But also, the, like, one more piece of information about that is if you elected like pass fail or credit no credit is like what it was called the, like, instead of failing it by getting an F. if you got a D or an F. And you elected pass/fail, then if you got a D or an F that would count as a fail. So,

Moderator 1:06:44

Really?

47 1:06:45

So, originally if you got a D in a class. And you elected a letter grade, then like you'd get a D, whatever. But if you elect pass fail then the D would fail you

48 1:06:57

Ours was the exact same way. If you get a C minus or higher, to pass a pass fail. Yeah.

Moderator 1:07:04

Really? and so a D would also be counted as an F in this new scheme.

48 1:07:11

Yeah.

Moderator 1:07:12

Okay, well I can see where that would be very crappy decision to have to make, but not very good information. 48 anything that your university did that you help that you felt was helpful for success.

48 1:07:29

I just want to touch on the pass/fail thing really quick, um, the reason our university did it is because anything lower than a C minus doesn't actually count as a pass for your major anyway, in the first place. So if you're taking a like an elective course and you get a D, that's fine. But um, if you're taking a required course and you get a D. That's a fail, like, ooh, so it's not like the pass fail really changed much that's just kind of how it was all the time.

Moderator 1:07:57

Do you guys know? Um, So this just raises a question about GPAs right because I guess the D. on a GPA is different than an F on the GPA. Do you know if you took this credit no credit or pass fail option. If that was going to impact your GPA or was that just a separate.

48 1:08:17

Pass/failed doesn't affect your GPA at all.

47 1:08:20

Same, same with University of REDACTED.

Moderator 1:08:23

Okay. Okay. All right. All right, great. Thanks for clarifying that.

48 1:08:28

Yeah. Anyway, my, um, my University's response I touched on it earlier, I think it was very good. They were very attentive to the students voices. I actually sent emails to two of our regents they call them. They had the region meetings, open to the public, you can watch them on YouTube. So I would watch those and if I agreed or disagreed with something somebody said I'd send them an email, and they would respond very quickly. We had the same situation as 47 with the pass fail they set the deadline to like April 30, I think. And then a bunch of us started signing a petition to extend that date, and eventually they listened to us and they extended it to like 10 days after the end of the semester for us to choose. Yeah, so we got plenty of time to do that. Originally they were only going to refund us like a portion of our room and board fees for the second half of the semester. But that's actually something I sent an email to one of the region's about, and they decided to reimburse us for the entire remainder of the semester or room and board fees or refund, that they're giving us went from like 1500 to like 3000. So, it's good. I feel like our university did a great job of of helping everybody out during all this.

Moderator 1:09:51

So if I'm if I'm hearing you correctly it sounds to me like they were not only open to feedback from students but they actually acted on.

48 1:10:00

Yeah, yeah, they didn't just like listen to us and say like thanks for your input, and that is right, they did something about it. Right.

Moderator 1:10:08

Okay, and 47 that that was not your experience.

47 1:10:14

The. Well, I guess what specifically were you asking, was that my...

Moderator 1:10:21

Did-- did you do you have a sense so if I heard 48 correctly. Right. He was very pleased with the fact that his university not only encouraged students feedback and gave them opportunities for feedback but they actually acted on it, and adjusted their policies based on some of that feedback. Do you have a sense of whether or not your university, did a good job bad job in that regard.

47 1:10:46

I'd say a bad job because...um, 48 What surprised me by what you said is that your university like hosted the meetings on you-- or they posted it to YouTube and then like that made everybody available to reply to it.

48 1:11:01

Yeah, they did live streams on YouTube.

47 1:11:04

We were just kind of like updated on the gist of the meetings and it was usually through emails that were like, eight or more paragraphs long that were super vague and never told us any concrete information on on Reddit like students make fun of it all the time like we get lovely daily emails from our chancellor, with a whole lot of nothing. I'm sure they asked people what they could do, like I'm sure they asked students what they could do to do a better job at like being a university in this thing, but they never like made it public. What they were actually doing. It was just kind of. We just had to trust that their best interest in mind. Right, with the, with the pass fail petition thing, we had a similar petition the University of REDACTED students signed. And once I got enough of. Once I got enough support they only extend the deadline like a couple of days instead of like 10 days after finals like for you. Whoo. So, it's almost like they, they just gave us like 30% of what we wanted.

Moderator 1:12:21

I see, okay, well thanks for sharing. If you could roll back the clock. What do you wish your university would have done to help to better help students be successful. During this online learning 47 what, what do you wish the University of REDACTED would have done.

47 1:12:47

I really don't know because it's a really tough position to be put in to be a university with 50,000 students or more. And

Moderator 1:12:57

Ok

47 1:12:58

Yeah and it's not like they could just refund students for not offering a quality education anymore, because then like the university wouldn't be in business anymore.I don't know it

Moderator 1:13:13

Okay, well that's...

47 1:13:14

Sorry I don't really have concrete feedback for that.

Moderator 1:13:16

No problem. No problem. Thanks. 48 what I mean you've already had a lot of praise but if if there was anything else that you think they could have done better what what might that be.

48 1:13:28

I wish we had like a more direct representative from the university to speak to the student body. It's like our, our president john Gable. We all love her like she's very popular at the university. She's not very active though like we don't see her all that much. I wish during this whole coronavirus thing instead of getting emails every once in a while like we also got those just like ended and they were always vague. I wish they would have like somebody just post videos to YouTube for the students of the university or something. And just like give us periodic updates. Instead of having to go into the region meetings and listen to them and figure it out for yourself. I wish somebody would just come out and give us like a clear update like hey this is what's going on this is what we're thinking, and not just leave us in the dark, because they did kind of do that they kind of just left us sitting there waiting and wondering what they're going to do, and I wish they just give us more clear updates.

Moderator 1:14:32

Right. Okay, great. Well thank you guys. We're just going to close with one more question, which is, Is there anything that you'd like to mention. Anyways ways that your ability to be successful in school was affected, that we haven't discussed. Anything else you'd like for us to record while we're here. 48?

48 1:14:55

Um, I would like to say, Are you a professor Moderator?

Moderator 1:14:58

I am.

48 1:14:59

I would like to say, um, for your online classes I would recommend opening up a discussion board of sorts, where students can post questions to either you the TA or other students. That was something that I really wish that my classes would have had and none of them had it. And I feel like that would be very helpful to have some sort of discussion board.

Moderator 1:15:23

Great. Thank you for that. Appreciate it. 47 anything that we haven't discussed that you'd like to mention?

47 1:15:32

I just wished the university would have been more transparent. And like, I don't know, keep us out of the dark but better.

48 1:15:43

Yeah.

47 1:15:44

Other than that, I think I've touched on everything.

Moderator 1:15:47

Okay. All right, great. Well, that is the end, I want to thank you guys once again. I really appreciate your help with this research project, and hope you get through the rest of the summer and next fall. As unscathed as possible. Whatever that means. So thanks again and. Oh, hey there's someone waiting in the waiting room. Oh, I bet anyway thank you. We'll see you later.

Emailed responses LS White Men

SURVEY ID 6

Overview

The COVID-19 pandemic has presented big challenges for undergraduate students. As you know, universities across the nation have closed, moving all instruction online with very little time for students or faculty to prepare. Many students have encountered challenges related to the closing of campuses, relocation, and the movement to online instruction. In this study we want to learn how the closing of campus and the pandemic in general has affected you. We want your opinions about the good and bad, on how things went as well or didn’t, what your professors and universities did that was helpful and unhelpful, and what you wish they would have done.

**Let’s start by discussing where you went when your campus shut down. I’d like to hear from each of you.**

First, I’d like you to tell me about the space in the home you moved to. What space did have to do your school work? Was this space private or shared? If shared, who you shared it with.

**Fortunately, I go to a community college so I am dependent on my family so I had a shared space to fall back on during the strenuous times.**

What challenges did you encounter attending classes or doing your schoolwork in this space? Could you tell me about why this was challenging for you?

**Motivation was a big risk from the rapid transition because the lectures were prerecorded, which I wouldn’t want any other way… however it would delay my attention and approach topics at a much slower rate. Overall, I adjusted to the schedule.**

What are things that you, or other people in your house, did that made it easier for you to attend classes and do your school work?

**To have an appropriate internet connection was the most valuable. The next most important factor was a space to study and attend lectures without distractions. I also enjoyed the asynchronous aspect of**

If you live with siblings, to what extent were there differences in how much parents supported each of you in your ability to attend classes and complete schoolwork? For example, were some kids in the house given more time, space, and consideration to do school by parents than others? Could you tell me more about why you think it was alike or different?

**I live with an older sibling and she just graduated as a physician assistant, which was stressful for her. She needed to find a job as an essential worker, but lacked the experience needed in stressful times. It was different because she needed to get a job, meanwhile I needed to stay home. I don’t have younger siblings.**

**In our next set of questions, let’s talk about what other responsibilities you had *after* your campus closed and how these differed from responsibilities you had *before* campus closed.**

Let’s start with care-taking responsibilities. Could you please describe your care-taking responsibilities? This can include childcare, eldercare, or caring for ill loved ones. Please describe how your care-taking responsibilities after the shut-down were alike or different from your responsibilities before the shutdown.

**My father started to feeling some symptoms towards the end of March, with a loss of appetite and taste. He felt fatigued for nearly two weeks and on/off. I cared for him the best I could, but I wasn’t familiar with such a low-state in him.**

Now let’s talk about house-hold responsibilities and how they changed before and after the shut-down. Please describe your other household responsibilities such as chores, cooking, caring for pets during this time and how they were alike or different from before school closed.

**I definitely had more responsibilities, there was so much more cleaning to be done around the house. Each trip meant I needed to disinfect everything in sight. I was able to cook more and experiment with new recipes which was a positive effect.**

After the shut-down, to what extent were you expected to take on more household work, childcare, and/or family caregiving responsibilities than other people in your home? For example, parents may expect more of one sibling, or partners and/or roommates might not contribute equally.

**Although I am not head of household, I resumed with my daily routine of chores that needed to be done around the house. Whether it be vacuuming or doing the laundry, I did my efforts and continue to contribute. My sister held more expectations, but I would collaborate with her if need be.**

**Thank you all for sharing. Now let’s talk about how your academics were impacted by the shutdown. I will ask you how the shutdown has impacted a variety of things. If you don’t think it impacted something I ask you about just say “no impact.”**

In what ways if any has the shutdown impacted your academic performance (like your grades) and your understanding of the subject matter of your courses?

**No impact**

In what ways, if any, has the shutdown impacted your career goals? (follow-up: could you tell me a little bit more about why they have changed?)

**I was supposed to shadow physicians for five weeks in Spain, right now. I can’t do that. I was supposed to research on my campus and gain experience in clinical trials, but I can’t do that either. I was also elected on the executive board for a club, but that’s on pause. I had an initiative that was making great progress, until it had to be put on “pause”.**

In what ways if any has the shutdown impacted the availability of other learning opportunities such as internships?

**Specifically, in my situation as an undergrad interested in health science lacking valuable certifications, opportunities for internships in healthcare setting is just so difficult right now, it is a high risk.**

Did new learning and/or career development opportunities emerge during the shutdown and if so, what were they?

**No impact**

In what ways if any has the shutdown impacted your confidence in your ability to successfully complete your bachelor’s degree at all or in a timely fashion?

**I was able to take a summer course which actually sped up the process.**

In what ways if any did the campus shut down impact your finances?

**No impact**

**In our next set of questions, let’s talk about what your professors did and did not do during the move to online learning.**

What are some examples of strategies, tools, or technologies that your professors used that you found to be very effective (in that they made it easier to learn) during online learning?

**Pausing the lecture when losing focus and coming back to it at a better time (pre-recorded lectures)**

What are some examples of strategies, tools, or technologies that your professors used that you found to be ineffective (in that they did not help you learn) during online learning?

**Discussion boards.**

What are some things your professors did during online learning that made you feel like they cared about their students?
**Extensions on assignments and a range of hours for assessments.**

What are some things your professors did during online learning that made you feel like they didn’t care about their students?

**Keeping exam dates in place during the worst of it all, but who is to say they weren’t reprimanded? Also, threatening to using webcam/microphones during exam “to combat cheaters”, that seems violating.**

**In our final set of questions, we would like to know more about your university. Let’s talk about how your university responded to the move to online learning.**

What are some things that your university did (e.g., pass/fail options, townhalls, remote counseling) to help students be successful during online learning? Mention any resources the university provided or actions they took to help students be successful or feel supported.

**Pass/fail options, remote counseling, and club meetings online.**

What do you wish your university did to better help students be successful during online learning?

**They’re doing the best I can, but if I have to install any program to watch me while I do my homework/exams/assignments I will absolutely be uncomfortable.**

In general, are there any ways that your ability to be successful in school was affected by the pandemic that we have not discussed?

**Other than delay of goals, no impact.**

**That is the end of my questions. Thank you for participating in this focus group.**

**High SES White Men**

Moderator 0:04

We're recording now I just saw it pop up. Okay, here we go. So let me let me read again. Welcome. Welcome to this focus group. Uh my name is Moderator. I am a professor, a researcher at Redacted. Uh our note taker today will be Note-taker, whom I work with on an almost daily basis. Um before we get started with the questions and everything, let me just run through the the sort of ground rules for everybody. So we will be asking a number of open ended questions. There are no wrong answers, but people may have different points of view and that's totally fine. Please feel free to share your point of view even if it differs from what others have said. Keep in mind that we're just as interested in negative comments as positive comments. We want everyone to feel comfortable participating. We encourage everyone to talk. The only thing we do have to ask um is that we don't talk over each other so that everybody can have their voice heard. Um because we have to keep this to an hour, we may need to cut you off if you're in the middle of something. We have a lot of questions to get through. Um and we have, we're going to have four, hopefully five people show up. So I apologize in advance if I have to keep things moving along but please understand that this is not because we're not interested. It's just that we have to keep our our schedule. Um right. So what else? We want to get to the full breadth of experiences and opinions. Um but some of you may have very similar experiences or opinions. And in that case, if if someone in the group has already answered a question and your answer would be very similar, feel free to just say my answer is the same as Joe's or Sam's or whoever you're you're following. Uh finally, it's difficult for us to take notes on everything that is said in this group, so we will be recording this session so that we can transcribe it later. It's very important for you to to know though that we will not associate your name with anything that is said in this focus group. Everything is 100% confidential. It's also important for you to know that you can refuse to answer any question or you can withdraw from the group at any time if you're feeling uncomfortable about anything. Um we will ask all of you to respect each other's confidentiality and not repeat things that were said within this group uh to anyone outside of this group. And now I will open the floor and ask if there are any questions before we get started. Seems pretty straightforward, I hope. All right. Great. Um, so let's just first of all, go around. uh and everybody just tell us two things. Please, what year of college did you just finish? Did you just finish your first year, your second year, whatever. And if you could describe in one word, what life has been like during the pandemic, I'd love to know what that word is. So 7, maybe I'll start with you. What year did you just finish and one word description of life during the pandemic?

7 3:25

Okay, yeah. Uh so I just finished up freshman year at REDACTED. And I would say my one word would just be pretty boring (laugh).

Moderator 3:34

I'm I'm guessing you're not the only one that feels that way.

7 3:37

Yeah.

Moderator 3:39

5

5 3:41

Yeah, so I just finished out my freshman year at REDACTED and the one word I would use to describe is choppy

Moderator 3:49

Choppy. Hm, okay. 10.

10 3:55

Uh, so I just finished my uh junior year at the University of REDACTED and I guess the one word I would use is pretty boring.

Moderator 4:03

Pretty boring. Yeah. And 13 sir.

13 4:08

I just finished my freshman year at REDACTED and definitely pretty boring.

Moderator 4:13

Definitely pretty boring. Okay, well, I'm again, glad all of you joined us, um you're going to get a chance to tell us more about the boredom and the choppiness and all of it. Um but let me again just read from our script so that everybody uh has the big picture in mind. So the COVID-19 pandemic has presented big challenges for undergrad students. As you know, universities across the nation they closed, they moved all instruction online with very little time for students or faculty to prepare. Many students encountered challenges related to this closing, related to relocation, movement to online instruction. In this study, we want to learn how the closing of campus and the pandemic in general affected you and we want your opinions about the good and the bad on how things went, as well as what your professors and universities did that was helpful or unhelpful, and what you wish they would have done. So with that context set, let's start by discussing where you went when your campus shut down. So I'm just gonna cycle through uh all of you and call on you one at a time, if you don't mind. Uh to sort of cut down on the lag time. So 7, after the shutdown, where did you move to?

7 5:36

So I went to REDACTED it was just where I grew up.

Moderator 5:40

Okay, and is this is this like your family home or an apartment complex or what?

7 5:47

Yeah, it's a family home.

Moderator 5:48

You move back in with your family.

7 5:50

Yep, you got it.

Moderator 5:51

Got it. Perfect. 5, where did you move to?

5 5:55

Yeah, I just moved back to my family home in REDACTED.

Moderator 6:00

Okay, and 10.

10 6:03

Uh I went back to my family home in REDACTED.

Moderator 6:07

REDACTED. Okay. And 13,

13 6:10

I went back to my family home in REDACTED.

Moderator 6:13

All right. All right, very good. Um so I'd like to know a little bit more about um the space in which you lived. Uh so specifically, could you tell us more about the space you had in your home to do your schoolwork? You know, was there a designated office? Were you in a bedroom? What was the space where you were doing your schoolwork? And specifically, if you could talk about whether that space was private, or if it was shared? And if it was a shared space, uh with whom you shared that space? So 7, what was what was the space you had to do your work like?

7 6:55

So I actually worked right here. This is um, it's like our home gym, but my sister and I set up desks here. So yeah, that answers the other question. I worked with my sister. And yeah, we're pretty much in here like nine to five doing schoolwork and...

Moderator 7:09

Gotcha, gotcha. So you had a a sort of office, if you will, but it was a shared office space between you and your sister.

7 7:17

Exactly. Yeah.

Moderator 7:17

Perfect. 5?

5 7:21

Yeah, I already had a desk in my bedroom so I just worked in my bedroom, and it was pretty private. And all I do was put my laptop up, and I could just like, work, pretty comfortably.

Moderator 7:31

Okay, great, 10.

10 7:34

Uh, yeah, so there's sort of a play room, uh office type room in my house that isn't in use anymore so I set up one of those, like, uh fold out tables in there and I've just been using my laptop and it's been pretty private. Uh for the most part.

Moderator 7:50

Okay. Um could you say a little bit more about when it wasn't private? What what sort of non privacy issues would come up?

10 7:58

Um, so my dad's work was considered to be an essential business. So he was at his office for the most part, but since this is sort of the office room, whenever he'd be at home doing his work, he'd be in here as well. So that would be the biggest disruption I have is him doing like conference calls next to me.

Moderator 8:15

Oh, right right. Okay. Yeah, that would be disruptive wouldn't it. 13. Sir, where were you working?

13 8:22

I just brought my laptop down to my kitchen table, and it really wasn't private. But it was all right. Um uh my brother would pop in my mom, dad and my dog barks a lot sometimes.

Moderator 8:34

Okay, so it was you said it was the kitchen table?

13 8:37

Yeah. Just in our kitchen.

Moderator 8:39

I see. So that's a pretty community space.

13 8:41

Yeah.

Moderator 8:42

People and dogs apparently would be moving in and out of it. All right. Got it. Great. Thank you very much. Um what challenges did you encounter attending classes or doing your schoolwork in this space? What were the challenges that you faced 7?

7 9:01

I think the biggest thing was just staying on schedule. Like at REDACTED, you get the whole environment and you go walk to math at nine o'clock, everything's super rigid. But this is more you just kind of wake up whenever you want, you watch recorded lectures. So that was the biggest challenge, just staying like on task and sticking to a schedule.

Moderator 9:20

I see. Um so that certainly is a big issue. Would you say that there were any issues about getting your work done in that space, though? Like, was it difficult to share that space with your sister? Or did that work out pretty well? What would you say?

7 9:32

No, I would say it worked out pretty well. One of us usually just laughed if we had to talk in the class.

Moderator 9:36

Gotcha. Gotcha. Okay. 5.

5 9:39

Uh so yeah like the biggest issue was like, I had to take this one class where we had to have this, like, um pretty in depth software. And my my computers at home aren't really the most powerful. So like the program would like crash or just be difficult to load, and like upload.

Moderator 9:58

Yeah. So you had tech tech issues.

5 10:02

Yeah.

Moderator 10:03

Okay. 10.

10 10:06

Uh, I'd say the biggest challenge for me was probably scheduling because I had a linear algebra and differential equations so I was doing basically daily math assignments and work through that. So I basically got like a to do list app to help me sort of sort through the workload because otherwise I just let it pile up to the end of the week.

Moderator 10:27

Okay, okay. Um so, would you say that in terms of the space you were in though, like did you find having to share that space with your dad when he was on teleconference or whatever, like, do you feel like that was part of the challenge? Or do you really feel it was mostly just scheduling and staying on task?

10 10:46

Uh I'd say staying on task is probably the biggest part. Once I'm like working, I can stay focused and I don't get distracted, but just making myself actually wake up before noon and start on my math assignments was probably the hardest part.

Moderator 11:00

Yeah, yeah. Okay, wonderful. Thanks. 13.

13 11:04

I agree with 10. Um similar thing, like, just staying motivated to do your work and to get everything done and not just procrastinate until the last minute.

Moderator 11:14

Yeah, yeah. Yeah, that's very understandable. Um so what are the things that you or other people in your house did that made it easier for you to attend classes and/or to do your schoolwork? So like 10 just mentioned an app that helped him keep track of assignments and classes is that am I understand that correctly? Okay. So there was an app that helped you, um but that's sort of what we're wondering here. What sort of things did you or other people in the house do that made it easier for you to attend your classes? 7? I think when everyone else went to work and was focused, it was pretty easy to do the same. Because pretty much doing the work day my parents were working in their offices, and then it was just everyone was just doing their thing. So there wasn't too many distractions going on that made it hard to do work. Okay. All right, 5,

5 12:11

I'm probably my my dad has like a PhD in physics, and I had like physics and math classes. So whenever I needed like, help on a question, I could just ask him and like, he just knew how to do it. So that was a help.

Moderator 12:23

You had a personal tutor.

5 12:25

Yeah.

Moderator 12:26

Right. Very nice. Very nice. 10, was there anything else other than the app that you'd like to mention?

10 12:33

Um, probably just the non school related stuff. So like figuring out meals and like activities to do outside of the schoolwork would be sort of handled by like my mom or other people in the house. So I could, I wouldn't have to worry about like, what I was going to get for lunch or dinner because that would already be handled for me.

5 12:51

Hm so you felt like those other uh life tasks because they were taken care of sort of off your plate and you could focus on school.

10 13:00

Yeah, they basically just helped me focus more on my schoolwork.

5 13:04

Got it. Got it. All right, 13.

13 13:06

Uh my family set like strict schedules with them. So they basically were out of the house for the most part when I was doing work, either like working themselves or doing like exercise classes and stuff like that. So that helped me have a quiet environment and get my work done.

Moderator 13:22

So having plenty of sort of alone time, if you will.

13 13:25

Yeah.

Moderator 13:27

Yeah. Okay. Right. Got it. Uh that's great. Thank you all so much for sharing. I appreciate that. Um our last question along these lines is about siblings. So if you don't live with siblings, this one won't apply to you. Apologies in advance. Uh but if you do live with siblings, the question is, to what extent do you feel there were differences in how much your parents supported each of you, you and your siblings, in your ability to attend classes and complete schoolwork? I mean, so for example, were some kids in the house given more time, space, consideration to do the to do their schoolwork by parents than others? Do you feel it was pretty equitable? What was the was the differences if any, between you and your siblings, 7?

7 14:16

I thought it was pretty equitable. Uh we both had a pretty similar setup here. So.

Moderator 14:21

Yeah, right. Okay. 5?

5 14:24

Yeah like my brother is in high school. And I don't think like the teachers did like as good as job transitioning to online. So they were like, they were like on him more to like get his work done than me.

Moderator 14:37

So you felt like you had a bit more freedom? Or maybe you you didn't receive as much oversight um from your parents as your younger brother?

5 14:46

Yeah.

Moderator 14:48

Okay, great. Got it. 10.

10 14:52

Uh so sort of similarly, I had uh my younger siblings. I have one in high school and one that was in middle school. Um and my parents sort of just trusted me to stay on top of my own tasks. So they would not give them more resources, but just uh make them be more on top of their tasks than they were for me. But I was fine with that, because that's the the best way that I work is just to be left to my own devices. Sort of.

Moderator 15:20

Got it. Got it. Okay, great. Thanks. And 13.

13 15:24

I'm the only one of all my siblings who's still in college. So they didn't have like any work to do like that.

Moderator 15:30

I see. So you have no one to compare to in this regard.

13 15:33

Yeah.

Moderator 15:33

Got it. Got it. All right, great. Thanks. Um okay, so now we're gonna shift gears. Um I'd like to talk about other responsibilities you may have had after your campus closed, and the thing we're sort of getting at here is how those responsibilities might have been the same or different. Um so specifically, could you describe for us uh what caregiving responsibilities you might have had? Whether it was child care or elder care or caring for someone in your family or a close friend who was who was sick, caring for an ill loved one? Um and again, really what we're interested in is whether there was a big difference uh between those caregiving responsibilities before or after the shutdown. 7?

7 16:28

Yeah, I think any difference I felt was pretty small. I mean, it's just the feeding the dog, making meals again, rather than just picking up. So pretty minor stuff.

Moderator 16:37

Okay. Okay. But you did have a little bit of uh pet care increase. Is that correct?

7 16:44

Yeah.Yeah. A couple other distractions besides school going on.

Moderator 16:47

Okay, but nothing that you feel was a major detriment?

7 16:51

No, definitely not.

Moderator 16:53

Got it. Okay. 5?

5 16:55

Yeah, I just had to like feed my cat once in a while but that takes like two seconds, so.

Moderator 17:00

So again, not a not a major difference.

5 17:04

Yeah.

Moderator 17:04

Okay. 10?

10 17:07

Uh yeah, I'd say the only minor difference was whenever my dog would bark I'd have to let him out to go to the bathroom. But outside of that it was basically nothing.

5 17:16

Yeah, yeah. Okay, 13 any differences or no?

13 17:21

Just pets. that's it.

Moderator 17:24

Hi. I'm afraid I didn't hear what you said though. Well,

13 17:29

Just pets, like letting out pets, feeding pets.

Moderator 17:32

Okay.

13 17:32

That's really it.

Moderator 17:33

So it's amazing. All four of you sounds like you're in basically the same boat. You have no pets at school to take care of and the only difference was that you'd have an occasional barking dog or or need to feed them or something like that. That's very good. Thank you. Um I have a similar sort of question about other household responsibilities, maybe chores or cooking. Uh how would you describe the difference between your household responsibilities like chores and cooking before and after the shutdown? 7?

10 18:13

Yeah, before the shutdown on campus I pretty much had no like cleaning responsibilities. I just lived in a dorm room. So there was minor things like unloading the dishwasher, you know, cleaning the kitchen after dinner, um cleaning the bathrooms every once in a while. So that was all new after the shutdown, but again, nothing major.

Moderator 18:31

Okay, okay. 5?

5 18:34

Yeah like there were like a few more chores like instead of like like I had to cook more because that there aren't because like the peop- or college didn't like prepare the food for me anymore. And like, I had to like mow the lawn, shovel snow, just like minor things, but like nothing that was like that had cost a lot of time.

Moderator 18:55

Okay. Okay. 10?

10 18:58

Um, so I'd say I probably had fewer chores coming back home because I was an off campus apartment. Um and that like right before the shutdown, I had to clean a lot of stuff and I was helping uh my girlfriend move out of her apartment and everything was sort of really hectic. So once I got back home was actually a lot easier for me. Um and then I just do like occasional cooking or like cleaning of dishes but all that happened after classes, so it didn't interrupt me at all.

Moderator 19:26

And and compared to living on your own off campus, you felt like this was a decrease in your domestic duties, so to speak.

10 19:34

Oh, yeah, absolutely. It was it was a lot easier.

Moderator 19:37

Okay, got it. 13?

13 19:40

Uh I just had a little bit of an increase with like mowing grass and dishes and just the small things, but that didn't really cut in my time for schoolwork at all. Okay.

Moderator 19:50

Alright. Great. Thank you so much. Um let's see where am I? So oh, I'm sorry, I forgot what this question was but now I remember. Um so it's sort of similar to the the one we just talked about with differences. Um to what extent were you expected to take on more household work, childcare, family caregiving responsibilities than other people in your home? So for example, sometimes parents may expect more of one sibling um than another sibling or have roommates or or something like so if you compare your household work, childcare, family caregiving responsibilities to other people in the home, would you say that you did more or less about the same what how would you characterize it? 7?

7 20:42

I would say about the same. Uh my parents went pretty easy on us just because they knew we were end of the semester pretty deep and want to make sure we succeeded.

Moderator 20:49

Yeah, yeah. Okay. 5?

5 20:52

Yeah I'd say it's about the same like everyone in my family was just like, working in like one way or another so.

Moderator 21:01

Okay. 10?

10 21:04

Yeah, it was about the same for me too between my siblings.

Moderator 21:07

Mhm, mhm. 13?

13 21:08

A little bit less actually, because uh I was doing classes and my older brother wasn't. So he was just off of work because he couldn't go back. So they made him do more stuff around the house while I kind of got to just do schoolwork.

Moderator 21:21

I see. Oh, I see. So I I think I remember now you were the youngest. Is that?

13 21:26

Yes.

Moderator 21:27

So you have uh elder siblings. And so when they had no work to do, and you're still taking online classes, then other things got transferred to your siblings. Is that what you're saying?

13 21:36

Yeah.

Moderator 21:37

Got it. Got it. Okay, great. Thank you very much. So, we've talked about where you went. We've talked about what your, you know, responsibilities um at home might have been. Now we want to talk about how academics might have been impacted by this shutdown. So I'm going to ask you how the shutdown impacted a variety of different things. Uh i f you don't think that the pandemic impacted something, just feel free to say no impact, and we'll we'll move right on along. So in what ways, if any, has the shutdown impacted your academic performance and your understanding of the subject matter of your courses? 7?

7 22:24

Yeah, I would say it's slightly decreased. Uh I mean, definitely learning in person where you get to talk to the professor and classmates is, I'd say that's ideal, and the online learning isn't, but it's still manageable.

Moderator 22:36

Mhm. So a a small small decrease is that?

7 22:39

Yeah, I would would say small decrease.

Moderator 22:40

Okay. 5, what would you say about your academic performance and your understanding of the subject matter of your courses?

5 22:48

Um four out of the five classes I took were lecture classes. And those actually, and like the crisis actually like, helped me in those classes because I was actually like, like, go to office hours, which I wasn't able to do before the crisis, and like talk to the professor and just do the work when like it was more convenient for me. But like for the other class, it was like, it was more like a group project class where we had to work with people and like the lecture was live and that like decreased.

Moderator 23:22

Hmm. I see. So it depended on the class.

5 23:25

Yeah.

Moderator 23:26

I see. Although it's interesting that you, you say in four out of five of your classes, you were actually more available to go to office hours, than you were in person. Did I understand that correctly?

5 23:39

Yeah, it was it was mainly one one class, but it was very helpful.

Moderator 23:43

Okay, great, great. 10, what would you say about academic performance and understanding of your courses?

10 23:50

Uh, I'd say for my like, harder math based courses that actually made it a little bit easier to learn the material because uh we were basically given we wouldn't have zoom meetings, we'd have just uh pre recorded lecture videos. And so that was really nice because I could pause it whenever I wanted to take notes. And uh I could do the material immediately after I watched the video at my own pace. Um so I I felt like for those classes that made it a lot easier, but for um the more non quantitative courses I was taking it made it a little bit harder because I wasn't able to talk with the professor. And I also felt like I focused on those courses less because they felt less difficult.

Moderator 24:32

Mhm mhm. Interesting. So again, it depended on the course. 13, what was your experience?

13 24:39

Again, it depended on the course. Um, for with what 10 said with like, the more um like math based stuff, I thought that was easier because you could go through it like again, if you didn't understand it the first time. But for like a class like for me chemistry, it just got really like boring. And since normally in class, we'd be doing experiments and other things, but now we weren't. We were just listening to someone talk. So it was pretty boring.

5 25:08

The boredom was hard to take. I see.

13 25:09

Yeah.

Moderator 25:10

Can I'm curious, though, um in your chemistry class, were you able to go back and rewatch a lecture or pause or something like that if you didn't understand it or was that not an option in chem?

13 25:21

You could, but they were all pre recorded. So when you went back and watched it, so if you didn't understand it before, like, chances were you weren't gonna understand it again, because it's just terminology. Like math you could, you could figure out the problem, but with terminology, if you don't know what the word means, and you have to like look it up, and it takes a while to do all that.

Moderator 25:39

Gotcha. Gotcha. Okay. Great. Thank you very much.

Um in what ways, if any, has the shutdown impacted your career goals? 7, has it impacted your career goals in any way?

10 25:54

No, no, I I don't think so. No, in fact.

Moderator 25:57

Okay. 5?

5 25:59

Um like yeah I'm trying to get like a summer internship for 2021 and I had a feeling this like took away some internships because like we're in a recession.

Moderator 26:09

Right. Right. Okay, 10?

10 26:13

Uh, yeah, so I was a junior. And so I was preparing for a summer internship and I had an offer that got rescinded and I had other final round interviews that sort of just went like, belly up when the companies just canceled their internships. And then um additionally, I'm in actuarial science. So for that, we have to take professional examinations and you have to go to these prometric testing centers and register that and like, do that online and their servers got so slowed down. And for a while, uh I couldn't find a testing center within like, a three state radius of me. So it became like impossible for me to basically get the professional exam that I wanted, but eventually it quieted down and it was more possible for me to get the uh exam registered that I needed.

13 27:03

I see. So it it certainly had very severe short term impacts. But do you feel like the pandemic has had any impact on your sort of long term career goals?

10 27:18

Uh long term? I'm not sure because I'm applying for insurance companies. So obviously, health insurance is getting pretty heavily impacted by COVID-19. But it's, it's really hard to tell at this point. I I don't know if it'll be a net positive for health insurance companies or not. But ultimately, I can go into any other type of insurance and like car insurance isn't really being affected by COVID-19 right now.

Moderator 27:43

I see. Okay. 13, any impact on your career goals?

13 27:47

No impact.

Moderator 27:48

No impact. Alright. Great. Thank you. Uh in what ways, if any, has the shutdown impacted the availability of other learning opportunities such as internships? I guess 5 and 10 have already spoken a little to that. Uh but 7, do you have a sense of whether this um pandemic impacted your availability to other learning opportunities? Or intern-

7 28:15

Yeah I actually I think I'm in computer science and I don't think it was too impacted. Um just hearing from some of my friends and the companies that I interviewed at, it sounds like most of their internships carried out through the summer online. So

Moderator 28:27

Yeah

7 28:27

I think it's not impacted my field.

Moderator 28:29

Okay. And just for the record, I'll just restate that 5 you mentioned that this you were negatively impacted by by this. I remember hearing that correct?

5 28:39

Yeah. It just seemed like there was like less to apply to too.

Moderator 28:44

Yeah. Yeah. Alright. And 10, you had also said that you had a bunch of opportunities dry up this summer?

10 28:51

Yeah, I'd say aside from the opportunities drying up, it's been well for studying for exams, it was actually really helpful because I could just do that full time at home, and it didn't really matter because I had nothing else that I could do. And then also I can go at my own pace and learn, like programming or do work like that. So it's in in that respect, it's been a positive but uh in terms of the opportunities it was largely negatively impacted me.

Moderator 29:18

Yeah, yeah. 13.

13 29:22

Um, so I'm a bio major, so a lot of the stuff I do do is, um like, outdoor field work, where you're kind of like alone. So it didn't have that much of impact.

Moderator 29:31

Uh huh. Uh huh. Okay, very interesting. Thank you. Um, did you did did new learning and or career development opportunities emerge during the shutdown? And if so, could you tell us more about that? Did any new opportunities arise? 7?

13 29:51

No, not that I'm aware of.

Moderator 29:53

Okay. 5?

5 29:56

Um like one thing that was helpful was like I was taking like, a math class and like, I wasn't doing the best in that class. And, and I wasn't able to talk with my professor just because I had classes during his office hours. And then like when the crisis hit I could like actually talk to him during his office hours, because like he changed them just because the curriculum with the videos and his office hours change, so I could actually make them so that was helpful.

Moderator 30:25

Yeah. Yeah. All right. 10, any new learning opportunities arise that you're aware of?

10 30:31

None.

Moderator 30:33

Okay, and 13?

13 30:34

None.

Moderator 30:35

None. Alright. Alright uh. Let's see, where are we here? Uh in what ways, if any, would you say that the shutdown impacted your confidence in your ability to successfully get your bachelor's degree uh at all or in a timely fashion? How how is your confidence to get a degree impacted if at all, 7?

7 31:00

I would say it was probably boosted. Um I got a little worried when we got shut down just because my schedule was thrown off and everything, but just having the ability to do all these classes remotely, I think just increased my confidence because if this continues more, we can clearly do it. We've done it before.

Moderator 31:16

Hm I see. I see. So getting through the first uh initial, like shock, sort of increased your confidence to deal with whatever comes in the future. Is that what I'm hearing?

7 31:30

Yeah, you got it. Exactly.

Moderator 31:31

All right. 5?

5 31:33

Yeah, like I think it had like pretty much no effect. Like I got all the classes done. I learned everything and I'm just like, it's like, bam, just a step closer to getting that my degree.

Moderator 31:46

Okay, 10 any impact on your confidence?

10 31:50

Uhh I think it helped my confidence a little bit just in regards to the fact that I could choose a pass/fail option for classes that I didn't want to affect my GPA. So it made it really, really easy for me to get a 4.0 for that semester. So that definitely helps.

Moderator 32:06

Okay, so you I see, so this this ability to choose which classes to get your, what was it? You said a pass/fail is that?

10 32:14

Yeah yeah , so you you either take it and it would weigh in your GPA or you can take it for a pass/fail, and a lot of the classes that you would need, like 70s in before they let you do the pass fail and get a 60 in. So some of the prereqs were even easier to get than they were before.

Moderator 32:28

I see. And so this boosted your confidence in the in a stellar GPA when you graduate. Got it. Alright, 13.

13 32:37

I think the pass/fail was what helped with confidence because it gave you that little, like leeway in case you do stray like you're not as focused and then hopfully you pass the class.

Moderator 32:48

Okay, great. Thank you. Um let's see. What's our next question? Next question. In what ways if any, did the campus shut down impact your finances? 7?

7 33:03

I would say it didn't impact them.

5 33:05

Okay, 5, any impact on your finances?

Um, it didn't, but I'd like didn't really, I was expecting only come home for like a week. So I brought like a week's worth of stuff. So like, all my stuff was at college, so I didn't really have access to my stuff. That was a mess.

Moderator 33:24

Right. 10 any impact on your finances?

10 33:28

Um well, obviously, there was a pretty big opportunity cost of missing the internship. Um and then outside of that, I still had the lease that I have to pay at my apartment. So it just sort of negatively affected me in that way. But I didn't see any sort of like, accounting sort of negative finance. It was just the opportunities that I missed that cost me.

Moderator 33:49

Mhm. But, and I don't mean to press too much here, but finances were a big issue for lots of students. And when you say you had to keep paying for your lease, like that wasn't a financial drain for you?

10 34:04

Um well I'd already planned on having to do it. So it wasn't anything unexpected for me.

5 34:08

I see. And you you had the the reserves to take care of that?

10 34:12

Yeah.

Moderator 34:12

Gotcha. Gotcha. Okay. 13, any impact on your finances?

13 34:17

None.

Moderator 34:18

None. All right. Okay, next uh topic is... What is my next topic? Oh, yes, your professors. So now we'd like to hear um about what your professors did or did not do during the move to online learning. So what would you say are some examples of strategies, tools, technologies that your professors used that you found to be very effective during online learning? 7, what what did you find effective that your professors did?

10 34:56

Yeah, the two class styles I liked was some would do live lectures and then posted afterwards so you'd have the opportunity, or you'd have the option of viewing it live or later, and others would post the recorded lecture before class. And then they would open up class for like an office hours session where you can just talk to them live and get questions answered. I think both of those were effective.

Moderator 35:17

Mhm mhm. Okay. 5?

5 35:20

Um I like the one when the professor's just posted a lecture video. So when they had office hours they you could just come and talk to them if you had any questions or concerns about the content.

10 35:33

And so just to be clear, when you say office hours is this like, uh what 7 was mentioning that the office hour would have been during normal class hours?

5 35:43

Uh yeah, yeah, that that was helpful. But this pretty much like it doesn't matter if it was during normal office hours just so long as he, like he had office hours.

Moderator 35:54

I see. I see. So you just liked um those who did pre recorded lectures. And then you could go to their office hours as needed to get questions answered or whatever.

5 36:06

Yeah.

Moderator 36:07

Got it. Okay. 10, what did you find effective that your professors did?

10 36:13

Uh I liked the pre recorded lectures, which is what all of my professors did. Um and then, uh like 5 said they'd have like the the zoom office hours or whatever. Um I think a lot of my professors started getting frustrated because most students wouldn't go to the zoom office hours, and they felt like they were wasting their time just sitting on the zoom screen for an hour or whatever.

Moderator 36:37

Yeah, but you went, uh you took advantage of this?

10 36:41

I didn't, but I- I that was what I felt was effective for me, but I felt like they had a lot of frustration.

Moderator 36:49

Mhm.

5 36:50

Yeah, one thing that like helped with that is it's like I'm taking a math class where like, you actually have to like, like sign up to office hours through like starfish. Like the professor isn't just sitting there waiting for like a student to show up.

Moderator 37:05

Hmm, I see.

5 37:06

So that might...

Moderator 37:08

Im sorry?

5 37:09

So so that can help with the problem.

Moderator 37:12

Uh huh. So the professor would have some like a heads up if you were going to come and then they didn't have to stick around if nobody sent him a heads up. Is that what you're saying?

5 37:21

Yeah.

Moderator 37:23

Got it. Alright. 13, what did you find that your professors did that was effective?

13 37:27

Um I liked when they, um like, posted their class at the same time as it normally met, because it like it helped me personally keep on a schedule. And I also liked one of my um history professors he had like, really good enthusiasm, and he'd go on campus like walking his dog and stuff and send us like a live video of him doing that during the lecture. It was just really cool, because it helped us all stay motivated and ready.

Moderator 37:53

(laughs) Wait a second, just so I can wrap my head around this. Your instructor livestreamed walking a dog?

13 38:02

Yeah, so he'd have his dog with him and he'd go to like a statue or a like a monument in in REDACTED. And he'd kind of just tie it into our lesson. Like it was a history class. So it's pretty easy to tie in.

Moderator 38:13

Oh okay. I'm sorry I was I was imagining a math teacher walking their dog (Laughs).

13 38:18

No

Moderator 38:19

This was actually relevant to the course you were taking.

13 38:21

Yeah.

Moderator 38:22

Got it.

13 38:22

Like, one time we went over foods, and he dressed up as uh Heinz ketchup. So it just helped everyone stay happy, I guess.

Moderator 38:31

Hmm. I see. I see. I see. So that that sounds to me like a a personality. Um that person's personality was effective in keeping you guys engaged and interested and so on. They did.

13 38:45

Yeah exactly.

Moderator 38:46

Alright. Wonderful, wonderful, great example. Okay. Um let's see what's next? Oh, yeah. So the next question then is what would you say are examples of strategies, tools, technologies, that your professors used that were not effective in helping you learn? 7, what did not go so well?

10 39:09

Yeah, nothing comes to mind at the moment. Um I'll interject though if I.

Moderator 39:14

Sure. Yep. If and by the way, if if somebody says something that sort of joggles a memory, just feel free to chime in and say, oh, yeah, that happened to me too. Okay.

7 39:24

Okay.

Moderator 39:25

Great. 5, what was not effective for you?

5 39:27

Um, not all of my professors had office hours. So it would have been nice if like, every one of my professors had office hours.

Moderator 39:36

I see. So then I I'm curious, does this mean that some of your professors just canceled office hours all together?

5 39:46

Uh I- I think so.Yeah.

Moderator 39:49

Okay. Okay. Alright. Yeah, that certainly would be very ineffective, wouldn't it? 10, what what didn't go so well?

10 40:02

Um, I can't really think of anything that they used that wasn't effective, because there was really only the pre recorded lectures and the zoom office hours. And outside of that it was basically just email the professor or use the like online homeworks or whatever for review.

Moderator 40:20

Mhm

10 40:21

Yeah, I can't think of anything that was ineffective.

Moderator 40:25

Okay, 13, anything ineffective?

13 40:27

Um, one of my professors like they switched their lecture. So it was the department head who actually was doing the pre recorded lectures. And he stopped doing the lectures, I guess. And I just couldn't like hear the lady who was talking. She was very quiet. So that was really the only thing.

10 40:45

Oh, but yeah, that's kind of a big one. So you're watching a pre recorded lecture and the audio just was inaudible.

13 40:54

Yeah, for like I had to, like when I did have my parents home at that time, and I was listening like they had to be completely quiet or else I wouldn't be able to hear.

10 41:03

Ah, yeah. Okay, that's a big that's a big problem, I would say.

Moderator 41:08

Okay, um, let's see my next question. Oh, yeah. So my next question is um sort of a connection, a personal connection one. So what are some of the things that your professors did during online learning that made you feel like they cared? Like they cared about your success or about you or about how you were I don't know healthy? Like, what did professors do that made you feel like they cared? 7?

7 41:37

Yeah, I remember being in office hours for my math class and it was like three or four of us just talking for like, five minutes about totally unrelated stuff. But just kind of chatting with the professor about what's going on in general. I think that helps get a community vibe going.

Moderator 41:53

Mhm, mhm. 5, anything?

5 41:58

Yeah I I took one online class that like shouldn't have been affected much because of the virus because it was already online. And like my professor emailed me when like, this whole thing was happening and was just like, "hey if you need anything, just talk to me like I care about like your health, like mentally. So I just want you to be like, um as comfortable as possible". And that was just like, reassuring because it showed that the professor like cared. And was willing to help us through this time.

Moderator 42:28

Right, so just an explicit message that they cared about you.

5 42:32

Yeah.

Moderator 42:32

Okay, great. Thanks. 10. Anything your professors did that made you feel like they care?

10 42:39

Um, I felt like um well my math professors would post uh I think one of them posted like a review before the final or something like that. And I think they also laxed up on some of the grading policies. So um like I said, some of the classes were uh pass/fail that I was taking, so I ended up not having to take the final because they didn't make it a requirement anymore because my grade would already have been high enough anyways, but outside of that, I don't think there were any things that they necessarily did that showed that they cared outside of just making sure that the lectures were up and that they were answering emails on time.

Moderator 43:19

Right. Okay alright. 13?

13 43:21

I had the one very enthusiastic professor, which helped. Then another professor I had um, whenever like every two weeks or so she had like an optional zoom meeting where people in the class because the class was online so we didn't really meet. Uh we'd just talk about how stuff is going and if everyone's staying healthy and stuff. So that was cool.

Moderator 43:42

I see. So that was not, it wasn't um about course content, or instructions. She was just, it was a she you said?

13 43:50

Yes.

Moderator 43:50

It was a she she was just hosting this sort of get together like a coffee so to speak where you could talk about

13 43:57

Yeah

Moderator 43:57

Got it. Where you could talk about anything. Okay, wonderful, great example. Thanks. Um well, let's ask the opposite question. Do you have examples of things that your professors did? Again, during the online learning that made you feel like maybe they didn't care. 7?

13 44:21

I remember, they brought in a guest speaker in my ag class. And the guy was talking about how he would be surprised if next spring was in person. And it didn't seem like he didn't care, but it just it really killed the mood. The fact that he is so pessimistic about the future, I mean, whether it's true or not, it just didn't really need to be said.

Moderator 44:42

Hm. Okay. All right. 5?

5 44:47

Um it was just like some professors like obviously put more effort into the transition than others. So like the ones that didn't put as much effort just like seemed like they didn't really care about it as much.

Moderator 45:00

Mhm. Can you? Can you give me uh an example maybe of what it looks like when an instructor wasn't putting in the effort versus when they were? How would I recognize that so to speak?

5 45:14

Well, it depends on the class, but for like my lecture classes, like the teachers were, like, available constantly and like would like, have like, the videos up and stuff on time, and like, make sure that you would know the content and someone who didn't or wasn't out would like not have office hours, and the content wasn't like as fluid.

Moderator 45:39

Mhm mhm. It was just sort of clear that some people were putting in more effort into this online instruction. Is that what you're saying?

5 45:46

Yeah. Yeah.

Moderator 45:48

Got it. Thanks. 10. What would you say?

10 45:52

Um so this is sort of answering the last question because I forgot to say something, but it it would just be some it would be more of like a comparative thing. So you would see some professors who'd put in a bunch of effort and then others would just sort of put in the minimal um but I didn't really blame them for that because uh REDACTED was really bad about Coronavirus. And they basically told the professors up until like 5pm before spring break that we were going to not close for Coronavirus and they kept extending it and extending it and they never gave them time to really prepare. But I had I had one professor go through so much effort that he made a YouTube channel and then animated himself. And there was so much work put in it that he even animated like the physics on his dreadlocks would move when he was like his character was speaking on screen. So it it sort of made the other professors look uh bad by comparison.

Moderator 46:50

Right. Right. So you so in your experience, there was a very clear difference between some professors who were putting in tons of work and others who were just sort of doing the minimal to get by.

10 47:04

Right, exactly.

Moderator 47:05

Yeah. Okay, great. 13.

13 47:08

I think all of my professors like put in effort, like even the one whose lectures got changed from him doing them to um another lady doing them. He still would send us like emails and stuff about, um like quizzes coming up and course material and he'd still be there for office hours.

Moderator 47:25

So you don't feel like, or rather, let me put the positive way you feel like all of your instructors uh were doing their best and caring about the success of students and so on?

13 47:36

Yeah.

Moderator 47:37

Okay. Excellent. Great. Thanks. Alright. Well, that brings us to the last sort of topic block of questions. Um now, I want to talk about how your university responded to the move to online learning. And here I need to distinguish between what your professors did and what your university did. We're really now asking what your university did. So what were some things that your university did, for instance, and some of you mentioned already, they went to a pass/fail option. Some universities had big town halls, like what were some of the things that the university did to help students be successful during online learning? 7?

7 48:24

Yeah, I think you mentioned the pass/fail, which is super awesome. But also just having like advising appointments virtually, and mock interviews, virtually. Just moving all the services that they had before making them still available I think was great.

Moderator 48:36

Uh huh. I see. So um so those are two interesting ones. So you were still able to meet with your academic advisors.

7 48:44

Correct.

Moderator 48:45

And what was the other one you mentioned?

7 48:48

Uh mock interviews .

Moderator 48:49

Mock interviews. So they were online? Uh I don't know. I guess zoom. You can do a mock interview and that's what you're referring to?

7 48:56

Yeah, exactly.

Moderator 48:58

Got it. Got it. Great. 5, what what did the university do?

5 49:03

Um they like made zoom accounts for everybody, which I know isn't the easiest thing to do. But that was like very helpful. And if we didn't have zoom, I don't this whole transition would have been a disaster.

Moderator 49:17

I see. So the fact that they helped every student get set up with this online platform that was very helpful for you.

5 49:24

Yeah. And like they made sure that this online platform was actually work like I was like doing like a WebEx one the other day and that thing was just so choppy that.

Moderator 49:36

It's important that what you transfer to works.

5 49:39

Yeah.

Moderator 49:40

Yeah. Okay. Great. 10, what, what did your institute institution rather do?

10 49:48

Right, so I go to the University of REDACTED and uh I sort of mentioned this earlier, but we went the pass/fail option was the big thing because that that is a lifesaver and a grade saver and my favorite thing that I've ever had in my three years of college. Um but I'd say outside of that, uh REDACTED made the transition for students and professors pretty hard because they really waited until the last second because like I said, they waited until 5pm on the Friday before spring break, to say that spring break would be extended a week. And then somewhere during spring break, they said, well, actually, we're just going to go online for the rest of the semester. So they really made it really, really last second for a lot of people and I know a lot of people who left a lot of stuff in their apartments or dorms or whatever, that they couldn't get back. And there were a lot of people who had to purchase flights back to REDACTED because there's a ton of out of state students. And so it it made it made it really hard on the the students and then they also didn't really prepare any of the professors for zoom and stuff like that. So it was it was a tougher transition.

Moderator 50:57

Yeah, yeah. 13, what would you say uh your institution did to help students succeed?

13 51:04

Definitely the pass/fail. And I also liked how uh flexible they were because I know, at least REDACTED switching 40,000 plus kids to online classes is not an easy thing. And I personally didn't have those problems. I thought it went pretty well. And they saw what other schools were doing and they did the same thing if it worked, and they planned it pretty well.

Moderator 51:26

Okay. Alright. Excellent. Um well, let me see. I guess uh 10 has already sort of answered this question for us. But of course, 10 when we get back to you, if you have more to add, please do. But what would you say you wish your university did differently to help students be successful during this online learning? What what could REDACTED have done better? 7?

7 51:57

Yeah, nothing nothing really comes to mind. I think they did the best they could and I wouldn't have any suggestions.

Moderator 52:02

Okay. They're going to be very happy to hear that, by the way. (laughs) 5, what are your thoughts?

5 52:09

Uh, yeah, I think there was like no way to have a perfect transition. So what they did can't really ask for much more.

Moderator 52:16

No, nothing you can imagine would've made your success uh better?

5 52:22

No just the timing was really convenient because like we kind of went home for spring break and never came back. So there was just like some buffer time to like prepare.

Moderator 52:31

Right. Okay, 10 anything to add that you didn't already mention?

10 52:36

Um sort of like I said before, it was really last minute to the point that I had a 4pm uh lecture and we were telling the professor before he knew that we were going online. And apparently that day, the dean had told all the professors that they're not going online and not to prepare. So there was about a week and a half where we just didn't have assignments because there was a a big buffer. I think it was like a week after the extended spring break that stuff didn't get back on schedule. So it it was really slow.

Moderator 53:06

Hm. I see. 13, what are your thoughts? What could your institution have done better?

13 53:13

Um, I think REDACTED ended up switching to online while we were over spring break. So the only issue was we couldn't really get our stuff and like my roommate, he lives up in like REDACTED. So he wasn't able to come back until like, way back to get his stuff. So we had a we had a day where we could go up and like we had like 30 minutes to get as much as we could. And I had that. But then he wasn't able to get this stuff until the beginning of summer, like after the school year is over.

Moderator 53:40

Mhm. Right.

5 53:42

Yeah. And I never got the email for that so would have been nice if I got the email.

Moderator 53:48

You never got the email saying when you could come and get your stuff out of the dorms?

5 53:53

Yeah.

Moderator 53:55

I see.

13 53:56

I never got an email too. I had just scheduled myself like they didn't really give us a date. They just said, like online and said, hey, if your state is in like yellow phase or something, you're allowed to go out and get it.

Moderator 54:08

Wow. Yeah, that makes it tricky to schedule doesn't it? Alright well, thank you so much this has been really wonderful. I just got one more very general question for you. Um are there any ways that your ability to be successful in school was affected by this pandemic that we haven't discussed? Is there anything we haven't touched on that you'd like to tell us about?

7 54:34

No, no, I don't think so.

Moderator 54:36

No, 5, anything we didn't think of that you'd like to tell us?

5 54:42

Um, I, the pandemic wasn't much of a problem for me, like academically. I think now since I'm a freshman I only have lecture classes. But if I was like a senior and I had like more group projects and more like um just like the senior engineering classes where like it's more like, interaction. I feel like that would have been more affected in terms of my education.

Moderator 55:10

Yeah, yeah, I think I think I hear ya in those other classes. It would have been rougher. 10, anything we didn't bring up that you'd like to mention?

10 55:21

Hmm hmm. Except for the fact that I had like maybe two programming classes, uh which were already basically online and really easy to do uh I don't I don't think there was anything that affected me. I I think it that the classes I was taking were really conducive to an online environment. So it made it pretty easy.

Moderator 55:43

Got it. Okay, 13.

13 55:45

Uh the only thing I can think of is going into the next year, like I heard, I don't know how true this is, but a lot of stuffs gonna be closed, like, um, gyms, rec facilities. So for me at least, it's just like, well, how's that going to impact us like socially and stuff? Are we gonna be in our rooms the whole time? Or who all is coming back to campus? All that stuff.

Moderator 56:07

So you're still having some um anxiety around the uncertainty of the future you mean?

13 56:13

Yeah.

Moderator 56:14

Not knowing what to expect.

13 56:15

Yeah, just well, I don't want to go there and just sit in my room and do classes all day. I want to be able to go out and live the college life.

Moderator 56:22

Right, right. Alright. Well, thank you so much. Um I really appreciate all of you taking the time and your input is super valuable for the research project we're working on. And if you have anything else that uh you know comes to mind afterwards you have our contact information, and otherwise, this is it. So thank you very much. I appreciate you guys all have a wonderful night.

**Low SES Asian Women**

Moderator 0:12

Okay, awesome. So welcome to our focus group today. My name is Moderator. I'm a research coordinator from Redacted. Today, our note taker will be Moderator. Moderator is over there in the corner. Moderator do you want to turn on your camera and just wave to everyone? But you can also leave it off if you want. So today we're going to be discussing a number of open ended questions. There's no wrong answers, but um everyone may have different points of view. So feel free to share those points of view, even if it differs from what others have said. So we're just as interested in negative comments as we are in positive comments. And we want everyone to feel comfortable participating. So we encourage everyone to talk. But because we need to keep this an hour, I may have to cut you off. And so I apologize in advance. If I need to need you to wrap up your thoughts so we can move on to another person, I'm just going to raise my hand like this. We want the full breadth of experiences and opinions from everyone today. And so you may have really similar experiences to someone else. If that's the case, you can just say my answer is the same as Sam's. It's going to be difficult for us to take notes on everything that's said today in the group. So we're going to be recording this session to transcribe it later. We're not going to associate your name with anything that you say today in the focus group. In addition, you may refuse to answer any questions or withdraw from the group at any time. And we understand that confidentiality, confidentiality and privacy is really important. So we're going to ask everyone to keep everything private today in this group and not repeat it outside. Um are there any questions before we get started? Alright, so first let's just go around and we'll have each person say their name, what year in college they are, and what life has been like for you during the pandemic. So let's start with um 20.

20 2:16

Um it's pronounced 20.

Moderator 2:21

Okay.

20 2:21

Yeah. I'm a current sophomore at REDACTED University and I in during this experience its been like crazy adjusting. And just like being at home again, because like, whenever I'm at home during breaks, I don't want to do anything but as a result of this pandemic, I had to like force myself to to do like all the work and just like concentrating, like, get it done and everything.

Moderator 2:46

And so if you could summarize that in one word, what would it be?

20 2:51

Uh hectic.

Moderator 2:53

Hectic. Okay, and so is it 15?

15 2:58

Uh I go by 15 or 15. Either is fine. I am a rising junior at REDACTED. Um, and so it's one word. Um probably just. It's been interesting. It's stressful, but definitely interesting. So I don't remember there was anything else you wanted or.

Moderator 3:20

Nope that was it. And so I see is it 28?

28 3:28

Yeah, 28. So um it's been really stressful for me because I'm an international student. So I'm originally from REDACTED. And right when the pandemic hit, all the flights were canceled and everything so I'm living with my boyfriend's family right now. And it's been just a crazy um transition to from like in person to online. So it's been really busy.

Moderator 3:55

And is busy do you is that the one word you would describe?

28 3:58

Yeah, like hectic and busy.

Moderator 4:01

Okay, and so I see 30?

30 4:07

Uh hi.

Moderator 4:07

Describe the pandemic is that in one word.

30 4:11

Um, it was challenging to make the move. Like at first I was excited, like, you know, having like maybe a few weeks off from school it'd be like less stressful. But I realized that sooner or later that actually caught up to me because relying on the professors on their, on their zoom sessions and whatnot, it was really hard to make the transition like fixing your study habits, and working on like family schedules, because you know, you're tasked with something you don't realize, like, how much stress you have in school. So it's like, so it's hard to make the transition.

Moderator 4:39

Okay. So, um, stressed would be the one word that you would use to describe what life has been like in the pandemic?

30 4:48

Uh, yeah, I guess so.

Moderator 4:50

Okay, and 21, so we uh are describing what life has been like for us in one word during the pandemic.

21 4:59

Um I'd say lonely more just because of social distancing and not being able to see friends as much and uh college being canceled and all of that. So, yeah, lonely.

Moderator 5:14

Okay. All right, so let's go ahead and get started into the bulk of our focus group discussion questions today. So, as everyone has kind of mentioned, the COVID-19 pandemic has presented some really big challenges for undergraduate students. As you know universities across the nation have closed, moving all instruction online with very little time for students or faculty to prepare. Many students have encountered challenges related to the closing of campuses, relocation, and the movement to online instruction. So today, we're going to in this study, we want to learn more about how the closing of campus and the epidemic has in general affected you so we want all of your opinions like whether they were good or bad, things that went well or didn't go well, and what your professors and universities did that was helpful or unhelpful and what you wish they would have done. So let's start today by discussing where you went when your campus shut down. And so I'd like to hear from each of you and we'll just go in the same order that we started in. So 20, could you um tell us where about the space in the home that you moved to? What space did you have to do your schoolwork? And was this space this space private or shared?

20 6:26

Luckily, I was able to go back home in REDACTED, in my small apartment with my family, fortunately, and the workspace that I have is currently y'all are on my uh beautiful desk right now. So I have like a little like work area in my room that I have dedicated to doing like schoolwork and other computer stuff. And yeah, that's about it.

Moderator 6:50

And would you say that space is fairly private?

20 6:53

Uh no, not really.

Moderator 6:55

Okay. And so who do you share this space with?

20 6:58

I share this space with my mom.

Moderator 7:00

Okay, and 15, so tell me about the space in the home that you moved to.

15 7:07

Like, what like my room or like where I do my work? Um.

Moderator 7:11

yeah, so the space in the home that you moved to. So where you do your schoolwork primarily.

15 7:16

So I'm kind of in like, an open area, it's sort of like connected to like the living room. So it's definitely like shared. Um, the and there's like, they'll be like, people walking through and I'll have like, I can hear the TV and such. It's been kind of gross, to be honest. But um it's kind of like I have to do this because that's the only place in my house with like, a decent table and like the Wi Fi doesn't like reach far enough to my room. And so it's yeah, that's kind of what it's been.

Moderator 7:45

Alright and do you share this space with a lot of other people in your family?

15 7:50

Um, so my mom will like, be in the same room sometimes when I'm like doing my work and we also have a roommate and he'll like walk in and out and so it's, yeah, it's definitely shared.

Moderator 8:01

Okay, and 28 so what about the space in your home that you do your schoolwork? Is it and is it private or shared?

28 8:08

It is um shared so I had an apartment at the university um that I was living in. But um it's a small college town so everything shut down. So my boyfriend's family lives in REDACTED, so it's like a big like a suburb. And they fortunately have the office room that I can like peacefully work in. There's not many distractions, but I share it with my boyfriend and his dad whenever he has to work from home.

Moderator 8:38

Thank you for sharing. And 30?

30 8:42

Um, well, we, yeah we have like a, I have a lot of siblings. So every time I try sit down do any work upstairs I share my room with my a couple of my sisters and so if I ever sit down on the bed and try to do work, there's always distractions and whatnot. So um my share my space that I made was like going downstairs to one of my brother's rooms, because they're always at work or something so my space is when they they aren't home. And it it is private to an extent because I can hear the other noises from outside cuz, you know, when one person goes downstairs, everyone else will also come downstairs. So it's kind of like for a for a couple hours if I really need it they'll they'll leave me alone so I'll have that kind of space. And they know sometimes they'll be distractions.

Moderator 9:24

Okay, and 21.

21 9:27

Um, so as soon as, like, around the time when uh campuses were shut down, I kind of just stayed in my apartment on campus. Um, all my roommates moved out and everything so it was just me there for a couple for two months. And then like after finals and stuff, I went back home. So now I'm home and I'm, I've been like, I have my own private space just in um my bedroom. Because I have a desk there uh but it's uh. Yeah, it's like there's other I share the space with basically my whole family so people will like walk in and walk out and all of that um while I'm working.

Moderator 10:14

Okay, and so you all have touched on this a little bit but I want to know what challenges you encountered when you were attending classes or doing your schoolwork in this space. So we can start in the same order. So 20.

20 10:28

The challenges that I faced during uh remote learning of the pandemic was like trying to concentrate and doing my schoolwork because there would be like external noises happening in the living room. And and since, I live in an apartment like it's really small and cramped. So like that noise travels all the way to like my room unfortunately. And um other than the concentration, trying to like stay connected with my professors, because they would always have zoom office hours during their time during like, the time that we had our like class. Let me give you an example. Like I had my math 141 class at 9:05am and my professor would have uh would have office hours during that time but unfortunately like I couldn't wake up that early like because like my like my school life and my home life is like really different. So I couldn't like wake up like that early to like get help so like just trying to like stay connected was like another challenge for me.

Moderator 11:30

Alright, thank you for sharing and 15.

15 11:34

So I think it's definitely just the area being shared having been like a really big problem like in the middle of class, um because we'll be I'll be having class and we'll um have be having discussion and I could hear like my mom in the background talking on the phone and I just feel like a little self conscious about turning on my microphone and such because like I it's like, I know that you can hear her ranting in the background. Um but also like, I'll be in the middle of like taking an exam and um either like the TV will be on or um someone will come like and ask something and it's like, I feel bad because I have to like snap at them. Like I'm in the middle of an exam right now but like, so it's definitely just mostly the distractions that are the problem.

Moderator 12:13

Okay, and 28?

28 12:17

So yeah, sometimes when I'm working people will interrupt me just talk to me and everything. And because it's not my family, I feel like sometimes like, you can't just like say that, what, like you can't tell them to um that you're like really busy or something. So that's been a problem. But other than that, I think just um the being able to balance everything because like, uh my home life, and my school life, I usually like to keep it separate. So I've never been able to study in one place. I usually go to the library or a coffee shop. So just being stuck at one place to do everything has been really like stressful.

Moderator 12:58

Okay, and 30.

30 13:01

Um, yeah, I would say that distractions is definitely one big thing. Like when you're trying to do your work and then everyone's just like, oh, we're cuz because, you know, uh most of my siblings, they aren't in college now they're like in high school, middle school. And so it's it's like easier for them to just like, not study or not focus, because their classes are a lot more lenient. So they don't understand whenever I have to like take hours to like study for an exam or something because they're always trying to include me in their plan. And so like, just the distraction itself has been like the worst like, trying to also because like, I would get easily distracted, just like wanting to join their plans and then like, just like, tell myself, oh it's okay, I'll just study at night around 3am or whatnot, but they won't like always, like pan out. So it's like, trying to keep yourself focused and stay away from everything.

Moderator 13:44

Okay, and 21.

21 13:46

I think for me, because I was in the apartment, like on campus or off campus, um when everything was like when I was still in classes and stuff. So my big thing was, I like to study in different areas, at cert-, like I can't just sit in one place and study. Um so that was definitely one big thing because I like I tried to move around the apartment and find different places to study. Like I would move coffee tables closer to the futon and stuff like that. Um but it just didn't really work that well. And then also uh technology because like in we're college students we chose like really cheap Wi Fi that would just like barely work. Um so then Wi Fi problems and then also like finding printers and um all of that because the like campus was shut down. So we had to like, I don't have a printer in my apartment. So that was like I had to walk a couple miles to go to this um place that had printers open and stuff. So yeah, it it was definitely technology and location issues.

Moderator 14:57

Okay, so we just talked about the challenges but now I want to know a little bit more about was like what happened in your house? Or what did the people do in your house that made it easier for you to attend classes or do your schoolwork? 20, could you let me know um what people did that might have been helpful?

20 15:16

So, basically my, my mom and my brother didn't like my whole entire family didn't enter in like my room like during the day. So like I could have like a dist- a distraction free learning uh environment and um they wouldn't like they wouldn't like talk to me but like they would often bring me dinner while I was doing work. So that was like very nice of them. And my brother he he was a second semester high school senior so his like, year was like, caught up short, unfortunately. But uh my brother was always like a good person for me to like talk to talk like my feelings to, even though he's like a boy. So that was like a positive in like my environment.

Moderator 15:59

Okay, and 15.

15 16:01

Uh could you repeat the question?

Moderator 16:03

Yeah. So was there anything that anyone did in your house um or were there things that made it easier for you to attend classes and do your schoolwork?

15 16:13

Not really, to be honest, um, like, if like it it was too loud sometimes like I'd asked my mom to like turn down the TV so and she would be willing to do that, but like, she wouldn't like, want to, like go elsewhere or anything. So it's kind of um like she would bring me like what 20 said that she would like she would um come and like bring me food occasionally. And so stuff like that, but overall, like there wasn't really anything.

Moderator 16:40

Okay, and 28.

28 16:43

Yeah, like 20 said that they bring me coffee and food whenever I need. And I have this this is supposed to like only office space for his dad, but I'm allowed to use it and as much as I want whenever I want. They have printers and scanners that I can use. So that's been really helpful for me. And um I think just being able to talk to my boyfriend about my stresses and what deadlines I have, it's been really nice.

Moderator 17:11

Okay. And 30.

30 17:15

Um I'm trying to think like, I guess they've been helpful in the aspect where they do give me hours where I can, like if I'm like really stressed out, and like, I tell them like oh, I really cannot be bothered today. Because one of the besides distractions one of the other problems was that my uh, my fath- my father, my grandparents or whatnot they always have something to fill out something, an appointment to go to and whatnot, and they always rely on always comes down to me um for for me to accompany them somewhere. And so if I, if I'm really like, um adamant about like, oh, I really need this time to be um studying or something before the um exam finals, they would understand they would listen to that. And um I do have uh a sister to talk to and what and I do have friends to talk to whenever it feels really stressed out. Like I can FaceTime them. Like, just like, just tell them everything. And then you know, it just feels like a weight off my shoulders until the next day comes. I have to go through it all over again. But yeah.

Moderator 18:07

Alright and 21.

21 18:09

Um, so I was living alone at the time. So I think one of the bigger benefits of just staying on campus or in that area where I usually um stay for the rest of the school year was just that the environment was kind of like I'm used to studying the apartment and on campus in that area. So it was like, it was helpful because it felt like I was still going to school in a way. Um, so yeah, I would definitely say the environment was one of the like just being on campus was really helpful.

Moderator 18:43

Okay, so if you live with siblings, to what extent were there differences in how much your parents supported each of you in your ability to attend classes and complete your schoolwork? So for example, where some kids in the house given more space or time or consideration? 20.

20 19:01

Um my parents treat me my brother and I equally so like we had, like he had his own space to work and I had my own space to work. So you we were treated more um equally but at the same time my parents like gave me like more leniency because I was a college student. And I had and I was like taking like a physics class a math class. So those are like really tough, rigorous courses, unfortunately. So like they gave me like more leniency whereas with my brother, he took like AP Physics and AP calc, like his senior year, which are tough classes, but at the same time, they're not as hard compared to mine. So they gave me like, more time or just like they like gave me more slack compared to him.

Moderator 19:45

Okay, and 15.

15 19:48

I'm an only child, so didn't really have that.

Moderator 19:50

Okay. And then 28 you said you're with your boyfriend.

28 19:54

Yeah. So I don't have any.

Moderator 19:57

Uh 30. Were you with siblings?

30 20:00

Uh, yeah, I do. Um, I mean, my parents uh I mean, like I said before, like, it comes down to me to do a lot of like, on the appointments and filling out the forms or whatnot. I mean, um we do have we, when I say when I think about responsibilities, a lot does come down me and one of my other siblings, even if even though I do have uh homework and whatnot, but if I do if I'm vocal by like, oh, I can't do this right now. Can you, can we do it, can we give it to the other one? Or can we give it to like someone older than me to do it? Sometimes it works out sometimes it doesn't like I just got used to it. It's fine.

Moderator 20:33

Okay. And 21.

21 20:38

Um, so I was living alone when I was doing like courses but now that I'm home, I have two younger siblings and they um for like most of June and stuff they are like, not in college yet. So they still had classes and stuff going on. Uh so what happened is like they each had different rooms that they would do classes in and my parents kind of just knew our schedule. Like they knew when I would be busy. They knew when uh my two sisters would have classes and stuff. So they would kind of just like leave us alone for those amounts of time. Um but there wasn't really ever any, like, this person should be treated higher or better than the other person kind of thing.

Moderator 21:23

Okay, so in our next set of questions, um we want to learn more about kind of some of the responsibilities that you had after your campus closed in comparison to what before your campus closed and how those might have changed. So let's start with caretaking responsibilities. Could um everyone let us know what kind of caretaking responsibilities they had? This can be things like child care, elder care, or ill loved ones and how your caretaking responsibilities may have changed before or after the shutdown. And if they were how they were like and how they were different.

So 20. 20, did your camera freeze? Okay, let's move on to 15 just to keep things moving. Could you just let me know how your caretaking responsibilities may have changed? Um if you didn't have any you can just say they were this is not a relevant question.

15 22:24

Yeah, this isn't relevant to me. I didn't have any caretaking responsibilities.

Moderator 22:28

Okay. And 28. Did you have anything that changed for you? Okay.

28 22:32

No I didn't have any caretaking responsibilites.

Moderator 22:35

Okay, and 30?

30 22:38

Uh yeah, um, honestly though, uh, before college shut down, I would purposely spend more time on campus to avoid like responsibilities responsibility uh placed on me until the weekend. And but now that without campus being there, I'd have like I can't leave to, I can't leave to campus. So now without campus being there, I'm at home 24 seven, so much of the care- my caretaking responsibilities for my parents um and my siblings have gone up. Like I said before, like, accompany them to appointments, filling out um forms, and like, just just doing grocery shopping going out for the family. Yeah, it did increase in my aspect.

Moderator 23:16

Okay. And could you tell me a little bit more about why they increased? Was it just because you were just physically present at home?

30 23:23

Yes. Most definitely that because I was just physically present and so if they just saw me there, they just like, oh, hey, you're available. Can you do this for me right now? And it's just me being more at home. And like, my father just seeing me just being there. So he like comes to me much more often for his problems. Now that I'm there.

Moderator 23:42

Okay, so 20. Uh I'm glad to see that you were able to come back. We saw your camera freeze. So um we're just talking about if we had any caretaking responsibilities before um the shutdown or after the shutdown, and if this isn't relevant to you, you can just say it's not relevant.

20 24:03

Uh this was not relevant for me and I'm I'm sorry that, that I like it might come back like the internet, my house is unstable, unfortunately. So like, there's like some issue, so I apologize for that.

Moderator 24:15

Oh, there's no reason to apologize. You know, if you need to keep going in and out, we'll work through it. And 21.

21 24:24

Um, so when I was at home, or like when I'm at home right now, um they've definitely increased just because my internship also got canceled for the summer. So I had like, nothing really to do. Um, so my parents like know that and they expect me to like, um keep my other sisters engaged and like, play with them and stuff like that. So just to yeah, just like spend more time with family again.

Moderator 24:56

Okay, and so let's talk about some other responsibilities have you have like, so household chores, um and how they changed before and after the shutdown. So these can be things like cooking, caring for pets, um and how these are alike or different from before and after the shutdown. So 15.

15 25:21

So before um was my own dorm and so it was kind of like, I just had to, like, do whatever. Like I could decide like how messy it like I would be willing to be and such like that. And now that I'm home, it's like, um so my mom will like often tell me to like how I have to, like clean this or like do this um empty like the dishwasher. And so I have like, a few more house um household chores now that like, like, it wasn't as big a deal during the school year because I was like, studying like all the time, but now that's like over it's like, um, she expects me to like definitely be doing like more work and such. And also like, I do have pets at home. So um so like, I'll have to like either like, put like because we have chickens, so I got to go out and make sure like, at night that like the chickens are like in and also like feed the dogs and such. So that's just been the gist of it.

Moderator 26:11

Okay, and 28.

28 26:14

I'm sorry my um microphone had some issues. But did you ask him about chores that I had to do?

Moderator 26:20

Yeah.

28 26:21

Ok

Moderator 26:21

So how your household chores changed before and after.

28 26:25

I think they're mostly the same because I'm just doing chores for myself so laundry and then dishes and just cooking but I have been a lot more cooking than before because it's not accessible to eat out that much.

Moderator 26:38

Mhm okay, and 20.

20 26:44

Every Saturday when I was at school, I would like wipe down my dorm, I would like mop the floors and vacuum and make sure that everything was like clean and everything. And um I didn't have to do as much cooking because I was on my campus. I was on my I was on a meal plan on campus, so like, I would like go to the dining hall and like get food. In terms of pets, I don't have any pets. But um, but um ever since like coming back home I would like have to like throw out the trash, do more cooking and like, not only clean my area but like clean the living room, the kitchen and uh like everywhere, basically.

Moderator 27:23

Okay, and 30.

30 27:27

Um, I feel like because of the shutdown, uh my chores also increased, but I feel much more a lot more stressed. Because there's much more time to complete those chores like before I would have to plan around like my clubs and grocery shopping, because grocery shopping, it definitely increased now more. But now I don't feel as stressed col- completing those grocery shopping or like doing like cleaning the house or whatnot like that. And also, like, I'm taking another summer class right now. So um even that is like, a lot less stressful than being on camp- during school settings. Because where I have to like think about the commute, like it's an hour commute for me so I think about the commute and whatnot and that like kills time, but now I'm like at home so I have that extra hour too to like, do what I do need to do at home. So yeah.

Moderator 28:11

Okay and 21.

21 28:17

Um, so I kind of divide my, like, quarantine experience into two parts like being in my apartment and then coming back home. Um but yeah, so before uh Corona I like had uh three other roommates and then we would all um like contribute to like cleaning the apartment and cooking and stuff like that. But then after, like once Corona hit, um they all went back so I had to like kind of take on all of that responsibility. So I was cooking for myself every day. Um and I was cleaning the apartment a lot more often than I normally did. Um so it was it got kinda like I had additional chores to do um once Corona hit for the first part. But then since coming back home, I haven't really cooked or cleaned as much because my like sisters and my mom and my dad kind of do all of that stuff. So, yeah, my responsibilities increased a lot and then decreased.

Moderator 29:22

Okay. So after the shutdown, to what extent were you expected to take on more household work, childcare, or other family caregiving responsibilities? I know for um like 21 that might not be as relevant and neither for 28 but I'd still like to hear from each of you. So 15.

15 29:42

So, I think there definitely was like expectation for me to like, take on a lot more chores. Um, like, it's definitely not. It hasn't happened to the extent that my mom would like though um, um mainly because. But like I've mentioned, like just like taking care of like the animals that we have here um. And just like some general cleaning, dishwashing, stuff stuff like that. So it there definitely was our like expectations to like, clean up. Um I got out of mopping though, so it's okay. So yeah, sorry.

Moderator 30:21

Could you tell me? Um, so why do you think there were expectations?

15 30:26

Just just that, like the fact that like, I'm like here and physically present. Um like, um my mom's. Well, I think in general that like, I don't really know what like other households are like, but I mean, I imagine most of them are like, if there are like kids, especially like college age there, then you're kind of expected to like, go and help like, clean the house. Um like sweep the floors and help mop and such like that. And so especially with my mom, like she's really kind of traditionally Asian and so it's just, um, if you're kind of like really expected to like put in household work and chores.

Moderator 30:59

Okay, and 28 was this relevant to you?

28 31:04

Yeah, so um I'm not living with my family right now. But what 15 said is perfectly like how it is if I was back home, and I was at home, I would be expected to do a lot more chores and help out my mom with every single thing. now I'm privileged right now because um my boyfriend's parents understand that I don't have time to do everything. But with a lot of, I'm Asian as well, so a lot of Asian parents will expect you like to do chores.

Moderator 31:33

Okay. And 20.

20 31:36

I definitely agree with 15 and 28 on like, taking on more responsibilities. In addition, in addition to that, I have to like look over my brother to make sure that he did all of his schoolwork and that he was like, on top of his grades, because my brother loves to play computer games all the time, unfortunately, and my mom always yells at him. So I'm gonna and I have to be like really mean to him be like, oh you have to do your work or like please like, get this turned in. So I had to like look over my brother during quarantine, unfortunately. But yes, I do agree with 15 and 28 on like looking over and like taking on more responsibilities.

Moderator 32:14

And do you think that taking care of your brother and sort of managing that was something that was expected of you or something you sort of organically did and why?

20 32:24

It was expected for me because because you know, I'm like a girl and I have to like look over my little brother like it's just like an Asian standard really.

Moderator 32:36

Okay, and 30.

30 32:40

Um I do agree with the rest of them. Like it is an Asian side for like, um, especially as a girl to like help out the more the household cleaning and cooking and whatnot. That's what I experienced. Um what I experienced. And also to add on to what 20 said about like her siblings, I do have two siblings in elementary school and I definitely had to help them with uh their work because now that they move online, they aren't as responsible of doing their work unless they're there because they don't know how to use the computers. Or if they do, they have little knowledge of how to do so. And like, my parents expected us to have them on board. And like, just help them with everything. So just being at home itself, definitely increased our responsibilities.

Moderator 33:21

Okay, and 21.

21 33:24

Um, so yeah, when I was at or when I'm at home now, um I think it's definitely expected, again to like, take care or help around with um my siblings. But um, also like, part of it is expected but also, I kind of just don't really have anything else to do. So. I kind of do it by myself also. So it's like, half and half almost.

Moderator 33:55

Okay. So thank you all for sharing all of that. So let's talk about how your academics were impacted by the shutdown. So I'm going to ask you how the shutdown impacted a variety of things if you don't think it impacted something you can just say no impact. So in what ways, if any, has the shutdown impacted your academic performance like your grades and your understanding of the subject matter in your courses. 15.

15 34:25

I think it definitely has impacted like a lot of things like just like schoolwork and such because um you're not in person in some some cases your professors don't seem as accessible, then you might not know the material as well or it might be harder to like be motivated to study and so I think it's generally had a negative impact but it also varies like on professor. Like, I have some professors who are like really excellent, like they did a really good job of switching to online and like that way actually made my learning better. Um, because like, I was able to, like go over the material when I needed it. And so I wasn't always going to class like super tired. Um overall though, it definitely I think mostly had a negative impact on grades because I know like a lot of people were like cheating on exams and so that really really skewed the curve and then like and then to fix the curve like professors would go and make the exams a lot harder. And so I think that was just one of the bigger effects and especially because everyone like pass/fail their courses it also um it also really changed the parameters of Dean's list like, like, if you had a GPA that would like have always made it in the past it wouldn't because now you need like a 3.94 at least to get on Dean's list and stuff like that. So yeah, it's definitely had weird impacts in academics.

Moderator 35:40

Okay, and 28.

28 35:44

I think um some classes I did better than expected and some I did worse than expected, because it was depending on the professor. Um some classes our professors instead of doing exams because they thought it would lead to more cheating they assign like week long or project per 15 minute test, which I think was unfair because um I if I was in class I would have just a 30 minute test and study for that, but this way I had to write papers and like write projects to make up for those tests that was a negative impact. Others like some professors will just leave the material for you to learn whenever you want it. So that helped me too. But overall, it wasn't like, it was more coursework and less accessibility for the professor.

Moderator 36:31

Okay, and 20.

20 36:34

Um I'm gonna have to agree with 15 on terms of like, uh people cheating on the exams and like skewing the curve because unfortunately I had to um SAT, which is like our pass/fail at REDACTED, um my math class because like, the professor didn't do a good job with his lectures unfortunately and like, as and I put in as much work like as I did at school, and yet I couldn't get the grade like that I wanted. So it negatively, like my grades were like fine this semester but like they weren't great. But um it negatively, like impacted like my academic performance and just like not being present at like school and like getting IRL help like really like, it like screwed with me basically. So that was that's like what I have to say.

Moderator 37:28

Yeah could you tell me a little bit more about the cheating and costs? I know 15 mentioned it but could you tell me um more about that like how you knew people were cheating or the students who were cheating? This is all confidential by the way.

20 37:44

Yeah. So so um with Econ, I took Econ 104, which is macroeconomics at REDACTED. And so basically our professor made it like multiple choice and you could like easily and he allotted us 30, 30 extra minutes for than unusual because our tests would be roughly two hours if it was like at school, but because of COVID and like Canvas was like really acting up, weird at for some reason. So he allotted an additional 30 minutes for like clicking through the questions and like pressing and all that good stuff. So you could like easily look up the questions online via Chegg or a Quizlet. So that's how like people cheat.

Moderator 38:26

Was it fairly common knowledge that students were cheating? 15 are you nodding your head in agreement?

20 38:34

Yes. Yeah. Like if, like, people cheated, like, they're going to take advantage as much as they can. Call me a snitch, whatever you want, but like people will cheat, like to get a better grade.

Moderator 38:47

Okay. And

28 38:49

So,

Moderator 38:50

Oh go ahead 28

28 38:51

Yeah, so along with that, um, we have big class GroupMes for every class that everyone's in, and I had to leave every single GroupMe before a big test or a project, because people would just send questions or ask questions in that, and I think it was common knowledge that you could, like, people were using Chegg, but you weren't supposed to like snitch on anyone.

Moderator 39:13

Hm was it certain types of students that you noticed who were cheating?

28 39:20

Um so people who already had Chegg before, um usually what they would do is, if during an exam, you can't access your phone, so if you're writing an exam, you can't just ask someone a question in the big GroupMe. But if it's online, people can just pick up the phone and there's a big class GroupMe that they can send a question to or whatever. So I think just that I don't know what types of people but it's just more easier to cheat if you are not physically taking a test.

Moderator 39:51

Yeah, 15.

15 39:54

So like, um, I think that like, in general, you're probably find. In my opinion, I think that there's definitely a lot of cheating over like the entire spectrum, whether like students who are as good and students who aren't as good. So like, um I'm in the the engineering program and all my friends are like engineers and so like you would and they're like top honors students, and you wouldn't think that they'd cheat because you'd think that they would, like, know what they're doing except, like, there's just so much pressure I think, even in like top programs to like, get good grades, and there's a lot of like, emphasis on that. And so like, if you're, if you want it enough, if you're desperate enough, I think if you're given the chance, you probably would cheat and especially because in this situation, you know that like everyone else is going to be cheating and so like there's really no point in like, like being willing to like give up an edge that you know, everyone else will have and so it's yeah, it's pretty it's definitely extremely widespread and it's common knowledge that everyone's just cheating. Like, like in one of my classes, everyone just straight up got together and cheated like the entire class.

Moderator 40:55

Oh, 28.

28 40:56

Sorry. With the like, she brought up a really good point that if you are a person who generally does not cheat, and everything's online and you think everyone else is cheating, I think it just motivated to be cheating because otherwise you like, it's unfair that you get a lower grade because of the curves and because everyone else is getting a higher score. So I do feel that people who wouldn't cheat would be thinking that they would be unfairly treated and have lower grades because everyone else is doing what they would not do.

Moderator 41:26

Thank you all for sharing. We do need to move on to 30. So how was your the shutdown? Did it impact your academic performance like your grades?

30 41:37

Um, I forgot who mentioned the point but it does vary with like professors. I I took um I took uh four classes, well five if you count like lab and recitation, but in those classes, there are two classes where um my teacher very, like a lot more accommodating because of the shutdown, with like Internet access and like, how it is and whatnot, like how we can be affected at home like make themselves a little more available after class. Or use a lot more uh longer time extension on assignments. And then there are other professors who are just like took the shut down as like um as a way to just, like you know, just record their sessions like being just like assume that because they're recorded that we won't need any more help besides that. But I do want to talk on the uh talk on the fact that there are TAs though. Like go TAs. Like if it wasn't for the TAs I personally would have like, not been as um would not be passing as well as I would have if I the school didn't shut down. Like the TAs they would take their time on Saturdays to like give us more help, to talk to us over zoom, like go over like um more questions or practice tests and exams. So it really does vary from teacher to teacher. Um but compared to if school did not shut down, uh the classes um, I think I did better on the most part for most of the classes and then some of the classes I got, I would have liked myself to do just a tiny bit better. Yeah.

Moderator 42:53

Okay. And 21.

21 42:56

Yeah, I kind of had an opposite experience. I feel like all of my professors were really just became a lot more understanding after um the outbreak. So like they, I had one professor who canceled her final, um which was amazing and really helped. She basically or made it optional. She made an optional final. Um and then a lot of professors were like, responding to their emails quicker than they did during the school year. And there were a lot like, they were very understanding if you missed a homework, they would just um let you complete the homework and not get any, like marks off or anything. So I think that overall, like, I mean, ideally, I would have learned a lot I would have learned the material a lot better in a classroom setting, but um the professors that I had really adapted and helped us a lot. So in that sense, it kind of made um me perform better in a way.

Moderator 44:03

Okay. Thank you. Um, so has the shutdown impacted your career goals? 15.

15 44:15

Career wise, um, no, although um, I had like, I had an internship this summer that was like, going to go ahead in this company that typically like will rehire interns and actually like, hire them and give them full time employment like afterwards so, but like that actually got cancelled because of the shutdown. And so I've just been trying to, like, it hasn't impacted my goals, but it has, like, impacted like sort of my progress toward it.

Moderator 44:42

Okay. And 28 has it impacted your career goals?

28 44:47

No, it hasn't. Uh I still have my internship thankfully so it's been the same.

Moderator 44:53

Okay, and we will ask about internships specifically next but just talking about career goals. 20, did it impact your career goals?

20 45:03

Um I did not have an internship lined up so like luckily nothing. My career, it won't be affected. But however, um like because of COVID and everything I had, like a shift in mind um of what I want to do with my life like, like just seeing, like what's happening in America right now. Like, I might like go into politics with my technical background, like after I like work in the like, in the EE field for like, several like decades probably, like I might like cut into politics or.

Moderator 45:35

Could you tell me a little specifically about what exactly it is in America that impacted your career goals?

20 45:42

It's just like, where, like this administration, where the current administration is going with like COVID and everything and compared to other countries such as Germany, New Zealand, like they've all got COVID-19 under control, because of because of like their like excellent health care, testing everyone like frequently and um just like like their leaders are women obviously felt like they had like a really good plan on like tackling this virus. So like with this administration and like everything and like how polarized like politics are in this nation, like wearing a mask is apparently political, however you can potentially save the lives of not only yourself but like of other people around you who may not survive COVID-19. Like my dad has diabetes unfortunately so he, so he is at like a higher risk of of like, not surviving COVID-19 whereas, like some of my friends have healthy parents who may like tough it out with COVID.

Moderator 46:48

Okay, and 30, has this impacted any of your career goals?

30 46:55

Um, I didn't have any uh like 20 I didn't have any internships lined up um, but there was one I was I was to apply to but then they already canceled it before applications were even um due for submission. But um, I'm in the engineering field right now and I've always been like flirting with the idea of like med school and like going to pre med or something. But ever since shutdown um happen, I've been going on social media a lot more and like the app Tik Tok, and I see a lot more professional, like professional doctors and nurses and whatnot on the app, like sharing their stories about how, how hard they've come from, like, from like, low backgrounds and whatnot. And it really made me inspiring, like, encouraged me to, like, really think about switching over fields. And like my brothers, um, one is a nurse and uh one is another another one is a pharmacist and seeing them just like, you know, just come back with like, crazy stories like, oh, we have this many patients in or whatnot. It just made me re like think like, oh, maybe I want to do that. Like, do I really want to do engineering or just go into pre med field. But yeah, it's like it's like right now it's just me uh still engineering field and not being able to make the move to go to a different field for career wise.

Moderator 47:57

Okay, and 21.

21 48:00

Um yeah, so I don't really think it has changed my career goals a lot. Um I was planning on taking a gap year before any kind of um graduate school or medical school anyway, so it just, I'm still planning on doing that. So it I yeah I didn't really change any of my goals.

Moderator 48:23

Okay. So in what ways has the shutdown impacted the availability of other learning opportunities such as internships. 15.

15 48:34

So I had an internship that was like, I had a pretty good internship that was lined up. Um and like, it's just kind of gotten canceled because of COVID. And so um it's, it's um, it's sad because like otherwise, like with that internship I could have been like getting hands on experience and like, and like paid and such but like instead it's kind of just been like a summer of trying to find like, employment but um but like instead right now like I'm currently just taking courses instead.

Moderator 49:08

Okay, and 28.

28 49:12

So I still have my internship and I feel very lucky to have that because lots of my friends had theirs canceled, but it hasn't really impacted me in terms of the opportunities that have been available to me.

Moderator 49:24

Okay, and 20.

20 49:26

Uh I did not have an internship lined up so this does not apply to me.

Moderator 49:32

Okay, and 30.

30 49:35

Yeah, um I had hopes of having an internship this summer. But because that was not an option anymore, I just had to go I just like um 15 just took a course for the summer and such to to bide my time. Like, you know, just spend my time that way.

Moderator 49:50

Okay, and 21.

21 49:54

Um, so it was kind of disappointing because this was like the year I'm supposed to have like a really good internship. Like the past couple summers I've just been doing coursework and um taking classes over the summer. So I applied to like, over 70 different internships and all of them got canceled. And even I like got some like acceptance letters and stuff. So a lot, most of them got canceled before we could even hear back. Um but then I did get some acceptances and those also got canceled. So it was really just kind of disappointing because I had spent so much time and energy and like having all these professors write recommendations and they had to spend so much time and energy on like helping me try to get an internship and then everything just kind of didn't happen. So yeah, it was just that was like it was rough for me to go through all of that.

Moderator 50:56

Okay and so the next question is, did any new learning opportunities or career developments happen? And if it didn't, you can just say no. 15.

15 51:10

So, the company I originally had my internship with, so they cancelled their entire intern program. But like because they're in state, I think they actually started something with my university um that I got invited to do, which is kind of like a sort of, like work from home kind project. So it's very different from the internship and um I'm not making as much and I definitely don't think it's as valuable as internships because I, I honestly don't think I really, like learned anything, but at least it's like, it's something and I have like, like, new skills that I'm kind of sort of learning so. Yeah.

Moderator 51:44

Okay, and 28.

28 51:47

Um, I have an on campus job that REDACTED University Libraries that I like work with online courses, so they want me back next year. So I feel like that helped a little bit but uh other than that, I don't think there's anything that's different.

Moderator 52:03

Okay, and 20.

20 52:06

Uh no learning op- new no new learning opportunities arised for me.

Moderator 52:12

Okay, and 30.

30 52:15

Yeah, I don't think this uh question applies to me. Nothing also arised for me.

Moderator 52:19

Okay, and 21.

21 52:21

Um, so there's a lot of like free online courses that like um REDACTED and REDACTED and stuff are offering so since I had so much free time, I decided to take um some of those up. So I guess I did, like, somehow benefit from Corona and everything. But yeah, other than that I haven't really done anything.

Moderator 52:46

Okay, and so in what ways, if any, has the shutdown impacted your confidence in your ability to successfully complete your bachelor's degree at all or in a timely fashion and if this didn't impact you, you can just say no impact. 15.

15 53:03

I'm sorry, could you say that again? If it impacted what?

Moderator 53:07

The confidence in your ability to successfully complete your bachelor's degree at all or in a timely fashion.

15 53:13

Okay. So it definitely hasn't impacted my confidence, like, obtaining my bachelor's degree. Like I know, like, I'm going to get it, but I also like, but just with the way classes have been going and like, um, and how, like, learning has gotten like a lot harder sometimes it's like, I wonder, like, am I like, do I know what I'm doing? So like, while I know, I'm definitely gonna get my degree it's all it's just a question of confidence and whether like, I'm actually like learning anything or if I can even get like, the grades that I need, necessarily.

Moderator 53:43

Okay, and 28.

28 53:46

Yeah, I think it's really um impacted me because um recently the administration released an order saying that if universities were supposed to have online classes, then international students will have to leave the country. And um I'd lose my visa and everything. So um if my university decides to have completely online classes at any point, that would really impede my education. Um right now I do have some in per- like hybrid classes that count towards the in person requirement. So right now I'm safe, but it's still really uncertain for me.

Moderator 54:23

Okay, and 20.

20 54:25

Um coming from like an Asian background and everything uh my parents have been, like really supportive of me like paying for my books and whatnot. Um so this has not impacted my me uh obtaining a bachelor's degree however, like, I have, like I have an engin- like to let me rephrase this, but like this upcoming semester, I have an EE class and a comp eng class, which are like, which I need those classes to graduate. So like I don't know if I can successfully like, like, pass those classes if I'm not understanding the material because it's going to be over zoom, and I'm not sure my brain will connect those things together, and like later down the road, I'll have to apply that knowledge to my job someday.

Moderator 55:14

Okay, and 30?

30 55:17

Um, well, I just like started school. Like um I just finished freshman year. So for me, it was um obtaining my bachelor's degree especially in engineering, I don't, I I do think I'm going to get my bachelor's degree. But like, like they said before, how well am I learning the material? Um, definitely freshman year I learned the material a lot. I mean first semester I learned material a lot better, like fall semester. But spring semester it was more of a a give or take, like how much I learned because all it was over zoom again. And like multiple choice questions it's like, oh, you can like half you can like half do the questions and then you quit. You can like figure out the answer at the end. So it's more like I can get my bachelor's degree, but how do I have earned it like do I know the material well enough to get them is the question.

Moderator 56:01

Okay, and 21.

21 56:07

Sorry. Um, so I don't think it impacted me as much other than just like, not being able to, I just learned better in a classroom setting than um online lectures. So, in terms of understanding the material, it's definitely impacted my education but not in terms of getting my bachelor's degree on time.

Moderator 56:28

Okay, so in what ways if any, has the shutdown impacted your finances? 15.

15 56:35

It definitely made them a lot worse because like well I mentioned, like my um the internship I had was was gonna, like paid pretty well and like, on the contrary, now, it's like, I don't have that income. And so um because of that, like it had it is stressful trying to figure out like, how I'm going to like pay for schooling and such.

Moderator 56:55

Alright and 28.

28 56:57

This is not impacting my finances.

Moderator 57:00

Okay, and 20.

20 57:03

Fortunately, this has not impacted my finances either.

Moderator 57:07

Okay, and 30.

30 57:10

Um same with them. Like it hasn't impact my finances. If anything, it made it better cuz I don't have to pay too much car fare anymore, I guess.

Moderator 57:18

Okay, and 21.

21 57:20

Yeah, it hasn't really impacted my finances.

Moderator 57:24

Okay, so we just have a few minutes left. Um there's a few questions about your university and your professors that we wanted to ask. And if we can't get through all of them, we may ask you to respond to them in email, um which you can do on whenever you're available. And remember, you can refuse to answer any question. So. Okay, so in our yeah, we have two minutes left. So we'll see if we can get through one more question, which is what are some examples of strategies, tools or technologies that your professors used, that you found to be very effective, and that they made it easier to learn? 15.

15 57:59

So one thing my profess- one of my professors did that was did really, really well is that so we use like Blackboard and so we have like Blackboard Collaborate, which is kind of sort of like zoom, but like there's a way you can do it so you can make small group discussion sections with like, um like people who are in the class. And that made it a lot easier because when you're in smaller groups, like especially like, um, because when you're on like, video conference with a class, you're I think you're less likely to ask questions and discuss. And so because like, um she purposely, like made it, so we have like smaller groups and I'm with the same people, so we knew who we're talking about. You wouldn't worry about feeling-- like feeling dumb, and you could get through the problems. And so like, I think that's definitely been the most useful thing I've seen on online learning.

Moderator 58:41

Okay, and 28.

28 58:44

Um my professors hosted a Q&A session weekly that if we had any problems with how the course was going or concepts they would help us solve. So I think that was really helpful knowing that you didn't just weren't by yourself. Um but and discussion boards. A lot of professors had discussion boards that they would help with. So yeah.

Moderator 59:04

Okay, 20.

20 59:05

Uh this is a combination between 15 and 28 of using the discussion boards on like Canvas for me. And also we, uh my professors hosted zoom office hours during the week.

Moderator 59:19

Okay, and 30.

30 59:25

Um, well, for me, uh these are the, uh I mean, one of my teachers she, they uh really encouraged us to make uh group chats and whatnot and so like, at the end of each discussion, they would leave like uh a whiteboard up for us to um, to discuss and like post our post our emails and whatnot. So because of that, encouraged like the kids who usually we wouldn't like talk to each other. Just go to attend class, take our own notes, do our own homework, and just uh take our exams but now it encouraged us to like talk together and like trade homework and like ask questions and help each other out. So for that it was a plus for me.

Moderator 59:58

Okay, and 21.

21 59:59

Um as I said before, it was just like that they were really fast at responding to their emails, which helped a lot. Um and we could ask them questions.

Moderator 1:00:14

Okay, everyone. Um, so we're at the end of our focus group. We'll email you just a few more questions. Um have a great day. Thank you so much for participating.

30 1:00:25

Thank you for having us. Have a good day. Bye.

Low SES Asian Women Email Responses

1. What are some examples of strategies, tools, or technologies that your professors used that you found to be ineffective (in that they did not help you learn) during online learning?
2. What are some things your professors did during online learning that made you feel like they cared about their students?
3. What are some things your professors did during online learning that made you feel like they did not care about their students?
4. What are some things that your university did (e.g., pass/fail options, townhalls, remote counseling) to help students be successful during online learning? Mention any resources the university provided or actions they took to help students be successful or feel supported.
5. What do you wish your university did to better help students be successful during online learning?
6. In general, are there any ways that your ability to be successful in school was affected by the pandemic that we have not discussed?

Survey ID 15

1. Professors responded to emails in a timely fashion and made accomodations for issues (for example, one professor postponed a test because a lot of students had power outages). In one of my smaller classes, my professor asked us how we were doing every class. Another professor made it clear we could talk to her if we had internet connectivity issues.

4. My university allowed optional pass/fail. They also moved a lot of resources online (like Dean of Students' hours and such) and had a whole page of support services. All the cultural center staff and tutoring services moved online too. I'm not too sure of the full list since I didn't really use any of the resources (I didn't use resources much even before quarantine).

Survey ID 21

1. I didn't think discussion boards were effective at all. It seemed like a waste of time and didn't help my understanding of the material.
2. My professors would respond to emails instantly. I got some responses within minutes or at least an hour or so. It was surprising because during the fall, it would take a while for professors to respond to emails, sometimes even days.
3. I had one professor upload lectures she recorded from last year and then she would just email us about additions to those lectures or things we shouldnt pay attention too. It seemed like she took the easy way out and made it harder for us because we had to work to notice certain points or ignore them.
4. Pass/fail options definitely helped because if you were doing badly in a class and didn't want it affecting your gpa, it could be made into a pass. It was also nice knowing other universities for graduate schools and medical schools are going to take spring 2020 with a grain of salt and not diminish your transcript because of pass/fail.
5. My university did a lot to help students, I don't think there is anything they could have done better.
6. No, I discussed everything

Survey ID 28

1) Just linking other resources online instead of explaining material over lectures.

2) Checking in with students, setting up virtual office hours and q/a sessions to help students who are struggling with concepts, and being accessible and responsive via email.

3) Not replying to emails, not updating students about the course syllabus changes in a timely manner, not having virtual office hours or any way to reach out to them except for email.

4) Pass/Fail option really helped ease the stress of getting a good grade and instead focusing on learning the material. Extending the deadlines to drop/withdraw from classes also helped.

5) I wish the university provided us with more information and resources to reach out to about problems with courses or professors. And also held professors accountable for communicating with their students and providing updates on syllabus changes.

6) No I think that covers it!

Survey ID 20

1. The pre-recorded Zoom lectures were ineffective because I would easily get

distracted and go on my phone while mindlessly scroll through Twitter. Also, I

found them boring, and I could not connect it to my classwork.

2. My professors took certain assignments off from the syllabus and were more lenient

towards us about turning in assignments and extending due dates/deadlines. For

example, my English professor took away a paper and replaced it with an A while

my computer science professor gave us an extension for our final project granted

the university gave them more time for grading. Also, all of my professors were

open to their emails and always available to talk to us via Zoom whether it was

school-related or not.

3. None
4. Redacted did alternative grading which was essentially pass/fail in which SAT

represented a passing grade whereas V represented a D and Z represented an F.

They hosted numerous townhalls on topics regarding housing refunds, financial

help, and other resources. They included CAPS (mental health facility at Redacted), multicultural resource center to help students of color through this time. The

LGBTQA+ center was available remotely for students as well as Redacted

learning, which provided tutoring services for subjects such as math, science, and

economics.

5. I wished that Redacted was transparent and clear about everything because they

told us that we were going to remote learning during spring break and people were

still on vacation or working so we did not have time to go back and grab our things.

Personally, I could not get my stuff until July because of the pandemic and my dad’s

work schedule.

6. None

**High SES Asian Women**

Moderator 14:45

Okay, we can go ahead and get started now. Um so welcome to the focus group. My name is Moderator I know it says Moderator on the screen I'm just using an account of another research coordinator. So I'm a research coordinator from Redacted and Note-taker is our note taker today so if you could wave to everyone, Note-taker. Um just a little bit of like background rules, we'll be asking a number of open ended questions today. There are no wrong answers but people may have differing points of view. Please feel free to share your point of view, even if it differs from what others have said. Keep in mind that we're just as interested in negative comments as positive comments. We want everyone to feel comfortable participating and we encourage everyone to talk. Because we have to keep this to an hour I may have to cut you off so I apologize in advance. We want to get the full breadth of experiences and opinions but some of you may have very similar experiences and opinions. So if your answer is the same as another person you may simply say my answer is the same as, say Sam's. It is difficult for us to take notes on everything that is said in the group. So we'll also be recording this session so that we we may transcribe it later. We will not associate your name with anything you say in the focus group. You may refuse to answer any question or withdraw from the group at any time. We understand how important it is that this information is kept private and confidential. We will ask participants to respect each other's confidentiality and not repeat things said within the group to anyone outside of the group. Are there any questions before we get started?

27 16:27

No, I don't have any.

Moderator 16:30

Um so first let's go around and have each person say their name, what year of college you just finished, and please describe the pandemic, what life has been like during the pandemic using one word. We can start.

38 16:45

Yeah, I'll go first. Uh I'm 38. Uh I'm majoring in computer science and I've been during this pandemic. It's been frustrating.

Moderator 16:56

Okay frustrating. What about 36?

36 17:00

Um I'm 36. I am majoring in environmental sciences and chemistry. I just finished my first year, and the pandemic has been stressful.

Moderator 17:12

Okay.

27 17:14

Um my name is 27, and I am a statistics major. I'm currently a junior, and I would say that the pandemic has been very different.

Moderator 17:24

Thank you all for sharing. And now just before we get started with questions I have a little bit of an overview of the project we're working on. So, as you know, the COVID-19 pandemic has presented big challenges for undergraduate students. Universities across the nation have closed moving all instruction online with very little time for students or faculty to prepare. Um many students have encountered challenges related to the closing of campuses, relocation, and the movement to online instruction. In this study, we want to learn how the closing of campus and the pandemic in general has affected you. And we want your opinions about the good and the bad on how things went, as well as what your professors and universities did that was helpful and unhelpful, and what you wish they would have done. So we're going to start by discussing where you went when your campus shut down. I'd like to hear from each of you. So tell me about the space in the home you moved to. What space did you have to do your schoolwork there? Um was the space private or shared and if you shared it, who did you share it with? We can start with 38 again.

38 18:32

Yeah I'm from REDACTED University so I was on campus when the lockdown first happened. Uh when they decided to go online, uh I was in my dorm, but looking at conditions there after two weeks I moved to my uncle's and aunt's house which was around 10 minutes away. So I did the rest of the course uh coursework there.

Moderator 18:54

And when you were at your uncle and aunt's house what kind of space like where did you do your schoolwork?

38 18:59

Uh I lived in the guest room so mostly I did it there.

Moderator 19:04

Okay. And did you have this space to yourself?

38 19:07

Yeah yeah, I did have the space to myself.

Moderator 19:09

Okay, and 36.

36 19:12

Um after school went online, I moved off of my dorm on campus, and I've been living with my sister ever since at her place. So I've just been crashing on the couch. And I'm still doing that until campus goes back online. I mean offline, sorry.

Moderator 19:37

What kind of space did you have to do schoolwork, or where did you do schoolwork, when you were staying with your sister?

36 19:41

There's like a table in the living room. I just work there.

Moderator 19:47

And did you have this space to yourself or did you share it with your sister?

36 19:51

Um I share it with my sister and her two other housemates.

Moderator 19:55

Okay. And what about you, 27? Where did you move to when um the shift to online school?

27 20:02

Yeah, so I was a student at the University of REDACTED. So I was in an apartment with four or three other people, four total. Um so I moved from my apartment back to my house which is um about like 10 hours away from REDACTED so um I have a house of six people, so it's definitely a noisy environment but I did a lot of my studying in my room at my desk, and I have my own room I don't share it with anyone else.

Moderator 20:32

Okay got it. Um so my next question is what challenges did you encounter um attending classes or doing schoolwork in this space? And we can start with 38 again.

38 20:44

Uh I think the biggest challenge, since I'm a computer science major there's a lot of coding. I think it was really hard to get help, because it's not easy to code while sharing screen. It's quite difficult to understand what you like a TA is saying, so I think that was the hard part. I don't think the office hours were uh regulated properly. I think a lot of people didn't know what was going on, and it was quite confusing.

Moderator 21:13

Okay. 36 what kind of challenges did you encounter related to the space you were working in?

36 21:20

Yeah kind of similar to 38, but I think the biggest challenge for my professors was um for in person lectures, they would often write out problems on the blackboards and they weren't able to do that over zoom, so they would try to annotate on the lecture slides, and it was really hard to read. So I guess they weren't familiar with the technology and it was just confusing for the students.

Moderator 21:51

Um so in addition to the like the problems you had with the professor using technology, were there any things like specific to the environment you were in, like noise or?

36 22:05

Not really, actually.

Moderator 22:07

Okay, thank you. And 27.

27 22:10

Um, I would say for me the biggest problem in the environment that I was in was probably noise. I have like a lot of younger siblings so they were also trying to complete their schoolwork and there was like a lot of yelling and like, frustration, so that was tough. And then I also live in a very rural area so sometimes I had problems with my Wi Fi, um which would definitely affect when I would get my work completed. Um but that's about it.

Moderator 22:40

Okay so noise and Wi Fi. Um my next question is what are things that you or other people in the house did that made it easier for you to attend classes and or do schoolwork?

38 22:56

So I lived with my uncle and aunt so most of the time they would go for work, because they still had to, and so the house was mostly to myself so I didn't have much disturbances uh like, physically.

Moderator 23:12

Okay. 36.

36 23:16

Uh when I lived with my sister if we both had zoom meetings at the same time we would just be in separate rooms, and try not to disturb each other.

Moderator 23:26

Okay and 27.

27 23:29

Um, I would say that if I had zoom meetings or any lectures my parents would try to keep my siblings quiet, and they also helped me out by like cooking my food and doing simple things so I felt less stressed out.

Moderator 23:42

Okay. And if you did live with siblings to what extent were there differences in how much parents supported each of you and your ability to attend classes and complete schoolwork? So for example were some kids in the house given more time, space, and consideration to do school by parents than others.

38 24:08

Um my cousins uh came and went to the house uh because they needed stuff. So there was not particularly any disturbance we kind of kept to our own rooms, and to our own spaces so we didn't disturb each other at all.

Moderator 24:26

And did you feel that um, so you were living with your uncle and aunt right? So they

38 24:34

Yeah.

Moderator 24:34

Um you felt that you had the same amount of like time and space to do work compared to your cousins?

38 24:40

Yeah, because we all had our own rooms and my cousins would sometimes use the uh living room, which I didn't really use because they there would always be TV but I think I had my own space in my own room.

Moderator 24:55

Okay and 36.

36 24:58

Could you repeat the question actually?

Moderator 25:00

Sure. So, if you lived with siblings, to what extent were there differences in how much parents supported each of you and your ability to attend classes and complete schoolwork. Uh for example, were some kids in the house given more time or space?

36 25:18

Um well, my sister already graduated, so she wasn't in school, but neither of us really get any support from our parents.

Moderator 25:28

Okay, thank you. And 27.

27 25:32

Um, I would say that yes, some of my siblings were given more time and privacy, but I think that's because of like the age difference, for me. I'm almost twenty years old so I think my parents just expect me to adapt to the environment and just continue to do my own thing, whereas my other siblings um they might have like harder classes or they're really struggling and they need the quiet so yeah they definitely did get more attention and privacy and quietness than I did.

Moderator 26:05

Okay. My next set of questions will be related to what other responsibilities you had after your campus closed and how these differed from responsibilities um before campus closed. So please describe your caregiving responsibilities, which may include child care, elder care, or caring for ill loved ones, and how they were like or different from your responsibilities before the shutdown.

38 26:34

Um, since I was living in the dorm I didn't particularly have any of those responsibilites. Yeah.

Moderator 26:42

And did you um do you have more, or maybe the same amount of responsibilities after the shutdown, or during the shutdown I mean?

38 26:52

I mean not not those responsibilities particularly but of course like moving stuff. And um since I'm an international student of course there was the flight shutdown and all of that. So those were some extra responsibilities after the shutdown.

Moderator 27:09

36, how did any caregiving responsibilities change, once there was a shift to online learning?

36 27:16

Um the they didn't really change that much except on campus I had access to the dining halls so I didn't have to worry about like getting my own food and cooking. But also, there was a time when my sister got really sick and I had to drive her to the emergency room, like twice and that was kind of scary.

Moderator 27:41

Yeah. Okay. 27, did you see um experience a change in caregiving responsibilities?

27 27:49

Um, I would say before when I lived in my apartment I didn't have any caregiving responsibilities, and when I came back home uh I didn't, I still didn't really have any because my parents made sure they were taking care of my younger siblings so I didn't really have to watch over them or anything.

Moderator 28:05

Okay. And I know some of you touched on this but could you tell me about any household responsibilities, such as chores, cooking, or caring for pets during this time, and how they were alike or different before school closed?

38 28:26

Um since, yeah I moved from dorm to my uncle's house so I think in dorm I didn't really have much except just cleaning my room. But I think after moving to my aunt's and uncle's I kind of um helped in household chores, usually just dishwashing and sometimes helping cooking and cutting.

Moderator 28:46

So it sounds like a little bit of an increase in responsibilities, is that right?

38 28:50

Yeah, a little bit, yeah.

Moderator 28:52

Okay. 36.

36 28:55

Yeah, I would say about the same as 38. Um yeah, same answer.

Moderator 29:01

Okay, and 27.

27 29:04

Um I would say for me before my responsibilities would include like cooking, cleaning, doing everything on my own because I was in an apartment and had to, obviously take responsibility but when I came home, my parents were really um nice about cooking food for me, making sure I didn't have to do too many chores. Um so I would say my responsibilities decreased.

Moderator 29:29

Okay. And to what extent were you expected to take on more household work, childcare, or family caregiving responsibilities than other people in your house? So for example, parents may have expected more of one sibling or partners slash roommates may not have contributed equally.

38 29:50

Um I I don't think there was any difference in expectation. I think I just helped whenever I was free. Um yeah, I don't think other than that, there was any expectation or difference.

Moderator 30:04

Okay.

36 30:07

And same answer.

Moderator 30:09

Okay.

27 30:10

I would say for me in my apartment, my roommates definitely didn't contribute equally to cleaning and that kind of those kinds of responsibilities, but in my house um I just kind of helped out whenever I was free just like 38. So, same answer essentially.

Moderator 30:29

Okay. Now we're going to talk about how your academics were impacted by the shutdown. Um I will ask you how the shutdown has impacted a variety of things. If you don't think it has impacted you you can just say no impact. So in what ways, if any, has the shutdown impacted your academic performance (grades) and your understanding of the subject matter of your courses?

38 30:56

Um so, my technical courses were just computer science and calculus 2. I don't think there was any change in grades but I think I struggled a lot in just keeping up and keeping up the grades that I had. Yeah, that's it.

Moderator 31:14

Could you explain what um what made it difficult to like keep up the grades?

38 31:21

I um probably because there was no interaction. I think we were just given pre recorded videos so it was hard to ask questions immediately so we had to mail and wait for the answer. And it is kind of different when it's face to face, and through mail, especially with math. Uh usually, the professor or the TAs would upload those but it was still a bit confusing. Uh so yeah.

Moderator 31:49

36, how were your grades and understanding of the course material impacted?

36 31:54

My grades weren't impacted as much but my understanding of the material definitely was because, um similar to 38, the the asynchronous instruction and pre recorded lectures didn't allow you to interact with instructors as well. So, like there was definitely a lot of concepts that I just never really grasped. So, it it was more difficult to to get a full understanding of the material.

Moderator 32:29

Okay, so the lack of interaction was difficult.

36 32:32

Yeah.

Moderator 32:33

27, what about you?

27 32:35

I would say, so the three most important classes I took were calc 3, and then a calc 3 based stats class, and then a computer science class, and my calc 3 class had been online to begin with. So my grade for that class wasn't really impacted because I had to hold myself responsible for learning on my own. Um for my statistics class when it transferred, or transitioned online I think I actually ended up doing better because I had more time to soak in the material and like rewatch the lectures, um but for my computer science class I think the lack of interaction definitely impacted my understanding, um and affected my grade in the class as well. Because I think being able to talk to someone about your code definitely helps you understand things better.

Moderator 33:25

Okay so again like the lack of interaction seems like it was hard. In what ways if any has a shutdown impacted your career goals?

38 33:40

Um, I I don't think much. I think my career goals are the same. I still plan on graduating in three years and moving to the job force. Um of course there's the worry about the decreasing jobs, um but yeah I I would still like to go to the job force.

Moderator 33:59

Okay.

36 34:02

Um no impact for me.

Moderator 34:05

And 27.

27 34:07

I would say now that I've had like more free time to think about what I want to do with my career, like the specific career path I want to pursue has changed a little bit, but my career goals, I guess are the same. I still want to graduate as soon as possible and get a job.

Moderator 34:23

So sorry, you said your career path changed or?

27 34:26

Yes.

Moderator 34:28

Okay. And just having more time to think about what you want to do affected it?

27 34:32

Yes.

Moderator 34:33

Okay. In what ways if any has shut down impacted the availability of other learning opportunities such as internships?

38 34:47

Um so, this has just been my first year so I was not particularly looking for internships, but hopefully next summer it will be there.

Moderator 34:58

Okay, 36.

36 35:00

Um for me, I actually got an internship. Like I applied at the start of quarantine and I am currently doing it right now. It's like a small group, and um we try to follow COVID guidelines.

Moderator 35:17

Okay and do you think this opportunity would have been available had it not been for the pandemic because I know some internships for example were made remote.

36 35:27

I think the pandemic gave me a bit more time to like, look into opportunities and I might not have applied if not for that. But otherwise, yeah.

Moderator 35:40

Okay. 27.

27 35:43

Um, I think, when I had applied for some internships right when the whole coronavirus situation happened I had like two or three interviews get cancelled because the companies just decided they didn't want to take in any interns. Um so I think that was tough, but I didn't continue my search after that so I don't have any comments on after.

Moderator 36:11

Were there any new learning or career development opportunities that emerged during the shutdown and if so could you explain what they were?

38 36:19

Um no, not to my knowledge.

Moderator 36:22

Okay.

36 36:24

Same answer.

27 36:28

Can you repeat the question?

Moderator 36:30

Sure. Uh were there any new learning or career development opportunities that emerged during the shutdown and if so could you explain?

27 36:38

Um, for me, uh my school offered like two classes that you could take over the summer to work on like data analysis skills, or like social media marketing skills and if you um complete both of the classes you get like an interview for an internship in the fall, so I've been doing that. Um and that was just a new opportunity that I took advantage of.

Moderator 37:02

Okay. So if in what ways if any has the shutdown impacted your confidence in your ability to successfully successfully complete your bachelor's degree at all or in a timely fashion?

38 37:19

Um I think it has impacted um a little since uh REDACTED also uh offered an completely online course for the fall, and it does not particularly give all the courses that I wanted to take. So I'm just wondering how long this pandemic will go on and how limited the courses that will be offered by REDACTED so we are looking into how to take a semester off or like a gap year maybe if uh cases get worse.

Moderator 37:53

And sorry is this because uh REDACTED is not, is it REDACTED right?

38 37:59

Yeah.

Moderator 38:00

Um because they're not offering the courses? Is that why?

38 38:05

Yeah, uh they're offering some courses but not all. The courses uh they're offering are mostly taken by freshmen and sophomore, so I'm alright for the moment but if this thing goes on it will be quite, I don't know.

Moderator 38:20

I see, 36.

36 38:24

Um I am a little worried about completing my bachelor's on time if the pandemic like never ends. Because I don't want to take all of my college courses online. Like I have a lot of lab courses I need to take that don't really work over like online instruction. You kind of have to be in person for those. So I might have to end up like taking a leave of absence or something like that I wouldn't really know.

Moderator 38:58

27 has your uh confidence to complete your bachelor's degree at all or in a timely manner been affected?

27 39:06

Um, so I would say that if, if this whole situation continues and courses are online next semester as well, I think that will be a problem because I don't want to keep taking online courses because I think for STEM majors especially that's kind of difficult because it requires a lot of hands on learning. Um so I would probably take a leave of absence for a semester or something. Um but um yeah, that's that's basically it.

Moderator 39:38

Okay. In what ways, if any, did the campus shutdown impact your finances?

38 39:51

Um no impact.

36 39:58

Also no impact.

27 40:02

I would say for me, I usually get a job every summer but this summer I didn't feel as comfortable working because the virus is spreading very rapidly in my city and I don't, I didn't feel good about having person to person contact. Um but it hasn't really impacted my finances, because it's just more of like a side, side thing.

Moderator 40:25

Okay. Now we're going to talk about what your professors did and did not do during the move to online learning. So what are some examples of strategies, tools, or technologies that your professors used that you found to be effective? So they made it easier to learn during this time of online learning.

38 40:46

Uh for my computer classes uh they included new Piazza forums to engage in discussion and ask doubts, but they also included online um calls and video calls uh from the TAs to ask doubts about codes and coding and the projects and stuff. For math, uh our professor used to mail us regularly with what to do and uh how much we needed to do and complete by the week. So that that was helpful. Yeah.

Moderator 41:20

So it sounds like a lot of communication.

38 41:23

Yeah, it was basically just like using a lot of platforms just to communicate and engage in discussion.

Moderator 41:31

Okay. 36, what are some things that your professors did that made it easier to learn?

36 41:37

Um most of my professors, didn't actually do much to help us learn better online, but um my chemistry class was actually not not too bad because a lot of the TAs we had would hold extra, extra office hours before exams and make sure that everyone who needed extra help had had access to it.

Moderator 42:03

Okay and 27.

27 42:07

Um I would say that my professors increased um how accessible they were to students. Uh they used a lot of different platforms. Um for example, my statistics professor used Twitch to stream his lectures, and then they would be saved on there. Um and he also used Discord. Um so we could communicate with him literally at any time of the day and he'd respond really quickly, and we also used Piazza um and uh zoom as well.

Moderator 42:39

And for his lectures, you said they were recorded so could you go back and watch them?

27 42:43

Yeah, so they were live streamed on Twitch and then I think Twitch saves them for two weeks so you can go and watch them anytime between the two weeks timespan.

Moderator 42:52

Okay. On the flipside were there any things that um your professor did like some examples of strategies or tools that were ineffective, so they didn't help you learn?

38 43:08

So for one of my computer classes, uh our professor changed a midterm and a final exam to a very big project. And it was a huge coding project, and that did not help because it was like 30% of the grade, and it was very complicated. It was not supposed to be there in the first place so it when it was there it was graded by a a junior or a senior TA and it was incredibly hard.

Moderator 43:41

36, were there any things that your professor did that your professors did that you found to be ineffective?

36 43:47

Uh but nothing really comes to mind, actually.

Moderator 43:56

Okay, well, if you think of anything, feel free to join in.

36 44:00

Okay.

Moderator 44:01

27.

27 44:02

Um, for me, the only thing that I can think of is some of my professors tried to change, like the grading for their classes. I know my statistics professor he had a midterm replacement policy and he tried to cancel that, but all of the students got upset about it and he changed it back so yeah but nothing else comes to mind.

Moderator 44:24

And the changing of this policy was as a result of the pandemic or online learning?

27 44:29

Yes, it was after the pandemic.

Moderator 44:31

Okay.

27 44:32

He thought it would make things better, but everyone got upset because he had changed it from uh your final replacing the lowest midterm grade to no midterm replacement.

Moderator 44:45

Okay.

36 44:47

Actually, um for my, my calculus class my professor, because of the pandemic, changed the weighting of the final from I think 40% to 50%. And that wasn't helpful because he wanted to ensure that people were like being honest because there was a proctored final. But that was not helpful.

Moderator 45:16

Okay. What were some of the things that your professors did during online learning that made you feel like they cared about their students?

38 45:30

Um, not much really.

Moderator 45:34

Okay.

36 45:38

Yeah. Also, not much.

27 45:42

I would say for me um my statistics professor specifically like just the amount that he made himself like accessible. Um if I had a question at like 7pm in the night he would literally answer it within five minutes. Like I could just contact him at any time, and also all of my professors were really supportive. They would send out a lot of like, um understanding, like motivational emails to us every week, um saying that they really understood like our what position we were put in.

Moderator 46:17

Okay so a lot of communication again. Were there things that your professors did during online learning that made you feel like they didn't care about their students?

38 46:30

Um, definitely the introduction of the final project that I just said, and also a lot of policy changes. Um and uh like there were like syllabus was changed too so even that did not help. Yeah.

Moderator 46:50

What kind of policy changes were there besides the project?

38 46:54

Um mostly the uh grades, the percentage, the weightage that it had on our final grade. Uh because the project was so hard, they had to change the weightage and they also canceled the second part of the project because it was very hard but they also kept it as a extra credit. So it was just like a lot of confusion and, yeah.

Moderator 47:19

Okay. 36 were there things that your professors did that made you feel like they did not care about their students?

36 47:25

Yeah. Also, just like I mentioned earlier, make, even though I understand that they're making it harder to cheat in their classes, it also made it harder to succeed, even without cheating. But, yeah, the weighting changes really made it more difficult, because it put a lot of stress on students during exams.

Moderator 47:52

Right. And 27.

27 47:56

Um, I would say for me, pretty much nothing. I felt like all of my professors really cared about me.

Moderator 48:03

Okay. Um my last three questions will have to do with how your university responded to the move to online learning. So what are some things that your university did uh to help students be successful during online learning? You can mention any resources the university provided or actions they took to help students be successful or feel supported. So for example, pass fail options, town halls, or remote counseling, things like that.

38 48:39

Um, I think REDACTED did a lot. I think they had provisions for internet, they kept the library open if you didn't have internet in your place. They also extended the pass fail option. You could do it uh till before the final exams, start. Um yeah, and they also had online counseling from the mental health center. Yeah.

Moderator 49:03

Okay.

36 49:05

So my university has this policy where you can't take more than 25% of your classes, as pass/fail, but they lifted that during the pandemic and so all the classes you take pass fail didn't count toward that percentage. And also, remote counseling was made available. Like before, if you went to counseling on campus I think there was a limit to how many you could go to, but they lifted the limit.

Moderator 49:37

Okay. And 27 what are some things your university did?

27 49:41

Um I would say my university did credit no credit. They had extended that option to students and it also applied to classes within your major. Um and then that option was extended till right before finals. Um initially it was in April, when the deadline was for when you could choose that but they extended it to May. Um, I think they also had like some technology assistance program where if you needed um like a laptop or a webcam they could send that to you and loan it out to you. They also had some type of like funding, where if students had financial need they could fill out a form and get up to like $1,000. Um and I'm I'm not sure about counseling, but I think they did, they did an okay job with helping students.

Moderator 50:30

Is it the credit/no credit, similar to a pass/fail?

27 50:34

Um, yes. So with the pass fail option they had decided that uh a professor would, if, if a professor wanted to do pass fail for their class, it would apply to the entire class, whereas credit no credit was elected by students, and it doesn't apply to the entire class but I think they are similar.

Moderator 50:55

Okay.

27 50:56

I'm not sure about like the grade cut offs for both.

Moderator 51:01

Got it. Is there anything you wish your university did better uh to help students be successful during online learning?

38 51:14

Um not particularly the learning part, but I guess as an international student there was a lot of confusion about visa. So I wish REDACTED was more transparent with what they were doing regarding it. Um yeah.

Moderator 51:33

Okay so more transparency. 36, was there anything you wish your university did better during this time?

36 51:41

Also more transparency. Like, um they're still um they've been withholding a lot of information on like our housing applications for next year and whether housing will even be available and whether we'll have housing guarantees honored when we go back to campus. So, I think a lot of the information that we would like to hear, or would like to know, they just haven't been giving us any, any confirmation.

Moderator 52:19

I see, and 27, is there anything you wish your university did better?

27 52:23

I would say regarding learning, having the credit no credit option was really helpful, but regarding their response to like the entire situation I think that, um, initially, the university had kind of said okay you're coming back to school. And then all of a sudden during our spring break they just like canceled school and then everyone was expected to like go back and move out. And they forced people to move out and um they shut down some of the dorms and also like regarding classes for the fall. They like shut, they shut off our registration so I don't really know if my classes are going to be online or in person. Um and if they are online, I think that's gonna be a little bit of a problem, but I guess I'll just have to adjust.

Moderator 53:12

Okay. And my last question. Um in general, are there any ways that your ability to be successful in school was affected by by the pandemic um that we have not discussed?

38 53:24

Uh I think we have discussed most of it.

Moderator 53:33

36.

36 53:34

I definitely felt like a huge drop in motivation and focus throughout the pandemic because working at doing classwork at home was really different from like having spaces to do work on campus and that really impacted my performance in my classes.

Moderator 53:56

Okay and 27.

27 53:58

I would say same answer as 36.

Moderator 54:02

Okay um that's all the questions I have for you today. Thank you so much for sharing and joining us. If you could just send us an email with the name you used during the focus group and then once we confirm that you finished the survey, we'll send you the gift card. Thank you again. Thank you.

**Low SES Asian Men**

Moderator 15:40

All right, it looks like everyone's here. Um Moderator I believe there's only supposed to have four participants today. But if there's anyone else coming you can let them in. So we can go ahead and get started. Welcome to the focus group. My name is Moderator and I'm a research coordinator from Redacted. It says Moderator, but I'm just using another account. So Note-taker will be our note taker today, Note-taker if you could wave or just say hi.

Hi, everyone.

So before we start, I'm just going to go over some ground rules. Today we're going to be asking a number of open ended questions. There are no wrong answers, but people may have different points of view. Please feel free to share your point of view, even if it differs from what others have said. Keep in mind that we're just as interested in negative comments as positive comments. And we want everyone to feel comfortable participating and encourage everyone to talk. Because we have to keep this to an hour, I may need to cut you off, so I apologize in advance. We want the full breadth of experiences and opinions but some of you may have very similar experiences and opinions. If your answer's the same as another person, you may simply say my answer is the same as say example Sam's or whoever you're agreeing with. It is difficult for us to take notes on everything that is said in the group. So we will also be recording the session so that we may transcribe it later. We will not associate your name with anything you say in the focus group. You may refuse to answer any question or withdraw from the group at any time. And we understand how important it is that this information is kept private and confidential. We'll ask participants to respect each other's confidentiality and not repeat things said within the group to anyone outside of the group. Are there any questions before we get started? Okay, so first let's go around and have each person say their name what your college you just finished. And please describe in one word, what this what life during this pandemic has been like. So we can start with 31.

31 17:55

Hello. I just finished my freshman year and If I had to describe My life during the pandemic, I would say slower.

Moderator 18:09

25, what about you?

25 18:11

Hi, so, um, I just finished my sophomore year and I'll describe it as um uh interesting.

Moderator 18:21

And then please correct me if I don't pronounce it correctly. 17.

17 18:26

Yes, I finished my freshman year of college and I would describe it as irritating.

Moderator 18:34

And then 39 how abou- what about you

39 18:37

so I finished my first semester in college, and this whole time has been very lazy.

Moderator 18:44

Right. Thanks for sharing. One second. Sorry about that. Okay, and then just a little bit of an overview of the project. So the COVID-19 pandemic has presented big challenges for undergraduate students As you know, universities across the nation have closed moving all instruction online with very little time for students or faculty to prepare. Many students have encountered challenges related to the closing of campuses, relocation and the movement to online instruction. So in this study, we want to learn how the closing of campus and the pandemic in general has affected you. We want your opinions about the good and the bad on how things went. And we also want to know what your professors and universities did that was helpful, but also unhelpful and what you wish they would have done. So we're going to start by discussing where you went when your campus shut down. And I'd like to hear from each of you. So if you could tell me about the space in the home that you move to what space did you have to do your schoolwork there? So was the space private or shared and if it was shared, who did you share it with? 31, let's start with you again.

31 19:53

Alright so well when I went back to my house, I just took over uh my room and it's a private space. So yeah.

Moderator 20:03

Okay, and then you are doing your work in the room?

31 20:06

Oh, yeah, for sure.

Moderator 20:07

Yeah. Okay. And then 25, what about you? What? Where did you go? When there's a switch to online learning?

25 20:17

Yeah. So same with 31 and I just went home and now did my did my work and schoolwork at uh- in my room at home.

Moderator 20:23

Ok and was your room priavte? Or did you share it with anyone?

25 20:26

Um private

Moderator 20:29

Ok 17 What about you?

17 20:31

At first, my university, like gave us the option to stay on campus and then I did that for a week. But then it just wasn't working for me with no one there no resources or anything. So I decided to go back home and just utilize one of the office rooms my parents had.

Moderator 20:47

Okay, so you spent the majority of time at home and so was that office shared or was that. Did you have that to yourself?

17 20:54

Oh, it was to myself.

Moderator 20:55

Okay. And then 39, could you comment on where you went?

39 21:01

I studied from my home, I live with my parents. So it stayed. It stayed same after the pandemic, and it was a private room.

Moderator 21:15

So my next question is what kind of challenges did you encounter when you were attending classes or doing schoolwork in this space? 31?

31 21:27

I sorry, are you saying like challenges like only pertaining to like doing schoolwork?

Moderator 21:32

Yeah, like challenges related to school work in that particular space.

31 21:36

Oh, I mean, I guess for me it was just kind of like uh I had more trouble focusing because like, it was difficult to separate like, Um like play from work because everything was for me it was like in the same space. So like, I definitely did have to spend a lot more time studying because uh it's just like I just kept getting distracted by my phone. By my Computer and stuff like that when, like when I'm on campus, I just like, you know, I just need to cram and I just like go to the library or something. I can just like, pump in hours and get out. You know what I mean? So,yeah.

Moderator 22:12

Okay. And then what about you, 25? What kind of challenges did you encounter in this space?

25 22:17

Yeah, so similar to 31, um, it's hard to differentiate between, you know um, relaxing and leisure versus actually doing work because everything was in the same space. So yeah, I kind of had that same struggle.

Moderator 22:30

And then 17?

17 22:33

I relate to both of those answers because I am very bad at procrastinating and just being at home. It enhanced that because like, I'm right next to my TV. My mom and dad are home too. And like I would just get distracted by the littlest of things. My parents would be doing something or like my dogs would be barking and it was just hard to concentrate on school in general.

Moderator 22:53

Okay, so more distractions. And then 39 what kind of challenges did you encounter in the space

39 23:00

So, uh one thing is that since schools were closed, I have a younger brother, so I had to help him in his homework. And another thing is, yeah, distractions, like not catching up with the deadlines and everything.

Moderator 23:17

I see, okay. So my next question is, what are some things that you or other people in the house that made it easier? Sorry, let me start over. What are some things that you or other people in the House did that made it easier for you to attend classes? Or do your schoolwork? Let's start with you with 31 again.

31 23:41

Uh so what I did, I mean, it's kind of weird, but my professors recorded lectures. So I just stopped going to live lectures and I kind of like switched my sleeping schedule. So my parents would be like up in the day and stuff and I switched my sleeping schedule, so I'd be up at night. So I'd be like, kind of be like nocturnal. You know what I mean? So I'll be asleep during the day like during, like when everyone else is awake and then at night, I'd be doing all my stuff because it'd be quiet it'd be dark, you know, you know what I mean, I can do everything and not get distracted- well as much at least.

Moderator 24:13

So did you switch your sleep schedule so it'd be more condusive to learning?

31 24:16

Yeah, basically. Because like if it was during the day there's like, there's like so much noise like fucking like ice cream trucks are like going around and like, you know, like people are like screaming and stuff but at night it's like very relaxing for the most part.

Moderator 24:29

It's a quieter environment. 25, what about you? What did you or your family do that? That made it like, easier to learn or do your work?

25 24:39

Um, so basically the if I was doing work, I would, you know, lock my door and then they would kinda understand that i'm doing work or studying or watching a lecture and yeah, I would also I would have a um um door tag, like doing work on tape on from my door, and then people would know not to bother me and stuff.

Moderator 25:01

Okay, so kind of signaling that you needed some time to do at work or quiet. Um 17. So what about you? And what did your family do to help you? To help you do your work or study?

17 25:16

Being at home my parents basically just respected my space. And whenever I had like a test or something, they would just like try to be quiet. And like just the whole idea of like being quarantined at home. Like I have no other social obligations to go to or like, I don't have to worry about food anymore because like, my parents would make that for me. And then I would just have less stuff to worry about and just could focus on school.

Moderator 25:38

Okay, And 39.

39 25:42

Yeah, so I um made a schedule with my sister. So she was like that, Three days of the week she'll help my brother and those three days. I'm never going out of my room it's locked. So I was never leaving my bed. I brought a table. You can put it on your bed. You can do your stuff there. And yeah, basically everything from your bed, doing sleeping and then doing the homeworks and stuff. Yeah.

Moderator 26:08

So it sounds like you were taking turns with your sister helping your siblings?

39 26:13

Yes. Yeah.

Moderator 26:21

So if you live with siblings um to what extent were there differences in how much your parents supported each of you and your ability to attend classes and complete schoolwork. For example, were some kids in the house given more time, space and consideration to do schoolwork by parents than others?

We'll start with you, 31 again.

31 26:43

Um I have a brother but he doesn't live with us anymore. So I was alone.

Moderator 26:47

Okay. And then 25?

25 26:55

Um, yeah, sorry. I yeah, we had the same um, we have the same privacy. And same attention given to both really.

Moderator 27:03

Okay. 17?

17 27:07

I have a sister but she's not a student she works so it didn't really conflict with anything.

Moderator 27:14

And 39.

39 27:16

So my younger brother, he is a kid. So he was getting a little more time with the parents and I I- don't go. Like, I like to be separated from everyone. So that was that.

Moderator 27:33

And your younger sibling, were they school age?

39 27:39

Yeah, school aged. Yeah, second grader.

Moderator 27:42

Okay. So now my next set of questions, we're going to talk about what other responsibilities you had after your campus closed, and how these differ from responsibilities you had before campus close. So please describe your caregiving responsibilities, including Child Care, elder care or caring for ill loved ones and how they were alike or different from your responsibilities before shut down. We can start with 31 again.

31 28:13

Uh I mean, for me on campus, I was just kind of like minding my own business just like care for my own stuff, washing my own clothes, you know, like making sure I eat something but at home it's just kind of like it kind of just like transitioned to like just like the same as back in high school. Just like my parents make the food but like, I'll just do like more chores and stuff. So I would say like, even though the responsibilities are different, I would say it's basically the same amount of work. So yeah.

Moderator 28:44

25, what about you?

25 28:46

Yeah, so um, I really didn't have to care for anyone except for myself really. So basically just the same thing as if I were to go on campus just different, you know, different stuff I have to do. Whereas like, at campus I would you know, I like- find some meal, find something to eat. It's the same thing but just have to do it at home. So it's basically the same thing, but just kind of adapt to the home scenario or home situation.

Moderator 29:10

So no change in caregiving responsibilities. What about you 17? Did you encounter any change in caregiving responsibilities?

17 29:20

I wouldn't say I got more responsibilities. I felt like it stayed the same. And like I agreed with 31's answer, where everything reverted back to like the high school state where I had to do schoolwork, and do like chores and like help around the house and just stuff like that.

Moderator 29:36

Got it. And then 39, you mentioned earlier about helping a sibling with homework or schoolwork. If you could talk about how your caregiving responsibilities changed?

39 29:50

Oh no other than that, no, my parents were staying home so I felt like a lot of stuff that I need to do take care of myself like laundries and stuff. They were doing everything for me. So, yeah.

Moderator 30:06

I know we touched on this a little bit earlier but if you could describe other household responsibilities such as chores, cooking caring for pets during this time and how they are, alike, or different from before school closed. and 31?

31 30:24

I mean, I think they're more or less the same. I feel like I especially for I know like we have like, like three freshmen here. Um I feel like for most of us, it's like this quarantine hasn't been as bad as for upperclassmen, because we were just in high school year ago, like we were literally like living this a year ago. So it's not that much of a change. So I can't really say anything that's changed too much, but other than like, we're just going back to how we used to live so it's not that dramatic in my opinion.

Moderator 30:54

Okay. And then 25, How have your, say the amount of chores you had to do or other responsibilities changed?

25 31:05

Um, yeah, so basically, I didn't have any change in responsibilities just kinda kept being a student doing that.

Moderator 31:14

Okay. And 17, could you comment on how your household responsibilities changed during this time?

17 31:26

being in college I primarily focused on like just doing my homework. The only responsibilities I would say I had were like keeping my dorm clean and doing laundry. Like at home, I do those things too on top of things like washing the dishes, cleaning around the house, cutting grass and helping my parents with whatever they need, basically.

Moderator 31:45

So it sounds like correct me if I'm wrong, but responsibilities like household responsibilities slightly increased or

17 31:53

Yeah they increased

Moderator 31:55

Okay. 39, what about you?

39 32:00

So I felt like um, so first I completely agree with 31. It's, it was, we went back to our life of high school days. And I felt like I it was slightly decrease of responsibilities for me, from my end was basically I had to do my homework and help my siblings. That's it.

Moderator 32:22

And then when you say um slightly decreased, so what kinds of things

39 32:28

bascially before that because my dad wasn't home. So he was working, I had to do the laundry, the grocery. And after the pandemic started. Everyone was home so they were doing every step so I never had to step foot outside of the home. Yeah.

Moderator 32:47

Okay. And my next question is also related about household responsibilities. So what To what extent were you expected to take on more household work, child care or family caregiving responsibilities compared to other people in your home? So for example, parents may expect more of one sibling or partners, or roommates may not contribute equally. 31, if you could comment on this.

31 33:24

Oh shoot my fault, I don't really think I can comment on this one because I don't think it applies to me because I'm kind of a only child right now. So yeah.

Moderator 33:34

25?

25 33:35

yeah, same with me I didn't have any new responsibilities.

Moderator 33:40

Okay, and sorry, I don't recall if you had siblings.

25 33:44

Yeah I have siblings but um, they're they're slightly bit older.

Moderator 33:48

Oh that's right, okay thanks. And 17, what about you?

17 33:53

I have a sister but nothing really changed in the household.

Moderator 33:57

Um but did did you feel that to you? had the same or different amount of responsibilities as your sister.

17 34:04

I probably had more because she had to focus on working and everything, but it wasn't much difference.

Moderator 34:10

Okay, then, how about you, 39?

39 34:14

So I mentioned that I have a younger sibling, second grader. Besides that, I have a sister. She is a 12th grader. So she was doing the cooking and stuff. So I felt like she had a little bit more responsibility than me. She was helping mom and household works. So yeah, I felt like she was getting more responsibility on the harsh side.

Moderator 34:42

Okay. So now we're going to shift to academics. We're going to talk about how your academics were impacted by the shutdown. And I'm gonna ask you how it impacted a variety of things. If you don't think it impacted something you could just say no impact. My first question from this group of questions is, in what ways if any, has the shutdown impacted? impacted your academic performance? So your grades and your understanding of the subject matter in your courses?

31 35:16

Am I first.

Moderator 35:18

Sure, yeah, we can keep the same order.

31 35:19

Okay. So I got a lot to say about this one. So, I feel like for me, my understanding of the material increased because of Corona. So I feel like it increased because in lecture, um like, for me, at least like lecture would be kind of slow. So being it, the fact that it was like recorded, I could watch it like at four times speed. It just let me blow through the lecture. And then because I was able to go through the lecture so quickly, I'll just understand it a lot quicker and do a lot more. I could do a lot more work. But um performance wise, I feel like my performance dipped because of so basically Because all the tests were online, there was like the huge like cheating scandal, right? I feel like with the cheating scandal, it's like, I have like 100 people in my class, the classes graded on the curve. It's like a super competitive class, as far as I'm concerned, like 80% of the kids or like even more are going to cheat. So not only do I have this pressure to cheat, because the test because the class is graded on a curve, I have to either I feel like, Ieither cheat, or I had to study the material to the point where I can assure myself that I can get 100 and it doesn't matter that everyone else cheated. You know what I mean? So just from the pressure of kind of like this added pressure of like this cheating, affecting the curve, and um just like all this other stuff, and professors making the tests harder because it's online. I feel like my performance dipped, but my understanding increased a lot. So yeah.

Moderator 36:55

Thank you. What about you, 25, How were your grades and your Understanding impacted during the pandemic?

25 37:03

Yes, I would say um, my understanding would be typically the same. I mean, I kept, I just I studied just the normal amount, I studied the same. It just had to adapt to how the professor's cause some of the professors were really like kind of old. So they weren't good with technology. So I had to there was that curve. So they had a learning curve result to use technology. So students, by default had to adapt to how the professor taught, even if he or she was not familiar with technology. So we had to kind of kind of adapt to how he was teaching the course. So in that case, we had one professor who was kind of who's rather old so he was not really familiar with technology and there was a lot of technical technical difficulties that we had to as students had to combat with

Moderator 37:56

So did, from what I'm hearing your understanding stayed the same, but these technical difficulties difficulties affect your performance. So grade wise,

25 38:07

Um I would say so for one class in particular, because that one class where the Professor was not really uh aquanted with technology that that one class kind of hindered my understanding because of lectures, he would literally um, he would write, he would take a piece of loose leaf, he would he would write it he would write the notes and then he would kind of hovered over the webcam and then there'd be blurry and no one could no one could, um, he couldn't he didn't even record the lectures afterwards. So you couldn't you couldn't even look at the lectures afterwards, you would have to go take notes really rather quickly. And then you would with each page he would, you know, wave it to the webcam, and it just, it was a terribly inefficient way to learn on an advanced calculus topic.

Moderator 38:52

Sounds like understanding and kind of grades were dependent on the professor.

25 38:56

Yeah. Exactly. It was dependent on how the professor adapted to the online learning structure.

Moderator 39:04

Okay. And then 17, how were your grades and your understanding of the course material affected?

17 39:10

Oh, this question really depends on like, the kind of class I'm talking about. So if it's like, if we're talking about my harder level classes, I would say my understanding and my grades in those classes uh went down because the material was hard enough, like doing it at home, I had no motivation and like, I have trouble learning online. And I didn't have any other resources like tutors or like friends to help me. So in hard classes, they went down, but like my easier classes, such as history or something, those became pre recorded lectures. I could just skim through material and then just take the test or whatever. So I would say it was easier in that sense.

Moderator 39:48

Okay. 39, what about you?

39 39:51

Yeah, so I'd like to divide it into two parts because first I was doing the spring session, and then I'm doing summer as well. Well, so for the spring part since we had to shut down in the middle of our classes, right? So I had two classes, I was like, I was very confident and interested about them, two other classes liberal classes uh I was, I wouldn't never want to take this classes, but I had to right. So when this pandemic started, and they were taking classes, um exams online, so I was like, I just have to pass the test. This course done, I'm done with the course. So I was using resources online, my grades, super high. The understanding of the subject matter, I didn't understand anything I even don't remember I'm in- I don't I don't even remember most of the stuff. Though subject two subject uh two classess I was very interested. I had to learn them very well because they were doing the kind of open book exams. So no matter what resource you use, You have to be like you have to get the in depth understanding of the matter. So and also I love those classes so I felt like I got better grades, and my understanding got better as well. For the summer class, it's, I felt better. I'm understanding a lot and my grades are going higher. So yeah, I'm very comfortable with the summer.

Moderator 41:26

So what I'm hearing in the in the spring, your understanding it would decrease in classes you weren't interested in and increased? Or maybe you had a you're confident in your understanding in classes you're interested in. Is that right?

39 41:43

Yeah.

Moderator 41:44

And then what do you think changed between the spring and then the summer where you maybe it sounds like the summer you've felt comfortable in all your classes.

39 41:53

Because in the summer, two classes. Both of the classes were constructed in a way it's like open book 30 to 40% of your classes were based on totally innovative things. So no matter what you use, there are a lot of options right, Chegg, stuff, you cannot find any solution. So you have to understand, and then you have to come up with your own solution. So it forced us to get a better understanding of the subject.

Moderator 42:24

I see. Okay. My next question is, in what way, if any, has the shutdown impacted your career goals? 31 we'll start with you again.

31 42:39

Umm I'm thinking, I feel like the shutdown. So as far as me so um so how do I say this? So, right now I'm like in between premed and going into a Chem PhD, But so I'm in a lab right now. And obviously, because I got sent home, I can't work in the lab. So I guess getting sent home kind of pushed me more towards premed, instead of going for like a PhD in chemistry because I can't even work in the lab. So I've just been like, staying away from that and only focusing on like, stuff that I can do. So, yeah.

Moderator 43:22

What about you, 25? Have your career goals changed?

25 43:25

Um, no, no changes in career goals.

Moderator 43:28

Okay. And 17.

17 43:31

They haven't really changed much. But like, I take note of how like certain companies reacted in response to the pandemic and how they treat their employees. And I just, I just note that down for future reference.

Moderator 43:44

And could you explain why you're noting it down?

17 43:48

Because like, okay, depending on a certain company and how they treat their employees. It's just dictated to me how good of a company that is like whether or not they allowed their employees to work from home or whether or not they mandated them to come to work regardless of the pandemic going on and stuff like that.

Moderator 44:04

So that'll kind of affect your, from what I'm hearing affect your decision and where you want to apply or something like that.

17 44:10

Yes. It will.

Moderator 44:11

Okay. And 39, have your career goals been affected?

39 44:17

So about the profession no, but but the companies yeah I agree with 31, because now I'm thinking more of those companies that provide you with more freedom. You have more options, more choices. Mostly you can work from home, they provide you with that much freedom. Yeah, I'm looking. I'm gonna look forward to trying to join them.

Moderator 44:42

So maybe, same field, but right-

39 44:46

Company was different

Moderator 44:48

Got it.Thank you. Um So, following up with that in what ways if any has the shutdown impacted the availability of Other learning opportunities such as internships?

31 45:04

Um, well at least for me because um like I said before I am premed, I've been trying to volunteer at different hospitals and like different clinics, but because of COVID um I think it's just like strictly health related, but because of COVID they've been restricting the amount of like people who can get in the building, so I haven't been able to get anything done with that, unfortunately.

Moderator 45:29

How about you, 25 have any? Uh how has the shutdown impacted the availability of learning opportunities for you, like internships?

25 45:39

Um, not really, I haven't had any impact on any internships.

Moderator 45:48

And 17, what about you?

17 45:50

uh it hasn't really impacted me either I wasn't planning on interning this summer

Moderator 45:54

Okay, 39 Have you felt any impacts on..

39 45:58

Uh yeah, because Usually I wasn't I wasn't planning for an internship because I was working part time. But then I quit uh my workplace uh was shut down and I was I'm taking my summer classes. So before, so I feel like it positively impacted me because before the virus thing, uh Uber had internship way up. So you had to move out of state to do the internship, now they're doing it online. And I got like, for eight weeks with them, I can do it right from my home. So I felt like it helped me because I have I don't have to move out of my home. I can do my classes and also the internship. Yeah.

Moderator 46:45

Okay, so you actually increase the oppor- this?

39 46:47

Yeah

Moderator 46:48

this situation creates opportunity. Okay. And then were there any other learning opportunities or career development opportunities that emerged during the shutdown? And if so, if you could tell me what they were, 31?

31 47:06

Uh I mean, at least for me, um because we got put online I actually started doing umm what is it called Crisis Text Line counseling. So I don't think I would have done that without the without the lockdown. I think I would have done something like in person, but because we got forced inside, I was just like, whatever I'll apply and I got in So yeah.

Moderator 47:31

25, what about you?

25 47:35

Sorry, can you repeat the question.

Moderator 47:37

Sure. So were there any new learning or career development opportunities that emerged during the shutdown? If so, like, could you explain what they were?

25 47:49

Oh, um, no, no, no, no learning or career opportunities.

Moderator 47:56

17?

17 47:57

Yeah, I'm in the same boat. There's nothing much new

Moderator 48:01

Okay, and 39, I know you mentioned the internship, but were there any other opportunity um

39 48:08

so our college, they were they were doing a partnership with Coursera and they provided till August free some courses like free but I didn't take it. So yeah.

Moderator 48:21

Okay, got it. My next question is in what ways if any, has the shutdown impacted your confidence in your ability to success successfully complete your bachelor's degree at all or in a timely fashion?

31 48:39

Uh well, at least for me, it hasn't like really like altered my confidence. It just kind of made me a lot more scared for my GPA. So completing it stayed the same, but in terms of like GPA and how I doing classes, uh it definitely decreased my confidence mainly just because of the all the cheating and stuff in most of my classes that there's a curve. So, yeah.

Moderator 49:02

Okay, got it. How about you, 25?

25 49:05

Yeah, I would say, um, it, it kind of maybe increased my confidence because, um, because the COVID uh the covid pandemic actually made summer classes more, uh more available, at least in my school. So a lot of students took advantage of that. So they're graduating on earlier or even if they weren't graduating on time there, they are now graduating on time, because the more wide availability of summer classes, then students who want to graduate quicker could graduate quicker because the more range of summer classes that were offered because of the pandemic.

Moderator 49:41

so when you say classes were more available, could you explain what you mean by that? Is it just there's more classes or?

25 49:48

Yeah, so exactly. There would be the school implemented more more online classes that pertained to graduation requirements. So say, like, if there was before previous summer there was like, maybe 20 classes that apply to Gen Ed's or General requirements. There may be double that this year so more so they encouraged this university wants to encourage more students taking online classes to make up for the deficit in budget where as when students kind of where they have to refund students for um dorm or or stuff like that so they wanna kind of recoup that by offering more summer classes.

Moderator 50:22

Right and what about you 17?

17 50:26

I feel like my confidence has gone down because especially after seeing how hard online classes for me how hard online classes were for me the first time around, and like how now my my university has announced that half of our classes in the fall are going to be online too. It's just like I'm scared that classes being online again will hurt my GPA.

Moderator 50:47

Okay, I see. 39 has your confidence in your ability to complete your degree or in a timely, timely manner has that been affected?

39 50:59

So about that part the duration no, I think, no, it hasn't changed but I'm feeling less confident because I, for my major, you have to do a lot of practical stuffs and projects together uh on- online we don't have that much of chance. It's just exam so I don't know if it continues like that, I don't know if we're going to be if we're going to be as efficient as as if it was on campus education.

Moderator 51:30

Okay. My last question in this question set is in what ways if any, did the campus shut down impact your finances?

31 51:46

Oh, sorry. But um, I feel like uh it made the finances better because dorming and like the meal plans and stuff are expensive as hell. So at least because my fall semester is online, so I don't have to pay the dorming fee, which is like half the cost for me going to school anyways and um also, my university refunded like a portion of the housing costs and meal plan costs for the spring semester, so we just got money back. And living at home is a lot cheaper than living at university and stuff. And I also feel like at university like when you go out and stuff you'd spend a lot more money but since I'm at home like what am i going to spend money on you know what I mean? So just that vein, I guess it did help financially.

Moderator 52:32

So finances got easier. 25 how how were you affected?

25 52:39

Yeah, I agree with 31 on this one. I saved a lot more money by staying home. I mean, being forced not to be able to buy- get food from restaurants, gas uh my university actually started gave a gave some money tuition back. And there's just been a lot more monetary reasons like like, for example, this survey, right? it's 50 dollors Amazon gift card, come on like, we get we have more we have more stuff to you know we have more ways to get money so.

Moderator 53:07

Okay, so again positively impacted and 17. How are your finances impacting?

17 53:13

My answer is basically the same as 31 like being at home my parents pay for food, everything basically and then with the refund and everything that has really helped too then for my university they have like online study labs basically like this and like they just pay us to participate in stuff online.

Moderator 53:30

Okay, so again a positive impact. 39, how are you your finances impacted?

39 53:36

So it's mostly the same except I don't have I didn't have to like it saves my transportation cost. But yeah, other than that it stayed the same. The fees were same everything same. One thing I'd like to mention here, because I feel for the shutdown and everything most of the students got some kind of grant right? I think so. It Help. I think it helped a lot of us like that extra money.

Moderator 54:04

Okay so students are getting extra. And so you say you were saying transportation costs? So were you commuting to school originally? I'm sorry, I missed in the beginning.

39 54:14

Yeah, because I was attending school from my home so.

Moderator 54:18

oh I see. Okay. Thank you. My next set of questions will have to do with what professors did and did not do during the move to online learning. So what are some examples of strategies, tools, or technologies that your professor used, that you found to be effective so they made learning easier during all that learning?

31 54:43

Oh, at least for me, recording lectures, like I said, the fact that they were recording lectures that didn't have to go to live lectures, I can watch it like four times speed, made life so much easier for me, and also a lot well, another thing my professors did was they were a lot more active in class in my opinion, like they held more office hours, they posted more review sheets, they just try to do everything they could to help us out. And so I guess I'm pretty lucky for that.

Moderator 55:11

Do you think you would get all these resources? So like the review sheets if it weren't for the ....

31 55:15

Hell no, never I don't think they'd ever do like put in this much work because like I feel like so when I saw at least in my calc class, we took the first midterm in person, we take the second midterm during quarantine. And the amount of extra time the professor and the TA put in compared to the first and second midterm It was literally like night and day. Like, I think the I think there's like one set of office hours for the first midterm and there had to be at least like five or six for the second midterm. So it was insane how much they tried to help us out

Moderator 55:57

Wow so the the effort they put in sounds like it increased a lot

31 56:02

Yeah.

Moderator 56:05

25, what are some things that your professors did that helped with your learning?

25 56:09

Yeah, so um, they made some of my professors made like recorded lectures, they could watch back on, on 2x speed and then you could just always refer back to them when you're doing homework or you're unfamiliar or unclear about a certain topic. It goes refer back to the videos, whereas in real life, you couldn't really do that. So yeah.

Moderator 56:29

Got it. 17?

17 56:33

Not only did my professors like prerecord lectures, but like they uploaded everything, review sessions, PowerPoints, the things they utilized in class, like everything was uploaded, and I could always like go back and look at it. In a way I feel like some of the tests they made were, were easier. And yeah.

Moderator 56:51

Okay, so being able to go back to look at those resources was helpful.

17 56:54

Mm hmm.

Moderator 56:56

39, what about you?

39 56:58

So yeah, The common thing here is the recorded classes. So everything recorded, posted questions, solved answer posted. Also some of my professors, they were... So they were... uh it's different platforms. It's like, online homeworks. But like kinda open book, you learn as you complete them. It forces you to focus on your studies. And also some of our professors were creating forums where everyone can post their questions. And then he was like instantly replying. He was there all day. It's like, there was no fixed office hours. Some of the professor every day they were teaching, they were all day, active at the forum. So whenever someone's asking questions or answering, also students could reply back. It's kind of social media for students. So yeah, it helped a lot.

Moderator 57:54

And so that wasn't available before the switch to online learning, like

39 58:01

Yeah.

Moderator 58:06

So on the flip side of my last question, what are some examples of strategies, tools or technologies that your professors use that you found to be ineffective, so they didn't help you learn during online learning? 31?

31 58:21

Um, one thing that they did was or at least they tried to do was um, discussion boards. But I'd had it up for like two weeks and not a single person used them. So I think it just took them down. So yeah, that really was not helpful at all.

Moderator 58:39

25?

25 58:42

Yeah, so I, as I mentioned before, I had one professor who didn't really adapt to the online learning structure very well. And I feel like he kind of got frustrated with the online, the online learning structure and kind of, in a way took it out on students. So it really made, impacted it-- A lot of students grades -- because ih, he would refuse to accommodate a lot of students needs and stuff. So yeah.

Moderator 59:10

17, what are some things that your professors did that made it harder to learn

17 59:15

For some of the live lectures that they did, just having it being live made it very long and boring to rewatch, because like, people would mess around in the chat, the professor wouldn't know what they were doing. And the same questions would be asked over and over, basically.

Moderator 59:33

I see. So were those live lectures purely live, or would they be posted later?

17 59:40

They'll be posted later but the thing about that is like, I wouldn't go to the live lecture, but I would rewatch it. I would watch the pre recorded one later. It would -- it would just be filled with so many like mistakes, so many interruptions and everything.

Moderator 59:54

Sounds like more distractions in there. 39, what are some things that your professors did that It made it harder for you to learn.

39 1:00:03

So one of my professor, it was a harder course for my major. So he wasn't taking any class at all. No Live Meeting, no zoom session, no office hours, nothing. And I don't know, maybe he was having some issues with his communication, but it was very hard to get in touch with him. He was just... with all due respect, he was just posting notes, each chapter six, seven pages, and then reciting the notes in an mp3 recording. That's it. After two, three weeks, he post some questions. You have to write the answers and upload that --that's it. So didn't learn anything from that class.

Moderator 1:00:49

So just posting notes and that was kind of it

39 1:00:54

Yeah, no communication, nothing.

Moderator 1:01:01

My next question is, what are some things your professors did during online learning that made it feel like they cared about their students? 31?

31 1:01:09

well, I mean, for me, like I said, um, they just became a lot more active. In their learning and not to say they weren't active, or in some of them not to say they weren't active, like, in person, but when we went online, they became much more accessible, and stuff like that. So, yeah.

Moderator 1:01:34

And what kinds of things do they do to show that they were accessible?

31 1:01:38

Um, well, for one of the for one of my professors, as you could say, is he used to have like a set time for Office Hours like, like Monday 6pm in my office, but what he did was that he said Monday through Friday, here's my personal number. And here's my email, call me or text me from four to seven. If I respond to you hop on zoom, and we can have a quick office hour. So here's an on demand office hour, which I thought was very helpful.

Moderator 1:02:11

Um, how about you, 25? What are some things that your professors did that showed that they cared about their students?

25 1:02:18

A lot of my professors offered office hours and personal office hours and many of them gave their personal cell phone numbers and stuff that we could reach. Reach to whenever we have certain questions given given a specific time. So like office hours with virtual

Moderator 1:02:39

okay. 17?

17 1:02:41

My professors didn't go out of their way to give us their number or anything, but I would say they had more office hours, and they just responded faster to emails and like any student concerns or anything, so there is that.

Moderator 1:02:55

Okay, so being responsive, and then 39, what are some things that you're Professors did to show they cared about their students.

39 1:03:02

So as I mentioned, some professors were active all the day on the forum. They were like active all the day some profess-- So one professor, she created a whatsapp group where it was not about the study. It's like it was when it was going very bad. And March later march in April, she used to like, personally ask everyone, "how are you doing?" and sending some quotes, some funny videos, like to cheer everyone up. So yeah, that felt very personal. And the connection.

Moderator 1:03:36

So going beyond just schoolwork and maybe people's personalized checking in with them.

39 1:03:41

Yeah.

Moderator 1:03:47

So what are some things on the other hand that your professors did that might have shown that they did not care about their students.

31 1:03:56

One thing that my professor did, maybe-- Maybe I took it the wrong way. Maybe I didn't. But she gave me a midterm that took me over 15 hours to complete and I had only a single day to do it. So not maybe, maybe she didn't realize how long it would be. Because the thing was only like 10 questions, but the fact that it took 15 hours out of like a regular day, she may have shown that she didn't care about that, or I don't even know but 15 hour midterm is a very interesting, to say the least.

Moderator 1:04:40

Right and 25?

25 1:04:44

I'm sorry. Okay. can You repeat the question.

Moderator 1:04:46

Sure. Um, what are some things your professors did during online learning that made you feel like they didn't care about their students?

25 1:04:54

Oh, I'm sorry, as I mentioned for one professor who, who actually made the grading rubric even harder. So, previous for pre COVID he were graded on on two, we were graded on three exams and they were more spaced out and he would offer -- he would offer some leniency like say, if you did one, if you did really poor on one exam and really uh, good on the other and it showed like massive improvement he would take that into consideration. Whereas ever since it on switched online... he-- we only had two exams and the last one was worth on 60% and the other one was 40% so-- it was it was really a stressful situation where if you-- if you -- if you do well it really well on the one exam and then poorly on the other, it would be very unlikely for you to pass the class or --- and he just really made the graded Rubric really kind of unfair and um, not accommodating to students needs in the current pandemic.

Moderator 1:05:55

17, what are some things your professors did that might have shown that they didn't care about their students?

17 1:06:00

All of my professors gave us more homework and that homework took more time than a normal homework would take in person. And on top of that, for one of my classes, all the midterms are normally multiple choice. But during the pandemic, instead of doing multiple choice, my prompt required us to write a two to three page essays.

Moderator 1:06:23

39, was there anything that your professors did to show that they didn't care about their students?

39 1:06:28

Yeah, so I mentioned about uh, one professor, it was very hard to communicate. And also I was taking one art class where we had to visit a place, like artwork, take a selfie and write a paper on it. So I felt this is very, like, disconnected with everything going on. You have to go in person take a picture, it felt... I personally felt like the professor doesn't care about us. Sending out sending us out there. So yeah, other than that, no

Moderator 1:07:01

And now we're at the last three questions. We're going to talk about how your university responded to the move to online learning. So what are some things that your university did? Some examples are like pass fail options, town halls or remote counseling. To help students be successful during online learning, you can mention any resources the university provided or actions they took to help students be successful or feel supported.

31 1:07:29

My University just gave us pass/fail, and they also made counselors like post uh, online times when you could contact them. And other than that, I believe that's it.

Moderator 1:07:46

25?

25 1:07:48

Yeah, so similar to 31 um, My university recently went to an optional pass/fail. So if you didn't do well, and of course you could opt in for the uh, fail and then you could keep your letter grades If you did well in the class

Moderator 1:08:03

17 what are some things that your university did?

17 1:08:06

My University also went pass/fail. And in addition to that, like any resources we would need such as academic advising or psychological counseling-- Everything moved online.

Moderator 1:08:20

And 39?

39 1:08:22

So yeah, the transition to online was very fast. Also, they were providing loans uh, laptop to borrow, to the students. And they kept the food pantry open. Like, even in the lockdown, it was open. And they're so all University partnered with-- I don't know which company they were providing our internet services for two months free. And also the office, they delayed the date of the payment for students so yeah, it was very good. Good gesture.

Moderator 1:08:59

So If you like, technology and then also, financially it sounds like.

39 1:09:06

Yeah.

Moderator 1:09:08

Okay, and are there is there anything that you wish University did better to help students be successful during online learning?

31 1:09:17

Um, I wish I wish my university had a lot stricter guidelines to reduce cheating during tests. Like, just in general, because like I already mentioned before, but I feel like a lot of my like anxiety and like stress from the semester was literally just from worrying about the curve being destroyed by cheaters. So not only did I have to study, like a lot more, like I studied a lot more even though like, I breeze through lectures, just because like I just had to have, I just had to have this mindset of, I need to get 100 or I'm going to get screwed out of the curve. And think, thank God I did good enough to do how I wanted to do Because a lot of my friends they got they had to pass the class even though if we took it in person, they would have done much better because just because of the rampant amount of cheating because, like I understand like they can't really stop cheating but I do think there are necessary precautions that you can take to reduce it. Like for one don't make the test as hard or to just like just like Proctor track everything because it's extremely difficult to cheat during Proctor track, but I don't know why they didn't want to spend the resources to do that. But I feel like if they did that it would relieved a lot of stress out of a lot of students.

Moderator 1:10:36

25 what are some things that you wish your university did?

25 1:10:40

Um, I think my university handled it very well I don't like nothing comes to mind where I could say I think that um they could have done it any better because they did enact the pass/fail option. So even if your professor was uncommon, unaccommodating, you could opt in for the pass/fail and then yeah

Moderator 1:11:01

Okay, 17 is there anything you wish that your university better?

17 1:11:04

No, not really. I think my university did a pretty good job. Besides, besides, like all the cheaters and everything, they did a pretty good job with everything.

Moderator 1:11:12

okay. 39, was there anything that you went to university did better?

39 1:11:18

No, no, I felt like it was it was they did a good job on. However, for one or two classes. We were very confused until the very last days. Is there going to be a final exam or not? So yeah, that part. We --we--We weren't very sure. Other than that, yeah. They did a good job.

Moderator 1:11:41

And then my last question, in general, sorry, in general, are there any ways that your ability to be successful in school was effective in the excuse me, sorry. I'll just start over in general are there any ways that your ability to To be successful in school was affected by the pandemic that we have not discussed yet.

31 1:12:05

Um, Um, I don't know, I just feel like I feel like we could have focused more on like the mental aspect of the pandemic because even though we did touch on like yeah, you get more responsibilities is that but I just think the mental aspect of losing a semester in college, um, like just kind of like acclimating out of college and then expecting us to maintain the same amount of rigor is uh-- is kind of, difficult for a lot of people and and honestly I already explained but the hardest thing for me was just like the cheating aspect and I don't know how you're going to grade on a curve when you when this kind of obviously be cheaters In my opinion, but it was beyond me. So yeah.

Moderator 1:12:58

Would you like to add anything that can be successful in school during the pandemic?

25 1:13:05

Um No. I am good

Moderator 1:13:08

17?

17 1:13:09

would say like not having a outlet, like going out with friends or something besides focusing on academics all the time, and not not being able to do anything. I feel I feel like really hindered me.

Moderator 1:13:23

39, was there anything else that affected your ability to be successful?

39 1:13:27

Uh, sure, no. Most of the things were discussed, but as a general comment, I'd like to add, so I, I think we could have discussed more about financial stuff because a lot of people were laid off. So I think it might have affected some. So yeah.

Moderator 1:13:50

Okay. Thank you all for sharing. That's the end of my questions. If you could just email us the name you used during this um, during this focus group and then after that, once we confirm that your survey is completed, we will send your gift cards and again, thank you all for sharing today.

**High SES Asian Men**

Moderator 0:47

Okay, it's been a couple minutes, so I think we're just gonna go ahead and get started. So, welcome to the focus group. My name is Moderator. I'm a research coordinator from Redacted. Moderator is going to be our note taker for today. So Moderator if you want to say hi, you can do it now.

Hi everybody.

We will be asking a number of open ended questions. There are no wrong answers, but people may have different points of view. Please feel free to share your point of view, even if it differs um from what someone else may have said. Keep in mind that we're just as interested in negative comments as we are positive comments. We just want everyone to feel comfortable participating. So I'm going to encourage everyone to talk. Because we have to keep this to an hour, I might have to cut you off so I apologize for that in advance. We want to get the full breadth of experiences and opinions, but some of you may have very similar experiences and opinions and if that's the case, you can just say my answer is the same as Sam's for example. It is difficult for us to take notes on everything that I said that you say in the group, so we will be also recording the session so that we can transcribe it later. But we're not going to associate your name with anything that you say here today in focus group. And you have the right to refuse to answer any question that we ask here today. We understand that it's very important to keep this information private and confidential so we'll ask participants to respect each other's confidentiality and not repeat things said within the group to anyone outside of the group. Are there any questions before we get started?

Unknown Speaker 4:04

No, I'm good. I'm good.

Moderator 4:07

Cool. Um, so let's just get into it. Um I'm going to ask that everyone go around and say your name and what year of college you just finished and one word to describe what life has been like for you during the pandemic.

26 4:27

Uh hey, I'm 26. Uh I go to REDACTED University. One word would probably be hectic, I guess. Hectic's a good word.

Moderator 4:39

Okay.

37 4:42

Uh I'm 37. Uh I go to REDACTED uh University of REDACTED. And it's been strange to go around waiting for

41 4:55

I'm 41. I just finished my freshman year at REDACTED University and one word to describe how the pandemic has been, I I have to say just interesting.

Moderator 5:06

Okay. Um so COVID-19 has presented big challenges for undergraduate students. As you know, universities across the nation have closed moving all institution online with very little time for students or faculty to prepare. Many students have encountered challenges related to the closing of campuses, relocation, and the movement to online instruction. In this study we want to learn how the closing of campus and the pandemic in general has affected you. We want your opinions about the good and the bad on how things went and as well as as your pro- how your professors and universities um performed. So let's start by discussing where you went when campus shut down. 26, if you would like to start again.

26 5:52

Okay, so, my camp- camp- so my campus is in REDACTED, obviously, but I'm from REDACTED myself. So once thing shut down I moved back home to uh REDACTED, REDACTED with my parents. And that's where I live. I've been living since.

37 6:13

Uh so yeah, uh when the campus closed I I had to go back to uh REDACTED, uh which is in REDACTED near REDACTED. Uh yeah. So that was it, right?

Moderator 6:27

Mhm.

26 6:28

Uh REDACTED is in REDACTED, the capital of REDACTED and I just live in REDACTED. It's like two and a half hours north. So I just moved back home immediately after campus closed. Well we were on spring break already, and we just never returned.

Moderator 6:40

Okay. And now can you all tell me about the space in your home that you moved to? Like what kind of environment did you have to do schoolwork, if it was private, or if you shared it with someone? Things like that.

41 6:51

Uh I mean for me, um so I have my bedroom, and what not which I used to work in throughout high school, but I found it more productive we have a small office space and I ended up converting that area to just kind of where I would attend my classes or zoom meetings and whatnot. And it's where I am right now. So it kind of shifted and been working out of a private office, I would say. Yeah.

Moderator 7:19

Okay.

37 7:22

Uh so I have a study and everything in my room, so uh uh so it was pretty comfortable. So, yeah.

Moderator 7:30

Okay, and I'm assuming that's a private space as well.

37 7:33

Yeah yeah a private space.

Moderator 7:40

And 26, can you tell you about the space that you moved into?

26 7:44

Uh just a pri- just like my own room, my own room in in the house, and so it's pretty private I would say, yeah.

Moderator 7:49

Okay. Okay. Um, now what challenges did you all encounter attending classes or doing your schoolwork in the space you were in? And 41 you can start us again.

41 8:00

Uh I would say consistency was a big thing, consistency and uh focus. It was hard to adjust because now you're going from uh being in a classroom environment where psychologically when you step into that classroom, you're thinking biology, right? Or if you walk into chem class, you're thinking chemistry. And now you're home. And you know, anytime you're home, when you're away from college on break, you're on break. Your mind enters the mode of break, but now you have to do school from home. So uh what was the question again? Sorry, I don't want to get off track.

Moderator 8:31

Oh, it was just what challenges did you encounter attending classes or doing schoolwork in your space?

41 8:37

So I think adjusting was a big thing in the sense that you have to force yourself to, okay, I'm going to the office now to work because this is where I'm going to be doing school work versus earlier, it would have been I'm going to class and now I'm going to be doing school work there. And I think adjusting wise I think learning was a lot harder because you didn't have that physical um the physical recognition of okay, I'm in a classroom where I'm gonna learn now. You're just in your house.

Moderator 9:08

Okay and 37, what kind of challenges did you experience in your space?

37 9:15

Uh so definitely the timing of the class was uh really uh difficult for me because uh because of the timezone and stuff. Uh so that was a great challenge to that to wake up in the middle of the night to attend cla- some of my classes. And often I missed my classes because, you know, uh cuz the schedule and stuff and the other thing um what uh 41 exactly said so it was it was a challenge to get in that uh thought through mindset inside my you know room.

Moderator 9:45

Okay, okay. And 26.

26 9:49

Uh I would say um just commitment and just, I don't know, motivation is probably the biggest one for me. I don't know just something about sitting at home, and like in this and then like, in like a room I mean. Attending class just made me like lose motivation. And so it was very difficult to keep going to class. I'm not sure what what why exactly, but I just didn't feel like studying anymore or like attending class and there were like plenty of times when I skipped because I just didn't get motivated.

Moderator 10:21

Okay. So what are some things that you or the other people in the house did that made it easier for you to attend class and do your schoolwork?

26 10:35

I think there became like a, I had to communicate my family, but because I was working in office space, it's kind of like, okay, hey, everybody, you guys are doing what you're doing, but heads up, I'm going to be in class for the next hour and a half. So I think communicating with my family and I think the respect and understanding that okay, this is going to be a different type of environment and now that he's home and he's attending classes, he's going to need that time to himself because he's going to be doing schoolwork. Because at the end of the day, you're still in school. So I didn't think communication and um understanding with family as uh in terms of okay, what do I need to continue attending classes and what, what they can do with anything, which in this case was do nothing and just let me do what I need to do. Right.

Moderator 11:14

Okay. Okay. That makes sense. 37, what about you?

37 11:23

So yeah uh so uh when I when I have uh tests and stuff, like uh the audio is being recorded, so I had to tell my like, my family members, like don't come inside the room or ask me what I'm doing want to hear anything or something like that. So do you understand that I'm having tests and stuff? And uh that's it.

Moderator 11:41

Okay. 26.

26 11:46

Can you repeat the question one more time?

Moderator 11:49

Yeah. What are some things that you or other people in the house did that made it easier for you to attend classes or do your schoolwork?

26 11:57

Oh okay. I guess my parents making food was a big one. Like they would just randomly bring in food if I was hungry, and that kind of just gave me energy. And uh also just watching like movies or tv together that like brought spirits up in the house in general, just because I there was nothing else to do, because everything was in lockdown. So yeah, I would say those two things helped a lot.

Moderator 12:20

So it sounds like socializing with your, the people in your house actually made your matters better.

26 12:25

Definitely. Definitely.

Moderator 12:26

Okay.

41 12:26

Can I can I actually add to that real quick?

Moderator 12:28

Sure.

41 12:29

Cuz you brought up a good point. Um, 26, because that's another thing is, I felt like when I'm home now, because I have family and and, you know, the washing machine is not like, okay, when is it available? Right? And so like okay, I need to go cook my own food or I need to go to the dining hall. It's all in your house at that point, at least in my case that's what it was right. Where I had to go to the dining hall and whatnot. So a lot of your resources and needs if they weren't taken care of they're at least easier to access, which gave you more opportunity if you decided to take it, to focus on your school or whatever it is.

26 13:02

Yeah, definitely. Definitely, definitely agree with that.

Moderator 13:05

Okay, okay.

37 13:07

Me too.

Moderator 13:08

All right. So if you live with siblings, to what extent were there differences in how much your parents supported each of you in your ability to attend class and complete schoolwork? So for example, were there some kids in the house that received more time and space or consideration to do school by parents than other kids in the house?

41 13:32

So, since you're asking in terms of how was the effort different from the parents' aspect towards each of us?

Moderator 13:38

How. Yes, I'm asking what are the differences in the support provided to each sibling, if you notice any differences?

41 13:45

Okay, so I have two younger siblings, one in high school and one elementary school. And so the difference in support there I would say is, there was less support of checking up on me, right, whereas for the elementary schooler it was, hey, do you have class today? Hey, did you do your homework? And so I think you're more supportive, I don't know if supportive is the right word. You're more concerned with the younger sibling, because for them online, it's like, okay, they need to learn how to use a computer. They need to learn how to use zoom, right? Whereas it's easier for us in college because we know what we're doing or we're expected to adjust accordingly ourselves. So there was more attention given to the younger siblings in that sense, and uh more checking up on them and you as a college to you're left, you know, as the oldest independent to you you're trusted that you're going to take care of what needs to be done.

Moderator 14:38

Okay, so it sounds like independence as a result of age.

41 14:42

Yeah.

Moderator 14:43

Okay. Okay.

37 14:47

Uh for me, we have the same consideration because uh my sister she's a year older than me, so she's uh she's a sophomore now and uh I mean, yeah uh second semester sophomore. And yeah uh yeah so it was it was basically equal for me. So yeah. There was no special consideration for either person. It's always been like that so.

Moderator 15:10

And when you say it's always been like that, you mean it's always been like the same amount of support for the both of you?

37 15:15

That's right. That's right.

Moderator 15:16

Okay, cool. And 26, what about you?

26 15:20

Uh I'm actually an only child but I could talk about that if you want want me to.

Moderator 15:24

Uh if you have something to say you can, but if you don't have siblings, then don't feel like you have to say something.

26 15:31

Okay, okay. I guess, because I'm an only child like I'm the only one my parents have to focus on. So that just helped a lot.

Moderator 15:37

Okay. Okay. So now let's talk about what other responsibilities you had after campus closed, and how these different differed from responsibilities you had before campus closed. Describe your caregiving responsibilities including child care, elder care or caring for ill loved ones, and how they were alike or different from your responsibilities before the shutdown.

26 16:02

I think um my responsibility around the house you know, it's as if I never left to go to college. It's back to, you know, are you doing your own laundry? Are you keeping your room clean? Right? Some on some days okay do I need to clean this? Okay am I I'm responsible for maybe vacuuming or something. It becomes you're back with living with families. Everybody has to take care of the house because we all live in it. So responsibilities in that sense increased because it's not it's not there's no longer you know, one person in a dorm or one person two people in an apartment. You're back with your family so although you have school and that is your primary focus, it became okay, now you're around your sisters all the time. Are you ensuring that their education is good. Right, if my younger siblings needed help with something, I have to I don't want to say I have to be more of a big brother but because I'm physically here, I'm not on campus, I'm expected to you know aid them, be support for them.

Moderator 16:54

So it sounds like more responsibilities. Both with taking care of the house and taking a little bit of care of your sisters.

41 16:59

Exactly, yeah.

Moderator 17:00

Okay.

37 17:03

Uh so I've never had to be responsible for uh it because uh I didn't have any younger siblings or anything so and um so all I had to care for is like myself when I was in college, and I think uh coming back home, it's like, I think my responsibility has decreased because I know I like I uh no longer have to go to the, you know, the cafeteria to get food. It's always on the table. And you know, I don't have to do my uh laundry cuz you I have people to do that for me and stuff. So I think I've been uh less responsible, you know, getting back home, if that makes sense.

Moderator 17:40

Okay, because your parents are doing more of those caretaking responsibilities.

37 17:44

Well, not exactly my parents. Uh we have uh people that uh like uh maid and stuff.

Moderator 17:53

Okay, okay. All right. And 26, what about you?

26 18:01

Uh wasn't there. There were no siblings. I don't really have any caretaking responsibilities that I had to take over I I would say.

Moderator 18:07

Okay.

41 18:08

I I think an important distinction real quick. Sorry to. But then

Moderator 18:12

No need to apologize.

41 18:14

Yeah. Uh both of them, you guys brought up good points and it made me think yeah it in it's not that responsibilities are maybe less or more, it's that they've shifted, I think. Because you guys brought up good point, right that, you know, maybe now I'm not worried about getting food as much, right. But now because I'm home, I'm worried about the home. I'm worried about my family because I'm around them more. So I think in my case, personally, responsibilities have shifted, not really more or less, but they've become different.

26 18:38

Yeah, that's definitely true. I would agree with that.

Moderator 18:41

Okay, okay. So, um this is a question touching on some stuff we already talked about, but if you have more to contribute, now's the time. Describe your other household responsibilities such as chores, cooking, caring for pets during this time, and how they were alike or different from before school closed.

41 19:02

I mean, as I said before, it's um I mean you're back to being, I don't want to say you left your family right, but you're back to being a part of that family. So so you're expected to contribute to okay, you know, maybe I need to, maybe I do need to make breakfast for myself a couple mornings, right? Maybe I do need to help my sister out with her homework, etc. So, in that sense, I think that the only thing that's changed is, is your social responsibilities, because you're around your family all the time. Um, I think that answers the question.

Moderator 19:35

What do you mean by social responsibilities?

41 19:38

In the sense that now you are you know you're not away from campus and you're not, you know, away from them. You're with family now. So it's kind of, okay. If I can go see grandma on the weekend, and we've been quarantining, then, you know, it was kind of expected in terms of just our family dynamic in my situation. It's just our family's all very close. It's kind of like okay, you know, you're expected more to check up on your other family members now that you're home, versus you were in college and you're away, we understand that you're busy doing your own thing and you have a different life there.

Okay, okay.

37 20:11

Uh could you repeat the question? Sorry.

Moderator 20:15

Sure. Um, describe your other household responsibilities such as chores, cooking, or caring for pets during this time and how they were alike or different from before school closed.

Did that clarify the question?

26 20:44

He might have forgotten to umute.

37 20:46

Yeah, yeah, my mic was off for that. Okay. Uh so yeah. Uh so before the school closed, you know, um I had to make uh food for myself sometimes because I would miss the cafeterias or something and um I had to do my own laundry and like I I think after the school closed I had, like, I I didn't need to worry about those things. Uh as I said before, because uh uh I had uh I had people, you know, taking care of that stuff so. So yeah.

Moderator 21:16

Okay, that makes sense. 26, to what uh what other kind of household responsibilities did you have after the school closing?

26 21:28

Uh laundry was a big one. Just having to take over like washing, washing and drying clothes and obviously we have a washing machine. So that so that's like not like, a as huge of a thing as it was in college where you had to, like, make the trek to the laundry room. Um cook cooking cooking was handled by my parents, but back in college, I had to like make a trip to the dining hall or to the restaurant every time I wanted food. Um, what other chores? I would say that's about it. Other than that, I just, I I would clean I would help help if I was cleaning the house sometimes, but that was only like once a once a week or once every two weeks, but that's about it.

Moderator 22:07

Okay. And finally, to what extent were you expected to take on more household work, child childcare, and or family caregiving responsibilities than other people in your home? So for example, parents, may have expected more of one sibling or partners or roommates might not contribute equally.

41 22:29

I think what it became is, you know, in terms of the list of responsibilities, yeah, they're different, right. Okay, you have to maybe clean your own room more or because it's not that you know I'm I think my room is fine. But if my parents don't, then you don't want to have that rift, right. So you're expected to do that, but I think what it comes down to is also the fact that at least in my scenario, um the parents were understanding of okay, school first. So if needed, right, if there was some days where they're like, okay, hey, why isn't your room clean? Or hey, dude, why aren't you know, help your sister out with some homework. And if I'm sitting here and I'm thinking, well I have a test to study for, they're willing to put, you know, my online education higher above any responsibilities. So in that sense, they were given to me, but if education was a priority for me at that time, the responsibilities could be negated as so far as. Now one thing they told me was just, you know, we can give you anything you need. Now that you're home, like we'll we'll do your laundry gladly, we'll cook for you gladly, just make sure that you're, you're adjusting well to online and you're taking care of education.

Moderator 23:36

Okay, that makes sense.

37 23:40

Uh so before the uh school closed and after school closed, the only thing I've been responsible for is um uh, you know, getting my education on track and uh making sure my grades don't uh drop and stuff. So, I think that's that's been like the primary thing uh for me before and after, and as I said before, before the school closed um, I had like a few responsibilities for myself like to take care of myself. Other than that, I don't think there's been much to do.

Moderator 24:14

Okay.

26 24:18

I would say for me responsibilities they, I wasn't given any major additional ones, but I just since everything was in the house, and my parents would help out with the food and whatnot, some responsibilities are reduced. And so I could focus, they were like, just focus on your academics, similar to what 41 said.

Moderator 24:37

Okay, that makes sense. I'm hearing some, um some common themes here. So now let's talk about how your academics were impacted by the shutdown. I will ask how the shutdown was, has impacted a variety of things. If you don't think it impacted something I ask you about, just say no impact. So first, in what ways, if any, has a shutdown impacted your academic performance, or grades, and your understanding of the subject matter of your courses.

41 25:06

It's impacted my grades. It it shouldn't impact them in a healthy way, but it did. It did because realistically speaking, every like you said earlier, you mentioned in the beginning, teachers, everybody had to adjust in a short period of time, versus how in the fall, professors are having time to come up with the course content and ways to teach the class that will fit the online style. But when everyone's thrown into this, a lot of professors, they're human, just like us. So we're all trying to adjust in a short period of time and what that turned into was, it's not perfect. And therefore, it kind of became a little bit easier to do better in some classes, because of that sense. So because of that, it impacted my grades in a positive way.

Moderator 25:54

Okay, and what about your understanding of the material? How was that affected by the closing?

41 25:59

Oh, that was definitely negative. (laughs)

Moderator 26:00

Okay.

41 26:01

I I hate to say it, but um when you're thrown in a situation like this, there's only so much that professors can do and especially if you're already used to being in person and you're in your into a rhythm, right, we be- you become accustomed to this process of going to class, of learning material this way, of you know, this is the schedule. This is when tests are, this is when homework is, and now when that all that is shaken, right, when your foundation and what you're used to is shaken, it becomes a lot harder to digest that material as well. And I mentioned this earlier, but physically being in class or physically, uh knowing that I'm doing this subject at this time, it it it psychologically become used to it and you do better in that sense. But now when you're attending six different subjects from the same chair, in your room, it's easy to get mixed up and it's easy to just skim over material for the sake of short term memorization, but long term memorization, I think and understanding was definitely negatively impacted in my case.

Moderator 26:58

Okay.

37 27:02

I completely agree with 41. So yeah. For most of my tests, I memorize all all of it for like a short time. So my understanding was definitely negative, than you know, uh what I would have understanded if I was in class and you, if I was interacting with teachers and asking questions more in class, and stuff like that. Um and uh but as far as my grades are concerned, I didn't I don't think it affected me much because uh because I think the tests were made easier. Some people that I found it was easier than the tests were easier than what I had to do if I if I had in person classes and stuff. So um other that, if I had to be if I had to be very specific, uh I have a I have chemistry lab as well last semester and the the only grade drop I had was uh for chemistry lab because uh we were only able to do about three to four labs and uh from out of 25 labs if they had to take an average of three to four labs, so wasn't that great because I didn't do well in uh in the first, you know, first three of my labs. So now other than that I didn't I don't think uh my grades were impacted that much. And um so like I said, um understanding was uh decreased when uh compared to uh the class uh which was um in person.

Moderator 28:22

Okay. And go ahead.

26 28:28

Uh I would say my grades were positively affected. Like the professors went out of their way to like, make sure that uh the reading was pretty lenient, and thing and it was hard, it was easier to do well in the class just because of the crisis. And like, I'm, like, my, my family's pretty well off, but I assume for like poor families they wanted to make sure that they weren't affected and so that kind of spilled off onto me even though it wasn't directed towards me. And so I was able to do pretty well grades wise. But in terms of understanding the material, um I would say, I understood it maybe like 80% as well as I would have if the pandemic hadn't happened. Uh the main the detractor was just the cla- the hands on, like laboratory classes that those kind of got really jumbled and we had to do some weird online lab, which just wasn't just didn't work. And so, I would say that just the lack of hands on, I'm an I'm an engineering student, so the lack of hands on classes just really detracted from the education but anything that was more theoretical, or um lecture based, I was still able to grasp pretty well, I would say.

Moderator 29:45

Okay, okay.

41 29:46

I think it's a coincidence all three of us had lab classes, but yeah, definitely, labs were hit hard just because, you know, in my case, if we were supposed to be doing dissections of jellyfish you really don't get the hands on um like 26 was saying. And so it's just there's no way you can go around that right. Because it's just not hands on. You just can't do it so understanding material that way it's different looking at a picture versus actually doing it yourself.

26 30:09

Yeah, definitely.

Moderator 30:11

Right.

37 30:11

I agree.

Moderator 30:13

Okay, unanimous conclusion. So in what ways if any has the shut down impacted the availability of other learning opportunities, like internships?

26 30:25

Um so, so much. I was planning to shadow this summer. I'm a pre med student, so I was going to shadow this summer. And um you know, I was in the middle of the process in February, but then as soon as you know, COVID hit um. I mean, no doctors' letting you shadow. They want to minimize anything, right. So internship opportunities, and I know personally, friends who've had internships with Amazon internships with like, other companies as well and they all get cancelled or some of them gone moved online. And it's just not the same because as we've seen, talking through this conversation, ,uh there's no substitute for in person, whether it be um classes or internships, etc. And in terms of what was the other part of the question? There was internships and there was another

Moderator 31:13

Oh no, it was just such as internships.

41 31:15

Such as yeah. So just it it negated them and it's kind of it it pushed us back in terms of pre medical students.

Moderator 31:26

Okay.

37 31:26

Uh for me, I wasn't looking for any internships and um I had I wasn't uh doing any internships so it didn't affect me.

Moderator 31:37

Okay.

26 31:40

Um prior to the the like pre pre pandemic, before anything happened, I had an offer like from this pretty cool uh 3D printing company in downtown REDACTED and it was it was going to be pretty cool. I was pretty excited. It was in the middle of downtown, and it's gonna be awesome. And then once the pandemic happened, they obviously canceled. And so one of the professors I work with for research, managed to like land managed to land me an online internship with another company that he had connections to. And I did that. I like I just completed that. And it was all right. I mean, I obviously it wasn't terrible, but I just didn't get the same ex like experience that I would have, like, you know, going to an actual company and actually working hands on with other engineers. Because this was just like a bunch of zoom calls a bunch of online work. Um they send me some parts and some engineering engineering work to do but it just wasn't the same and I definitely feel like like it's I'm so grateful I got to do an internship, but I the online internship just doesn't work as well and uh I would have vastly, vastly preferred the internship offer I had at the start.

Moderator 32:50

Okay, understood. Did new learning and or career development opportunities emerge during the shutdown, and if so what were they?

41 33:04

New career opportunities you said?

Moderator 33:07

Mhm.

26 33:08

I don't know if this answers your question. In terms of, I wouldn't call it a career opportunity. It was a volunteer opportunity if you want me to expand on that I can.

Moderator 33:16

Sure, go ahead.

41 33:17

Um, so I do work with a nonprofit that one of my high school teachers started and I've been greatly involved in since then. But obviously, when the pandemic hit, like we just talked about a lot of internships got moved online or they canceled. I can't shadow anymore, but it did bring about opportunities for you know, aiding other people. And one of the opportunities that came that I took advantage of was with that nonprofit, you had the opportunity to gather supplies, groceries, hygiene products, and keeping our social distance, our mask on, deliver them door to door to families who are not, you know, cannot afford to get these products or you know, do not wish to go out during this time. And so in that sense you had the opportunity arise if you're able to. I think delivery I mean, we know Amazon I mean delivery skyrocketed because they capitalized on the opportunity. No one's leaving their house. So in that sense, the ability to provide for others, that opportunity arised. Because on day to day basis, why would you go deliver hygiene products to anybody right? You would you'd maybe have a food drive that people come to, but going to other people's houses, one on one is a different opportunity that arose.

Moderator 34:24

Okay, interesting.

37 34:29

I had no impact, so.

Moderator 34:31

Okay. 26, did you notice any new career or professional development opportunities arising after the closing?

26 34:41

Uh I guess just the the only one would be the online internships which I mentioned before, those there was kind of like an like a a rise in those mainly. Um those long internships are they're all ri- I'm I'm grateful that they that they exist and they were I definitely learned stuff, but they're obviously not the same. Um in terms of other opportunities, there was also, I was working in a research lab prior to prior to the shutdown. After the shutdown, we continued our work except remotely. So that that also was not as effective just because we were not working actually in in we were not working in the lab anymore. But yeah, I would say the main one is the online internship.

Moderator 35:26

Okay. So in what ways, if any, um sorry. In what ways, if any, has the shut down impacted your confidence in your ability to successfully complete your bachelor's degree at all or in a timely fashion?

26 35:45

I don't think it's impacted it. Um, classes although the transition was hectic, they continued. And uh credits were given, you know, assuming so but even we had the opportunity um what REDACTED decided was because of the transition, you could decide whether or not you want to have a pass fail given for a class instead of a letter grade. And regardless of that, even if you passed, you would still get credit for that class. So, um, it wasn't affected. I'm still on track. Uh I don't think anybody that I know personally has gotten off track in terms of the degree.

Moderator 36:23

Okay. And we will ask about pass fail options later as well.

37 36:27

Uh no impact.

Moderator 36:32

Okay, 26.

26 36:35

Can you repeat it one more time?

Moderator 36:39

Yeah. In what ways, if any, has the shutdown impacted your confidence in your ability to successfully complete your bachelor's degree at all or in a timely fashion?

26 36:50

Okay, I would say it uh it actually accelerated it because basically, I was able to sign up for community college classes that were online and since they were online they were much easier. And so I'm I'm actually graduating a semester early because of the pandemic, because I I was able to catch up on credits.

Moderator 37:08

Okay. In what ways, if any, has the campus shut down impact your finances?

41 37:15

Uh I mean, we had, you're not paying as much for food, mostly because now it's your family taking care of groceries and whatnot. So it's not coming out of your own pocket. So personally, I think um we saved but uh as a family um. I mean, you're in the house more so your bills go up a little bit in terms of heating, in terms of electricity, which I think is expected and it's something that we'll see you know, nationwide, not just finances, I think, you know, actually end up saving.

Moderator 37:52

Okay.

37 37:55

Uh for me, uh definitely saving because, you know, I didn't have to pay rent for almost a a semester. And uh because uh the campus was shut down early, you know March when the almost the semester started and um, and yeah, definitely I'm sure that's it. So, yeah, it was a it was a positive you know, um financial thing.

Moderator 38:22

Okay.

37 38:22

Getting money.

Moderator 38:24

Okay.

26 38:26

So I would say definitely saved money. Uh my university refunded the the rent, um they refunded dining, uh and then I obviously am not paying for things out of my my own money. My parents are paying for it. So, overall it was just a big saving of money for us.

Moderator 38:45

Okay. Now let's talk about what your professors did and did not do during the move to online learning. What are some examples of strategies, tools or technologies that your professors used, that you found to be very effective during online learning?

26 39:03

For the most part unanimously, my professors, um not all of the ones, but the ones that did put effort into transitioning online, it was zoom calls. It was zoom lectures. And um to expand on that, you know recording lectures, and uploading them. Um what I did not find effective was professors who did not hold zoom lectures, but just recorded on their own time and uploaded pre recorded material versus uh professors who hold live zoom meetings and will upload it afterwards for review. I think there was a difference there. I was more likely to attend a live session than I was to just go and look at already pre recorded material, but those were, that was the big thing that most professors did.

Moderator 39:47

Okay.

37 39:50

Yeah for me as well. So whenever we had live recording classes um, it was it was more of a classroom experience and it was easier to focus uh in class, and uh whenever I didn't have a, you know, uh when I had a pre recorded session, and uh I was I could access it anytime of the day, uh that being a positive thing, I wasn't able to focus as much in the past. Uh and uh yeah, and that's it. And uh so our school has a, you know, a a online teaching platform. So and uh they moved it, they shifted to the classes to it and that was that was very interactive as well. So anytime I had questions I could just post it on they had a discussion board and stuff, so. That was helpful.

Moderator 40:40

Okay.

26 40:42

Uh we we had a discussion board as well, which was also very helpful. Uh we got responses in a timely manner. Um posting lectures online, so you don't have to attend class is also really helpful. A number of teachers a number of teachers shifted to an asynchronous format rather than a synchronous one and I I vastly prefer an asynchronous format, just because I think it's much easier to learn. Like when when you're in this pandemic situation, it just makes more sense to be asynchronous, because you're not being pressed to attend at a certain time. You can do it at your own convenience and that, that allows you to tackle the material in in the fashion that you see best fit for yourself, which in turn motivates you a bit more. So I really like the asynchronous format, and as as as well as the posting of zoom lectures.

Moderator 41:34

Okay. So um on the opposite end. What are some examples of strategies, tools, or technologies that your professors used that that made you that made it harder for you to learn during online learning?

26 41:49

I think the harder to learn in my lectures it came with him not using any tools, but I don't know if you want me to expand more on that or if that's a different question at all, but in terms of tools that made it harder, uh I can't think of anything. It's it's just not using opera- like effective opportunities or tools, it was the lack of.

Moderator 42:10

Okay.

37 42:11

Uh for me, um there weren't any review sessions before exams uh after we changed uh to an online format. So I thought, you know, that that made it tougher for me to study uh for tests because now I had to come up with, uh you know, go search for mat- for material to practice for exams, whereas before the campus shut down, uh you know, the professors used to hand out, uh you know, uh revision material or uh, you know, a practice uh quizzes and stuff in review sessions, which was uh really helpful.

26 42:53

Uh we're sorry uh we're talking about strategies that we don't like, right?

Moderator 42:57

Right

26 42:57

So, things that affected us negatively? Okay, so requiring us to attend lectures at a certain time via zoom was really really annoying and it just it made me not want to not want to pay attention. It made me not want to do well in the classes. This was a demotivator because when it is when you are attending a lecture in person it's much much different from attending it via zoom. So requiring zoom attendance just I don't know why but it just doesn't work. And I--I really dislike that and it definitely was a demotivator for me and I didn't pay attention. I just ended up watching it later. So it was like a waste of time.

Moderator 43:38

Okay. What are some things your professors did during online learning that made you feel like they cared about their students?

26 43:50

Vocal effort and and listening effort, right. So they they were vocal about, "hey, we understand that this is a transition for everybody, just as much as it is for students, not just for professors." So they're expressing that and then they're listening. They're willing to say, hey, you know, email me or what do you guys have to say? What how what would you like to see from us in terms of how do you want to learn and what works better for you? And they try to accommodate from that sense. So I think just effective communication and recognition of the problem at hand and the transition at hand was what made good professors um good during the pandemic.

Moderator 44:30

Okay, so transparency. Uh 37, what about you?

37 44:35

Uh can you repeat please. I'm sorry.

Moderator 44:37

Sure, um, what are some things your professors did during online learning that made you feel like they cared about their students?

37 44:46

Uh we uh every once in a while, some of my professors uh you know, email me uh saying uh, you know uh, if I was behind in any assignment or anything, they would, you know, give us a a reminder uh through emails. Like um, you have you have to submit this in two days or a week or something. So that was there, but I guess that was there before the campus you know shutdown. They would just say it in class. But instead, uh they just say it in class, I think there was a personal you know, email so I, you know, I I it got my attention uh better than you know, uh when the professor used to say it in class. So other than that, I don't think uh there was any other options.

Moderator 45:38

Okay. And 26.

26 45:44

Uh settings, they said, so one one of my professors set up like direct chat lines between all the students so we could just direct message him at any time. And that was, I really like that. It made me feel like you know, I have access to talk to him privately anytime. He is listening. He wants to help. Um that was really helpful. Um emailing students, so I did really badly on one test, my teacher emailed me. And she contacted me, she was like, "hey, what's up? Like, are you doing is everything okay?" I really appreciated that. I would say just in general, the professor taught like the professor demonstrating to you that, hey, I'm here. I want to help. Uh that's, that that helped a lot.

Moderator 46:23

Okay.

26 46:24

I'm a, I'm a add real quick, if you don't mind.

Moderator 46:26

Sure.

41 46:26

Um, you mentioned transparency. Yes, and I think the second part would be consistency. Because I had I did have a few professors who, you know, they showed interest in, you know, well how do you want to learn and what works best for you in the beginning. And it becomes very easy to tell when a profess- when a professor is saying that solely because they themselves don't know what to do and they're just looking for ideas versus they're generally trying to care for you and trying to figure out how to help you learn. I had a professor who was consistent in you know, sending a bi weekly emails or whatnot in terms of hey, whatever's going on in the nation right now, I found an article about this that you guys might be interested in that kind of associates or that relates to our subject or, uh you know, hope you guys are doing well with this this that has come in the news recently. So consistency along with transparency.

Moderator 47:14

Okay. Um and once again on the flip side, what are some things your professors did during online learning that made you feel like they did not care about their students?

41 47:30

I think um one of the things one of my professors did, they would hold, you know, zoom sessions, but they'd be very sporadic. There was no you see, like a lack of communication and you see a lack of respond, responding to emails, and whatnot. So just showing that not directly but maybe indirectl that you've kind of given up on even trying to come to a, you know, a conclusion or a solution to how you want to run the class and just leaving it to us and giving us just due dates, and then not being there. And um I think that was big thing that kind of it demotivates not just you as a student, but you as you know, an individual during this time. Like, okay, if one of my professors is giving up on the class, what why am I still expected to try to participate and fight for a grade?

Moderator 48:20

Mhm. Okay.

37 48:24

Uh so as I said, um I have a massive difference in time zones. So when I, when I asked some of my professors who had live recording sessions, uh like if it was possible for them to, you know, have a recorded session, as well as a live one so if they would just record the session, then just post it online, uh you know, which would make it more convenient for me. Uh they said that was against policy or something. So that was a a bit of...with her so beyond that, I think it was alright.

Moderator 49:01

Okay, so not making proper accommodations for students with different experiences.

37 49:06

Yeah, so the lack of consideration for international students was, I think, a a problem.

Moderator 49:13

Okay.

26 49:17

Uh I would say the same thing because I had a timezone major timezone difference. And so not refusing to post lectures online and requiring live attendance is really not really something that that was unhelpful and caused problems for me and I just felt like they weren't going out of their way. They weren't not going out of their way, they weren't, they didn't want to be helpful, even when they could have is what I felt like was going on.

Moderator 49:45

A lack of accommodation from both of you guys, right?

26 49:48

Yeah, definitely.

Moderator 49:52

Okay, now let's talk about how your university responded to the move to online learning. What are some things that your university did, for example, pass fail options, town halls, remote counseling, to help students be successful during online learning? Mention any resources the university provided or actions they took to help students to be successful or feel supported.

41 50:15

Oh, man-- I think um counseling services were available. They were moved online. Uh weekly updates, which do get very annoying, but um the university sends out like weekly updates in terms of what are they doing as a university um in response to the virus um because REDACTED is the capital of REDACTED, but you know it, they have a--a medical campus less than a mile away that's associated with the university. And so cases coming in from there and whatnot. They were, I wouldn't say they were being truly transparent, but they were giving us information that they wanted to give to try to make us feel supported and comforted through weekly updates, and a lot of universities, um services were moved online. Like I mentioned earlier, counseling or tutoring and whatnot. Now, while they weren't necessarily the easiest to access, or there were a hassle they were there and they were advertised.

Moderator 51:13

Okay.

37 51:14

Uh yeah, there was uh counseling sessions available online uh after those two programs, and other than that, I think uh there was a few surveys or something. Uh but I I never took any of the surveys. So I don't exactly know how this how those worked. So, I think that's all.

Moderator 51:35

Okay.

26 51:39

Yeah, we just got we, we got weekly updates to make sure we know what's going on with in regards to the virus. We got um counseling services available. The university offered the pass fail option, which helped a lot of students out a lot who were affected really pretty badly. Yeah, I would say those are the three main things.

Moderator 51:59

Okay.

37 52:01

If I may add, yeah, we had uh the pass fail option as well. So yeah about that. Yeah.

Moderator 52:07

Okay. What do you wish your university did to better help students be successful during online learning?

26 52:15

Communicate that they don't know what they're doing. I think as a as a university, while it might be easier for an individual to admit that, it is harder for university because of the standards that they want to uphold as a university and for the accreditation. They they really want to make it look like they know what they're doing and that the decisions they're making are the best and in the interest of the entirety of their faculties, students and staff. Whereas realistically speaking, it is virtually impossible for any university to completely and peacefully and to go about the exact right direction and right maneuvers and make the right decisions to go. Universities are going to make mistakes. And I think that if our university was uh more communicative in the sense that, hey, these are options that we're exploring to solve this and this issue. And, you know, or, hey, we took this option, we didn't like it, we're going to retract and try something new, versus trying so hard to sugarcoat your decisions as the best decisions.

Moderator 53:18

Being responsible or taking responsibility for previous actions that might not have been as effective as they hoped it would be.

41 53:25

100%.

Moderator 53:26

All right, cool.

37 53:27

Uh, could you repeat the questions?

Moderator 53:32

Sure. What do you wish your university did to help students be more successful during online learning?

37 53:38

Uh I guess the university had been more considerate about, you know, the student population, I mean, the international population because I have a lot of international friends as well. And me being an international student, I felt like the university did not uh, you know, consider us as much and um we I think there's about uh more than 3,000 uh international students in our campus and I think with a university such uh with a huge number of international students should have been more considerate about the international student uh possibility for, uh you know, they they talk a great deal about, you know, with a lot of culture in the campus, but I think when when I think during this odd times, I think that that lack of consideration was really seen by us.

Moderator 54:27

Okay, and what are some ways you wish your university showed that they were more concerned about the well being of international students?

37 54:36

Um other than the pass fail option, which helped some of uh the students you know, uh to get a a more uh better uh report card, I don't think uh they did, as much as I you know wanted them to or uh what I hoped them to. And yeah as I said, I I tried emailing my professors saying that, you know, the the huge transitions and is there anything we can do? And they would say they said, you know uh, as of now, this the campus has this policy and we can't, you know, overrule. I mean, I don't blame the professors I think it was the management because uh we uh the university didn't have a president during the time of the lockdown or when the campus shut, and they had to come up with a interim president. And I think that made a lot of process much slower.

Moderator 55:35

Okay.

26 55:36

Uh I would say the university should have just as 41 just admitted that they were this uncharted territory. You don't know how to tackle this. They should have taken more student input. Uh I know 37 mentioned that they're sending out surveys, I don't think we even got surveys. So some kind of weekly survey, just polling of, in general questions of how how they're doing would have helped a lot. In general, just taking in student input and gauging how students feel about this, or this decision or that decision uh would have helped out a lot.

Moderator 56:21

Okay. Um now in general, are there any ways that your ability to be successful in school was affected by the pandemic that we have not discussed yet?

26 56:36

I think just additional material, as we mentioned earlier. Just ability, it was a lot harder to you know, get in that mindset of, I need to study for this, this, this and I need to digest and actually learn this material long term.

Moderator 56:52

Okay.

37 56:53

Uh same answer as 41.

26 57:01

Yeah, it was the same thing for me.

Moderator 57:05

Okay, well if there's nothing else to add, that is the end of my questions. Thank you for participating in this focus group. Don't forget to respond to that last email about the name that you're using currently. Um, and have a great day everyone.

**Low SES Hispanic Women**

Moderator 16:51

Yeah, no worries. Okay, looks like we have everyone today so thanks for joining us. Um welcome to the focus group. My name is Moderator and I'm a research coordinator from Redacted. Um and Note-taker is our note taker today. I know it says Redatced but it's Redacted. So a little bit of um discussion of rules. We'll be asking a number of open ended questions. There are no wrong answers but people may have different points of view. Please feel free to share your point of view, even if it differs from what others have said. Keep in mind that we're just as interested in negative comments as positive comments. We want everyone to feel comfortable participating and encourage everyone to talk. Because we have to keep this an hour I may need to cut you off so I apologize in advance. Um we want to get the full breadth of experiences and opinions but some of you may have very similar experiences and opinions. If your answer's the same as another person you may simply say my name, or my answer is the same as Sam's or whoever's. It is difficult for us to take notes on everything that is said in the group. So we'll be we'll also be recording the session so we may transcribe it later. We won't associate your name with anything that you say in the focus group. You may refuse to answer any question or withdraw from the group at any time. And we understand how important it is that this information is kept private and confidential. We asked, we asked participants to respect each other's confidentiality and not repeat things said within the group to anyone outside of the group. Are there any questions before we get started.

Unknown Speaker 18:30

No. No.

Moderator 18:35

Um so, please say, or sorry, first start by going around and each person say what year of college you just finished and one word to describe what life has been like during the pandemic.

34 18:48

Um I go to Redacted College, and I guess stressful.

Moderator 18:54

Stressful. And uh how do you pronounce your name?

34 18:58

34.

Moderator 18:59

34.

34 19:00

Yeah.

Moderator 19:03

Uh 43 how about you?

43 19:05

I got to Redacted and it's honestly been kinda boring since the pandemic started.

Moderator 19:11

Ok, boring. Uh 54.

54 19:13

Uh I go to Redacted. Um, I think it's been actually kind of calming, you know it's it's a different pace.

Moderator 19:23

Ok calming. And 49.

49 19:29

I would describe this semester as um I think I have to agree with 34 like stressful would be the perfect word to describe it.

Moderator 19:41

Okay stressful.

So before we get started I'm just gonna go through like an overview of the project. So the COVID-19 pandemic has presented big challenges for undergraduate students. As you know, universities across the nation have closed, moving all instruction online with very little time for students or faculty to prepare. Many students have encountered challenges related to the closing of campuses, relocation, and the movement to online instruction. In this study we want to learn how the closing of campus and the pandemic in general has affected you. We want your opinions about the good and the bad on how things went as well as what your professors and universities did that was helpful and unhelpful and what you wish they would have done. So we're going to start by discussing where you went when your campus shut down. I'd like to hear from each of you. So my first question is, um if you could just tell me about the space in the home you moved to. What space did you have to do your schoolwork there and if you could comment on whether the space was private or shared, and who you shared it with?

34 20:47

Um, for me, it was my room, and it was most of the time it was quiet, but I shared it with my sister, which is in like elementary school or middle school.

Moderator 21:01

And so was she also doing work at the same time you were in the room?

34 21:04

Yes.

Moderator 21:06

Okay. And you said for the most part it was quiet.

34 21:09

Yeah.

Moderator 21:10

Okay. And were you um prior to the shutdown were you um commuting or did you live on campus?

34 21:19

I was commuting.

Moderator 21:21

Okay, thank you. 43, how about you?

43 21:25

I live in student apartments and I had my own private room.

Moderator 21:28

Uh so you lived in an apartment during the shutdown and is that the same as before the shutdown?

43 21:34

Yes it's a student apartment. So it's close to school and with other students.

Moderator 21:40

Okay. How about you, 54?

54 21:44

Uh, I was staying in a dorm. Um and then when I came back home I have my own private room and everything, but there is it is a lot noisier here in a sense like I have pets and I have like distractions, so it's a little bit harder in that sense.

Moderator 22:01

Okay. 49.

49 22:04

For me, I commute as well. So, um, but in my case with the pandemic I ended up going to my grandparents house. And uh even though I had a room for myself, it was still like noisy around the neighborhood and stuff so it was difficult to focus.

Moderator 22:24

And sorry you said you had the room to yourself in this house?

49 22:28

Yes.

Moderator 22:29

Okay. So what challenges did you encounter attending classes or doing schoolwork, in some in this space? 34, we can start with you again.

34 22:45

Yeah um for me, I guess it was that it was difficult for me to get help. Because sometimes, like, I like, I went to like uh for in school I went to people that could help me with my assignments. And, but when I when the the pandemic happened, I didn't have that much resources, and also it was kind of hard for me sometimes to reach my professors too. And sometimes it was kind of hard for me to pay attention to, like, what was happening in class.

Moderator 23:18

Okay. Were there specific things about your environment that made it hard to pay attention to what was going on in class?

34 23:25

I guess it was more my sister, cuz sometimes she interrupt me. So it was kind of like hard for me to like pay attention.

Moderator 23:32

Okay. 43, were there specific things about your environment um that made it challenging to attend class or do your school work in that space?

43 23:43

I just couldn't be as productive as before because I used to never do my homework in my room. I used to do it always at school and then and the my room is like a place to relax. I just couldn't focus on my things and my work.

Moderator 23:56

Okay,

43 23:56

I just became much less productive after the lockdown.

Moderator 23:59

Sorry, what was that, the last thing you said?

43 24:01

I became much less productive after the pandemic.

Moderator 24:05

Okay. 54 what kind of challenges did you encounter with working in the space?

54 24:11

Uh uh pretty much like the ones they listed. It was hard to get help, it was difficult to get in contact and learn from the screen. But I think I love my family but they didn't know the difference between um like when I'm in school, and like when I'm just like you know regular so they were definitely distraction there. Yeah.

Moderator 24:32

Okay and 49.

49 24:36

So, as I mentioned the noise in the neighborhood like the neighbors have some um parakeets birds and they would like be very noisy and it was very like stressful, if I have like a class, and if I have to participate. It was very stressful because you could hear that, and it would interfere also when I was listening to my professors.

Moderator 25:04

Okay, so some noise distractions.

What are some things that you or other people in the house that did that made it easier for you to attend classes or do your schoolwork? Uh we can go in the same order, 34.

34 25:20

So what was easier for me to what?

Moderator 25:23

Uh like what kinds of things did you or some of your family members do that made it easier for you to attend classes or do your schoolwork?

34 25:30

Um, I think getting my own space. But sometimes, my sister uh ended her classes so I was able to like tell my mom to get her out of the room so I could finish my schoolwork. And yeah, and also like I used to like close the door so that noises that they made that my parents made were not like heard for me.

Moderator 25:56

Okay, so when your family helped you have a quiet space, it sounds like.

34 26:00

Yes.

Moderator 26:02

43 how about you?

43 26:04

I live with other students so it was also a quiet place and just when they go like go to study and now you think oh I might as well study also.

Moderator 26:13

Okay. 54, what are some things that your family did, or that you did that made it easier for you to study?

54 26:20

Um, I I created a study space in my room. So, you know, I got a desk I like did it and kind of rearranged it how I had it in my dorm. Just to get me like in that same like I guess habitat as I used to be.

Moderator 26:35

Okay. 49.

49 26:39

Um, what was key for me, I think was giving my mother like my schedule of all my classes so that she would know like at this time I'm in class so, like, don't, so nobody would interrupt me. And if I had like a 20 minute break so and I had to like speed to, I don't know get lunch I wouldn't get like sidetracked or anything so yeah that really helped me be on like on top of my classes.

Moderator 27:07

Okay. So now we're going to talk about um other responsibilities that you had after the campus closed and how these differed from responsibilities that you had before campus closed. So please describe your caregiving responsibilities, including child care elder care or caring for ill loved ones, and how they were alike or different from your responsibilities before the shutdown.

34 27:36

Um, I believe, I believe that one of my responsibilities, was to take care of my sister after the uh college was closed. Before I used to not um take care of her that much because I used to be at school all the time. So I think that's one difference. And also work too because I used to work, but once the um once everything shut down, I I have more free time for myself and I wasn't working anymore.

Moderator 28:10

So, would you say that your caregiving responsibilities increased then?

34 28:15

Yes.

Moderator 28:16

Okay, 43.

43 28:20

Um after the lockdown. I was in organizations and so after it I just didn't have to do work for them anymore, so I guess I had less responsibilities.

Moderator 28:29

And you said you were in the

43 28:31

In organizations

Moderator 28:32

And but in a living in an apartment so were you didn't have any caregiving responsibilities or anything like that?

43 28:38

No I didn't.

Moderator 28:39

Okay. 54.

54 28:42

Um, it it was about the same. I just had more more chores I guess to do so that like took away time too, but I mean I it was still pretty much just myself I had to take care of, but yeah.

Moderator 29:00

Okay. And 49.

49 29:03

For me it was the same as 54. I had pretty much the same responsibilities I had before everything shut down.

Moderator 29:12

Okay and kind of similar to that if you could describe any other household responsibilities you had such as chores, cooking. caring for pets during this time, and how they were alike or different um before school closed.

34 29:30

Um, my chores before um everything shut down was most like um cleaning the plates. But like after like everything got shut down it was more like cleaning the whole house and the plates, I mean the plates and cooking too. So they increased

Moderator 29:49

Okay.

43 29:52

Mine stayed the same.

Moderator 29:54

They stayed the same. Okay.

43 29:55

Yeah.

Moderator 29:58

54.

54 29:59

Ah, well in the change I went from like you know eating Chick-fil-A like every other day to like actually like being home. And so, I had to like to actually clean and stuff like that. And it was just um I I also have a bird conure in my room, and so she's really loud so that was a big change, and she like requires a lot of attention. Yeah.

Moderator 30:21

And when you say you um instead of getting say like Chick-fil-A, you were cooking. Were you cooking for yourself or did you were you also responsible for cooking for other members of your house.

54 30:31

You see, I'm not great at cooking so my mom was the cook um but like I still had to cleanup afterwards so yeah there was like all these like time consuming things that took away from school.

Moderator 30:42

Okay and what kind of other besides like cooking what kind of other time consuming things were there?

54 30:47

Oh cleaning cleaning a lot of cleaning. Yeah, um I have a nephew here that's that's living with us and so you know there's a lot like, you know, it just has to be the cleanest. Oh you know yeah yeah (laughs).

Moderator 31:02

Okay. 49.

49 31:05

Okay, so in my house I'm basically responsible for like sweeping the house, and the rest of the chores we basically divided amongst ourselves like cleaning the dishes and stuff. But for me, I think it's pretty solid like I'm usually the one in charge of like sweeping the whole house, and like washing the bathrooms and stuff.

Moderator 31:26

Did you have these responsibilities before the the pandemic?

49 31:32

No, they were about the same. I think I had less less actually, when, after everything shut down since I wasn't in my house. So since it wasn't my own house, I was just in charge of like cleaning my, my room.

Moderator 31:48

Okay, so a slight decrease then?

34 31:52

Yes.

Moderator 31:53

Okay. So to what extent were you expected to take on more household work such as um or childcare or family caregiving responsibilities than other people in your home. So for example, parents may expect more of one sibling or partners or roommates might not contribute equally.

34 32:17

Can you say that again, the last part?

Moderator 32:19

Sure. So like um if you could tell me to what extent you were expected to take on more household work or like childcare compared compared to other people in the house. So like for example parents might expect more of one sibling or your roommates might not contribute equally.

34 32:41

Um, I believe that when it comes to like cleaning, it was kind of like unequal, because mostly cleaning was done by my parents and me and my sister clean like sometimes. And when and all those chores like cooking and stuff, I feel like it was more my parents did more that more that than I did.

Moderator 33:06

And then could you explain why you, you said that uh you felt that you and your parents did more than your sibling.

34 33:12

Um, because like most of the time that the people that were like cleaning the house, or or the plates, it was either me or my parents. And every time like they told my sister to do it, she just like ignore them, and they just did it. She didn't want to do it.

Moderator 33:29

I see. Okay, 43.

43 33:34

Um in my apartment we will just all clean after ourselves, and just do our own stuff. So, there were no like conflicts or anything.

Moderator 33:43

Okay. 54.

54 33:45

I mean, in in my other dorm, I, well in my dorm we all have a private room, and so it was like, I cleaned up after myself and that was that. Like over here I guess there was a change. Um yeah, just having to clean more I guess, yeah.

Moderator 34:03

And with the cleaning do you think that you had the same amount of responsibility as other people living in your home?

54 34:10

Uh I think it was a little bit more because my parents, you know since they're at work you know I you know I want the house to be clean by the time they get home. And so I'd like I mean it's kind of like unspoken things that I do but it's just something I do to be nice, help out.

Moderator 34:28

And I forget, did you say you had siblings?

54 34:31

Yeah. Mhm.

Moderator 34:33

And did.

54 34:33

I haven't.

Moderator 34:35

I'm sorry. Go ahead.

54 34:37

Oh, uh I do have an older sister um but as I I said she is with my nephew and so she's like taking care of him mostly. And so like I I don't really expect her like you know to be cleaning and stuff like that and then my little brother I mean, he's just, you know, playing video games all day. So, yeah.

Moderator 34:56

Okay, and 49.

49 34:59

I think that in my case, um my grandmother was the one who took on most responsibility in cleaning and stuff in like the house, because she has like her own way to that she likes to do things around her house so she did most of the cleaning in like the living areas and stuff. Um but in other chores like um washing the dishes, that that was basically the same between split between the adults of the house including me. But the rooms, each one, clean their own room.

Moderator 35:42

Okay. Now we're going to talk about how your academics were impacted by the shutdown. I'm going to ask you how the shutdown impacted a variety of things and if you don't think it has impacted something, you can just say no impact. So in what ways if any has a shutdown impacted your academic performance such as grades and your understanding of the material of your courses?

34 36:11

Um, I will say that, like, when it comes to the things that I learn, I didn't learn that much, but my grade kind of like went up because most of the stuff that look cuz most cuz we were not doing as much as we were supposed to be doing. So that's why she just gave us like my, my professor gave was like, easy stuff to do so that's why my grade like went up. But my like knowledge of what I learned it was it decreased.

Moderator 36:42

So you think if if it weren't for the shutdown you would have been given more challenging material or?

34 36:48

Yeah.

Moderator 36:50

And could you explain uh why you think your professor gave you some easier.

34 36:55

Um, I guess it was because of the pandemic cuz most people were going through some stuff, and they didn't want to like pressure us, or make us feel like like stressed because they didn't know like if everybody was going through the same um situation.

Moderator 37:16

Okay. 43.

43 37:19

Um I think we learned less than we would have because it took a while for the teachers and the students to adjust so they went much slower and they did skip over some stuff that we were planning to see, and they like um they did make stuff easier for for us because they knew we were stressed.

Moderator 37:37

Were your grades impacted?

43 37:40

Not really.

Moderator 37:43

54.

54 37:45

Uh it my grades did improve. Um but at at the same, I I feel as if I didn't retain the information. Uh like like I learned it and like I understood it well enough, like to like, you know, get a good grade but I I don't remember much of it now.

Moderator 38:05

What do you think contributed to maybe not retaining it as well?

54 38:10

Well, I think for me it's because I I like going to study groups and you know we'd work out problems and so you talk it out, and stuff like that. And then whenever I was here, I just, you know, studied for me. And then so I was like okay okay this is it goes, so like let's say like I'm doing calculus or something it's like 123 and like I know how to do in that form, but I'm doing it to like teaching it to somebody, I explain it out. And so it's easier to teach somebody, and then in that way retain it better. I I don't know if that's confusing I'm sorry. Um but it was just, it was just um easier but at the same time I don't know I don't remember much yeah.

Moderator 38:52

Ok that makes sense. 49.

49 38:57

So my grades were pretty much about the same as it it was present. So, but it was actually a lot harder to, it took more effort to maintain that grade.

Moderator 39:16

Uh what caused the what made it like have more effort to maintain the grade?

49 39:22

Well, since everyone was adjusting to having everything online, some professors were understanding and. But others, particularly tried to give the class and the material as if it was not online, as if it was a normal semester, and it wasn't. They gave the same amount of work, but the class weren't the same as before. I don't know if makes sense, but yeah.

Moderator 39:58

And did you feel like your understanding of the material, like so you said your grades were about the same but did your understanding change, even though it was more effort?

49 40:07

Um, I'd say it stayed. I would have understood the same, if it would have been not online. So.

Moderator 40:20

Okay. So, in what ways if any has the shutdown impacted your career goals?

34 40:31

Um, so I guess like cuz since, I was taking um a lab class, but because of the shutdown, we couldn't do that much hands on. So I feel like that's one way that impacted my curriculum.

Moderator 40:51

And how did that affect your career goal?

34 40:55

So I guess like mostly like instead of doing like um hands on we had these like PowerPoints. And also, another thing was the exams. Like some classes didn't want to give us exams exams or tests, because they thought that we were gonna cheat. So it was kind of like difficult for us to like, bring our grades, like, increase like our grades up.

Moderator 41:24

So you had a shift in like what you, what kind of career you want from this.?

34 41:28

Oh, you mean curriculum or career?

Moderator 41:30

Sorry, career.

34 41:32

Oh career? Oh no no no no.

Moderator 41:35

Okay. 43.

43 41:39

I was hoping to get experience during the summer with like an internship or research but most of them got canceled so I'm just doing classes instead.

Moderator 41:48

Did this uh change the kind of career you want to pursue?

43 41:52

No.

Moderator 41:55

54.

54 41:56

Uh for me I'm still consistent with what I want to do. Um I actually feel as if it gave me a bit more time to like really focus on what I want and like look into it more. Yeah, yeah.

Moderator 42:09

Okay. And 49.

49 42:11

I feel like for me, it didn't have an impact. My career goals are still the same.

Moderator 42:23

So in what ways if any has the shutdown impacted the availability of other learning opportunities such as internships?

34 42:33

For me it didn't have an impact because I wasn't thinking about getting an internship this um semester.

Moderator 42:41

Okay. 43.

43 42:45

Um, the ones that I applied for got cancelled so I couldn't do any.

54 42:54

I I felt as if it didn't really apply to me. Well, I I feel as if I didn't get affected because same I I wasn't gonna do do an internship this this semester.

Moderator 43:05

Okay so no impact. And 49.

49 43:10

Um I wasn't specifically like. I I looked into an internship, but I feel like, um, as a first year student, as a freshman, uh it's hard to find, like on your own, even though you do your research. So sometimes it's, um, it's good when upperclassmen from your same career or professors, they give you that like recomendation or like, um or they bring people to give um orientations etc. So, not having that, because everyone's like paying attention to adjusting to the new online class thing, then it's harder. So yes, I feel like it did have an impact.

Moderator 44:04

Were there any new learning or career development opportunities that emerged during the shutdown and if so what were they?

34 44:13

Um, so I guess like I learn like how to code after the shutdown because I have more like free time.

Moderator 44:21

Okay.

43 44:25

Can you repeat the question?

Moderator 44:26

Sure. Were there any new learning or career development opportunities that emerged during the shutdown?

43 44:35

Okay, uh no, not really.

Moderator 44:37

Okay.

54 44:39

Uh yeah, same for me. Not really.

Moderator 44:47

49.

49 44:50

I agree with them. In my case, no impact.

Moderator 44:58

So in what ways if any has the shutdown impacted uh your confidence to successfully complete your bachelor's degree at all or in a timely fashion?

34 45:09

Um for me I guess it didn't have any impact.

Moderator 45:14

Okay.

43 45:17

I guess since I knew there was nothing else to do, I just decided to take classes during the summer, and so now I feel more confident that I won't be overwhelmed about the hours during the regular semesters because I was going to take 18 next semester but now I only have to take like 14. So, I guess I'm more confident right now.

Moderator 45:34

Okay. 54.

54 45:36

Yeah, same for me. Like, I mean since there isn't much more to do I'm just taking calc classes right now so. I feel good going into this fall semester.

Moderator 45:47

So would you say you feel more confident or about the same?

54 45:51

Uh I more confident I guess. Yeah more prepared.

Moderator 45:59

And 49.

49 46:01

I feel like I'm less confident now. Before I used to have um like a plan of what classes I was going to take and the time it was going to take. But now since everything is moving towards online I'm like, doubting and worrying about how this semester online will be and if I can truly like follow through with my previous plan.

Moderator 46:30

Is this because you're worried about say like your academic performance or classes that are available?

49 46:37

Both.

Moderator 46:40

So is there are there less classes available if it's online?

49 46:46

It would make sense if it was more class available but for me I feel like I had a harder time finding classes online.

Moderator 46:57

And is this because uh your university's offering less?

49 47:03

I actually haven't looked into it that much but I feel like I've seen less sections, but it also may be due to this, because they reserve certain classes for the upcoming new students, new freshmen. So it could be because of that as well, that they have less, um less courses that I actually need to take.

Moderator 47:33

And in what ways if any did the campus shut down impact your finances?

34 47:41

Um, like like I said before I lost my job so right now I'm not working. Also, uh it was kind of hard for me to like contact financial aid for the next semester.

Moderator 47:57

Okay, so a negative impact. 43.

43 48:01

Um in the spring, it didn't have any impact but during the summer they didn't charge for like the, the gym fee or anything. They helped me so yes it was cheaper than we would have otherwise.

Moderator 48:15

Okay.

54 48:17

Um for me, since, like, I got like all my money back for like the housing and stuff like that that helped. And then um since I was at home and I you know I didn't want to be at home all the time I got a job. And so I guess it's good. Um and it's good in the sense um if I'm not going to be dorming there during the fall semester so I'm saving money that way as well. So it's it's good in that sense.

Moderator 48:47

Okay. 49.

49 48:51

I'd say it had a negative impact because since you when you're stuck at home um I think it, that really deterred from, like, my mom's work. Like she doesn't really work at a set company or anything. It's just like independent work, so it's hard for her to like go out and also like exposing oneself and their health. So, and also as, I don't know who was it that mentioned, difficult to contact financial aid through the university so that as well.

Moderator 49:30

Okay, so negative impact. Now we're going to talk about what your professors did and did not do during the move to online learning. So what are some things, so examples of strategies, tools, or technologies that your professors used that you found to be ineffective. Sorry, but effective so they made it easier for you to learn, or ineffective so they made it harder for you to learn during this time?

34 49:58

Um, so, for me like an effective way that it was that they had like they recorded the sections, so I could, I could go back and watch what was said. But for some of my professors, they kind of like they didn't meet like regularly, so it was kind of hard for me to like communicate with them and, and I was only, I was only able to like talk to them through email, which was like really hard.

Moderator 50:31

So. Uh, I just have one more question for 34. So in the emails, you said they weren't um responding quickly?

34 50:41

Yes, cuz sometimes I had like group assignments or project so it was kind of like hard for me to like, um send them an email and then like getting a response back so yeah.

Moderator 50:56

Sorry 43. What are some things that your professors did that were both effective, so they made it easier to learn, or ineffective that made it harder?

43 51:03

Uh I also had professors who recorded the classes so we can just watch them whenever in at our own pace. And I had some teachers who like knew that we were, we will be confused and they will be faster at responding emails than before, but there are others who were not good with technology so they were harder to contact.

Moderator 51:24

Okay.

54 51:26

Yeah, for me as well. It helped that they posted like the notes and stuff like that so it was easier to access and then that the videos were there. Like teachers who normally wouldn't upload like you know the recorded lecture actually posted them so it was like nice for that. Um but yeah sometimes when you send an email it it they they respond like really vague so that that wasn't great.

Moderator 51:52

You said vague?

54 51:53

Yeah, it'd just be like, or they'd answer the wrong question and so sometimes you don't want to like send like multiple emails so it's just I guess, communication, got a little bit harder. Yeah.

Moderator 52:06

Were you afriad that if you sent another email, you'd get like a negative response or something or?

54 52:11

Yeah I didn't want to be annoying. So, you know, and so I just had to figure out myself.

Moderator 52:18

Okay. 49.

49 52:22

One effective strategy that I liked was my mathematics professor, she did videos like she screen recorded herself um doing exercises and like examples as she explained and introduced the material. And she recorded them on YouTube, so that we could watch at any time and then we'd meet at our class hours and then we talk about the the topics, which was very helpful because for math you know, if you're getting introduced to a new material, you want to really see it so that you can grasp grasp the concept and that was really helpful. And an ineffective method I saw was giving, like, three projects at a time for like one same class. Because that overwhelms the students, and you really don't know where to start sometimes.

Moderator 53:22

Were all these projects given, would would they have been given if it weren't for the pandemic?

49 53:29

I believe so that they will be more spread out, but because we were kind of running short of time, they still decided to give the same amount of work, but since we didn't have any much time well they have to like give closer due dates, so you'd have like three things for like the same weekend for the same class so it was very stressful, but if it were a normal semester, everything could have been completed, you know at a decent time.

Moderator 54:00

I see. Okay. So what are some things that your professors did during online learning that made it feel like they cared about their students, or that they didn't care about their students?

34 54:14

Um, I think one way was that that they were like really understanding when it comes to deadlines. Like you could like turn it in after the deadline without them taking points off. And I think one thing that I felt like it wasn't that nice was the email thing. Because, like, I don't know who said it, sometimes they kind of like respond, but they didn't actually answer your question.

Moderator 54:43

Okay. 43.

43 54:47

Um, I have professors who everyone of my professors canceled like either a test or homework so we won't have to worry about it. And so yes they made it easier.

Moderator 54:58

Were there any things that your professors did on the other hand that made you feel like they didn't care about their students?

43 55:06

Not that I can think of right now.

Moderator 55:08

Okay. 54.

54 55:11

Um for me, I I think my professors were really helpful in that sense. Um like for chemistry um they, they changed the whole way of grading. So instead of like, we had two exams left for like the rest of the semester, but instead they split it up into instead of taking exams taking like weekly quizzes that were worth like 10% of our grade. So it actually, like, helped us like in the long term. And they stopped labs so everybody's lab grade was like guaranteed at least an 80 or above which is good you know normally for lab grades. And um yeah and then I had a a project that was supposed to be, like, like worth like almost the same as our final grade um, and, and she my, my, my professor she cut the work down, just you know, because they were understanding during this time.

Moderator 56:05

Right. Were there any things that your professors did that made you feel like they did not care?

54 56:14

Um, just, just that sometimes being difficult to like, you know, communicate with but like besides that I think they were it was pretty much a positive.

Moderator 56:29

Okay, and 49.

49 56:32

Most professors showed that they cared, in my opinion, just by being available at their office hours and like actually being there because I know professors that probably were like, not really available and not responsive and giving you feedback on on your doubts. On the other hand, I had a professor that showed that they didn't really care about us, because on a test, we, all of us failed terribly. And everyone was complaining and writing her emails like hey, these, these problems could have had better um written. They could have been written better, so we could understand and, you know, they could have been more explanatory. And the way she replied was basically, if I would have explained you all wouldn't have gotten it anyways, so she didn't even make the effort.

Moderator 57:32

Okay. Uh now we're going to talk about how your university responded to the move to online learning. So what are some things that your university did to help students be successful during online learning? You can mention any resources the university provided or actions they took to help students be successful or feel supported. So some examples are pass fail options, town halls, or remote counseling.

34 58:04

Um so my school they did remote counseling and they also did the pass and fail thing so yeah.

Moderator 58:16

Okay.

43 58:17

My school did the pass and fail thing, and it wasn't at first but after a while they added a a resource that we can just email any questions, any subject. Like they had some tutoring, but it's through email, and they didn't have that before.

Moderator 58:30

So online tutoring services you said.

43 58:33

Yeah, and like at any time. You don't have to like make an appointment or anything. It's just like just ask the question and get answers and that wasn't there before.

Moderator 58:42

Okay.

54 58:44

Uh for mine, yeah, they had pass fail as well and online tutoring available.

Moderator 58:53

49.

49 58:55

In my university, they also implemented the pass and fail system.

Moderator 59:02

Okay. What do you wish your university did to better help students um be successful during online learning?

34 59:13

Um, that's a hard question. Um I guess, like when it comes to like tutoring, like it was kind of hard for me to like find people that will actually help me, and I'll and when it comes to like my academics, but also I think it was also like financial aid. It was kind of like hard for me to like communicate with them and talk to them because of like how many how much, how much people, and some offices, also were like, closed all the time so communication was just non existent. So I guess like improved communication between the university and students.

Moderator 59:55

Okay and then you also said um better tutoring. Is that right?

34 59:59

Yeah.

Moderator 1:00:04

43.

43 1:00:05

Uh I think they it would have been better if they they used to have like a system at the library where we could just like ask for a book online or they can scan whichever, even if they use from a specific book but they stop that when the pandemic started. And it would have been better if they had kept it going.

Moderator 1:00:25

Okay so continued access, you said to library resources.

43 1:00:28

Yeah.

Moderator 1:00:30

Okay. 54.

54 1:00:35

I'm, um, that they could have done better. Um nothing. I can't nothing comes to mind besides like I know like international students had to like leave and then that was it. Like you know like it was spring break and like they just had to go back home. I I feel as if that could have possibly been handled better. Um but um besides that nothing really comes to mind.

Okay and 49.

49 1:01:02

I think I agree with 34. I think the central offices of the universities could have been more organized like financial aid, especially in this time where people are worried about money and have lots of questions about this semester and the next one. They were really unavailable to everyone and took like months to reply. So that was very hard.

Moderator 1:01:27

Ok so again, you wish they had better communication.

49 1:01:30

Yes.

Moderator 1:01:34

And then my last question is, in general, are there any ways that your ability to be successful in school was affected by the pandemic in a way that we haven't discussed yet?

34 1:01:46

I guess like I wasn't able to like talk to other professionals or network, because like most like all the organization and clubs that I was part in, I couldn't like go there anymore so I guess that's like one part that I feel like got affected.

Moderator 1:02:06

Okay.

43 1:02:08

Yeah, I think I have the same answer. And also, advisors were much more less available during this whole thing. They'd take like weeks to answer an email. And it just --it sucks when we're trying to register for classes and everything.

Moderator 1:02:24

Okay. 54.

54 1:02:27

Uh I think for me, it had both a positive and negative impact. Um the, the negative. Wait okay I lost my track of thought oh my god. Um oh okay okay I forgot. Okay okay I got I got it. Um yeah, I didn't go to clubs anymore and like I feel as if I was like barely because I my okay so it's my it was my first year. So my fall semester I had trouble like you know getting out of my circle and then the second semester I was like finally socializing. And so, and then it just got shut down so I feel as if I it lim- limited me in that sense of networking, but it was positive in the sense, it let me like, you know, be able to manage my stress be able to like focus on me and focus on what exactly I want in the long term.

Moderator 1:03:21

And what exactly what helped you focus in this sense?

54 1:03:24

I think, not having as many distractions. So I mean like during that time, you know like, like what Spring Break was like two weeks but you couldn't go anywhere. So like, you know, just having like that time at home like for with nothing to do gave you like a chance to like better yourself I guess.

Moderator 1:03:48

Okay. 49.

49 1:03:51

I think that um all this shutdown it caused me worries in terms of being home, and, for example, electricity going out or something and, you know, depending on the computer obviously to take class and such. Um we don't really own a generator so if electricity were to go out or like signal in my area um then it would affect me negatively. So it was like a constant worry, whereas when you're at campus, if the light goes where the light goes for everybody but when you're on your own right now

Moderator 1:04:32

Okay so more stress with uh in relation to like technology issues.

49 1:04:37

Yes.

Moderator 1:04:42

So that's all the questions I have today. Uh thank you so much for coming and being willing to share. Um if you could just send us an email with the name you used during this focus group and once we receive that and confirmation that you completed the survey we'll be able to send you your gift card. Thank you again.

Thank you. Thank you.

49 1:05:08

Thanks to you, You

54 1:05:12

stay safe out there. Okay, bye.

**High SES Hispanic Women**

Moderator 10:19

Hi everyone, we can go ahead and get started. If you feel comfortable, you can go ahead and turn on your camera. Okay. So, welcome to the focus group. My name is Moderator, a research coordinator at [Redacted] and today our note taker is going to be Moderator. Um, Moderator, if you can just say hi, you don't have to turn on your camera if you don't feel comfortable.

Uh hi everyone, thanks for coming today.

Okay, so today we're going to be asking a number of open ended questions. Um there's no wrong answers but people may have differing points of view today, so please feel free to share your point of view, even if it differs from something others have said. On the other hand, you may have really similar experiences to someone else and if your answer is the same as someone else, you can just say, my answer is the same as Sam's. So today we're interested in negative comments as well as positive comments. We want everyone to feel comfortable participating and we encourage everyone to talk today. So, we have to keep this to an hour so I may need to cut you off, so I apologize in advance. If I need to cut you off, um I'm just going to raise my hand like this so that way you know we need to move on to another person. It's going to be difficult for us to take notes today on everything that's said in the group. So we're also going to be recording this session so that we may transcribe it later. We're not going to associate anything that you say today with your name. In addition, you can refuse to answer any questions or withdraw from the group at any time. Um we want to respect other people's confidentiality so we ask that everyone not repeat anything that they've heard in the group today outside of the group. Okay. So are there any questions. All right, let's go ahead and get started. So first we're going to go around and say each person's name, and what year you are in college, and one word to describe what life has been like in the pandemic. So I see 63. So if you could just um let us know what year you are in college and one word to describe the pandemic to get started. So what has the pandemic been like for you?

63 12:35

So I'm a sophomore at Redacted, and a word that I would use to describe the pandemic would be stressful and crazy.

Moderator 12:48

Okay, and 51.

51 12:52

Um I'm also a sophomore and then I would describe the quarantine thing as exhausted.

Moderator 13:01

Okay, awesome so let's go ahead and we're going to get to the body of our focus group questions. So the COVID-19 pandemic has presented some big challenges for undergraduate students. As you know, nations across I mean universities across the nation have closed moving all instruction online with very little time for students or faculty to prepare. So many students have encountered challenges related to campus campuses, relocation, and just movement to entirely online instruction. So in this study we want to know how the closing of campus affected you. And how things went, whether they went well or bad, what you wish your professors would have done, what you wish your universities would have done, and things you found helpful or unhelpful. So we're going to start the discussion today by discussing where you went when your campus shut down. So, um 63, so tell me about the space in the home that you moved to. Um so where were you during the pandemic and where you went to go do your schoolwork um in that space, and whether the space was private or shared.

63 14:11

So originally, um, when the pandemic started I was actually on spring break vacation and I wasn't home. So I flew back home and I've been here ever since. Thankfully, um our home provides me a space where I can study, where I have a desk, where I have my privacy, and no one really bothers me. But I know that other people really don't have that um, you know, they're not as fortunate to have thi- this space that I have to study.

Moderator 14:38

Okay. So, is it where is your space in your home? Is it a room, or?

63 14:45

So, um my, my home is three, three floors three stories. Um and the third story is basically like an office area, study area um where we have desks. We have boards. It it was actually like a study area that we used all throughout high school and middle school.

Moderator 15:03

Okay. And 51. So where did you move to and where were are you doing your work in the place that you moved to?

51 15:11

So 63 is actually my twin sister.

Moderator 15:13

Ohh.

51 15:14

I just couldn't figure out how to change my name, because that's my cousin's name. Um, well we were in spring break like she said, and then we came back home. And at first it was like crazy because every every nobody knew what was going on, or what was going to happen. If we were going to start again and everything like that. But everything after that it went smoothly, at least, so yeah.

Moderator 15:43

Okay, so you both are twins.

51 15:47

Yes.

Moderator 15:48

Okay. All right. Um, okay. So, what challenges did you encounter attending class or doing your schoolwork, in this space? Um we can start with 63 again.

63 16:02

Um, so I would say that one of the main like the hardest things was that um like finding a study buddy for your class. At least I have my sister so like we took basically the same classes, but like um to study with other friends, get help on homeworks, go to um tutoring and things like that, it everything just became online, and I, to a certain point felt that I wasn't learning as much as I wanted to because classes were online. Um plus I really didn't have like the benefit of having the professor there, who I could ask questions, who I can reach out to at any time, cuz the professors were also busy dealing with the pandemic and their families so it it was all a little bit stressful because you couldn't get ahold of them as quickly as you wanted to and classes were online so it wasn't the same and we were were just sitting here all day um in our third story in our desk. So it became pretty sedentary I would say.

Moderator 17:00

Okay, and 51, did you experience similar challenges in this space.

51 17:07

I would say yes, but I think I had most problem, building a routine, like waking up every morning cuz like when we were in the campus we were in the dorms so we had like that vibe to get to get up and like start walking to class. But here it was just like the same routine every single day, like, get having to wake up at 8am which was awful. And then have to having to go upstairs. It was like, I didn't have like a motivation, basically, just to go to class, so it was that that for me was the hardest part.

Moderator 17:42

Okay. Um, could you tell me a little bit more about your motivation? So, what was making it really difficult to stay motivated?

51 17:52

So I would say like, for example you're you're in a over there in the campus you're in a classroom with all the people. Everybody's talking about what's next, what what are they going to do in the weekend. And here, you're just all alone, you know. So you don't have, like, the same feeling of getting up every single day to go to a class you're going to be alone in. So I guess that's what I would describe it as.

Moderator 18:19

All right, thank you. Um, so what are things that people in your house did that made it easier for you to attend class, or do your schoolwork? So, we'll start with 63 again.

63 18:33

So sometimes like um our fam, like our parents they wouldn't come up here during the day, or if they did, they would just be quiet like to keep us company. Um or if we were studying someplace else in the house they would try to keep it quiet. Um, but yeah that's basically what they did cuz we're we study independently and everything so.

Moderator 18:52

Were you and 51 in the same room, or did you have like separate rooms on your third story?

63 18:59

No, it's the same room.

Moderator 19:00

Okay. Um, so 51, what are some things that people or that you felt in your home, were easier for you to attend class?

51 19:10

Um once in a while our mom would bring us food. And I guess that really helped. Because I didn't have to work with a empty stomach.

Moderator 19:20

Okay, so let's talk about other responsibilities you may have had after your campus closed and how they different differed from responsibilities you had before your campus closed. So, what kind of responsibilities did you have, like including child care, or elder care, or caring for ill loved ones and how these were alike or different from your responsibilities before the shutdown? So 63, could you just describe any caregiving responsibilities that you had and whether that changed?

63 19:50

Yeah, so, um, during college like when we're away at college like our pets are over here at home, and when we come back at home um you know they're our pets so we have to like I have to take care of my dogs. Um so that was like one respons- additional responsibility like getting up, giving them food, taking them out. Um we would also have to like um be aware that our grandmother is 83 years old, so we we really had to be careful every time we went out to the supermarket, take showers when we got back home, disinfected everything, cuz, um, as we all know the coronavirus is pretty um fatal towards elderly people.

Moderator 20:33

Yeah. And so your grandma lives with you?

63 20:38

Yeah, she has her own house but um she's been, I think she's like staying now for like, just so my mom can take care of her, and she can't really be alone during this time.

Moderator 20:50

Okay. And 51, um could you just describe a little bit about, you know, similar question like any responsibilities you had and how they changed?

51 21:01

I guess other than the pets, over there we cared for each other, my sister and I. So, over there we were like always, like, keeping each other in like in contact and everything like that, but over here that wasn't necessary. So I guess that really didn't continue.

Moderator 21:21

Okay, so you all touched base on this a little bit when you talked about caring for pets, but were there other household responsibilities that you had during this time and how were they were alike or different from before school closed. So 63.

63 21:37

So, um, at when we're away at college, we normally clean like our dorm once a week so that's like the same thing over here. We help clean the house, but obviously it's more cleaning so it takes more time, cuz we have to clean the whole house with our mom cuz it when we're away it's just three people in the house but when we come back, it's just, it's five so it's more mess, um more things to keep track of. So I would say that definitely like changed like having to clean more, help more in the house. Like over there it was just like me and my sister so it was just like our stuff like cleaning our dorm, getting our stuff. Yeah. Basically it was I would say that was what changed.

Moderator 22:17

Okay. And 51, what about you?

51 22:21

I guess my opinion towards that changes from hers. Um, I believe that over there we had more responsibly more responsibility than we than we have over here. Cuz obviously we had we had to do grocery shopping, um our laundry, cleaning, while doing all that while studying and over here we just study now. Like we if we don't have the time, our mom does everything else. So I guess that's where my opinion towards what 63 said changes a little bit.

Moderator 22:51

Are you two the only siblings in your house or do you have other siblings?

51 22:56

No we're just the only ones.

Moderator 22:58

Okay. Okay, so let's talk about like expectations when it comes to household work. So to what extent do you think you were expected to take on more household work, child care, or family caregiving responsibilities um in your home? So for example, um, and parents may expect more from one person, um or partners or roommates may not have contributed equally, which is a little different for you all's situation because you all live together before college but maybe or before the shutdown but maybe if you could just compare and contrast how these expectations changed from when you were living on campus to now, back at home. Um 63, does that make sense?

63 23:45

Yeah, so um I would say that expectations, really didn't change much. Um, it was still like the same expectation to do good in school, to get good grades, to do well on your exams. Um, but like my, it's my my mom and my dad, like they knew that we studied, pretty much all the time. Um we're pre medical students so we we have a lot of classes and a lot of stuff to to do. And like when we came over here, they knew that we wouldn't be able to help as much as they would like us to do. So like they were okay with that. Um, with the whole semester like us just being up here studying um and they doing the rest of the things and we would help once in a while, but, like, expectations really didn't change much. It was basically the same.

Moderator 24:31

Okay, and 51.

51 24:35

It would be the same answer as my sister.

Moderator 24:39

Okay, so let's talk about how academics were impacted by the shutdown. So I'm just going to ask you about a variety of things that the shutdown may have impacted. If you don't think it impacted you, you can just say no impact. Um, so, 63, in what ways has the shutdown impacted your academic performance? Um this can be things like your grades, um and your understanding of the subject matter in your courses.

63 25:05

I would say no impact.

Moderator 25:07

Okay. And 51.

51 25:11

I would say impact. I was doing, do I have to describe it or no?

Moderator 25:17

Yeah, if you could describe it, that would be great.

51 25:19

Okay, um, I would say impact because in some classes I was doing poorly while I was in campus, but then when I came to the house and I was doing everything online my grading got better and all that.

Moderator 25:33

So why do you think it got better when you went home?

51 25:38

I just think I was allowed to use all my resources. So then I had, you know, more resources to do the things, but then in other classes, it went it was harder to understand the things, cuz it's not the same when it's online when it's compared to when it's in person.

Moderator 25:59

By resources, just to clarify, do you mean something like, like in your house? Like you had like physically more pins or something? That's a bad example. But or do you mean like you were able to use all of your notes and stuff while you were.

51 26:12

Yeah, all my notes and all that.

Moderator 26:15

Okay. All right. So, in what ways, if any, has the shutdown impacted your career goals, 63?

63 26:25

Um, I don't think it really has changed, like my career goals. I think it has strengthened them more. Um, since I am student who wants to go to medical school, um, obviously to study medicine, I think it's strengthened it because I've seen how um all of these the COVID and everything like many doctors have been dying, there's not many doctors. I think I would say it has strengthened my, my career goals. It's made them more clear as well.

Moderator 26:58

So you feel kind of inspired by like this global and it's motivated you and your work?Mhm, yes. Okay, and 51.

51 27:10

No impact.

Moderator 27:12

Okay. And so, in what ways has the shutdown if, in any way, impacted the availability of other learning opportunities? Um this can be things like internships. So 63.

63 27:25

Yeah. So, um because of the shutdown like I wanted to get into research uh on campus. Um, so that definitely is not happening anytime soon. So yeah, that definitely has um been a serious impact.

Moderator 27:41

Okay, and 51.

51 27:43

Another impact that we had was we were going to do an internship with a surgeon. But obviously, you don't want to go to hospitals, so that was cancelled on us.

Moderator 27:53

Okay, so did any learning or career development opportunities emerge during the shutdown and if so, what were they? So same order, we can start with 63.

63 28:10

Wait can you repeat the question because my

Moderator 28:11

Oh yeah sorry. Um did any new learning or career development opportunities emerge during the shutdown?

63 28:20

Um no, because our career learning opportunities like on the downside was um going to do an internship with a surgeon at a hospital, so that I don't think it's gonna happen like at least for another year. Um, and I would say like, yeah, I would say that that has been like the main issue.

Moderator 28:40

All right, and 51.

51 28:43

No impact.

Moderator 28:45

Okay. And so, um, in what ways if any has the shutdown impacted your confidence in your ability to successfully complete your bachelor's degree at all or in a timely fashion? So 63.

63 29:00

No impact.

Moderator 29:02

Um, 51.

51 29:04

Um a little bit of impact. Um, because for me it's harder to learn when something's online. And like to find the concentration to just keep focused on the computer, instead of anything else. I think that's making a little harder for me to understand materials. But I'm learn-, I'm getting used to. I'm learning new tactics to keep my concentration.

Moderator 29:30

Okay. So just to clarify, so do you feel like, you'll still be able to complete it in a timely fashion or do you feel like, um, your confidence is mostly just kind of shaken, because of how hard it is to transition to remote learning.

51 29:46

I know I'll be able to finish my bachelor's, I just think is what you said the confidence is a little bit shaken, but it'll, it'll get there.

Moderator 29:54

Okay. Um, so in what ways if any did the campus shut down impact your finances? So, 63.

63 30:03

Yeah, so, um, like I said in the beginning we were in at spring break with our family. Um so we had a per, we lost a flight. So, and they didn't give us refund, we had to buy new plane tickets, so we spent more money on that. And then we also had to purchase um an iPad, so that we could do our work because when we came over here, all of our notes, all of our notebooks and books, everything was at the dorm at Redacted. So we had to purchase an iPad so that we could keep track of all of our work.

Moderator 30:37

Okay, and 51.

51 30:41

Um same answer as 63.

Moderator 30:44

Okay And so let's talk about what your professors did or did not do during the move to online learning. So like what are some examples of strategies, tools, or technologies that your professors used that you found to be very effective, in that they made it easier to learn, and also things that they did that were really ineffective, that made it a lot harder to learn. So, 63.

63 31:08

So something that was pretty positive that our professors did, some of them, was that they kept like the class at the same time, live, and then they would upload the class later on, which was good because you could rewatch it if you needed to. Um I would say that was very good. Um that's pretty much all that they did, except our chemistry class, like our professor, he would pre record the class. And I would say like it was cool that he was pre recording it, but it wasn't it wasn't as effective as it could have been if he just gave the class live because that way people could ask questions along the way. He could stop once in a while. Like we would I would always have to be stopping like pause and then play, pause and play while I wrote cuz he went really fast. So I would say that was pretty ineffective from his part.

Moderator 31:53

Okay, and 51. What about you?

51 31:56

Um, some professors that I had did more discussion boards, and then they also had more office hours, which allowed me to go attend them and ask all the questions that I needed. And then for the strategies that they did that was ineffective was what 63 said about putting the, the class pre pre recorded.

Moderator 32:21

Okay. So what are some things that your professors did during online learning that made you feel like they cared about you and things that they did that made them made you feel like they didn't really care about you or their students?

63 32:33

So, I think that, like 51 said, the things that made us feel like they actually cared about us, was the fact that they held more office hours. Um our advise like our advisor um our academic advisor she would email us once in a while, see how we were, see how um our family was and how everything was going. Like that really shows that they care about us. But on the other hand, our chemistry professor, um all he would do is pre record the class and just send out like um email like standard emails all the time. So it really just showed that he was just you know like going on with the class but there wasn't really like, how are you? How is everyone doing? I hope everyone is fine, things like that.

Moderator 33:16

So did him just kind of going on, like, business per usual make you feel like,

63 33:23

Yeah.

Moderator 33:23

that he didn't care?

63 33:24

Like he would sometimes not even answer my emails, and that really bothered me because he would answer other emails and I was just like, I had a serious question. And so yeah.

Moderator 33:33

Right. Okay, and 51, what about you?

51 33:39

Um, something that made me feel that that professors actually cared, was that they answered to all the emails, anytime of the week even, even on the weekends. And then the other part would be the same as 63.

Moderator 33:57

So now let's talk about some things that your university did to respond to to the move to online learning. So what are some things that your university did pass fail options, town halls, remote counseling to help students be successful during online learning? And this can be any resources that the university provided, or other actions that they used to make students feel like they could be successful and feel supportive. Um, 63.

63 34:22

Yeah, so our the university, I would say they were very quick in moving everything online, like, even um the fitness classes at the IM building, they were all moved online. So that was like, that was really cool of them um from their part. Um, and the fact that they provided us with learning modules, um the tutorings. The Redacted tutoring it was also being done through zoom. Um Redacted learning was also being done through zoom. So that was like, at first, they said they weren't going to do it and then everyone started freaking out, and the students started pushing towards um them putting up the Redacted learning and they did which, that was really helpful.

Moderator 35:02

Okay

63 35:03

And they also did the pass and fail options but those were not applicable to everyone. Like for example, us that were pre medical students like medical schools won't accept a pass or fail option.

Moderator 35:15

Okay. Did the university make it clear like what things that you could do, instead of a pass/fail option?

63 35:22

No

Moderator 35:22

because you weren't able to use it? Okay. All right, and 51.

51 35:29

My answer would be the same as 63.

Moderator 35:32

Okay. So, what do you wish your university did to better help students be successful during online learning. So, 63.

63 35:41

Um I really don't have an answer for that.

Moderator 35:44

Okay. Is there a particular reason why you don't have an answer? Like you just felt like everything they did was great or?

63 35:51

Well, not everything like I would say like, up to date, they're still pretty slow on things because they said that classes would be a mixture of in person and online classes, and that's great. I mean, but it's also kind of dangerous cuz it's not like COVID-19 has not been eradicated from the US and it never will. But it it all we're seeing are spikes of the virus. And then I feel that they put that option when in real-- like they say it's a mixture of in person and online classes, when in reality like my schedule all my classes are online, except one discussion, whose class is online. So they're actually like, I would say like I'm if their answers are vague, they're not like sufficient. And we're going back to campus in August to pay for an apartment, when they could have said, you know what this whole semester will be online and that way apartments could have given an extend on the lease and all those things. Like they're I feel they're, to some extent, they're making us go back unnecessarily. That's why I have no answer.

Moderator 36:54

Okay, because you're not really-- your concerns with the university aren't really related necessarily to like successful during online learning, you're thinking about being successful during a pandemic. If that makes sense. Okay.

63 37:10

Yeah like being safe.

Moderator 37:11

Oh go ahead.

63 37:12

And yeah like being safe. That's like my main concern. I feel there, I feel there's like something else like government wise or that they want money or something like that, that's making them make those decisions.

Moderator 37:24

Got ya. Okay, thank you so much for sharing that. And 51, what about you?

51 37:30

Um, could you repeat the question because I totally forgot?

Moderator 37:33

Yeah, so what do you wish your university did to better help students be successful during online learning and kind of like what your sister said, it can be things like literally related to online learning, or just, you know, general life.

51 37:50

I guess what they did with the online learning and the zoom and the moving everything online, I think that was good. It didn't, in my opinion, it wasn't bad at all. But, um I would agree with 63 on saying that going back it's very dangerous, cuz people are coming from different places and you don't know where they've been and you know. So, I wouldn't really add more to the academic, there's probably things you could add more to academic, but I really don't know. And then, but yeah.

Moderator 38:24

Okay and just kind of a follow up question to both of you. So what are your feelings about going back?

63 38:32

I would say I'm a little bit scared. I'm, I feel kind of okay on going back because like for example, we're from Redacted. Um, so hurricane seasons here can be pretty intense. Like last season wasn't bad because we didn't really have any hurricanes, but supposedly this season is going to be bad. And if it were bad then we could be without power for three months, easily. So I think that going back would be a safe option for us to be able to finish the semester, but at the same time it's a little scary because like our apartment um the people from our apartments they were extending the leasing dates if you couldn't come back, but that was unless the university says that all classes would be online. And then they said that mix it would be a mixture of in person and online classes when in reality, um I don't know who they're lying to. All classes are online. There's hardly anything in person. So yeah.

Moderator 39:24

Okay. And what about you 51? So what are your kind of general feelings about going back?

51 39:29

Um I don't have any problem going back, but I do, I am a little scared with the in person class that we have. Um, and I guess right now there aren't that many cases in Redacted. They are, it's present, but I'm scared that when all students come to the same place, it it's gonna like blow up. So I think that's what I'm really scared about, and because Redacted is in the middle of Redacted, like to get out of there it's going to be harder and I think financially bad.

Moderator 40:00

Are you both in Redacted right now?

51 40:04

Yeah.

Moderator 40:06

Okay, so you have to fly back and forth between Redacted University and Redacted.

51 40:10

Yes.

Moderator 40:12

Okay. So, in general, are there any other ways that your ability to be successful in school was affected by the pandemic that we really haven't discussed today?

63 40:23

Um no, I don't think so.

51 40:26

No impact.

Moderator 40:28

Okay, um, those are the ends of my questions. Thank you all for participating today. Also I'm a twin too and you guys were giving me major vibes. Cuz I am also very very close to my sister. So, um, but anyway, have a great day. Thank you all for joining us.

Low SES Hispanic Men

Moderator 11:56

All right, I think we can go ahead and get started so welcome to the focus group. My name is Moderator and I'm a research coordinator from Redacted. Note-taker's going to be our note taker today. So just a little bit some rules for the focus group, we'll be asking a number of open ended questions, there are no wrong answers but people may have different points of view, please feel free to share your point of view, even if it differs from what others have said. Keep in mind that we're just as interested in negative comments as positive comments. We want everyone to feel comfortable participating, and we encourage everyone to talk. Because we have to keep this to an hour, I may need to cut you off so I apologize in advance. We want to get the full breadth of experiences and opinions but some of you may have very similar experiences and opinions. So if your answer is the same as another person you may simply say, my answers name it My answer is the same as a Sam's or whoever it is difficult for us to take notes on everything that is said in the group, so we'll also be recording the session so that we can transcribe it later. We won't associate your name with anything you say in the focus group. You may refuse to answer any question or withdraw from the group at any time. And we understand how important it is that this information is kept private and confidential. We ask that participants respect each other's confidentiality, and not repeat things said within the group to anyone outside of the group. Are there any questions before we get started. Okay, so first let's go around by saying your name, what year of college you just finish, and please describe what life has been like during the pandemic using one word.

45 13:52

Ok I'll start, ok I am 45 I just finished my freshman year, and mostly annoying I'd say annoying.

61 14:03

Um, yeah, I'm 61. I'm from REDACTED, I'm finishing my second year of college. And the word to describe it would be hard.

Moderator 14:13

Right. So just a little bit of an overview of the project. The covid 19 pandemic has presented big challenges for undergraduate students. As you know, universities across the nation have closed moving all instruction online, leaving little time for students or faculty to prepare. Many students have encountered challenges related to the closing of campuses relocation. And the movement to online instruction in this study we want to learn how the closing of campus in a pandemic in general has affected you. So we want your opinions about the good and the bad on how things went, as well as what your professors and universities did that was helpful and unhelpful. And what you wish they would have done. So we're going to start by discussing where you went when your campus shut down. So tell me about the space in the home you move to what space, do you have to do your schoolwork there and you tell me whether this space is private or shared and if you shared it. Who did you share it with

45 we can start with you again.

45 15:21

Okay, yeah. So, I went to my parents house and it's a little bit crowded, so it's mostly like shared so I mean, sometimes it's, like, we all have meetings at the same time it's kind of annoying.

Moderator 15:36

And were you living at home previously or reliving I was

45 15:39

I was in the dorms.

61 15:45

Yeah, I too was in the dorms, but with all the pandemic and stuff I moved with my grandparents to help them out with everyday stuff and it's really loud here. They don't know how to contain their voices so it was kind of hard concentrating on classes.

Moderator 16:05

When you're with your grandparents. Where were you doing work with like a shared space or did you have your own room.

61 16:11

Yeah, I have my own room, yeah it's it's kind of the room where, where everything is stored on the house but there's some nice ways I can work around.

Moderator 16:20

Okay. And were you the only one in this room working?

61 16:24

Yeah, yeah.

Moderator 16:25

Okay. What kind of challenges did you encounter attending classes or doing work related to related this to this space.

61 16:38

Um, go first, go first

45 16:41

no its fine... I think it's just harder to concentrate, given that you're in a shared space. Yeah, yeah, basically

Moderator 16:52

So was it the the noise.

45 16:56

Yeah. Is that just like, Yeah, I don't know like the feeling of like, just like people walking around and talking, yeah, basically.

61 17:08

Yeah, I kind of agree, cuz I don't usually study, sitting down on a desk, I used to really stay on campus and use the boards and really memorize things, or just go to the library, but sitting down, like it's not my, my first method to study and the noise yeah it's really a problem.

Moderator 17:31

Okay. What are some things that you or other people in the house. Did that made it easier for you to attend classes or do your schoolwork.

45 17:56

Um, for me, not much in general only when I had like proctored exams so usually I'm in like a shared space when I have like proctored exams or something I would like my parents will let me use their room for that, but most of the time. Nothing really.

Moderator 18:13

Okay.

61 18:15

I'm sorry. Can you repeat the question quickly I kind of forgot.

Moderator 18:18

Sure. What are some things that you or other people in the House did that made it easier for you to attend classes and do your schoolwork.

61 18:26

Oh okay okay so yeah my grandparents helped about a lot. Not with actual schoolwork, but they helped me organize stuff like they helped me keep tabs on what I have for today and when I have to finish and that that was a really big help.

Moderator 18:43

Okay. And is that something that you asked for their help or that they were willing to do.

61 18:52

I didn't ask for it but my, my grandma just offered herself to do it and it was kind of cool and really sweet.

Moderator 19:03

Now we're going to talk about some responsibilities you had after your campus closed and how these different from responsibilities you had before campus closed. So please describe your caregiving responsibilities, including child care elder care or caring for a loved ones, and how they were alike or different from your responsibilities responsibilities before the shutdown

45 19:26

I've had no such responsibilities before or after so.

Moderator 19:31

Okay.

61 19:33

I think I had less responsibility. While the campus was open because it was only like my, my goal was only to study that was the primordial thing. But when the pandemic occurred and all stuff. I'm the youngest in the family so I had to go grocery shopping I had to go to resolve any issues that my grandparents had or issues with their car, or the house or stuff. So my responsibility really became so to take care of them. First, and studies like studies were on a second plane, because you know family always come first.

Moderator 20:19

Okay. And kind of on that note, If you could describe any other household responsibilities such as chores cooking or caring for pets during this time, and how they were like are different before and afters shut down.

61 20:37

Yeah, no,

45 20:40

it was not a big deal for me but I mean like on campus you know just going to the dining hall it's really easy here like sometimes I would make food or do some stuff but like it wasn't a big deal for me.

Moderator 20:52

Did you see any, any change in other responsibilities besides cooking.

45 20:58

I mean only like small chores like everyone smiled help like washing dishes or cleaning but

61 21:08

I saw I saw a big change because in the dorm room it's all small I can take care of it real fast I can clean up real fast prepare food for myself, but being here with my grandparents in a house it's really, it's bigger it's harder to clean it's harder to sometimes tend to the necessities of my of my grandparents like lifting stuff like moving stuff helping sometimes I cook not all the time but it's mostly cleaning and organizing what has impacted me most.

Moderator 21:42

And to what extent were you expected to take on more household work childcare or family caregiving responsibilities and other people in your home. So for example parents may expect more of one sibling or partners or roommates might not contribute equally. 61?

61 22:04

Sorry. Yeah. Um, well it's. I'm an only child, really, and my grandparents are over 70, years old, so I have to take much of the pressure and I knew I was expected to to really take up all of the things that are big and things they could not do, because it's real hard for them so I intend it was expected from me that I do the big chores and stuff.

Moderator 22:37

And what made it feel like it was expected of you.

61 22:41

I there, it's, it's kind of a thing in my family where, where they haven't been really successful so they're always always pushing on me that they need me to be successful that they're laying everything on the table for me to be successful and they've sacrificed a lot. So that's already in my subconscious and I know that if they're expecting a lot from me, then I have to give them a light, because I'm I don't let them down. And you know, in studies as well as in, as in taking care of them.

Moderator 23:17

Okay.

45 23:23

Like, I'm almost an only child and like I don't really need to take care of my parents so there wasn't much of that for me, like mostly what I said before, just helping out with small chores. I feel like it's not really like expected of me to take on like a bunch of these things it's just like more I help a little bit.

Moderator 23:44

So now we're going to talk about your academics and how they were impacted by the shutdown. I'm going to ask you about how the shutdown has impacted, a variety of things. If you don't think it hasn't impacted something, you can just say no impact. So in what ways if any has shut down impacted your academic performance so like your grades and your understanding of the subject matter your courses,

45 24:12

definitely like... very negatively. Like I said before, it's harder concentrate and also, I feel like for all of my classes the like quality of teaching decreased so I had like a harder time learning because of that and because of like, I had a harder time like concentrating on them.

Moderator 24:32

What made you feel like the quality of teaching decreased.

45 24:37

I mean, this is like not being able to be hands on, I mean some of my classes had like technical issues with like the way they were being like, extreme so like a 80 minute class ended up being like 40 minutes because for half the time there was like issues and then I mean I think I had like a chemistry lab and there's no way to do that online so it just became like some, some like kind of meaningless assignments.

Moderator 25:02

Okay.

61 25:04

I agree with 45, it was really negative. And the campus that I studied in my college. There's a lot of professors are not like familiar with technology. So, if it was giving a virtual virtual transmission like you could sometimes hear dogs of the professor or the class, or simply in my calculus one class. They didn't even do a live class. He didn't even show up, he just said to read stuff from the book and it was really hard because I had to tend to YouTube and understanding calculus and on my own. Really, and the tests were pretty hard to because they were kind of manual. He sent photos of the test and you just had to copy it and send it back to him and that was really really annoying.

Moderator 25:58

And so you feel like this had an impact on your grades and understanding them.

61 26:03

Yeah. Yeah, I definitely think that I would have learned a lot more. If it was out of outside of a pandemic.

Moderator 26:18

In what ways if any has shut down impacted your career goals.

45 26:27

I don't think it has for me.

61 26:32

I don't think it has for me either. It's been hard but I'm, I'm on track, I'm still on track with what I have to do.

Moderator 26:40

Okay. How's it shut down impacted the availability of other learning opportunities such as internships.

45 26:53

I'm not at the point where I'm thinking about doing that quite yet so. No,

61 27:00

me neither I wasn't in in plans of having an internship yet.

Moderator 27:08

So did any new learning or career development career development opportunities emerge during the shutdown, and if so, what were they

45 27:17

Not for me.

61 27:21

Not for me either.

Moderator 27:27

Then my next question is in what ways if any has a shutdown impacted your confidence in your ability to successfully complete your bachelor's degree at all or in a timely fashion.

45 27:42

And I think, I think it's like I can still finish on time.

61 27:51

Yeah me too it, it has impacted me in a sort of way of thinking, how am I gonna pass next semester if it's online, but it definitely I think I will, I will achieve it.

Moderator 28:07

So is your confidence like. Sorry, one second. Did it with the online I sounds like a decrease a little or like you're a little bit more unsure but correct me if I'm wrong.

61 28:25

Yeah it decreased a little with our yeah it decrease a little

45 28:31

now yeah I mean, I think for me I didn't really say the answer clearly I guess, um, I think for me to decrease like I still think I'll be fine but you know it's like not as certain I guess.

Moderator 28:42

Okay. And then in what ways if any has the campus shutdown impacted your finances.

45 29:02

I was in the process of getting a part time job on campus, when we closed so I mean, that's not happening anymore, but I mean it's not like anything too big of a deal,for me at least.

Moderator 29:16

And then that job wasn't available anymore when the weather shut down.

45 29:19

Yeah, yeah.

61 29:23

I personally didnt encounter much finance issues I just had some money stored up, and I didn't have to buy food on campus. So I was kind of relieved.

Moderator 29:36

Okay. So now we're going to talk about what your professors did and did not do during the move to online learning. So what are some examples of strategies tools or technologies that your professors used that you found to be very effective, and that they made it easier to learn, or some, some things that your professors did that were also ineffective so it made it harder to learn.

45 30:06

Um, I can't remember anything that made it effective so I'll start out with the bad things. So for one of my classes, um, the only thing -- there wasn't anything that was live it was just pre-recorded. And I guess because there was no one there to ask questions, she would-- um, the professor just kind of like rambled on about something, trying to like answer supposed questions and it became like really hard to like watch and understand.

Moderator 30:35

What about it made it hard to watch?

45 30:38

I guess like -- it would-- did the, the pre recorded things were like long and not very useful. It's like she would like, I mean, I think it was with a good intention but it did not work out well. So, I don't know, like, I guess since she didn't have anyone there to ask her questions she would just like kind of explain something, but then she just like keep talking about it for way too long trying to like, make sure she covers everything. And it just like it got difficult to like keep, keep up with and like watch the whole thing.

Moderator 31:10

Okay. And then if you think of anything that was effective you can feel free to jump in whenever.

45 31:16

Okay.

61 31:18

Well I agree, I agree with 45, it was really things that were not effective because in my-- in difference to 45, I did have live classes, but most of the professors didn't even bother to answer questions on the live stream, you know? They just said that you can text them apart, that you can email them after class when when you did email them, they didn't ever reply. So that, that's kind of basically every class I took except one which would-- I think it was effective because it was it was a tennis course. I really, I really just wanted to learn tennis and I and I. I enrolled in that course but the professor did a really cool thing, which he tried to teach us the rules of the sport, but also the assignments were like visual, like he-- he would send a video about a movement that you have to do, and you have to record yourself doing that movement and He would give you pointers and he would give you ways to really practice it to so you could become better at home, you know, and it was, it wasn't the same experience as being on campus and actually practicing it, but I can, I feel that-- I am not, I have not abandoned the sport or the course because of the way that he tried to teach it.

45 32:48

Uh, okay so I actually thought of like two things I think they like contrast pretty well. So it was --so for my, my chemistry, this is a ba-bad thing first, so I had a chemistry lab and I think I said this before right it was-- There's no way to really do that lab online so they just gave us like some assignments about the subject. It was just like answering questions so it really wasn't... It just kind of didn't it, just kind of became a different class and I didn't really learn much from it. But then on the other hand, I had a physics lab which they used some, like an online lab thing, and it was actually pretty helpful because th- the in person labs sometimes we couldn't get things quite right, I mean the online one it's like program to work so um I thought that was a pretty good thing.

Moderator 33:39

So could you explain a little bit more about why the or why the like online lab part for the physics lab was helpful?

45 33:48

Because um, so like sometimes the, the one that was in person, like we were supposed to do a, like certain experiment but like the equipment was like kind of old and it didn't really work out, like the online one would always work so it was like clear what was happening and I feel like I actually learned, like th- the stuff that we were learning in like the in like the lectures, like, I saw how they applied better on the online version.

Moderator 34:13

Okay. I see, I see. What are some things your professors did during online learning that made you feel like they cared or that they did not care about their students?

45 34:31

So, about that class that I was talking about that only had pre recorded lectures-- Um I mean, I guess she tried but like at the same time there was like no written or live thing to ask questions and um, like everyone who sent emails like it's a bunch of people didn't get responses or like it took a long time to respond so that class was like pretty like annoying to, like, um, if you had like questions, it was, it was pretty annoying because... I don't know if you don't have anything live it's --it's kind of difficult to ask questions. On the other hand, like, I had um, a TA for my class that he basically was like, basically the whole time he was awake, he was like on WebEx just waiting if anyone wanted to join to ask questions so that was, that was helpful I guess.

Moderator 35:24

Okay.

61 35:27

So, so I think, I think the most big thing that I think that denotes the importance of them not caring. It's.. my professors scheduled tests at the same time, same day and it happens, multiple times. And then when I try to explain to them that I have another course that it's the same hour, the same test, they would argue with me, and they would say that I have to take it sooner but really, some Professor did-- didn't budge. So I had to go on to other professors and explain what the hell was happening so they could arrange me in a different position to take the test separately.

Moderator 36:14

And then, were there any things that your professors did that made you feel like they cared about their students?

61 36:21

Well, um, I had a professor who really... He just gave out his number. He said if you need some explanations-- he was an economics professor. If you need some explanations if you need to, FaceTime me to call me just do it at any time. And I put it to the test. One day was like kind of midnight and I and I texted him and he responded so that showed that he really cared.

Moderator 36:50

And now we're going to talk about how your university responded to the move to online learning. So, what are some things that your university did to help students be successful during online learning-- you can mention any resources the university provided or actions they took to help students be successful or feel supported. So you can mention things like pass fail options, town halls, or remote counseling are all examples.

45 37:19

I mean, yeah, I'd say the main thing that they--- I mean those three things that you listed-- mine, mine had and I--I don't uh, I feel like the only thing I really benefited from was the pass/fail. Yeah, I didn't really use anything else.

Moderator 37:36

Were there other things that the university, provided, like in addition to the examples I mentioned?

45 37:43

Um, I don't really remember anything else.

Moderator 37:47

Okay.

61 37:49

I agree with 45--The pass or fail was, I think the most helpful thing that the university, provided, but I can't think of another one, honestly.

Moderator 38:03

Is there anything you wish your university did better to help students be successful during online learning?

61 38:21

Well, yeah I-- I don't know exactly what I would wish for the university did to help us. I know that there were some parts of --of campus of students that they could have helped. I know they have the information to do so. Maybe I don't know exactly how. But I know they could have helped educationally at least a bit.

Moderator 38:48

Um, Could you elaborate on what kind of help, they could have given or you were saying a specific group of students.... Uh, what you mean by that?

61 38:58

Yeah, like they know, they know some of college students in my campus that they don't have any connections or they don't have the materials to keep remote learning. So I think that with all the pandemic and stuff and having such a big campus. They could have enabled, I mean some type of-- of space for people that don't have what they need to keep studying. Some internet connections I think some of them could drive to campus. I think they-- well, when I was in the dorms, they kicked us all out. Like, I think they would, it could have been really... They could have investigated more on who needed really to stay on campus just because of materials or the internet or-- or places to live. And they could have provided that you know, and I don't know it's it's really complicated, because maybe the libraries and virtual libraries are not that helpful. But I think the most part where it would be helpful is to really help those students who needed it like Internet connections, materials, and a place to stay in campus.

Moderator 40:16

So more resources for students?

45 40:20

Yeah, I was trying to think this whole time I mean, it's difficult I think one thing that maybe could have helped was, um, to have some sort of standard that classes had to follow, because like all at once you move to online like each class kind of did their own thing so like some of my classes just got rid of exam some, some of them had like proctored exams, some of them had live lectures and we had pre-recorded. And I don't know I just feel like it was even more tiring that you had to like keep up with what is going on in each class so maybe if they had like some sort of standard like they don't have to like, I don't know if they had to like dictate exactly how each class should be but, you know, have some sort of standards so it's not like each, each professor deciding their own independent thing.

61 41:07

I strongly, I strongly-- I'm sorry --I strongly agree with 45 here. Um, I think that if we had a standard for every class we wouldn't be surprised by inventions, or things of the professor, like we know what -- we would know what to expect from every course. So it would be a little more organized and a little bit easier than having to deal with, with the way the professor, adjust the course to his personality.

Moderator 41:37

This is to both--. I'm sorry, go ahead.

45 41:38

Yeah, I actually want-- just one more thing. Um, kind of on the same note-- Also, one of my classes, just kept changing how they were going to grade things like-- like after went online they made a plan but then they changed it like three times. So I like I really wish that, like if there was some sort of standard that wouldn't happen so in one of my classes like they said that the final was optional and you would you can take it only if --- like, they would only consider the score if it benefited you. So I thought I was set to get an A. But then, like, not--not like the day before I don't remember exactly when but like not too long before the exam they changed it so that everyone had to take it and they, it would count, but if-- if one of your midterms was lower than your final than it would replace it. So then I had to-- so I was expecting to like not need to take it, but then they actually made me take it and that definitely wasn't helpful.

Moderator 42:40

Okay. And in general, are there any ways that your ability to be successful in school was affected by the pandemic in a way that we haven't discussed yet.

45 42:55

Think for me we--we've covered all of it

61 43:02

Um, I am sorry the question was, the goals to be successful were they affected by the pandemic, that was question?

Moderator 43:09

My question was like in general, are there any ways that your ability to be successful in school was affected by the pandemic like in a way that we haven't discussed yet.

61 43:20

No, I think we discussed it. In my case, I'm really practical, I like to use a boards, I like to use libraries I like to use facilities to learn. So, that that was impacted me most in--in the closure of the campus and learning from-- from a desk, it's really more hard for me.

45 43:41

Actually I thought of something. Um, when we were on campus, um, we all used to like go to the lounge in our dorm and study together and like I just always studied on my own after going to the pandemic... I mean, I think like just being with other people helped me like focus more on my studies that I guess, I guess, yeah, that's --that's one thing that was bad.

Moderator 44:03

Okay. All right, so that's the end of my questions. Thank you for participating today if you could just send us an email with the name that you use during the focus group will be great.

45 44:18

Ok, thanks.

Moderator 44:19

Thank you.

High SES Hispanic Men

Moderator 11:50

Okay, yeah, we can hear you. So, all right, and, okay. So, welcome to today's focus group. My name is Moderator. I'm a research coordinator from [Redacted]. Today our note taker will be Moderator, who can just turn on her um microphone and just say hello. Perhaps turn on your camera Moderator.

Hi everyone, thanks for coming today.

55 12:15

Hi

53 12:15

Hello

Moderator 12:17

So today we're going to be asking a number of open ended questions. There are no wrong answers, but people may have differing points of view of course. Um please free to share your own point of view, even if it differs from someone else's. On the other hand, some of you may have very similar experiences to each other, and if your experiences are exactly the same, you can just simply say, my answer is the same as Sam's. So we're just as interested today in negative comments as we are in positive comments. And we want everyone to feel comfortable participating and we encourage everyone to share today, but because we need to keep this to an hour, I may need to cut you off, so I apologize in advance. I'll raise my hand like this, um so that way you know to finish your thought if we need to move on to another person. It's going to be difficult for us to take notes on everything today said in the group. So we will also be recording this session so that we we'll we can transcribe it later. Um, but nothing that you say in the group will be associated with your name. Um in addition, you may refuse to answer any questions at any time or withdraw from the group. In addition, we're going to ask everyone today to just respect others confidentiality by not repeating anything said outside of the group. So, before we get started. All right. Okay.

Okay. So, um. All right. Well then, let's get go around and first start by sayiing what our name is and what year in college you are, um, and one word to describe what life has been like in the pandemic. So, we can start with um 53. Is that how you pronounce your name?

53 13:59

Yes.

Moderator 14:01

Okay, so what year are you in college, and um what is life been in a pandemic in just one word?

53 14:11

I'm a jun- I'm a junior in college and in one word I suppose it would be um different.

Moderator 14:20

Okay.

53 14:21

I go to REDACTED.

Moderator 14:26

All right. Um, okay, and so 55.

55 14:31

Yeah so hi. My name is 55. And I am in my second year now. I just got done with my first year, and I am a computer science major in the University of REDACTED uh REDACTED campus. Yeah.

Moderator 14:49

Okay, and what has the pandemic been for you if you could describe it in one word?

55 14:53

It's been very, um, I'd say very uh unnerving, to a certain extent, you know. It's been kind of unnerving in a a very, you know the personal sense. Um also in the academic sense. You know cuz like this this level of of uncertainty to what's going to happen. And yeah, just been very unnerving, to be honest.

Moderator 15:14

Okay. Well thank you both for sharing. So let's get to the bulk of our focus group questions. So the COVID-19 pandemic as you know, has presented really big challenges for undergraduate students. So universities across the nation have closed moving all instruction online with very little time for students um or faculty to prepare. So many students have encountered challenges related to the closing of campus, relocation, and moving to online instruction. So in this study we want to learn how the closing of campus, and the pandemic in general has affected you and we want your opinions about the good and the bad, what went well, what didn't go well, what you wish your universities. So let's start by discussing where you went when your campus shut down. So, tell me about where you moved to and the space in that home where you did your school work. So whether that place was private or shared. Um in general, sort of a description of it. So 53, we can start with you.

53 16:14

Um, where I went when my school closed was I in my own room really, that's where I did most of my studying and schoolwork and also had my classes, as it was on a different floor from everything else.

Moderator 16:29

Okay, and was that space, uh private or did you share that space?

53 16:36

Uh it's a private space.

Moderator 16:38

Okay. And uh just to clarify, so did you move back to your childhood home or.

53 16:46

Oh, uh this was just the home we've always been.

Moderator 16:49

Okay. All right, and was that different than where you lived on campus?

53 16:54

Oh, I lived, I lived, I commuted to school, so.

Moderator 16:57

Okay. All right. And 55, what about you?

55 17:02

Yeah, so uh when the pandemic started, uh I guess my university you know just started slowly closing down and started moving towards online classes. And you know I had the privilege that I have a separate room in my house where I can do my work. I have like an office room um where I usually have all my things and um I am a single child. So, uh I basically the room, I have this room to myself and I can just be here and work on my things and I have my computer, right. And I just, you know, it's it's just my personal space, and before the pandemic actually, I used this space for uh just doing recreational stuff you know like you know talking to my friends or um just relaxing or watching stuff. But now it's kind of become where I uh spend most of my time on, you know, like 24 seven basically working on stuff um. And yeah, it just I basically just have this office room here where I do most of my things. Place where I can...

Moderator 18:00

Okay. So, what challenges did you encounter attending class or doing your schoolwork in this space? So 53.

53 18:08

Uh one of the challenges was that, um, I've had family over as well. So that can sort of get into the focus and concentration, especially since uh my brother he brought his cat over. So, dealing with uh pets and all that stuff has kind of just added a layer of I can't really fully focus on my schoolwork because I have to watch other things as well.

Moderator 18:35

Okay. And what about you, 55?

55 18:39

Some of the challenges that I've had is, um, usually my family members uh there sometimes like there's been some interferences you know with like, um especially with things that I have to do around the house and things that I have to take care of. You know um, sometimes they need help with stuff and sometimes there's just chores that I need to do, or things that I need to get done and so usually whenever I have classes or something I sometimes have to, you know, interrupt that space with uh having to take care of things in my personal life um but thankfully my at least my family has been pretty respectful with the way that I've done things and they've, you know, respected my time in that sense. And so I've I've appreciated that. But honestly, I think that that's been what's been hardest, you know, just the stuff that's come up, personal and having to deal with it.

Moderator 19:29

All right. So what are some things that you or other people in the house did that made it easier for you to attend class, or do your schoolwork? So, 53, we can start with you.

53 19:45

Um, they probably the easiest thing was the fact that since I was so close to home, I didn't really need to worry about commuting or doing things, or to, or trying to find something to eat, because that was all because I can always have ingredients at home, those yummy leftovers so I didn't have to like, wait for like the line for a microwave and all that stuff.

Moderator 20:08

Okay. And uh what about you, 55? What are things that people did in your house that made it easier for you to attend class or do your schoolwork?

55 20:17

Well, most people in my house, they like I said, they usually respected their own like our their own spaces, you know. So they try not to intervene too much in my space. Um they usually I feel like I've we've come to like a consensus in my house to kind of, you know, respect each others spaces and, you know, keep like an appropriate um, you know certain hours yeah a respectable distance I'd say when it comes to work. Um thankfully, for me, I feel like the transition wasn't that hard to online stuff because most of my work that I already did in college was already transferred to online, to a certain extent, and most of the stuff that I have was already digital, so I feel like it wasn't that much of an effort. So I feel like um uh at least my parents they always kind of, you know, helped me do stuff digital as well so I feel like they kind of helped me on that sense to kind of just transfer everything over and just it just made the transition easier for me.

Moderator 21:12

So now let's talk about other responsibilities you had after your campus closed and how those may have differed from responsibilities that you had before campus closed. So, to start describe any caretaking responsibilities you had like including child care, elder care or caring for ill loved ones, and how that was alike or different from your responsibilities before the shut down. So, 53.

53 21:38

Uh generally most of my responsibilities stayed the same. Um I still had to, but it generally included more like housework now such as uh making sure that the that everything in the home was uh clean because my mother uh works in a hospital so it's extra important that the house itself is also clean sometimes, as well as uh having to take care of my uh brother's pet.

Moderator 22:06

Okay. And what about you, 55?

55 22:10

Uh, personally, I um, like I don't have a bunch of ties to my family cuz most of my family's either in the states, and not in REDACTED right now um. So I feel like what we, what I have to take care of the most when it comes to family was more like my you know my core family like my parents and stuff. Um so my grandparents that live nearby, uh but it's kind of funny because you know the way to take care of them is by not trying not to you know be too much in contact with them uh because the with COVID, the more risks they are in, you know, getting the the virus. So it's been you know it's been kind of like in a weird position where I want to help but like at the same time it's like oh the best way to help you is by you know trying to like keep my distance. So, yeah, it's just been on that and like I said, um helping my parents around, especially in times like these around the house and and things that need to be done and precautions that need to be taken. So I feel like that's the definitely what I've done the most in this pandemic.

Moderator 23:06

Okay. And so, um, let's talk about other household responsibilities you may have had like chores, cooking, um caring for pets during this time and how they were alike or different from before school closed. And I know 53 that you mentioned that you were taking care of your brother's pet. But if you could talk about other household chores um to hear about them.

53 23:30

Um so one, one new thing I had to do was um, I think it was the mop probably um mopping around the house. Like normally my mother would do that but because work has been so busy I've been taking over for her, as well as um on certain days, I also cook for the family as well.

Moderator 23:52

Okay. And what about you 55?

55 23:56

Uh kind of like 53 as well, I have to take care of my pets cuz I live in a pretty rural area so we have quite a bit of animals that we need to take care of. Uh but in that sense that you mentioned, I would say that definitely I've had to start taking care of a lot of the sanitary chores. You know, kind of cleaning stuff as they come, if we go buy something making sure that it's clean, and stuff like that. Aside from that, um, you know just your typical chores, but I feel like now with COVID it's like a little you know a little bit more than that in the sense of that it's always you're gonna have to go a little bit you know the extra mile to do things, uh especially when it comes to you know with hygiene and stuff so yeah. I feel like just cleaning and taking care of both my parents you know and any help that they need outside or my animals, I would say is, what I would have been doing the most.

Moderator 24:44

Okay. So, to what extent were you expected to take on more household work like child care or caregiving responsibilities, um than other people in your home or just in general? So for example, did your parents have a certain expectation of you or did they expect more from one person? Um so 53, we can start with you.

53 25:09

Um taking care of the pet was a bit of a surprise because my brother was staying over for a while, but he was also but he was also, but he also left and then said he was going to come back and they didn't want to subject the cat to that sort of stress of constantly moving. So it was a bit of a surprise that I ended up taking care of the cat, and I've gotten bitten and stuff but other than that most of it has pretty much stayed the same in most occasions. Like nothing really too surprising.

Moderator 25:42

So were you were you expected by your parents to take care of the cat?

53 25:49

Uh, no at first because they thought I would just let my brother take it with him so me saying that I would take the cat kind of surprised them but yeah.

Moderator 26:03

Okay. And what about you, 55?

55 26:06

Honestly I expected to take care of people a little bit more closely, you know, like maybe take care of my grandparents or talk to them more, or just having to take care of my family a lot more. But truly, I what ended up happening was uh like I said, I I realized that the best way to take care of them was by trying not to be around them as much you know and having my precautions. So, to a certain extent yeah like I I definitely thought it was gonna be a little bit more um close and personal in the sense of having to take care of people but I realized that having to take care of people it's especially in pandemic it's a lot different than I thought it would be. So yeah, my expectations were different.

Moderator 26:46

So were you expected by your family or by your parents to take care of people, or was that an expectation that you had?

55 26:55

Um, I feel like it was more of a me expectation to be honest. My parents um they usually respect me and they don't really expect too much from me. Uh like in that sense you know they really try not to push me to anything. So, I feel like it was more me that I thought that it was going to be a bit a bit heavier.

Moderator 27:14

Okay, so now let's talk about your academics and how those were impacted by the shutdown. So I'm going to ask you about a variety of different things. If you don't think you were impacted, you can just say something like, no impact. So in what ways, if any, has the shutdown impacted your academic performance? These can be things like your grades and your understanding of the subject matter in your courses. So, 53.

53 27:39

Um I feel like it's impacted it quite a bit. But in different ways so like, it feels easier to get access to like, resources and organized study groups with other people.

Moderator 27:55

Okay. And um, 55 What about you?

55 28:02

I mean, you you're asking about my study life right specifically?

Moderator 28:06

So yeah. How has it impacted your academic performance, um or your understanding of the subject matter in your courses?

55 28:14

I definitely feel like my subject matter got affected a little bit because I didn't get the chance to learn as much as I would personally, you know.

Moderator 28:27

Okay.

55 28:27

I definitely I feel I could have learned a bit more with the dynamic of the classes.

Moderator 28:33

Why do you feel like you could have learned a little bit more? Could you just say a little bit more about that?

55 28:39

I feel like I could have just gotten invested more in the classes because the way that my professors, you know, talked with me I feel like it would have been better personal and I feel like I'm more of like close to, you know, person to person learner more.

Moderator 28:57

Okay, so in what ways if any has the shutdown impacted your career goals? So we'll start with 53 again.

53 29:05

Um sorry the voice got a bit uh

Moderator 29:09

Yeah, I can repeat it. So has the shutdown impacted your career goals in any way?

53 29:15

Uh not really no, because one of my career goals was uh to work for the city, and like uh, I'm an environmental engineer so I'm hoping maybe like in waste facility treatment. So I feel like that has stayed relatively the same, though, crap, though like I guess I am sort of considering, since it's not exactly a sanitary job, but yeah.

Moderator 29:43

Okay, and what about it are you considering? Like you're still considering going into it or you're considering doing something different?

53 29:49

Uh considering doing something similar, such as rather than instead of working with waste treatment I could be working with air quality or something like that because it's a bit more sanitary than the waste facility treatment plants.

Moderator 30:05

Okay. Um, and you 55.

55 30:10

Honestly, if anything, I feel like the quaratine helped my, my career a little bit more because now, like what I'm doing is computer science right. And I feel like those goals are even stronger now because I see how much people really do need technology, you know, especially in things like these. And I feel like I would be really helpful in the future if I could impact people in this way, you through computer science. So, if anything, I feel like it's solidified what I already wanted.

Moderator 30:42

Okay. So in what ways, if any, has the shutdown impacted the availability of other learning opportunities like internships? 53, we can start with you again.

53 30:54

Um, I feel like it's gotten a bit in the way of internships because uh where I live in uh REDACTED, it's where I live in REDACTED it's like right next to REDACTED but since I don't have access to a car, I need to take public transportation. And since most internships are in the city, I'm not really comfortable taking public transportation there because you need to be next to people in a closed space and that's the opposite of what you want to do in this pandemic.

Moderator 31:24

Yeah, and 55, what about you?

55 31:29

Can you repeat the question? My internet kind of cut out a bit.

Moderator 31:32

Yeah. So, in what ways, if any, has the shutdown impacted the availability of other learning opportunities like internships?

55 31:40

Yeah, so, talking about that I I recently made a a like I I did a like a thing to go to a Microsoft internship right, but due to the pandemic um that actually kind of got canceled because of measures they had to take. And so I feel like definitely my opportunities that I had set during the semester definitely got impacted differently. Um, but I feel like in a, in a way, I also got offered new opportunities you know like um this research program that we're doing right to help you guys. And like other investigation opportunities that have been presented to me through online methods as well. So definitely I feel like it opened some doors, but it also closed some ones that I already had planned for. So I feel like it's a little bit of both, you know.

Moderator 32:30

Okay. So, um, were there any learning opportunities that emerged, something, um so new learning career development opportunities, and if so, what were they? So, 53.

53 32:46

Uh I felt like I didn't really have uh many because I feel like this kind of came in so suddenly that like it sort of blindsided me in a way that like I was kind of just focusing on what do I need to do rather than what can I do?

Moderator 33:04

Okay. And um 55, so you sort of touched based on this a little bit, but was there any new opportunities that emerged?

55 33:14

Yeah, um, I feel like, if there's any new opportunities that emerged was especially stuff with investigations. I feel like definitely investigations are something that I knew I wanted to do but not so soon in my career. So I feel like now I've had a lot of chances to do investigations you know and do things related to that and maybe even touch things on bioinformatics which is one of my topics I am interested in and due to the pandemic I've been able to go more in depth over it, you know. So that definitely has been something that has been open for me during the pandemic.

Moderator 33:49

Okay. So, in um what ways um, if any, has shut down impacted your confidence in your ability to successfully complete your bachelor's degree at all or in a timely fashion? So 53, we can start with you.

53 34:04

Uh I do have some concerns with like how um uh with how uh remote learning is kind of like taking away experience from me because um one of the classes, two of the classes I'm taking uh Earth, both uh Earth Systems Analysis and Archaeology, those are classes you would generally want to take in person, as they're in uh, as they have like lab settings, but because of the but because we can't access the labs due to remote learning, I feel like I'm sort of missing out on some experience.

Moderator 34:43

Okay. And um what about you, 55?

55 34:47

Sorry can you repeat the question again?

Moderator 34:49

Yeah, so in what ways, if any, has the shutdown impacted your confidence in your ability to successfully complete your bachelor's degree at all or in a timely fashion?

55 34:58

Uh I feel like so far everything is up to schedule with my bachelor's. Um nothing has really been affected in such a negative way, I would say. Um I feel like I'm taking the classes that I need to take. You know I'm pretty up to date. I would say that um definitely maybe my performance in classes would be a little bit more, maybe a bit better or maybe I would be able to be more invested in the classes of my bachelor's. But aside from that, I think um not much to be honest. I feel like everything's been pretty on check.

Moderator 35:33

Okay. And so in what ways if any did the campus shutdown impact your finances? So, 53.

53 35:42

Uh, it helped save me money because I didn't have to spend on public transportation. I usually buy monthly. But since I didn't need to travel, like, it kind of helped me save money a bit. I also have to save got to save money on food.

Moderator 35:57

Okay. And 55, what about you?

55 36:06

Could you repeat the question again please?

Moderator 36:07

Yeah. In what ways, if any, has the campus shutdown impacted your finances?

55 36:13

My finances, I feel like the help of the government has given us has helped me quite a bit. Um, but aside from that, um not much to be honest. I feel like I've been paying for my classes you know everything's pretty much the same. And, yeah, that's pretty much what I think has happened.

Moderator 36:36

Okay. So now let's talk about what your professors did and did not do during the move to online learning. So what are some examples of strategies, tools, or technologies that your professors used that you found to be very effective, so they really really helped you learn, or very ineffective during online learning, so things you didn't think really helped you learn? So 53.

53 36:57

Uh being able to form groups of people to study with over the internet has kind of helped a bit more. I mean there was nothing stopping us from doing this before the pandemic, but because everyone's kind of been forced to I feel like there's more people collaborating has made it a bit easier.

Moderator 37:17

Okay. And where were you meeting online with people to study in groups? Was it just over zoom?

53 37:23

Uh, what were we using?

Moderator 37:25

Yeah like so when you were using to study groups was it that something like a university software or was it zoom, or just text messaging or?

53 37:35

Uh, it was in group chats.

Moderator 37:37

Okay. And, um, 55, what are some things that your professors-- tools or technologies-- used that you found to be really effective to help you learn or ineffective?

55 37:51

I definitely feel like, um, videos that my professors had prepared, you know like video material has been really very helpful. Um, aside from that, I would say that most of our classes were on zoom and Google meet. Right, zoom and Google meet have been what I've been using the most, which have been very helpful to stream their classes and their screens so that we could see what's going on a little bit. And aside from that, I'd say some note taking software that I've been using has been very helpful. Um, and yeah, I feel like that's been what technology that's helped me the most for now.

Moderator 38:25

Okay. So what are some things that professors did during online learning that made you feel like that they cared about you, or their students, or that they didn't care about you or their students. So 53.

53 38:39

Uh sorry, it kind of got cut out of it.

Moderator 38:42

Oh, you're fine. I can repeat it. So what are some things that your professors did during online learning that made you feel like they cared um and things that they did that made you feel like they didn't really care at all.

53 38:54

Um I could feel that some professors were like had a hard time feeling enthused about the remote learning because it was so different and I mean I don't really blame them because I feel like that sort of feeling was on all of us, because it was kind of awkward at first. But uh they managed to find a way to make everything work so I feel like they uh so I feel like they were able to do everything they could, because some classes were just new to it because they were labs.

Moderator 39:26

Okay. And so you think that their like not really being enthusiastic about the online learning um reflected how they, how they cared about their students or didn't care?

53 39:38

Uh I felt like they cared. It's just that uh I guess the pandemic has put worries, like sort of like over that sort of overtakes their, uh how much they care, I suppose.

Moderator 39:55

Okay. And 55, what about you? What are things your professors did that made you feel like they cared, or didn't care about you or their students?

55 40:05

I feel like they definitely took certain measures to make sure that we you know tried to learn more. Uh obviously this doesn't apply to every professor, but I feel like the professors that did you know try and like, um apply new technologies, even though they did not know how it worked, professors who the made the effort to, you know, go the extra mile and use new software and new apps, and they just you know the ones that I mentioned to help us learn more, I feel like like those little steps, you know that extra mile that they took to help us learn has what's been very helpful, especially the way of like teacher videos you know professors did like dedicated sessions and such. So yeah, I feel like those are some examples of the things that professors have done.

Moderator 40:47

Okay. So now let's talk about how you're university responded to the move to online learning. So what are some things that your university did like pass fail options, town halls, remote counseling, to help students be successful during online learning? These could be any resources that you felt like your university provided, um more actions that they took to help students be successful and feel supported. So, um 53 we can start with you.

53 41:17

Um, one of the things the school did, they did offer pass and fail options, but they also uh moved the whole tutoring uh their whole tutoring program online.

Moderator 41:32

Okay. And 55, what about you?

55 41:39

Uh personally, my professors did um, at least for this semester, they did pass or fail options. Um, they aside, I mean that was like the main thing they did, to be honest. Aside from that, um I think they took into consideration and gave us a few time you know a bit a bit more time to do our work and our projects. But aside from that, um I think that's about it honestly, two of the main things that happened during this semester.

Moderator 42:04

Okay. So what are some things you wish your university did um to better help students be successful during online learning. So 53, what about you?

53 42:15

Uh yeah, there was the whole tutoring thing but they also uh were able to provide laptops to students who needed it.

Moderator 42:24

Was there anything else that you wish they would have done?

53 42:26

Uh I kind of that they um would have, like, reduced a bit of their course uh fees, because we're not using the school so we're not using school uh tech so I feel like some fees uh waived a bit for the year. Yeah I think they did pretty good overall.

Moderator 42:56

Hi, 58. Um we just started but we can go through the questions again when I let um with you when I let 53 and 55 leave because we already almost done with the questions. Um, so, um in general, are there any other ways that your ability to be successful in school was affected by the pandemic that we haven't discussed in previous questions. Oh wait, 55. Sorry, I forgot. Um what do you wish your university did to be successful during online learning?

55 43:32

Uh, I wish they were a little bit more lenient, you know, um, especially with like the amount of workload they gave us and with the time to do things I feel like they did an okay job but I feel it could have been a bit better. So I feel like those are two of the main things that personally I would have wanted, but I feel like honestly given the situation and the suddenty of everything, it was it was all right. I I feel like it was okay.

Moderator 43:57

Okay. So um 53 and 55, um for you, in general, were there any other ways um that your ability to be successful in school was affected by the pandemic that we haven't discussed?

53 44:14

Um I feel like I do better when um my tutor, like uh I can like have those lectures just fine over the over the internet but when it comes to stuff like tutoring, I always prefer in person tutoring as I feel like it's a bit more in depth than just uh digital tutoring.

Moderator 44:38

Okay. And 55, was there anything we haven't discussed?

55 44:44

Um, I feel like definitely the university should develop a bit more mental health programs for their students. I feel like um those cont- type of contributions should be very prominent in this type of situation, because mental health's something that's you know impacts everybody. And I feel like exa- for example my university has a program for that, and I feel like they're gonna start expanding it more which is nice. So I feel like that is something that I would like to see more of definitely.

Moderator 45:12

Okay. Um, all right, 53 and 55. Those were the end of questions. So you guys can go ahead and leave if you would like. And then I can get started and ask you some questions 58.

55 45:25

All right, thank you very much. Okay.

Moderator 45:28

Have a good day. Thank you.

Hi 58 How are you? If you feel comfortable you can turn on your camera. It is just me and Moderator, our note taker. So I am a research coordinator.

58 45:45

I'm sorry I had my I had this scheduled for three so like I'm sorry I'm like really late.

Moderator 45:50

No you're fine life happens. So we can just ask you um here it'll just be us though, so there won't be other people.

58 45:59

Okay.

Moderator 46:00

Um but okay so my name is Moderator. I'm a research coordinator here at Penn State, um and our note taker today is going to be Moderator.

Hi, thanks for coming.

58 46:11

Hi

Moderator 46:13

Um, so um we're going to be asking another, a number of open ended questions. There aren't really um wrong answers. There's no one else here so there isn't really a need to discuss differing points of view but we want you to share both negative and positive comments. Um we want you to feel comfortable participating today. Um but it's it's just you so, uh.

58 46:38

Okay.

Moderator 46:39

Uh so it shouldn't be too much of a problem with time. Um, it's not really possible for us to take notes on everything that is said in the group. It's only possible for someone to type so fast. So we're also going to be recording it, um but we're not going to associate your name with anything you say today. And you can

58 46:56

Okay.

Moderator 46:56

refuse to answer any question or withdraw from at any time.

58 47:00

Okay.

Moderator 47:00

Does that sound good? Okay.

58 47:01

Yeah, sounds good.

Moderator 47:02

So, um, what is your year in school that you just finished and just one word to describe what life has been like in the pandemic for you?

58 47:13

Um, I just finished freshman year. I'm now a sophomore, about to finish up my first uh sophomore summer semester, and um life for me has been pretty uh one word is boring.

Moderator 47:27

All right. Okay. So um let me go ahead and get to the bulk of our questions. So, the COVID-19 pandemic has presented um challenges for undergraduate students across the nation. So as you know, schools have closed, all instruction has moved online, um leaving students and faculty with really little time to prepare. So, um in this study we want to learn more about how the closing of campus, and the pandemic has in general affected you and things that you thought went well that your professors did, um and things that your university did that you found helpful or unhelpful. So we're going to start by um discussing where you went when your campus shut down. So tell me about where you moved to, um or if you stayed on campus and what space you had to do your schoolwork and whether it was public or private.

58 48:16

Okay um I've been living in my parents' house my whole life and right now I still do, so nothing's changed there. And as for where I do my work, I just do it in my room.

Moderator 48:29

Okay. And is that a private space?

58 48:32

Yeah, it's a private space.

Moderator 48:34

Okay. And so, what challenges did you have um doing your schoolwork in that space or attending classes?

58 48:42

I think my main challenge was trying to keep myself focused, because when I was at a at the university environment, I was like, like my mind was separate from my home activities, which I associate with just being leisurely lazy at home. I always did my studying at like the library my homework and stuff like that. So for me it's just been a challenge to really pay attention and get myself motivated to do these things.

Moderator 49:10

Okay. So um what are some things that people did in the house that made it easier for you to attend classes or do your schoolwork, or things that

58 49:19

What was the question?

Moderator 49:19

maybe you came up with? So what are things that you or other people in your house did that made it easier for you to attend classes or do your schoolwork?

58 49:29

Um, mostly just keep to themselves because they have work to do too so it's just pretty quiet in the house. They're there when my parents are there when I need help, too, so it's nice.

Moderator 49:41

All right, so let's talk about responsibilities you had after your campus closed and how these differed from responsibilities that you may have had before your campus closed. So, um, let's first start with caregiving responsibilities like child care, or elder care, or caring for ill loved ones and how these were alike or different from your responsibilities before the shut down. Um did anything change for you?

58 50:05

No cuz I don't have anyone to take care of. I don't have any siblings, or kids, or elderly people, and I just have my dog pretty much. I do the same things just walk my dog, feed him, and stuff like that.

Moderator 50:20

Okay, so um let's talk about household responsibilities then. So, did you have any um chores, cooking, caring for pets during this time and how that was alike or different from before school closed?

58 50:33

Like I said, uh caring for my dog was pretty much the same and then yeah. I've also uh been in charge of cleaning the house, mowing the lawn in the past and uh nothing's changed since then.

Moderator 50:46

Okay. So, um, so let's talk about like the expectations for household work. So, um are you expected by your parents to do like mow the lawn and take care of your dog?

58 51:00

Well I am expected to take care of the dog uh. As for mowing the lawn, I do that with my dad.

Moderator 51:05

Okay.

58 51:05

And then like cleaning I just, I'm just told to uh do the stairs and the living room pretty much since those are the spaces, the living Room especially, I use the most.

Moderator 51:18

Okay. Um so let's talk about your academics and how they were impacted by the shutdown. So I'm gonna ask you about a variety of things. If it if you don't think it impacted you you can just say no impact. So in what ways has the shutdown impacted um your academic performance, um like your grades or your understanding of the subject matter in your courses?

58 51:41

Um I'd say my understanding has decreased compared to before, cuz that element of trying to keep focus so I used more outside resources such as Khan Academy and YouTube tutorials and stuff like that. And uh my grades have stayed the same mostly like high B low A level. Same as before.

Moderator 52:05

Okay, so do you think you were using more resources like Khan Academy just because of um the online class format? Like you just didn't have those available or because like there was something about the online format that made it difficult for you?

58 52:21

Yeah, it was the online format. I just couldn't keep focused on it as much.

Moderator 52:26

Okay. So did the shutdown impact your career goals at all?

58 52:32

Um, I'd say so because I was looking to apply to some internships. Um it was going to be the first time I'd ever tried to go out and get a job or internship, but uhnow with the shutdown there's like I believe there are less opportunities available for that. So I'd say it's impacted it somewhat.

Moderator 52:55

Yeah. Did it change your career goals, I guess?

58 52:59

Um, no, because I still want to be a AI, a machine learning researcher with computer science.

Moderator 53:09

Okay, so um sorry I'm having to adjust my you my dog is moving. So, um, so let's talk about the, um the availability of other learning opportunities. So you did mention internships. Um so were those internships, they didn't go online, they just canceled completely?

58 53:31

Um I wouldn't say they have but I guess that I haven't actually applied to any internships yet, first of all. And um I just I feel like it would be harder to get in but I'm still going to try and apply for these internships. Like, um Lockheed Martin was one of them that I wanted to apply to. But they still have it open.

Moderator 54:00

Okay. Um, so did any new learning opportunities or career development opportunities that you know of emerged during the shutdown and if so, kind of what were they?

58 54:13

Um, I haven't really looked too much into that yet, because I was just starting to research the opportunities.

Moderator 54:21

Okay. So, um, in what ways, if any, has the shutdown impacted your confidence in your ability to successfully complete your bachelor's degree at all or in a timely fashion?

58 54:32

Um it hasn't impacted my confidence at all. I still feel like I can do this. I might not like it as much as I did as going to university but I'm still going to do it and I still feel like I'm fully capable of doing it.

Moderator 54:49

Okay, and in what ways, if any, did the campus shut down impact your finances?

58 54:55

Um it did not. My parents they still have their jobs, just online and I still get my Bright Futures scholarship. So uh pretty much nothing's changed.

Moderator 55:08

Okay. So let's talk about what your professors did and did not do during the move to online learning. So I'd like to know about some examples of strategies, tools, or technologies that your professors used that you found to be very effective um in that they made it easier to learn, or things that you found that were really ineffective, that it made it harder to learn.

58 55:32

Okay. Can this be like from the fall semester or the summer semester?

Moderator 55:37

Um sure.

58 55:39

So like both?

Moderator 55:41

Yeah, I mean so during this time that your campus has moved from um

58 55:46

So during the transition.

Moderator 55:48

Yeah. So stuff that you found that were really helpful or unhelpful.

58 55:52

Okay. Um I thought what was helpful was when the teacher was uh responsive in zoom meetings cuz um at least to the chat cuz I know a lot of people don't like to talk out loud. So, and there was some professors who didn't look at the chat at all so like if people wanted to type a question, and they really wouldn't get it answered, unless it was from other students. Although, um I guess some positives are that the lectures were able to watch later. I like that they were recorded so it gave me more time and I think less pressure really to the, to keep, um keep the schedule.

Moderator 56:41

Okay. So, um, what are some things that your professors did during online learning that made you feel like they cared about you or other students and things that they did that made you feel like they didn't care?

58 56:56

Um, things that I thought when they cared were um, when they were responsive during office hours. Uh cuz um I remember I had one computer science teacher where I um I actually missed the test, due to my negligence to remember the date, but he still made an exception for me to uh take it at a later date. And I really appreciated that from him. And um some things that made me feel they don't care, I don't think I've come across a teacher that I thought didn't care about the students. Yeah.

Moderator 57:39

All right, so let's talk about how your university responded to the move to online learning. Um, I will say, my dog loves other dogs and it is now but dog, um. So what are some things that your university did um pass fail options, town halls, remote counseling, those sorts of things to help students be successful during online learning? And these can be like any resources that your university provided, or other actions they took to help students feel supported, um or felt like they could be successful.

58 58:12

Okay. Well um, I know they added a pass/fail option for the classes, but I didn't get to-- I didn't choose to do that. {dog barking] And um I guess they had a lot of [dog barking] They, they explained their plan as it was going along so it kind of gave some closure as the decisions that the were making and what steps they were gonna take for next semester.

Moderator 58:44

Okay. And so what are some things that you wish your university did to better help student be successful during online learning,

58 58:52

I'm sorry the mic cut out at the last part.

Moderator 58:54

Oh, you're fine. So what do you wish your university did to better help students be successful during the transition to online learning?

58 59:10

I don't, I don't think there's anything I would say that I wish the university did. Just like, I guess, yeah. Actually I think they should teach some of the professors to be more tech savvy, cuz um I know in a lot of the online lectures for some things that don't require technology like say physics or calculus, um some of the professors were spending a lot of time like figuring out the tools, like how to draw something or they accidentally erased the whole thing and they start over. So uh, I feel like they could teach the professor's how to be more tech savvy.

Moderator 59:48

And so in general, are there any other ways um that you were affected by the pandemic in terms of your ability to be successful in school that we haven't discussed?

58 1:00:00

Um I'd say that's about it, just uh I need to focus more and yeah.

Moderator 1:00:11

Okay. Awesome. Those were all my questions. Thank you so much for coming today and staying to answer them. Have a great day. It was nice meeting you.

58 1:00:21

You too. Thank you. Bye.

High SES Hispanic Men Emailed Response Survey 52

During the covid 19 pandemic, I was living with my parents, and commuting to school in the city. In my household, I have my own room with a lock on the door, and access to internet as well as my computer and wifi. This space is private, and if I need to I could announce that I would need to do work and my family would not bother me. While attending school work and doing work in my private space, I'd say the only challenges I faced was being surrounded by so many distractions. In my room I have a variety of video games and entertainment surrounding my workspace, so I often procrastinate. This was really the only issue. In my household, as mentioned before, I would announce that im doing work and do not want to be bothered. They would listen to me and I would be on my own for the next couple of hours unless its for something like eating food or to run a quick errand. I am the oldest in my household, so my parents generally know that I know what im doing. So I am rarely bothered or supported in doing my work. I am not saying that it is a bad thing, but in my house I work very independently. This is probably because my 2 siblings are younger than me, one is 5 and one is 13, so they need more attention and care when it comes to their development in the midst of a pandemic. My caretaking responsibilities include taking care of my younger siblings. I am trusted by my parents for this responsibility and I find myself doing it often because they have to leave the house a lot to run errands. Before and after the shutdown, my responsibilities remained the same, but I had to do them more. Since schools were closed, I had to become my 5 year old’s sister’s teacher for the second half of her kindergarten year. That and babysitting her and my brother sum up most of my responsibilities. After the shut down, there was definitely more expected of me since I am the oldest child. I was expected to do more chores and run more errands considering I had more free time to help my parents. There was less expected of my 13 year old brother, and that kind of bothered me a little bit because back when I was his age I ran many errands. The shutdown has not had too much impact on my academic performance, I was able to keep up my studies and get the same overall grades. The shutdown has impacted my career goals because it gave me more time to reflect on my future and the path i'm taking right now. I had a part time job before the shutdown but it moved to online, so the availability of internships did not affect me. No opportunities emerged during the shutdown The shutdown made me feel more confident about my ability to complete my bachelors because it gave me more time to do work and study and guarantee a pass for my classes. The campus shutting down benefited me financially because I did not have to pay $5.50 daily for MTA transportation or halal food everyday During online learning, some things my professors have been doing haven't been too special. I took five classes and they were all mostly the same. They came in the form of zoom meetings and blackboard assignments Tools that have been ineffective are the assigned tests and quizzes that often have errors during the timed portions. When the test starts it takes me time to even find it online, so I lose precious time. Things that my professors have done that made me feel like they cared about their students was offering one on one help both academically and emotionally. Although I didn't use this service, I think it was cool that they offered it. Some things my professors did that made me feel like they didn't care about their students was lack of communication. I had a professor who did not contact any students until around 2 months after lockdown started. So it kind of just felt like he gave up on the class. After lockdown started, his next and only assignment was the final, which was extremely easy. Some things that my university did during online learning to help included services like online mental health meetings, and the moving of different kinds of services to online. The services included writing and tutoring centers that moved to zoom meetings and personal online meetings. Despite what was just mentioned, I think my university should have had more consistent communication. My financial aid was selected for verification this year, but they haven't gotten back to me since I submitted the documents several months ago. It’s this, and questions about the semester of fall 2020 that are still up in the air and that they never answered for the students. My ability to be successful was only affected through the distractions like I mentioned earlier, through online learning, I think that all students have had to work more independently, and everyone functions differently. Some people work better than others in different environments, and I think that this shutdown has really demonstrated that. Thank you for selecting me for the study, and i'm sorry I could not make it to the zoom meeting. I hope that my answers to the questions provide you with a more in depth understanding of the pandemic’s effect on students.
